# Network Pharmacology Approach to Explore the Potential Mechanisms of Jieduan-Niwan Formula Treating Acute-on-Chronic Liver Failure

**DOI:** 10.1155/2020/1041307

**Published:** 2020-12-30

**Authors:** Jiajun Liang, Mengli Wu, Chen Bai, Chongyang Ma, Peng Fang, Weixin Hou, Xiaoyi Wei, Qiuyun Zhang, Yuqiong Du

**Affiliations:** ^1^School of Traditional Chinese Medicine, Capital Medical University, Beijing 100069, China; ^2^Beijing University of Chinese Medicine, Beijing 100029, China

## Abstract

**Background:**

Acute-on-chronic liver failure (ACLF) is a clinical syndrome with acute jaundice and coagulation dysfunction caused by various inducements on the basis of chronic liver disease. Western medical treatment is limited. Previous studies have confirmed that Jieduan-Niwan Formula (JDNW Formula), an empirical prescription for the treatment of ACLF, can inhibit inflammation and resist hepatocyte apoptosis. However, potential targets and mechanisms still need to be explored.

**Methods:**

In this study, network pharmacological analysis was performed to investigate the key components and potential mechanisms of JDNW Formula treating ACLF. Firstly, we predicted the potential active ingredients of JDNW Formula and the corresponding potential targets through TCMSP, BATMAN-TCM platform, and literature supplement. Then, the ACLF targets database was built using OMIM, DisGeNET, and GeneCard database. Based on the matching targets between JDNW Formula and ACLF, the PPI network was constructed for MCODE analysis and common targets were enriched by Metascape. Furthermore, the ACLF rat model was used to verify the potential mechanism of JDNW Formula in treating ACLF.

**Results:**

132 potential bioactive components of JDNW Formula and 168 common targets were obtained in this study. The enrichment analysis shows that the AMPK signaling pathway was associated with the treating effects of JDNW Formula. Quercetin was hypothesized to be the key bioactive ingredient in JDNW Formula and has a good binding affinity to AMPK based on molecular docking verification. JDNW Formula and quercetin were verified to treat ACLF by regulating the AMPK/PGC-1*α* signaling pathway as a prediction.

**Conclusion:**

The study predicted potential mechanisms of JDNW Formula in the treatment of ACLF, involving downregulation of inflammatory factor expression, antioxidant stress, and inhibition of hepatocyte apoptosis. JDNW Formula may improve mitochondrial quality in ACLF via the AMPK signaling pathway, which serves as a guide for further study.

## 1. Background

Acute-on-chronic liver failure (ACLF) is a clinical syndrome characterized by short-term and high mortality in patients with chronic liver disease who suffer an acute injury within a short period, resulting in acute or subacute decompensation of liver function and intrahepatic and extrahepatic organ failure [1]. The short-term mortality usually exceeds 50% in ACLF patients despite receiving aggressive supportive treatment [2]. Unfortunately, there is still no specific treatment for ACLF. As a definitive treatment, liver transplantation has not been widely used due to the limitation of donor resources and contraindications [3].

However, benefited by the characteristics of multicomponents, multitargets, and multipathways [4], Traditional Chinese Medicine (TCM) has the advantages of good synergism, low side effects, and satisfactory therapeutic effects [5, 6]. TCM therapies, with unique advantages over conventional treatments, are widely used in China and other Asian countries to treat liver disease [7]. *Jieduan-Niwan* Formula (JDNW Formula) is an empirical prescription developed by Professor *Ying Qian*, a nationally celebrated TCM expert, for the treatment of ACLF. This formula consists of ten Chinese herbs, including *Salvia miltiorrhiza* (*Dan Shen*, DS), Astragali Radix (*Huang Qi*, HQ), *Trichosanthes Trichosanthes* (*Gua Lou*, GL), *Phyllanthus amarus* (*Ku Wei Ye Xia Zhu*, KWYXZ), Herba Lysimachiae (*Jin Qian Cao*, JQC), *Viscum coloratum* (*Hu Ji Sheng*, HJS), *Panax notoginseng* (*San Qi*, SQ), Zedoary Rhizome (*E Zhu*, EZ), Rehmanniae Radix (*Sheng Di Huang*, SDH), and Radix Aconiti Lateralis Preparata (*Pao Fu Zi*, PFZ). Through the clinical observation of the efficacy of patients with ACLF, it is found that the comprehensive curative effect of JDNW Formula combined with the western medicine treatment group has an advantage compared with the western medicine treatment group [8]. Our previous studies have demonstrated that JDNW Formula increased the survival ratio of ACLF rats, improved liver function, downregulated the expression of inflammatory factors, and inhibited hepatocytes apoptosis by regulating mitochondrial apoptosis pathway mediated by E2F1 and JNK [9, 10]. However, the effective compounds and potential mechanisms of JDNW Formula in the treatment of ACLF still needed to be further explored.

**Figure 1:**
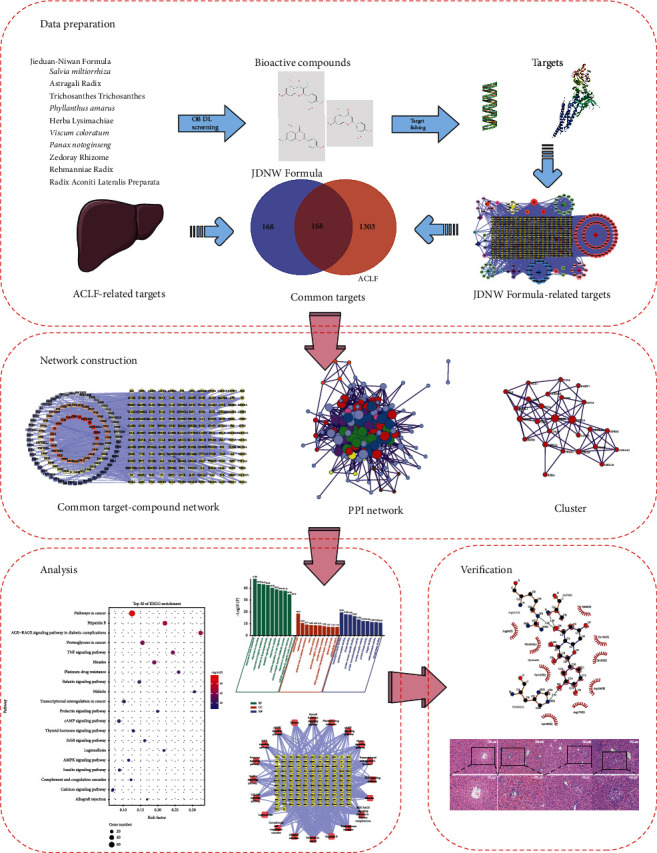
Flow chart of network pharmacology study of JDNW Formula in the treatment of ACLF.

In order to investigate the potential mechanism of JDNW Formula in the treatment of ACLF, the method of network pharmacology was adopted [11, 12]. This methodology abandoned the traditional model of “one drug, one target” and provided us a multidimensional approach to drug discovery, which is in line with the holistic concept of TCM and has similarities with TCM theory [13]. With the rapid development of modern bioinformatics, network pharmacology has become an important tool for the study of TCM. This study investigated the expected targets and biosignaling pathways of JDNW Formula in the treatment of ACLF based on network pharmacology and clarified the potential mechanism. The flow chart of this study is shown in Figure 1.

## 2. Materials and Methods

### 2.1. Screening of Potential Biological Activity Compounds of JDNW Formula

Traditional Chinese Medicine System Pharmacology Database [14] (TCMSP, http://tcmspw.com/tcmsp.php) and a Bioinformatics Analysis Tool for Molecular mechANism of Traditional Chinese Medicine [15] (BATMAN-TCM, http://bionet.ncpsb.org/batman-tcm/) were searched to preliminarily collect the candidate ingredients of ten herbs in JDNW Formula. Then, two ADEM parameters, oral bioavailability (OB) and druglikeness (DL), were selected to screen the potential bioactive compounds in candidate ingredients. OB, a parameter of pharmacokinetics in drug screening, represents the ability of the active ingredient to be absorbed into the system circulation to exert a biological effect [16]. DL, which helps in optimizing pharmacokinetics and drug properties, is used to assess the physicochemical similarity of a compound to a known drug in drug design [17]. Based on the relevant published literature, the ingredients with OB ≥ 30% and DL ≥ 0.18 were screened as bioactive ingredients [18]. Moreover, some other bioactive ingredients with actual targets but in low OB or DL were supplemented according to relevant literature reports [19, 20].

### 2.2. Target Screening

Firstly, the target information of the bioactive ingredients of JDNW Formula was obtained on the TCMSP platform. Besides, the PubChem database [21] (https://pubchem.ncbi.nlm.nih.gov/) was used to search the molecular chemical structure information of the default bioactive ingredients in TCMSP. The target was screened by submitting the molecular chemical structure of bioactive ingredients to Swiss Target Prediction (http://www.swisstargetprediction.ch/), a tool for target prediction based on 2-dimensional and 3-dimensional chemical similarity of ligands [22, 23], and the targets with probability ≥0.6 were screened as potential targets of JDNW Formula.

### 2.3. Disease Targets Database Building

The known targets related to ACLF were integrated by using the key work “Acute-on-Chronic Liver Failure” from Online Mendelian Inheritance in Man [24] (OMIM, http://www.omim.org/), DisGeNET [25] (https://www.disgenet.org/), and GeneCard [26] (https://www.genecards.org/). Disease targets were obtained by deleting duplicate target information. After all the targets were normalizing by gene symbols according to the Uniprot database (https://www.uniprot.org/), the targets of bioactive ingredients of JDNW Formula were matched with ACLF-related targets, and the target network was constructed. The overlapping targets were selected as the relevant target of the JDNW Formula for treating ACLF.

### 2.4. Network Construction and Analysis

To explore the intrinsic interactions between potential bioactive compounds and corresponding targets systematically, network construction was established by Cytoscape software [27] (Version 3.8.0) as follows: (1) herbs-compounds-targets network, (2) ACLF targets-JDNW Formula targets network, (3) common targets-compounds network, and (4) target-pathway network. Finally, the importance of nodes in networks was assessed by the topological parameter, “Degree,” which is defined as the number of links to a node [28]. The more important a node is, the higher value it outputs.

### 2.5. Protein-Protein Interaction (PPI) Network Construction and Enrichment Analysis

The common targets of JDNW Formula and ACLF were summited to Metascape [29] (http://metascape.org/gp/index.html), a platform with fast data updating, wide coverage, integration of multiple database resources, and rich annotation, to perform PPI network construction, Gene Ontology (GO), and KEGG pathway enrichment analysis. After constructing the PPI network, common targets were grouped into corresponding clusters according to the Molecular Complex Detection (MCODE) algorithm in Metascape. By setting min overlap = 3, min enrichment = 1.5, *P* ≤ 0.01, biological processes (BPs), molecular functions (MFs), cellular components (CCs), and metabolic pathways which the common genes involved were obtained.

### 2.6. Molecular Docking

CB-Dock (http://cao.labshare.cn/cb-dock/) is a web server for blind docking for cavity detection based on the widely used docking tool AutoDock Vina. The benchmark tests show that CB-Dock is superior to other state-of-the-art blind docking tools in predicting binding sites and binding conformations [30]. Quercetin was selected to dock with AMPK based on a network pharmacology study. MOL2 format files of quercetin were obtained from the TCMSP database and PDB format files of key targets were derived from the RCSB database [31] (http://rcsb.org). The files of ligand and receptor were input to CB-Dock to elevate the binding activities. The binding models were visualized using CB-Dock and Ligplot (Version 2.2) [32].

### 2.7. Experiment Validation

#### 2.7.1. Chemical Drug and Agents

Quercetin (Que, Q4951), human serum albumin (HSA, A9731-5G), D-galactosamine (D-GalN, G0500-25G), and lipopolysaccharide (LPS, L3012) were purchased from SigmaAldrich (St. Louis, MO, USA).

#### 2.7.2. Animals and Models

30 male Wistar rats, weighing 180–200 g, were housed in an SPF environment with normal water and food. All procedures were approved by the Animal Experiments and Experimental Animal Welfare Committee of Capital Medical University. The rats were randomly divided into 4 groups: normal control group (NC, *n* = 6), ACLF model group (ACLF, *n* = 8), JDNW Formula treatment group (JDNWF, *n* = 8), and quercetin treatment group (Que, *n* = 8). Rats were adaptively housed for 5 days before the experiment. As described in previous studies [10], rats were induced into immunological hepatic fibrosis using HSA. 6 weeks later, rats were given coadministration of 400 mg/kg D-GalN and 100 *μ*g/kg LPS intraperitoneal injection for the acute attack to construct the ACLF model. Rats in the NC group were given the same volume of normal saline instead. Before giving D-GalN/LPS, rats in the JDNWF group were given 21.7 g/kg JDNW Formula decoction and those in the Que group were given 100 mg/kg quercetin by intragastric administration for 7 days.

After 12 h of an acute attack, all rats were sacrificed. The right lobe of the liver was fixed in 10% neutral formalin buffer for pathological staining, and the rest of the tissues were stored at −80°C for further use.

#### 2.7.3. Liver Histology Observation

Formalin-fixed liver tissues were embedded in paraffin and stained with hematoxylin-eosin (HE). The stained samples were scanned by Pannoramic SCAN (3D HISTECH) at 400×.

#### 2.7.4. Western Blot Analysis

Liver tissues in each group were disrupted in lysis buffer and the supernatant was collected after centrifugation. The protein concentration of each supernatant was determined by BCA protein assay. Equal amounts of protein were separated by 10% sodium dodecyl sulfate-polyacrylamide gel (SDS-PAGE) electrophoresis and electrophoretically transferred to PVDF membranes (Millipore, USA). After blocking with 5% nonfat dry milk, bands were incubated overnight at 4°C with appropriate primary antibodies against PGC-1*α* (Proteintech, USA), p-AMPK (Cell Signal Technology, USA), and GAPDH (Santa Cruz Biotechnology, USA). Then, the membranes were incubated with corresponding secondary antibodies, including Donkey Anti-Mouse IgG (Proteintech, USA) and Goat Anti-Rabbit IgG (Lablead, China) at room temperature. Immunoreactivity was detected by electrochemiluminescence (ECL) reagent (Millipore, USA). The levels of protein expression were normalized relative to GAPDH. Quantitative analysis was performed by ImageJ software.

### 2.8. Statistical Analyses

The experiment data were analyzed using GraphPad software (Prism 9.00). All the results were presented as mean ± standard deviation (SD) and analyzed by one-way ANOVA. The least-significant difference (LSD) method was applied when the variance was equal; otherwise, Tamhane's T2 test was performed. *P* values < 0.05 were considered to be statistically significant.

## 3. Results

### 3.1. Potential Active Components in JDNW Formula

Through TCMSP, BATMAN-TCM, and literature mining, the ingredients of the JDNW Formula were initially obtained. Further, screening by two ADEM parameters (OB ≥ 30%, DL ≥ 0.18) and supplementing with active compounds with actual targets but in low OB or DL, 148 compounds were collected and were considered as potential active ingredients, including 59, 17, 10, 20, 10, 6, 7, 2, 10, and 7 from *Dan Shen*, *Huang Qi*, *Gua Lou*, *Ku Wei Ye Xia Zhu*, *Jin Qian Cao*, *Hu Ji Sheng*, *San Qi*, *E Zhu*, *Sheng Di Huang*, and *Pao Fu Zi*. After the deletion of duplicate values, 132 ingredients of JDNW Formula were eventually collected as potential active compounds and shown in Supplementary Table S1.

### 3.2. Targets of JDNW Formula and ACLF

**Figure 2:**
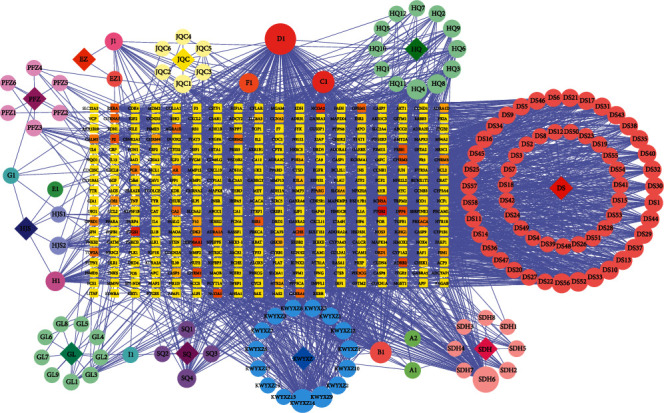
The compound-target network of JDNW Formula that consists of 478 nodes and 2206 edges. Circle and round square nodes denote the compounds and targets, respectively.

**Figure 3:**
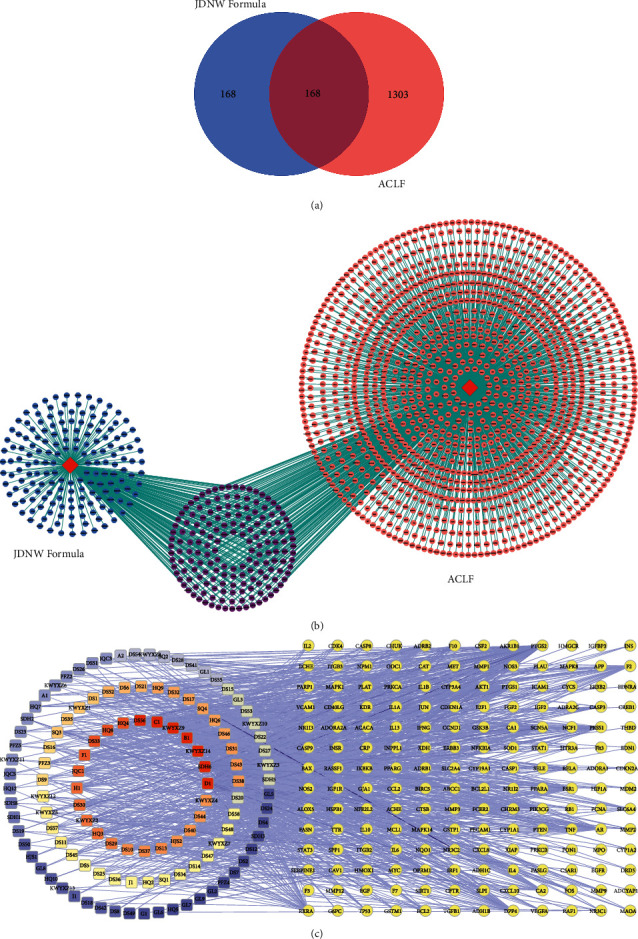
JDNW Formula and ACLF-related targets and overlapping targets. (a) Venn diagram showing 168 related targets of JDNW Formula and ACLF-related targets were shared. (b) The network of JDNW Formula and ACLF. (c) The network of compounds and common targets. Circle and round square nodes denote the targets and compounds, respectively. The node color changing from blue to red reflects that the degree value changes from low to high.

By combining known targets and chemical similarity predictions, a total of 336 corresponding potential targets were identified from 132 potential active ingredients of JDNW Formula (Supplementary Materials: Table S2). The Compound-Target network of JDNW Formula consisted of 478 nodes and 2206 edges (Figure 2). 1929 ACLF-related targets were obtained in the GeneCards database. The high score indicates that the ACLF is more relevant. Therefore, the targets with a score greater than the median were selected to supplement with OMIM and DisGeNET database. As a result, 1471 ACLF target genes were obtained after the deletion of duplicate values (Supplementary Materials: Table S3). To clarify the interaction between potential targets of JDNW Formula and ACLF-related targets, the intersection of both targets was draw by mapping the Venn diagram and constructing the targets network (Figures 3(a) and 3(b)). 168 common targets were shared between potential targets of JDNW Formula and ACLF-related targets (Supplementary Table S4). Finally, the compounds-common targets network was constructed to search for potential active compounds that are more closely related to the common targets (Figure 3(c)). The potential active compounds were ranked by degree value (Supplemental Materials: Table S5), and the node with the highest degree value was quercetin (degree = 105) after calculating.

### 3.3. Protein-Protein Interaction (PPI) Network Targets and Analysis

**Figure 4:**
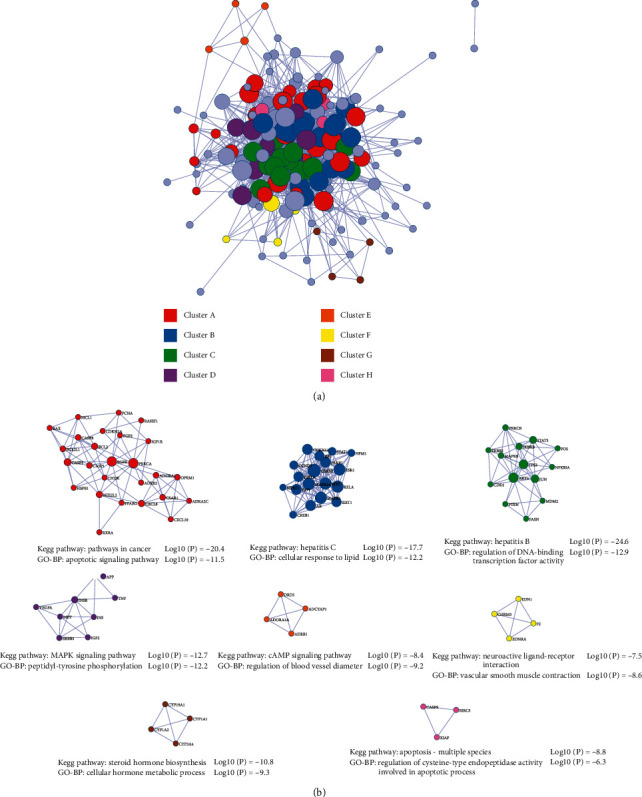
Protein-protein interaction (PPI) network of common targets between JDNW Formula and ACLF. (a) PPI network and (b) eight clusters with the corresponding description from the PPI network.

In the Metascape platform, 168 common targets were submitted to construct the PPI network using default parameters. The PPI network consists of 161 nodes and 1292 edges (Figure 4(a)). All nodes were arranged in descending order with degree value, with top 10 targets containing JUN (63), TP53 (61), EGFR (59), MAPK1 (58), AKT1 (57), RELA (56), GSK3B (54), MYC (51), PRKCA (50), and STAT3 (49) (Table 1). The targets in the network were grouped into 8 clusters with the MCODE algorithm in Metascape. The KEGG pathway and GO biological process analysis about these targets were shown as the functional description of the corresponding clusters independently (Figure 4(b)).

### 3.4. GO and KEGG Pathway Enrichment Analysis

**Figure 5:**
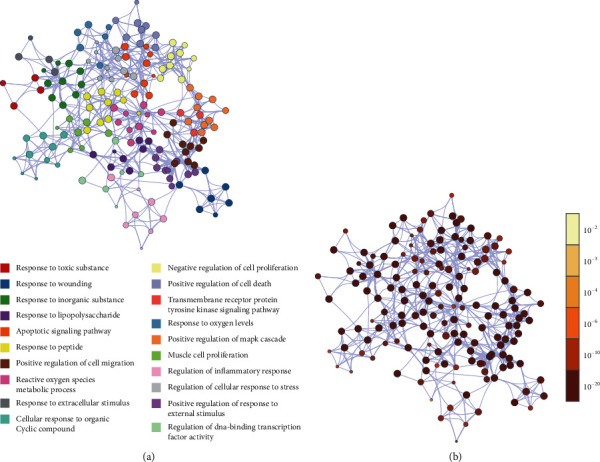
GO-BP enrichment network of 168 targets. (a) Colored based on BP clusters, where nodes of the same cluster are highly correlated with each other. (b) Colored by *P* value, where the darker the color, the more significant the *P* value.

**Figure 6:**
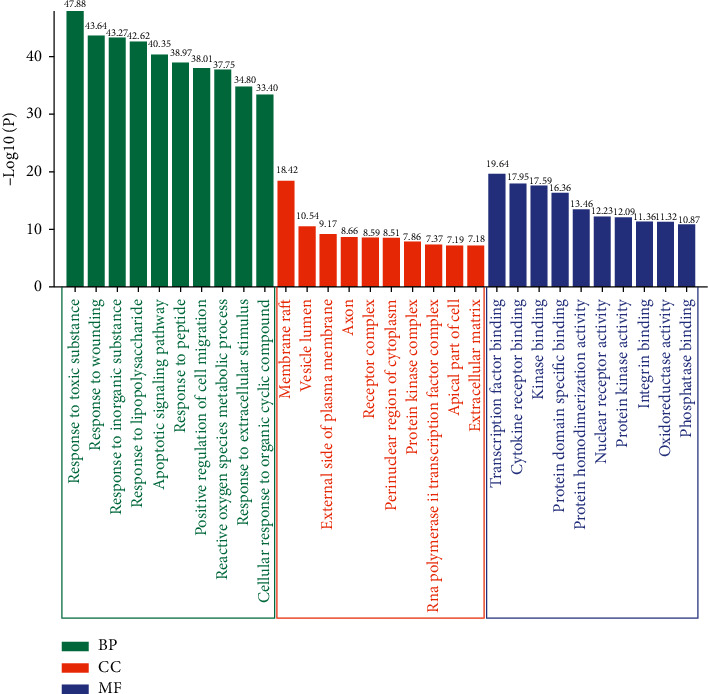
The GO enrichment of 168 targets of JDNW Formula. The top 10 items for each section were listed separately.

Metascape platform was used for enrichment analysis of 168 common targets, and the results with min overlap = 3, min enrichment = 1.5, and *P* < 0.01 were screened for clarifying the biological process (BP), cellular components (CCs), molecular function (MF), and molecular pathway involved in the treatment of ACLF with JDNW Formula (Supplementary Materials: Table S6). Terms were grouped into clusters based on their membership similarities in Metascape. As the GO-BP network shown in Figure 5, GO enrichment analysis revealed that the 168 common targets were involved in various biological processes, mainly including response to a toxic substance, response to lipopolysaccharide, apoptotic signaling pathway, positive regulation of cell migration, reactive oxygen species metabolic process, regulation of cell proliferation and death, response to oxygen levels, positive regulation of MAPK cascade, regulation of inflammatory response, and regulation of cellular response to stress. The above processes were related to various molecular functions, mainly including transcription factor binding, cytokine receptor binding, kinase binding, protein domain specific binding, nuclear receptor activity, oxidoreductase activity, and phosphatase binding. These biological processes occurred primarily in areas including membrane raft, vesicle lumen, external side of the plasma membrane, receptor complex, perinuclear region of cytoplasm, and extracellular matrix (Figure 6).

**Figure 7:**
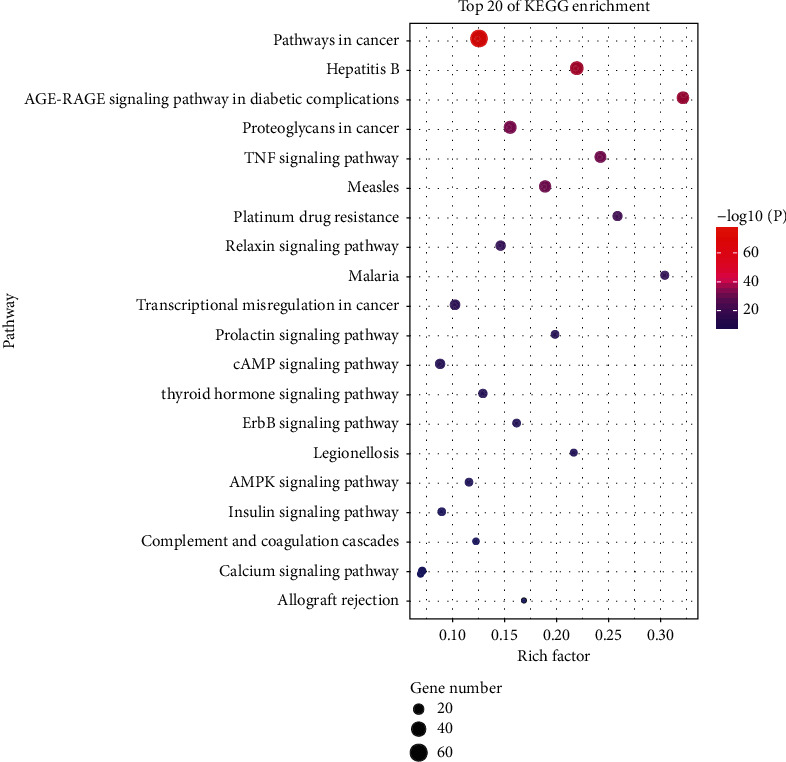
The KEGG enrichment analysis of the top 20 metabolic pathways.

**Figure 8:**
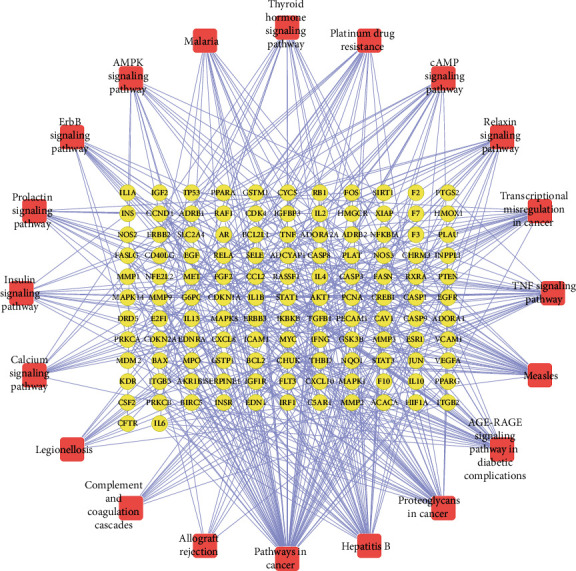
The network of targets involved in the major pathways. Circle and round square nodes denote the targets and signaling pathways, respectively.

KEGG pathway enrichment analysis revealed that key targets were enriched to a total of 357 pathways (Supplementary Materials: Table S7). Ranking by *P* value, the top 20 pathways are shown in Figure 7 and Table 2. According to the analysis result, these 168 targets were mainly enriched in pathways in cancer, hepatitis B, AGE-RAGE signaling pathway, TNF signaling pathway, cAMP signaling pathway, AMPK signaling pathway, and calcium signaling pathway, which are probably associated with ACLF. Top 20 pathways were imported into Cytoscape for visualization to obtain a network of the targets involved in the major pathways (Figure 8).

### 3.5. Molecular Docking Verification

**Figure 9:**
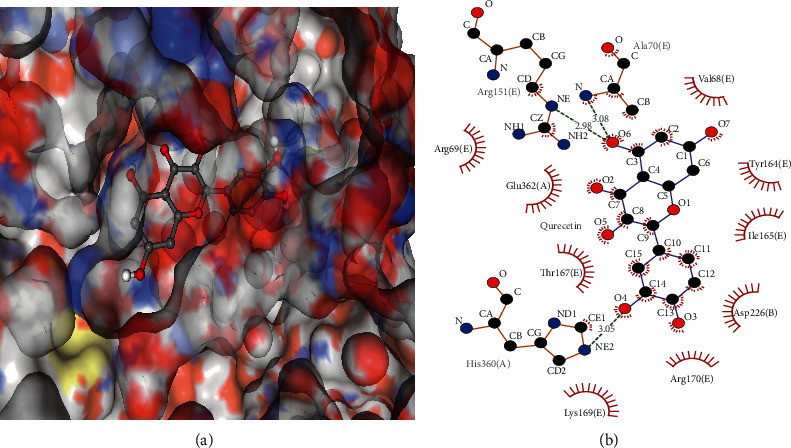
The molecular docking simulation of AMPK-quercetin. 3D figures (a) show the molecular model of quercetin in the binding pocket of the protein. 2D figures (b) show the interactions between quercetin and surrounding binding sites. Hydrogen bonds were displayed as green dashed lines. Hydrophobic interactions are indicated as red opposite arcs.

A molecular docking model of quercetin with AMPK (PDB ID: 4CFH) was performed on CB-Dock using default parameters (Figure 9(a)). Vina score, the binding energy, indicates the binding activity between the proteins and compounds [33]. The binding energy of the docking model was −8.3 kcal/mol. The lower the score value, the more stable the binding of the compound to the target. The result suggests the potential of binding between quercetin and AMPK. The molecular docking visualization is shown in Figure 9(b). The proteins-ligands two-dimensional interaction maps show that quercetin forms hydrogen bonds with several amino acid residues, including Ala70(E), Arg151(E), His360(A), and hydrophobic binding with Val68(E), Try164(E), Ile165(E), Asp226(B), Arg170(E), Lys169(E), Thr167(E), Glu362(A), and Arg69(E).

### 3.6. Experiment Validation

#### 3.6.1. Liver Histology Observation

**Figure 10:**
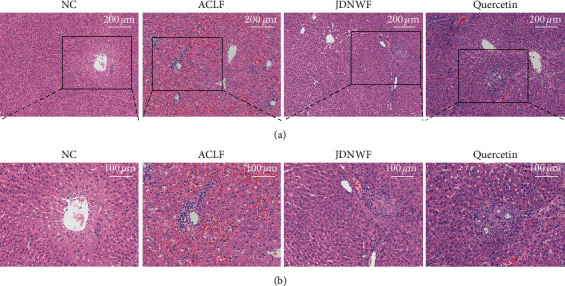
The histopathological changes in each group by HE staining. (a) Magnification ×400. (b) Magnification ×800.

In the NC group, the hepatic cords in the liver were regularly arranged and structurally intact. The morphology of hepatocytes was normal, without inflammatory cell infiltration. In the ACLF group, hepatic lobular structures were destroyed, and the hepatic sinusoid also has obvious hyperemia. Hepatocytes are seen to be swollen and necrotic, with the loss of the nucleus. There is a large amount of inflammatory cell infiltration in the portal area and around the focal point of hepatocellular necrosis. However, lighter inflammatory cell infiltration can be seen in the JDNWF group and quercetin group, accompanied by hepatic fibrous tissue hyperplasia and regular hepatic cord structures (Figure 10).

#### 3.6.2. Validation of the Expression of Pathway-Related Proteins

**Figure 11:**
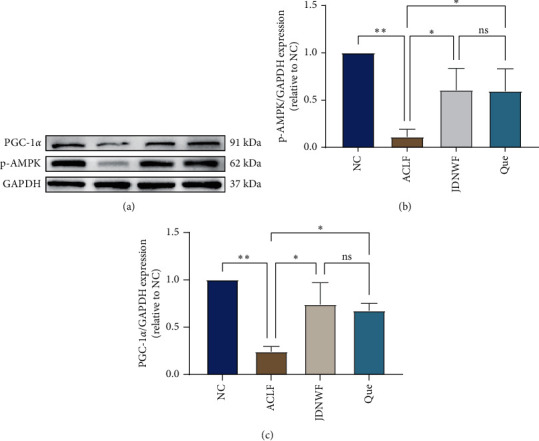
Effect of JDNW Formula and quercetin on the AMPK signaling pathway in ACLF rats. (a) Representative immunoblots for the p-AMPK, PGC-1*α,* and GAPDH proteins. (b) The relative expression levels of p-AMPK/GAPDH and PGC-1*α*/GAPDH. The data on quantified protein expressions were normalized by related GAPDH (fold change of NC). Data are presented as the mean ± SD (^*∗*^*p* < 0.05, ^*∗∗∗*^*p* < 0.001). The blots shown are representative of 3 independent experiments.

In order to investigate whether JDNW Formula and quercetin have a moderating effect on the AMPK signaling pathway, as predicted by network pharmacology and molecular docking, the protein levels of phospho-AMPK (p-AMPK) and PGC1-*α* were evaluated by Western Blot. Compared with the NC group, the level of p-AMPK and PGC-1*α*, the downstream signaling molecule, was inhibited in the ACLF group. However, JDNW Formula and quercetin treatment attenuated this decrease (Figure 11).

## 4. Discussion

ACLF is a clinical syndrome with acute jaundice and coagulation dysfunction caused by various inducements on the basis of chronic liver disease. It can be complicated with hepatic encephalopathy, ascites, electrolyte disturbance, infection, hepatorenal syndrome, and even extrahepatic multiple organ failure [34]. ACLF is associated with a high short-term mortality rate. For ACLF patients, lacking of specific western medical treatment, the most direct and effective treatment is liver transplantation. However, TCM has unique advantages in the treatment of ACLF. JDNW Formula is an empirical prescription developed by Professor Qian Ying, a nationally celebrated TCM expert, for the treatment of ACLF with good clinical efficacy. TCM prescription has the characteristics of multicomponents, multitargets, and multipathways. It is difficult to explain its pharmacological mechanism due to its properties. Therefore, in this study, for the first time, network pharmacology was used to analyze the network molecular mechanism of the JDNW Formula and to investigate the potential mechanism of this prescription in the treatment of ACLF.

By mining the database, 132 potential bioactive ingredients were identified in JDNW Formula, 1471 ACLF-related targets, and 168 common targets shared in JDNW Formula and ACLF. The node of quercetin, with the highest degree value in the compound-common targets network, was presumed to be the potential active ingredient that plays a key role in the mechanisms of the JDNW Formula. In fact, quercetin, with hepatoprotective effects, exhibits strong antioxidant activity and anti-inflammatory effects [35] and inhibits hepatocyte apoptosis in liver injury [36, 37]. Besides, mitochondria are considered an important intracellular target of quercetin. The mechanism is related to the regulation of mitochondrial biogenesis, modulation of oxidative respiration, and inhibition of mitochondria-induced apoptosis [37].

As the PPI network of common targets showed, JUN, TP53, EGFR, MAPK1, AKT1, RELA, GSK3B, MYC, PRKCA, and STAT3 were the top 10 targets ranking by degree, which indicated that JDNW Formula may play a potential role in improving ACLF by regulating these targets. What is more, targets that are highly correlated with each other were grouped into the same cluster with a corresponding description of GO-BP and KEGG pathway enrichment analysis to explore the biological mechanisms, suggesting that the therapeutic mechanism of JDNW Formula in ACLF is a combination of multiple targets. According to the network, targets of JDNW Formula for treating ACLF are involved in the responses to oxidative stress, responses to the pathogen-associated molecular pattern (PAMP), regulation of inflammatory responses, apoptotic pathways, and cell proliferation. In fact, our previous study has proved that JDNW Formula can alleviate ACLF by regulating JNK-induced mitochondrial apoptotic pathway and E2F1-mediated intrinsic apoptosis pathway in vivo [9, 10].

From the results of the present study, based on the KEGG enrichment analysis, the potential pathways of JDNW Formula in the treatment of ACLF were closely related to pathways in cancer, hepatitis B, TNF signaling pathway, cAMP signaling pathway, AMPK signaling pathway, and calcium signaling pathway. HBV (enriched in cluster C) and HCV (enriched in cluster B) infection and reactivation are important causes of ACLF. HCV can be recognized by RIG-I-like receptors and Toll-like receptors. It can activate natural killer cells and promote the expression of TNF-*α* by inducing the secretion of IFN-1 and IFN-3 [38]. However, HBV infection does not directly cause hepatocyte damage. It induces the activation and infiltration of innate immune cells, leading to hepatocyte apoptosis and accumulation of damage-associated molecular pattern (DAMP). Endogenous DAMPs act on Toll-like receptors or activate NF-*κ*B to further transmit inflammatory signals [39, 40]. TNF is an important inflammatory factor secreted by Kupffer cells in the inflammatory process of ACLF, which is involved in the secretion of many inflammatory factors and regulates NF-*κ*B and MAPK signaling pathways. The level of TNF is related to the severity and prognosis of ACLF [41]. In our previous study, it has been confirmed that JDNW Formula can reduce inflammatory factors such as TNF-*α* and IL-6 [10]. By regulating the pathways or related targets like PI3K/Akt pathway, GSK3, JNK, and AMPK, the cAMP signaling pathway is involved in hepatic physiological and pathological processes, including lipid metabolism, inflammation, oxidative stress, endoplasmic reticulum stress, and hepatocyte apoptosis [42]. As a key factor in the regulation of cellular energy balance and mitochondrial quality control, the AMPK signaling pathway controls mitochondrial biogenesis, mitochondrial dynamics, and mitophagy, which help to improve mitochondrial function [43]. The calcium signaling pathway leads to mitochondria damage, releases ROS, and finally stimulates NLRP3 inflammasome activation [44].

It is worth mentioning that the cancer pathway is also important in the treatment of ACLF. Because of the pathology of massive hepatocyte necrosis in ACLF, the treatment goal of ACLF patients is to protect hepatocytes and promote hepatocyte proliferation. Studies have shown that the transformation from fatty acid oxidation to glycolysis can produce effects similar to promoting the proliferation and apoptosis escape in tumor cells and improve the survival of hepatocytes under hypoxic and hyperammonemia conditions [45–47]. The KEGG pathway enrichment analysis further confirmed that the potential mechanism of JDNW Formula is based on the synergistic effect of multiple targets and multiple signaling pathways.

As discussed above, the enrichment pathway mainly involves the regulation of inflammation, oxidative stress, lipid metabolism, apoptosis, and cell proliferation. Mitochondrial dysfunction is closely related to inflammatory response, oxidative stress, and lipid metabolism [48, 49]. Research on blood metabolomics also revealed that there is significant mitochondrial dysfunction in patients with ACLF, leading to the development of organ failure [1]. Combined with the network pharmacology results and our previous study [9], it can be inferred that the main mechanism of JDNW Formula treating ACLF is probably to downregulate the expression of inflammatory factors, against oxidant stress, so as to inhibit hepatocyte apoptosis and promote regeneration of hepatocytes. What is more, protecting mitochondria may be a potential mechanism of JDNW Formula treating ACLF. As KEGG enrichment analysis indicated, the most relevant pathway may be the AMPK signaling pathway.

AMPK, a major regulator of mitochondria, can induce expression and acetylation of its downstream effector PGC-1*α* to promote mitochondrial biogenesis and ultimately maintain mitochondrial homeostasis [43, 50]. The molecular docking result also shows that quercetin exhibited good affinity to AMPK, which is related to hydrogen bonds with Ala70(E), Arg151(E), and His360(A). The bonding may induce the activation of AMPK downstream signal transduction to improve mitochondrial quality. Thus, a D-GalN/LPS-induced ACLF rat model was established to confirm the anti-ACLF effect of JDNW Formula and quercetin. The Liver pathological section shows that JDNW Formula and quercetin can reduce inflammatory infiltrate and hepatocellular necrosis in the liver. To further investigate the potential treatment of whether the AMPK signaling pathway was involved in the mechanism or not, protein expression of p-AMPK and PGC-1*α* was detected. In the present study, JDNW Formula and quercetin can attenuate the decrease of AMPK phosphorylation levels and the expression of PGC-1*α* in the ACLF group, suggesting that JDNW Formula and quercetin can modulate the AMPK/PGC-1*α* signaling pathway to treat ACLF.

In this study, the means of network pharmacology was used to explore the effects of JDNW Formula in the treatment of ACLF at the molecular level and explained the possible mechanisms of its effects. Except for the anti-inflammatory effects and inhibition of apoptosis, which we have proved, our study predicted that protecting mitochondria may be a potential mechanism of JDNW Formula for ACLF. Quercetin and the AMPK signaling pathway could be the key to the mechanism. However, there were still some limitations. For example, the potential active ingredients are screened primarily by databases using ADME parameters. Although the components were supplemented by reviewing the literature, there is no denying that certain ingredients will be omitted. In addition, quercetin and other potentially active compounds should be verified in subsequent studies and metabolomic analysis, for example, to find biomarkers.

## 5. Conclusion

In this study, the potential active compounds, genes, and the signaling pathway that may play a key role in the biological process were predicted, screened, and analyzed. Quercetin was presumed to be the key active compound of the JDNW Formula. It was elucidated that the possible mechanism of JDNW Formula treating ACLF is the synergistic effect of “multicomponents, multitargets, and multipathways,” including downregulation of inflammatory factor expression and antioxidant stress, inhibiting hepatocyte apoptosis, and improving mitochondrial quality. According to the enrichment analysis, JDNW Formula may improve mitochondrial quality in ACLF via the AMPK signaling pathway, which needs further study. More importantly, the results of this study provide a theoretical direction for the JDNW Formula in the treatment of ACLF. Further experimental validation is still necessary to identify the key active ingredients in the JDNW Formula and to investigate how this formula acts as a therapeutic agent for ACLF through relevant pathways.

## Figures and Tables

**Table 1:** The information of the top 10 common targets between JDNW Formula and ACLF.

Uniprot ID	Gene symbol	Description	Degree
P05412	JUN	Transcription factor AP-1	63
P04637	TP53	Cellular tumor antigen p53	61
P00533	EGFR	Epidermal growth factor receptor	59
P28482	MAPK1	Mitogen-activated protein kinase 1	58
P31749	AKT1	RAC-alpha serine/threonine-protein kinase	57
Q04206	RELA	Transcription factor p65	56
P49841	GSK3B	Glycogen synthase kinase-3 beta	54
P01106	MYC	Myc proto-oncogene protein	51
P17252	PRKCA	Protein kinase C alpha type	50
P40763	STAT3	Signal transducer and activator of transcription 3	49

**Table 2:** The information of top 20 pathways.

GO ID	Description	Gene number	*P* value	Rich factor
hsa05200	Pathways in cancer	72	2.52152E-76	0.125217391
hsa05161	Hepatitis B	39	2.54498E-50	0.220338983
ko04933	AGE-RAGE signaling pathway in diabetic complications	32	2.4597E-47	0.323232323
hsa05205	Proteoglycans in cancer	34	2.48392E-38	0.155963303
hsa04668	TNF signaling pathway	28	1.72293E-37	0.243478261
hsa05162	Measles	30	1.40858E-36	0.189873418
hsa01524	Platinum drug resistance	19	2.58784E-26	0.260273973
hsa04926	Relaxin signaling pathway	20	2.80746E-22	0.147058824
ko05144	Malaria	15	3.29022E-22	0.306122449
hsa05202	Transcriptional misregulation in cancer	21	5.13459E-20	0.102941176
ko04917	Prolactin signaling pathway	14	6.54424E-18	0.2
hsa04024	cAMP signaling pathway	20	8.3844E-18	0.088495575
hsa04919	Thyroid hormone signaling pathway	16	3.71782E-17	0.130081301
ko04012	ErbB signaling pathway	14	1.4152E-16	0.162790698
ko05134	Legionellosis	12	5.14222E-16	0.218181818
ko04152	AMPK signaling pathway	14	1.75187E-14	0.116666667
hsa04910	Insulin signaling pathway	14	6.32339E-13	0.090322581
hsa04610	Complement and coagulation cascades	11	6.37071E-12	0.123595506
ko04020	Calcium signaling pathway	13	8.49125E-11	0.071428571
hsa05330	Allograft rejection	8	3.66628E-10	0.170212766

## Data Availability

The datasets used and/or analyzed during the current study are available from the corresponding author on reasonable request.

## Abbreviations

ACLF: Acute-on-chronic liver failure
TCM: Traditional Chinese Medicine
JDNW Formula: *Jieduan-Niwan* Formula
OB: Oral bioavailability
DL: Druglikeness
PPI: Protein-protein interaction
MCODE: Molecular Complex Detection
GO: Gene Ontology
BP: Biological processes
CC: Cellular components
DS: *Salvia miltiorrhiza*
HQ: Astragali Radix
GL: *Trichosanthes Trichosanthes*
KWYXZ: Phyllanthus Amarus
JCQ: Herba Lysimachiae
HJS: *Viscum coloratum*
SQ: *Panax notoginseng*
EZ: Zedoary Rhizome
SDH: Rehmanniae Radix
PFZ: Radix Aconiti Lateralis Preparata
PAMP: Pathogen-associated molecular pattern
DAMP: Damage-associated molecular patterns.
MF: Molecular funtions
QUE: Quercetin.

## Supplementary Materials

Supplementary Materials Supplementary Material 1: Table S1: information of potentially bioactive compounds of Jieduan-Niwan Formula. Supplementary Material 2: Table S2: the details of targets from compounds in JDNW Formula. Supplementary Material 3: Table S3: 1471 known ACLF-related targets. Supplementary Material 4: Table S4: 168 potential targets shared in JDNW Formula and ACLF. Supplementary Material 5: Table S5: information of potentially bioactive compounds of 168 common targets. Supplementary Material 6: Table S6: GO cellular component enrichment analysis of key targets of JDNW Formula in the treatment of ACLF. Supplementary Material 7: Table S7: KEGG pathway enrichment analysis of key targets of JDNW Formula in the treatment of ACLF.
