# Supplementary material for: Network Pharmacology Approach to Explore the Potential Mechanisms of Jieduan-Niwan Formula Treating Acute-on-Chronic Liver Failure
Source: Evid Based Complement Alternat Med. 2020 Dec 30;2020:1041307. doi: 10.1155/2020/1041307 (PMC7787753; doi:10.1155/2020/1041307)
Supplement: Supplementary Materials — Supplementary Material 1: Table S1: information of potentially bioactive compounds of Jieduan-Niwan Formula. Supplementary Material 2: Table S2: the details of targets from compounds in JDNW Formula. Supplementary Material 3: Table S3: 1471 known ACLF-related targets. Supplementary Material 4: Table S4: 168 potential targets shared in JDNW Formula and ACLF. Supplementary Material 5: Table S5: information of potentially bioactive compounds of 168 common targets. Supplementary Material 6: Table S6: GO cellular component enrichment analysis of key targets of JDNW Formula in the treatment of ACLF. Supplementary Material 7: Table S7: KEGG pathway enrichment analysis of key targets of JDNW Formula in the treatment of ACLF. [file 1041307.f1.zip › 1041307.f1/Table S1.1041307.v2 (1).docx]

| **Information of potential bioactive compounds of Jieduan-Niwan Formula** | | | | |
| --- | --- | --- | --- | --- |
| **ID** | **Molecule name** | **OB** | **DL** | **Herb** |
| A1 | vanillic acid | 35.47 | 0.04 | SDH, KWYXZ |
| A2 | oleanolic acid | 29.02 | 0.76 | SDH, KWYXZ |
| B1 | luteolin | 36.16 | 0.25 | DS, KWYXZ |
| C1 | kaempferol | 41.88 | 0.24 | HQ, JQC, KWYXZ |
| D1 | quercetin | 46.43 | 0.28 | HQ, JQC, KWYXZ, SQ |
| E1 | Mairin | 55.38 | 0.78 | HQ, HJS |
| F1 | isorhamnetin | 49.6 | 0.31 | HQ, JQC, HJS |
| G1 | sitosterol | 36.91 | 0.75 | JQC, HJS, PFZ |
| H1 | beta-sitosterol | 36.91 | 0.75 | HJS, SQ |
| I1 | Mandenol | 42 | 0.19 | GL, SQ |
| J1 | hederagenin | 36.91 | 0.75 | HQ, EZ |
| DS1 | 1,2,5,6-tetrahydrotanshinone | 38.75 | 0.36 | DS |
| DS2 | Poriferasterol | 43.83 | 0.76 | DS |
| DS3 | poriferast-5-en-3beta-ol | 36.91 | 0.75 | DS |
| DS4 | isoimperatorin | 45.46 | 0.23 | DS |
| DS5 | sugiol | 36.11 | 0.28 | DS |
| DS6 | Dehydrotanshinone II A | 43.76 | 0.4 | DS |
| DS7 | Baicalin | 40.12 | 0.75 | DS |
| DS8 | digallate | 61.85 | 0.26 | DS |
| DS9 | 5,6-dihydroxy-7-isopropyl-1,1-dimethyl-2,3-dihydrophenanthren-4-one | 33.77 | 0.29 | DS |
| DS10 | 2-isopropyl-8-methylphenanthrene-3,4-dione | 40.86 | 0.23 | DS |
| DS11 | 3α-hydroxytanshinoneⅡa | 44.93 | 0.44 | DS |
| DS12 | (E)-3-[2-(3,4-dihydroxyphenyl)-7-hydroxy-benzofuran-4-yl]acrylic acid | 48.24 | 0.31 | DS |
| DS13 | 4-methylenemiltirone | 34.35 | 0.23 | DS |
| DS14 | 2-(4-hydroxy-3-methoxyphenyl)-5-(3-hydroxypropyl)-7-methoxy-3-benzofurancarboxaldehyde | 62.78 | 0.4 | DS |
| DS15 | formyltanshinone | 73.44 | 0.42 | DS |
| DS16 | 3-beta-Hydroxymethyllenetanshiquinone | 32.16 | 0.41 | DS |
| DS17 | Methylenetanshinquinone | 37.07 | 0.36 | DS |
| DS18 | przewalskin a | 37.11 | 0.65 | DS |
| DS19 | przewalskin b | 110.32 | 0.44 | DS |
| DS20 | Przewaquinone B | 62.24 | 0.41 | DS |
| DS21 | przewaquinone c | 55.74 | 0.4 | DS |
| DS22 | (6S,7R)-6,7-dihydroxy-1,6-dimethyl-8,9-dihydro-7H-naphtho[8,7-g]benzofuran-10,11-dione | 41.31 | 0.45 | DS |
| DS23 | przewaquinone f | 40.31 | 0.46 | DS |
| DS24 | sclareol | 43.67 | 0.21 | DS |
| DS25 | tanshinaldehyde | 52.47 | 0.45 | DS |
| DS26 | Danshenol B | 57.95 | 0.56 | DS |
| DS27 | Danshenol A | 56.97 | 0.52 | DS |
| DS28 | Salvilenone | 30.38 | 0.38 | DS |
| DS29 | cryptotanshinone | 52.34 | 0.4 | DS |
| DS30 | dan-shexinkum d | 38.88 | 0.55 | DS |
| DS31 | danshenspiroketallactone | 50.43 | 0.31 | DS |
| DS32 | deoxyneocryptotanshinone | 49.4 | 0.29 | DS |
| DS33 | dihydrotanshinlactone | 38.68 | 0.32 | DS |
| DS34 | dihydrotanshinoneⅠ | 45.04 | 0.36 | DS |
| DS35 | epidanshenspiroketallactone | 68.27 | 0.31 | DS |
| DS36 | C09092 | 36.07 | 0.25 | DS |
| DS37 | isocryptotanshi-none | 54.98 | 0.39 | DS |
| DS38 | Isotanshinone II | 49.92 | 0.4 | DS |
| DS39 | manool | 45.04 | 0.2 | DS |
| DS40 | miltionone Ⅰ | 49.68 | 0.32 | DS |
| DS41 | miltionone Ⅱ | 71.03 | 0.44 | DS |
| DS42 | miltipolone | 36.56 | 0.37 | DS |
| DS43 | Miltirone | 38.76 | 0.25 | DS |
| DS44 | neocryptotanshinone ii | 39.46 | 0.23 | DS |
| DS45 | neocryptotanshinone | 52.49 | 0.32 | DS |
| DS46 | 1-methyl-8,9-dihydro-7H-naphtho[5,6-g]benzofuran-6,10,11-trione | 34.72 | 0.37 | DS |
| DS47 | prolithospermic acid | 64.37 | 0.31 | DS |
| DS48 | (2R)-3-(3,4-dihydroxyphenyl)-2-[(Z)-3-(3,4-dihydroxyphenyl)acryloyl]oxy-propionic acid | 109.38 | 0.35 | DS |
| DS49 | salvianolic acid g | 45.56 | 0.61 | DS |
| DS50 | salvianolic acid j | 43.38 | 0.72 | DS |
| DS51 | salvilenone Ⅰ | 32.43 | 0.23 | DS |
| DS52 | salviolone | 31.72 | 0.24 | DS |
| DS53 | (6S)-6-hydroxy-1-methyl-6-methylol-8,9-dihydro-7H-naphtho[8,7-g]benzofuran-10,11-quinone | 75.39 | 0.46 | DS |
| DS54 | Tanshindiol B | 42.67 | 0.45 | DS |
| DS55 | Przewaquinone E | 42.85 | 0.45 | DS |
| DS56 | tanshinone iia | 49.89 | 0.4 | DS |
| DS57 | (6S)-6-(hydroxymethyl)-1,6-dimethyl-8,9-dihydro-7H-naphtho[8,7-g]benzofuran-10,11-dione | 65.26 | 0.45 | DS |
| DS58 | tanshinone Ⅵ | 45.64 | 0.3 | DS |
| EZ1 | bisdemethoxycurcumin | 77.38 | 0.26 | EZ |
| GL1 | Diosmetin | 31.14 | 0.27 | GL |
| GL2 | Spinasterol | 42.98 | 0.76 | GL |
| GL3 | Hydroxygenkwanin | 36.47 | 0.27 | GL |
| GL4 | Schottenol | 37.42 | 0.75 | GL |
| GL5 | 10α-cucurbita-5,24-diene-3β-ol | 44.02 | 0.74 | GL |
| GL6 | 5-dehydrokarounidiol | 30.23 | 0.77 | GL |
| GL7 | 7-oxo-dihydrokaro-unidiol | 36.85 | 0.75 | GL |
| GL8 | Linolenic acid ethyl ester | 46.1 | 0.2 | GL |
| GL9 | vitamin-e | 32.29 | 0.7 | GL |
| HJS1 | 3'-methyleriodictyol | 51.61 | 0.27 | HJS |
| HJS2 | Rhamnazin | 47.14 | 0.34 | HJS |
| HQ1 | (3S,8S,9S,10R,13R,14S,17R)-10,13-dimethyl-17-[(2R,5S)-5-propan-2-yloctan-2-yl]-2,3,4,7,8,9,11,12,14,15,16,17-dodecahydro-1H-cyclopenta[a]phenanthren-3-ol | 36.23 | 0.78 | HQ |
| HQ2 | Jaranol | 50.83 | 0.29 | HQ |
| HQ3 | 3,9-di-O-methylnissolin | 53.74 | 0.48 | HQ |
| HQ4 | 7-O-methylisomucronulatol | 36.74 | 0.92 | HQ |
| HQ5 | 9,10-dimethoxypterocarpan-3-O-β-D-glucoside | 64.26 | 0.42 | HQ |
| HQ6 | (6aR,11aR)-9,10-dimethoxy-6a,11a-dihydro-6H-benzofurano[3,2-c]chromen-3-ol | 31.1 | 0.67 | HQ |
| HQ7 | Bifendate | 69.67 | 0.21 | HQ |
| HQ8 | formononetin | 109.99 | 0.3 | HQ |
| HQ9 | Calycosin | 47.75 | 0.24 | HQ |
| HQ10 | FA | 68.96 | 0.71 | HQ |
| HQ11 | isomucronulatol-7,2'-di-O-glucosiole | 49.28 | 0.62 | HQ |
| HQ12 | 1,7-Dihydroxy-3,9-dimethoxy pterocarpene | 39.05 | 0.48 | HQ |
| JQC1 | acacetin | 34.97 | 0.24 | JQC |
| JQC2 | Linarin | 39.84 | 0.71 | JQC |
| JQC3 | Hesperetin | 70.31 | 0.27 | JQC |
| JQC4 | daucostero_qt | 36.91 | 0.75 | JQC |
| JQC5 | ent-Epicatechin | 48.96 | 0.24 | JQC |
| JQC6 | rhamnocitrin-3,4'-diglucOside | 32.52 | 0.64 | JQC |
| KWYXZ1 | astragalin | 14.03 | 0.74 | KWYXZ |
| KWYXZ2 | rutin | 3.2 | 0.68 | KWYXZ |
| KWYXZ3 | quercitrin | 4.04 | 0.74 | KWYXZ |
| KWYXZ4 | ellagic acid | 43.06 | 0.43 | KWYXZ |
| KWYXZ5 | Gallic acid | 31.69 | 0.04 | KWYXZ |
| KWYXZ6 | gallocatechin | 2.26 | 0.27 | KWYXZ |
| KWYXZ7 | corilagin | 3.01 | 0.44 | KWYXZ |
| KWYXZ8 | lupeol | 12.12 | 0.78 | KWYXZ |
| KWYXZ9 | ursolic acid | 16.77 | 0.75 | KWYXZ |
| KWYXZ10 | linalool | 49.37 | 0.04 | KWYXZ |
| KWYXZ11 | phytol | 33.82 | 0.13 | KWYXZ |
| KWYXZ12 | palmitic acid | 19.3 | 0.1 | KWYXZ |
| KWYXZ13 | 4-hydroxybenzaldehyde | 29.98 | 0.02 | KWYXZ |
| KWYXZ14 | apigenin | 23.06 | 0.21 | KWYXZ |
| KWYXZ15 | Hinokinin | 56.5 | 0.64 | KWYXZ |
| PFZ1 | 11,14-eicosadienoic acid | 39.99 | 0.2 | PFZ |
| PFZ2 | Delphin_qt | 57.76 | 0.28 | PFZ |
| PFZ3 | Deltoin | 46.69 | 0.37 | PFZ |
| PFZ4 | Deoxyandrographolide | 56.3 | 0.31 | PFZ |
| PFZ5 | Karanjin | 69.56 | 0.34 | PFZ |
| PFZ6 | (R)-Norcoclaurine | 82.54 | 0.21 | PFZ |
| SDH1 | Catalpol | 5.07 | 0.44 | SDH |
| SDH2 | Gamma-Aminobutyric Acid | 24.09 | 0.01 | SDH |
| SDH3 | [Acteoside](http://www.megabionet.org/tcmid/ingredient/23064/) | 2.94 | 0.62 | SDH |
| SDH4 | Rehmaglutin D | 57.09 | 0.1 | SDH |
| SDH5 | gentistic acid | 29.26 | 0.02 | SDH |
| SDH6 | versulin | 23.06 | 0.21 | SDH |
| SDH7 | Rehmaglutin A | 29.7 | 0.1 | SDH |
| SDH8 | Verbascoside | 2.94 | 0.62 | SDH |
| SQ1 | DFV | 32.76 | 0.18 | SQ |
| SQ2 | Diop | 43.59 | 0.39 | SQ |
| SQ3 | ginsenoside rh2 | 36.32 | 0.56 | SQ |
| SQ4 | Stigmasterol | 43.83 | 0.76 | SQ |
