# Supplementary material for: Network Pharmacology Approach to Explore the Potential Mechanisms of Jieduan-Niwan Formula Treating Acute-on-Chronic Liver Failure
Source: Evid Based Complement Alternat Med. 2020 Dec 30;2020:1041307. doi: 10.1155/2020/1041307 (PMC7787753; doi:10.1155/2020/1041307)
Supplement: Supplementary Materials — Supplementary Material 1: Table S1: information of potentially bioactive compounds of Jieduan-Niwan Formula. Supplementary Material 2: Table S2: the details of targets from compounds in JDNW Formula. Supplementary Material 3: Table S3: 1471 known ACLF-related targets. Supplementary Material 4: Table S4: 168 potential targets shared in JDNW Formula and ACLF. Supplementary Material 5: Table S5: information of potentially bioactive compounds of 168 common targets. Supplementary Material 6: Table S6: GO cellular component enrichment analysis of key targets of JDNW Formula in the treatment of ACLF. Supplementary Material 7: Table S7: KEGG pathway enrichment analysis of key targets of JDNW Formula in the treatment of ACLF. [file 1041307.f1.zip › 1041307.f1/Table S2.1041307.v2 (1).docx]

| **The details of targets from compounds in JDNW Formula** | | | | | |
| --- | --- | --- | --- | --- | --- |
| **ID** | **Molecule Name** | | **Target Protein Name** | **Gene Symbol** | **Uniprot ID** |
| DS1 | | 1,2,5,6-tetrahydrotanshinone | Prostaglandin G/H synthase 1 | PTGS1 | P23219 |
| DS1 | | 1,2,5,6-tetrahydrotanshinone | Muscarinic acetylcholine receptor M3 | CHRM3 | P20309 |
| DS1 | | 1,2,5,6-tetrahydrotanshinone | Muscarinic acetylcholine receptor M1 | CHRM1 | P11229 |
| DS1 | | 1,2,5,6-tetrahydrotanshinone | Sodium channel protein type 5 subunit alpha | SCN5A | Q14524 |
| DS1 | | 1,2,5,6-tetrahydrotanshinone | Muscarinic acetylcholine receptor M5 | CHRM5 | P08912 |
| DS1 | | 1,2,5,6-tetrahydrotanshinone | Prostaglandin G/H synthase 2 | PTGS2 | P35354 |
| DS1 | | 1,2,5,6-tetrahydrotanshinone | 5-hydroxytryptamine receptor 3A | HTR3A | P46098 |
| DS1 | | 1,2,5,6-tetrahydrotanshinone | Carbonic anhydrase II | CA2 | P00918 |
| DS1 | | 1,2,5,6-tetrahydrotanshinone | Muscarinic acetylcholine receptor M4 | CHRM4 | P08173 |
| DS1 | | 1,2,5,6-tetrahydrotanshinone | Retinoic acid receptor RXR-alpha | RXRA | P19793 |
| DS1 | | 1,2,5,6-tetrahydrotanshinone | Delta-type opioid receptor | OPRD1 | P41143 |
| DS1 | | 1,2,5,6-tetrahydrotanshinone | CGMP-inhibited 3',5'-cyclic phosphodiesterase A | PDE3A | Q14432 |
| DS1 | | 1,2,5,6-tetrahydrotanshinone | Alpha-1A adrenergic receptor | ADRA1A | P35348 |
| DS1 | | 1,2,5,6-tetrahydrotanshinone | Muscarinic acetylcholine receptor M2 | CHRM2 | P08172 |
| DS1 | | 1,2,5,6-tetrahydrotanshinone | Alpha-1B adrenergic receptor | ADRA1B | P35368 |
| DS1 | | 1,2,5,6-tetrahydrotanshinone | Sodium-dependent dopamine transporter | SLC6A3 | Q01959 |
| DS1 | | 1,2,5,6-tetrahydrotanshinone | Beta-2 adrenergic receptor | ADRB2 | P07550 |
| DS1 | | 1,2,5,6-tetrahydrotanshinone | Alpha-1D adrenergic receptor | ADRA1D | P25100 |
| DS1 | | 1,2,5,6-tetrahydrotanshinone | DNA topoisomerase II | TOP2A | P11388 |
| DS1 | | 1,2,5,6-tetrahydrotanshinone | Mu-type opioid receptor | OPRM1 | P35372 |
| DS1 | | 1,2,5,6-tetrahydrotanshinone | Gamma-aminobutyric acid receptor subunit alpha-1 | GABRA1 | P14867 |
| DS1 | | 1,2,5,6-tetrahydrotanshinone | Heat shock protein HSP 90 | HSP90AA1 | P07900 |
| DS1 | | 1,2,5,6-tetrahydrotanshinone | Neuronal acetylcholine receptor protein, alpha-7 chain | CHRNA7 | P36544 |
| DS1 | | 1,2,5,6-tetrahydrotanshinone | Nuclear receptor coactivator 2 | NCOA2 | Q15596 |
| DS1 | | 1,2,5,6-tetrahydrotanshinone | Nuclear receptor coactivator 1 | NCOA1 | Q15788 |
| DS1 | | 1,2,5,6-tetrahydrotanshinone | Dopamine D1 receptor | DRD1 | P21728 |
| DS1 | | 1,2,5,6-tetrahydrotanshinone | 5-hydroxytryptamine 2A receptor | HTR2A | P28223 |
| DS1 | | 1,2,5,6-tetrahydrotanshinone | Sodium-dependent serotonin transporter | SLC6A4 | P31645 |
| DS1 | | 1,2,5,6-tetrahydrotanshinone | Ig gamma-1 chain C region | IGHG1 | P01857 |
| DS2 | | Poriferasterol | Progesterone receptor | PGR | P06401 |
| DS2 | | Poriferasterol | Mineralocorticoid receptor | NR3C2 | P08235 |
| DS3 | | poriferast-5-en-3beta-ol | Progesterone receptor | PGR | P06401 |
| DS3 | | poriferast-5-en-3beta-ol | Nuclear receptor coactivator 2 | NCOA2 | Q15596 |
| DS4 | | isoimperatorin | Prostaglandin G/H synthase 2 | PTGS2 | P35354 |
| DS5 | | sugiol | Muscarinic acetylcholine receptor M3 | CHRM3 | P20309 |
| DS5 | | sugiol | Muscarinic acetylcholine receptor M1 | CHRM1 | P11229 |
| DS5 | | sugiol | Sodium channel protein type 5 subunit alpha | SCN5A | Q14524 |
| DS5 | | sugiol | Muscarinic acetylcholine receptor M5 | CHRM5 | P08912 |
| DS5 | | sugiol | Prostaglandin G/H synthase 2 | PTGS2 | P35354 |
| DS5 | | sugiol | Carbonic anhydrase II | CA2 | P00918 |
| DS5 | | sugiol | Muscarinic acetylcholine receptor M4 | CHRM4 | P08173 |
| DS5 | | sugiol | Delta-type opioid receptor | OPRD1 | P41143 |
| DS5 | | sugiol | Acetylcholinesterase | ACHE | P22303 |
| DS5 | | sugiol | Alpha-1A adrenergic receptor | ADRA1A | P35348 |
| DS5 | | sugiol | Muscarinic acetylcholine receptor M2 | CHRM2 | P08172 |
| DS5 | | sugiol | Alpha-1B adrenergic receptor | ADRA1B | P35368 |
| DS5 | | sugiol | Beta-2 adrenergic receptor | ADRB2 | P07550 |
| DS5 | | sugiol | Alpha-1D adrenergic receptor | ADRA1D | P25100 |
| DS5 | | sugiol | D(2) dopamine receptor | DRD2 | P14416 |
| DS5 | | sugiol | Mu-type opioid receptor | OPRM1 | P35372 |
| DS5 | | sugiol | Neuronal acetylcholine receptor protein, alpha-7 chain | CHRNA7 | P36544 |
| DS6 | | Dehydrotanshinone II A | Dopamine D1 receptor | DRD1 | P21728 |
| DS6 | | Dehydrotanshinone II A | Muscarinic acetylcholine receptor M3 | CHRM3 | P20309 |
| DS6 | | Dehydrotanshinone II A | Thrombin | F2 | P00734 |
| DS6 | | Dehydrotanshinone II A | Muscarinic acetylcholine receptor M1 | CHRM1 | P11229 |
| DS6 | | Dehydrotanshinone II A | Estrogen receptor | ESR1 | P03372 |
| DS6 | | Dehydrotanshinone II A | Androgen receptor | AR | P10275 |
| DS6 | | Dehydrotanshinone II A | Sodium channel protein type 5 subunit alpha | SCN5A | Q14524 |
| DS6 | | Dehydrotanshinone II A | Peroxisome proliferator activated receptor gamma | PPARG | P37231 |
| DS6 | | Dehydrotanshinone II A | Muscarinic acetylcholine receptor M5 | CHRM5 | P08912 |
| DS6 | | Dehydrotanshinone II A | Prostaglandin G/H synthase 2 | PTGS2 | P35354 |
| DS6 | | Dehydrotanshinone II A | Muscarinic acetylcholine receptor M4 | CHRM4 | P08173 |
| DS6 | | Dehydrotanshinone II A | Delta-type opioid receptor | OPRD1 | P41143 |
| DS6 | | Dehydrotanshinone II A | Acetylcholinesterase | ACHE | P22303 |
| DS6 | | Dehydrotanshinone II A | 5-hydroxytryptamine 2A receptor | HTR2A | P28223 |
| DS6 | | Dehydrotanshinone II A | Alpha-1A adrenergic receptor | ADRA1A | P35348 |
| DS6 | | Dehydrotanshinone II A | Beta-2 adrenergic receptor | ADRB2 | P07550 |
| DS6 | | Dehydrotanshinone II A | Mu-type opioid receptor | OPRM1 | P35372 |
| DS6 | | Dehydrotanshinone II A | Gamma-aminobutyric acid receptor subunit alpha-1 | GABRA1 | P14867 |
| DS6 | | Dehydrotanshinone II A | Dipeptidyl peptidase IV | DPP4 | P27487 |
| DS6 | | Dehydrotanshinone II A | Neuronal acetylcholine receptor protein, alpha-7 chain | CHRNA7 | P36544 |
| DS6 | | Dehydrotanshinone II A | Nuclear receptor coactivator 1 | NCOA1 | Q15788 |
| DS7 | | Baicalin | Coagulation factor Xa | F10 | P00742 |
| DS7 | | Baicalin | mRNA of Protein-tyrosine phosphatase, non-receptor type 1 | PTPN1 | P18031 |
| DS8 | | digallate | Prostaglandin G/H synthase 2 | PTGS2 | P35354 |
| DS8 | | digallate | Heat shock protein HSP 90 | HSP90AA1 | P07900 |
| DS8 | | digallate | Aldose reductase | AKR1B1 | P15121 |
| B1 | | luteolin | Prostaglandin G/H synthase 1 | PTGS1 | P23219 |
| B1 | | luteolin | Androgen receptor | AR | P10275 |
| B1 | | luteolin | Prostaglandin G/H synthase 2 | PTGS2 | P35354 |
| B1 | | luteolin | Heat shock protein HSP 90 | HSP90AA1 | P07900 |
| B1 | | luteolin | Trypsin-1 | PRSS1 | P07477 |
| B1 | | luteolin | Nuclear receptor coactivator 2 | NCOA2 | Q15596 |
| B1 | | luteolin | mRNA of PKA Catalytic Subunit C-alpha | PRKACA | P17612 |
| B1 | | luteolin | Dipeptidyl peptidase IV | DPP4 | P27487 |
| B1 | | luteolin | Phosphatidylinositol-4,5-bisphosphate 3-kinase catalytic subunit, gamma isoform | PIK3CG | P48736 |
| B1 | | luteolin | Transcription factor p65 | RELA | Q04206 |
| B1 | | luteolin | Epidermal growth factor receptor | EGFR | P00533 |
| B1 | | luteolin | RAC-alpha serine/threonine-protein kinase | AKT1 | P31749 |
| B1 | | luteolin | Vascular endothelial growth factor A | VEGFA | P15692 |
| B1 | | luteolin | G1/S-specific cyclin-D1 | CCND1 | P24385 |
| B1 | | luteolin | Bcl-2-like protein 1 | BCL2L1 | Q07817 |
| B1 | | luteolin | Cyclin-dependent kinase inhibitor 1 | CDKN1A | P38936 |
| B1 | | luteolin | Caspase-9 | CASP9 | P55211 |
| B1 | | luteolin | 72 kDa type IV collagenase | MMP2 | P08253 |
| B1 | | luteolin | Matrix metalloproteinase-9 | MMP9 | P14780 |
| B1 | | luteolin | Mitogen-activated protein kinase 1 | MAPK1 | P28482 |
| B1 | | luteolin | Interleukin-10 | IL10 | P22301 |
| B1 | | luteolin | Retinoblastoma-associated protein | RB1 | P06400 |
| B1 | | luteolin | Cell division protein kinase 4 | CDK4 | P11802 |
| B1 | | luteolin | Tumor necrosis factor | TNF | P01375 |
| B1 | | luteolin | Transcription factor AP-1 | JUN | P05412 |
| B1 | | luteolin | Interleukin-6 | IL6 | P05231 |
| B1 | | luteolin | Caspase-3 | CASP3 | P42574 |
| B1 | | luteolin | Cellular tumor antigen p53 | TP53 | P04637 |
| B1 | | luteolin | NF-kappa-B inhibitor alpha | NFKBIA | P25963 |
| B1 | | luteolin | Xanthine dehydrogenase/oxidase | XDH | P47989 |
| B1 | | luteolin | DNA topoisomerase 1 | TOP1 | P11387 |
| B1 | | luteolin | E3 ubiquitin-protein ligase Mdm2 | MDM2 | Q00987 |
| B1 | | luteolin | Amyloid beta A4 protein | APP | P05067 |
| B1 | | luteolin | Interstitial collagenase | MMP1 | P03956 |
| B1 | | luteolin | Proliferating cell nuclear antigen | PCNA | P12004 |
| B1 | | luteolin | Receptor tyrosine-protein kinase erbB-2 | ERBB2 | P04626 |
| B1 | | luteolin | Peroxisome proliferator-activated receptor gamma | PPARG | P37231 |
| B1 | | luteolin | Heme oxygenase 1 | HMOX1 | P09601 |
| B1 | | luteolin | Caspase-7 | CASP7 | P55210 |
| B1 | | luteolin | Intercellular adhesion molecule 1 | ICAM1 | P05362 |
| B1 | | luteolin | Induced myeloid leukemia cell differentiation protein Mcl-1 | MCL1 | Q07820 |
| B1 | | luteolin | Baculoviral IAP repeat-containing protein 5 | BIRC5 | O15392 |
| B1 | | luteolin | Interleukin-2 | IL2 | P60568 |
| B1 | | luteolin | G2/mitotic-specific cyclin-B1 | CCNB1 | P14635 |
| B1 | | luteolin | Tyrosinase | TYR | P14679 |
| B1 | | luteolin | Interferon gamma | IFNG | P01579 |
| B1 | | luteolin | Interleukin-4 | IL4 | P05112 |
| B1 | | luteolin | DNA topoisomerase 2-alpha | TOP2A | P11388 |
| B1 | | luteolin | Glutathione S-transferase P | GSTP1 | P09211 |
| B1 | | luteolin | Baculoviral IAP repeat-containing protein 4 | XIAP | P98170 |
| B1 | | luteolin | Solute carrier family 2, facilitated glucose transporter member 4 | SLC2A4 | P14672 |
| B1 | | luteolin | Insulin receptor | INSR | P06213 |
| B1 | | luteolin | CD40 ligand | CD40LG | P29965 |
| B1 | | luteolin | Prostaglandin E synthase | PTGES | O14684 |
| B1 | | luteolin | Kinetochore protein Nuf2 | NUF2 | Q9BZD4 |
| B1 | | luteolin | Adenylate cyclase type 2 | ADCY2 | Q08462 |
| B1 | | luteolin | Hepatocyte growth factor receptor | MET | P08581 |
| DS9 | | 5,6-dihydroxy-7-isopropyl-1,1-dimethyl-2,3-dihydrophenanthren-4-one | Prostaglandin G/H synthase 1 | PTGS1 | P23219 |
| DS9 | | 5,6-dihydroxy-7-isopropyl-1,1-dimethyl-2,3-dihydrophenanthren-4-one | Muscarinic acetylcholine receptor M3 | CHRM3 | P20309 |
| DS9 | | 5,6-dihydroxy-7-isopropyl-1,1-dimethyl-2,3-dihydrophenanthren-4-one | Muscarinic acetylcholine receptor M1 | CHRM1 | P11229 |
| DS9 | | 5,6-dihydroxy-7-isopropyl-1,1-dimethyl-2,3-dihydrophenanthren-4-one | Sodium channel protein type 5 subunit alpha | SCN5A | Q14524 |
| DS9 | | 5,6-dihydroxy-7-isopropyl-1,1-dimethyl-2,3-dihydrophenanthren-4-one | Prostaglandin G/H synthase 2 | PTGS2 | P35354 |
| DS9 | | 5,6-dihydroxy-7-isopropyl-1,1-dimethyl-2,3-dihydrophenanthren-4-one | Carbonic anhydrase II | CA2 | P00918 |
| DS9 | | 5,6-dihydroxy-7-isopropyl-1,1-dimethyl-2,3-dihydrophenanthren-4-one | Retinoic acid receptor RXR-alpha | RXRA | P19793 |
| DS9 | | 5,6-dihydroxy-7-isopropyl-1,1-dimethyl-2,3-dihydrophenanthren-4-one | Acetylcholinesterase | ACHE | P22303 |
| DS9 | | 5,6-dihydroxy-7-isopropyl-1,1-dimethyl-2,3-dihydrophenanthren-4-one | Alpha-1A adrenergic receptor | ADRA1A | P35348 |
| DS9 | | 5,6-dihydroxy-7-isopropyl-1,1-dimethyl-2,3-dihydrophenanthren-4-one | Alpha-1B adrenergic receptor | ADRA1B | P35368 |
| DS9 | | 5,6-dihydroxy-7-isopropyl-1,1-dimethyl-2,3-dihydrophenanthren-4-one | Beta-2 adrenergic receptor | ADRB2 | P07550 |
| DS9 | | 5,6-dihydroxy-7-isopropyl-1,1-dimethyl-2,3-dihydrophenanthren-4-one | DNA topoisomerase II | TOP2A | P11388 |
| DS9 | | 5,6-dihydroxy-7-isopropyl-1,1-dimethyl-2,3-dihydrophenanthren-4-one | Mu-type opioid receptor | OPRM1 | P35372 |
| DS9 | | 5,6-dihydroxy-7-isopropyl-1,1-dimethyl-2,3-dihydrophenanthren-4-one | Ig gamma-1 chain C region | IGHG1 | P01857 |
| DS9 | | 5,6-dihydroxy-7-isopropyl-1,1-dimethyl-2,3-dihydrophenanthren-4-one | Nuclear receptor coactivator 2 | NCOA2 | Q15596 |
| DS9 | | 5,6-dihydroxy-7-isopropyl-1,1-dimethyl-2,3-dihydrophenanthren-4-one | Nuclear receptor coactivator 1 | NCOA1 | Q15788 |
| DS9 | | 5,6-dihydroxy-7-isopropyl-1,1-dimethyl-2,3-dihydrophenanthren-4-one | Calmodulin | CALM1 | P0DP23 |
| DS10 | | 2-isopropyl-8-methylphenanthrene-3,4-dione | Prostaglandin G/H synthase 1 | PTGS1 | P23219 |
| DS10 | | 2-isopropyl-8-methylphenanthrene-3,4-dione | Dopamine D1 receptor | DRD1 | P21728 |
| DS10 | | 2-isopropyl-8-methylphenanthrene-3,4-dione | Muscarinic acetylcholine receptor M3 | CHRM3 | P20309 |
| DS10 | | 2-isopropyl-8-methylphenanthrene-3,4-dione | Muscarinic acetylcholine receptor M1 | CHRM1 | P11229 |
| DS10 | | 2-isopropyl-8-methylphenanthrene-3,4-dione | Estrogen receptor | ESR1 | P03372 |
| DS10 | | 2-isopropyl-8-methylphenanthrene-3,4-dione | Androgen receptor | AR | P10275 |
| DS10 | | 2-isopropyl-8-methylphenanthrene-3,4-dione | Sodium channel protein type 5 subunit alpha | SCN5A | Q14524 |
| DS10 | | 2-isopropyl-8-methylphenanthrene-3,4-dione | Peroxisome proliferator activated receptor gamma | PPARG | P37231 |
| DS10 | | 2-isopropyl-8-methylphenanthrene-3,4-dione | Muscarinic acetylcholine receptor M5 | CHRM5 | P08912 |
| DS10 | | 2-isopropyl-8-methylphenanthrene-3,4-dione | Prostaglandin G/H synthase 2 | PTGS2 | P35354 |
| DS10 | | 2-isopropyl-8-methylphenanthrene-3,4-dione | Nitric-oxide synthase, endothelial | NOS3 | P29474 |
| DS10 | | 2-isopropyl-8-methylphenanthrene-3,4-dione | 5-hydroxytryptamine receptor 3A | HTR3A | P46098 |
| DS10 | | 2-isopropyl-8-methylphenanthrene-3,4-dione | Muscarinic acetylcholine receptor M4 | CHRM4 | P08173 |
| DS10 | | 2-isopropyl-8-methylphenanthrene-3,4-dione | Retinoic acid receptor RXR-alpha | RXRA | P19793 |
| DS10 | | 2-isopropyl-8-methylphenanthrene-3,4-dione | 5-hydroxytryptamine 2A receptor | HTR2A | P28223 |
| DS10 | | 2-isopropyl-8-methylphenanthrene-3,4-dione | Alpha-1A adrenergic receptor | ADRA1A | P35348 |
| DS10 | | 2-isopropyl-8-methylphenanthrene-3,4-dione | Muscarinic acetylcholine receptor M2 | CHRM2 | P08172 |
| DS10 | | 2-isopropyl-8-methylphenanthrene-3,4-dione | Alpha-1B adrenergic receptor | ADRA1B | P35368 |
| DS10 | | 2-isopropyl-8-methylphenanthrene-3,4-dione | Sodium-dependent dopamine transporter | SLC6A3 | Q01959 |
| DS10 | | 2-isopropyl-8-methylphenanthrene-3,4-dione | Beta-2 adrenergic receptor | ADRB2 | P07550 |
| DS10 | | 2-isopropyl-8-methylphenanthrene-3,4-dione | Alpha-1D adrenergic receptor | ADRA1D | P25100 |
| DS10 | | 2-isopropyl-8-methylphenanthrene-3,4-dione | Sodium-dependent serotonin transporter | SLC6A4 | P31645 |
| DS10 | | 2-isopropyl-8-methylphenanthrene-3,4-dione | Mu-type opioid receptor | OPRM1 | P35372 |
| DS10 | | 2-isopropyl-8-methylphenanthrene-3,4-dione | Gamma-aminobutyric acid receptor subunit alpha-1 | GABRA1 | P14867 |
| DS10 | | 2-isopropyl-8-methylphenanthrene-3,4-dione | Cell division protein kinase 2 | CDK2 | P24941 |
| DS10 | | 2-isopropyl-8-methylphenanthrene-3,4-dione | Phosphatidylinositol-4,5-bisphosphate 3-kinase catalytic subunit, gamma isoform | PIK3CG | P48736 |
| DS10 | | 2-isopropyl-8-methylphenanthrene-3,4-dione | Neuronal acetylcholine receptor protein, alpha-7 chain | CHRNA7 | P36544 |
| DS10 | | 2-isopropyl-8-methylphenanthrene-3,4-dione | mRNA of PKA Catalytic Subunit C-alpha | PRKACA | P17612 |
| DS10 | | 2-isopropyl-8-methylphenanthrene-3,4-dione | Ig gamma-1 chain C region | IGHG1 | P01857 |
| DS10 | | 2-isopropyl-8-methylphenanthrene-3,4-dione | Proto-oncogene serine/threonine-protein kinase Pim-1 | PIM1 | P11309 |
| DS10 | | 2-isopropyl-8-methylphenanthrene-3,4-dione | Cyclin-A2 | CCNA2 | P20248 |
| DS10 | | 2-isopropyl-8-methylphenanthrene-3,4-dione | Nuclear receptor coactivator 2 | NCOA2 | Q15596 |
| DS10 | | 2-isopropyl-8-methylphenanthrene-3,4-dione | Calmodulin | CALM1 | P0DP23 |
| DS11 | | 3α-hydroxytanshinoneⅡa | Thrombin | F2 | P00734 |
| DS11 | | 3α-hydroxytanshinoneⅡa | Muscarinic acetylcholine receptor M1 | CHRM1 | P11229 |
| DS11 | | 3α-hydroxytanshinoneⅡa | Sodium channel protein type 5 subunit alpha | SCN5A | Q14524 |
| DS11 | | 3α-hydroxytanshinoneⅡa | Muscarinic acetylcholine receptor M5 | CHRM5 | P08912 |
| DS11 | | 3α-hydroxytanshinoneⅡa | Prostaglandin G/H synthase 2 | PTGS2 | P35354 |
| DS11 | | 3α-hydroxytanshinoneⅡa | Delta-type opioid receptor | OPRD1 | P41143 |
| DS11 | | 3α-hydroxytanshinoneⅡa | Acetylcholinesterase | ACHE | P22303 |
| DS11 | | 3α-hydroxytanshinoneⅡa | Beta-2 adrenergic receptor | ADRB2 | P07550 |
| DS11 | | 3α-hydroxytanshinoneⅡa | Mu-type opioid receptor | OPRM1 | P35372 |
| DS11 | | 3α-hydroxytanshinoneⅡa | Dipeptidyl peptidase IV | DPP4 | P27487 |
| DS11 | | 3α-hydroxytanshinoneⅡa | Neuronal acetylcholine receptor protein, alpha-7 chain | CHRNA7 | P36544 |
| DS11 | | 3α-hydroxytanshinoneⅡa | Trypsin-1 | PRSS1 | P07477 |
| DS11 | | 3α-hydroxytanshinoneⅡa | Nuclear receptor coactivator 1 | NCOA1 | Q15788 |
| DS12 | | (E)-3-[2-(3,4-dihydroxyphenyl)-7-hydroxy-benzofuran-4-yl]acrylic acid | Prostaglandin G/H synthase 2 | PTGS2 | P35354 |
| DS12 | | (E)-3-[2-(3,4-dihydroxyphenyl)-7-hydroxy-benzofuran-4-yl]acrylic acid | mRNA of Protein-tyrosine phosphatase, non-receptor type 1 | PTPN1 | P18031 |
| DS12 | | (E)-3-[2-(3,4-dihydroxyphenyl)-7-hydroxy-benzofuran-4-yl]acrylic acid | Heat shock protein HSP 90 | HSP90AA1 | P07900 |
| DS13 | | 4-methylenemiltirone | Prostaglandin G/H synthase 1 | PTGS1 | P23219 |
| DS13 | | 4-methylenemiltirone | Dopamine D1 receptor | DRD1 | P21728 |
| DS13 | | 4-methylenemiltirone | Muscarinic acetylcholine receptor M3 | CHRM3 | P20309 |
| DS13 | | 4-methylenemiltirone | Muscarinic acetylcholine receptor M1 | CHRM1 | P11229 |
| DS13 | | 4-methylenemiltirone | Estrogen receptor | ESR1 | P03372 |
| DS13 | | 4-methylenemiltirone | Androgen receptor | AR | P10275 |
| DS13 | | 4-methylenemiltirone | Sodium channel protein type 5 subunit alpha | SCN5A | Q14524 |
| DS13 | | 4-methylenemiltirone | Peroxisome proliferator activated receptor gamma | PPARG | P37231 |
| DS13 | | 4-methylenemiltirone | Muscarinic acetylcholine receptor M5 | CHRM5 | P08912 |
| DS13 | | 4-methylenemiltirone | Prostaglandin G/H synthase 2 | PTGS2 | P35354 |
| DS13 | | 4-methylenemiltirone | Nitric-oxide synthase, endothelial | NOS3 | P29474 |
| DS13 | | 4-methylenemiltirone | Alpha-2A adrenergic receptor | ADRA2A | P08913 |
| DS13 | | 4-methylenemiltirone | Carbonic anhydrase II | CA2 | P00918 |
| DS13 | | 4-methylenemiltirone | Alpha-2C adrenergic receptor | ADRA2C | P18825 |
| DS13 | | 4-methylenemiltirone | Muscarinic acetylcholine receptor M4 | CHRM4 | P08173 |
| DS13 | | 4-methylenemiltirone | Retinoic acid receptor RXR-alpha | RXRA | P19793 |
| DS13 | | 4-methylenemiltirone | Delta-type opioid receptor | OPRD1 | P41143 |
| DS13 | | 4-methylenemiltirone | 5-hydroxytryptamine 2A receptor | HTR2A | P28223 |
| DS13 | | 4-methylenemiltirone | Alpha-1A adrenergic receptor | ADRA1A | P35348 |
| DS13 | | 4-methylenemiltirone | Muscarinic acetylcholine receptor M2 | CHRM2 | P08172 |
| DS13 | | 4-methylenemiltirone | Alpha-1B adrenergic receptor | ADRA1B | P35368 |
| DS13 | | 4-methylenemiltirone | Sodium-dependent dopamine transporter | SLC6A3 | Q01959 |
| DS13 | | 4-methylenemiltirone | Beta-2 adrenergic receptor | ADRB2 | P07550 |
| DS13 | | 4-methylenemiltirone | Alpha-1D adrenergic receptor | ADRA1D | P25100 |
| DS13 | | 4-methylenemiltirone | DNA topoisomerase II | TOP2A | P11388 |
| DS13 | | 4-methylenemiltirone | Sodium-dependent serotonin transporter | SLC6A4 | P31645 |
| DS13 | | 4-methylenemiltirone | D(2) dopamine receptor | DRD2 | P14416 |
| DS13 | | 4-methylenemiltirone | Mu-type opioid receptor | OPRM1 | P35372 |
| DS13 | | 4-methylenemiltirone | Gamma-aminobutyric acid receptor subunit alpha-1 | GABRA1 | P14867 |
| DS13 | | 4-methylenemiltirone | Neuronal acetylcholine receptor protein, alpha-7 chain | CHRNA7 | P36544 |
| DS13 | | 4-methylenemiltirone | mRNA of PKA Catalytic Subunit C-alpha | PRKACA | P17612 |
| DS13 | | 4-methylenemiltirone | Nuclear receptor coactivator 2 | NCOA2 | Q15596 |
| DS13 | | 4-methylenemiltirone | Nuclear receptor coactivator 1 | NCOA1 | Q15788 |
| DS14 | | 2-(4-hydroxy-3-methoxyphenyl)-5-(3-hydroxypropyl)-7-methoxy-3-benzofurancarboxaldehyde | Nitric oxide synthase, inducible | NOS2 | P35228 |
| DS14 | | 2-(4-hydroxy-3-methoxyphenyl)-5-(3-hydroxypropyl)-7-methoxy-3-benzofurancarboxaldehyde | Thrombin | F2 | P00734 |
| DS14 | | 2-(4-hydroxy-3-methoxyphenyl)-5-(3-hydroxypropyl)-7-methoxy-3-benzofurancarboxaldehyde | Estrogen receptor | ESR1 | P03372 |
| DS14 | | 2-(4-hydroxy-3-methoxyphenyl)-5-(3-hydroxypropyl)-7-methoxy-3-benzofurancarboxaldehyde | Androgen receptor | AR | P10275 |
| DS14 | | 2-(4-hydroxy-3-methoxyphenyl)-5-(3-hydroxypropyl)-7-methoxy-3-benzofurancarboxaldehyde | Peroxisome proliferator activated receptor gamma | PPARG | P37231 |
| DS14 | | 2-(4-hydroxy-3-methoxyphenyl)-5-(3-hydroxypropyl)-7-methoxy-3-benzofurancarboxaldehyde | Estrogen receptor beta | ESR2 | Q92731 |
| DS14 | | 2-(4-hydroxy-3-methoxyphenyl)-5-(3-hydroxypropyl)-7-methoxy-3-benzofurancarboxaldehyde | Mitogen-activated protein kinase 14 | MAPK14 | Q16539 |
| DS14 | | 2-(4-hydroxy-3-methoxyphenyl)-5-(3-hydroxypropyl)-7-methoxy-3-benzofurancarboxaldehyde | Glycogen synthase kinase-3 beta | GSK3B | P49841 |
| DS14 | | 2-(4-hydroxy-3-methoxyphenyl)-5-(3-hydroxypropyl)-7-methoxy-3-benzofurancarboxaldehyde | Heat shock protein HSP 90 | HSP90AA1 | P07900 |
| DS14 | | 2-(4-hydroxy-3-methoxyphenyl)-5-(3-hydroxypropyl)-7-methoxy-3-benzofurancarboxaldehyde | Cell division protein kinase 2 | CDK2 | P24941 |
| DS14 | | 2-(4-hydroxy-3-methoxyphenyl)-5-(3-hydroxypropyl)-7-methoxy-3-benzofurancarboxaldehyde | Proto-oncogene serine/threonine-protein kinase Pim-1 | PIM1 | P11309 |
| DS14 | | 2-(4-hydroxy-3-methoxyphenyl)-5-(3-hydroxypropyl)-7-methoxy-3-benzofurancarboxaldehyde | Cyclin-A2 | CCNA2 | P20248 |
| DS15 | | formyltanshinone | Thrombin | F2 | P00734 |
| DS15 | | formyltanshinone | Androgen receptor | AR | P10275 |
| DS15 | | formyltanshinone | Prostaglandin G/H synthase 2 | PTGS2 | P35354 |
| DS15 | | formyltanshinone | Retinoic acid receptor RXR-alpha | RXRA | P19793 |
| DS15 | | formyltanshinone | Dipeptidyl peptidase IV | DPP4 | P27487 |
| DS15 | | formyltanshinone | Phosphatidylinositol-4,5-bisphosphate 3-kinase catalytic subunit, gamma isoform | PIK3CG | P48736 |
| DS15 | | formyltanshinone | mRNA of PKA Catalytic Subunit C-alpha | PRKACA | P17612 |
| DS15 | | formyltanshinone | Nuclear receptor coactivator 1 | NCOA1 | Q15788 |
| DS16 | | 3-beta-Hydroxymethyllenetanshiquinone | Dopamine D1 receptor | DRD1 | P21728 |
| DS16 | | 3-beta-Hydroxymethyllenetanshiquinone | Thrombin | F2 | P00734 |
| DS16 | | 3-beta-Hydroxymethyllenetanshiquinone | Muscarinic acetylcholine receptor M1 | CHRM1 | P11229 |
| DS16 | | 3-beta-Hydroxymethyllenetanshiquinone | Prostaglandin G/H synthase 2 | PTGS2 | P35354 |
| DS16 | | 3-beta-Hydroxymethyllenetanshiquinone | Carbonic anhydrase II | CA2 | P00918 |
| DS16 | | 3-beta-Hydroxymethyllenetanshiquinone | Retinoic acid receptor RXR-alpha | RXRA | P19793 |
| DS16 | | 3-beta-Hydroxymethyllenetanshiquinone | Delta-type opioid receptor | OPRD1 | P41143 |
| DS16 | | 3-beta-Hydroxymethyllenetanshiquinone | Acetylcholinesterase | ACHE | P22303 |
| DS16 | | 3-beta-Hydroxymethyllenetanshiquinone | Alpha-1A adrenergic receptor | ADRA1A | P35348 |
| DS16 | | 3-beta-Hydroxymethyllenetanshiquinone | Beta-2 adrenergic receptor | ADRB2 | P07550 |
| DS16 | | 3-beta-Hydroxymethyllenetanshiquinone | Mu-type opioid receptor | OPRM1 | P35372 |
| DS16 | | 3-beta-Hydroxymethyllenetanshiquinone | Dipeptidyl peptidase IV | DPP4 | P27487 |
| DS16 | | 3-beta-Hydroxymethyllenetanshiquinone | Heat shock protein HSP 90 | HSP90AA1 | P07900 |
| DS16 | | 3-beta-Hydroxymethyllenetanshiquinone | Neuronal acetylcholine receptor protein, alpha-7 chain | CHRNA7 | P36544 |
| DS16 | | 3-beta-Hydroxymethyllenetanshiquinone | Ig gamma-1 chain C region | IGHG1 | P01857 |
| DS16 | | 3-beta-Hydroxymethyllenetanshiquinone | Trypsin-1 | PRSS1 | P07477 |
| DS16 | | 3-beta-Hydroxymethyllenetanshiquinone | Nuclear receptor coactivator 1 | NCOA1 | Q15788 |
| DS17 | | Methylenetanshinquinone | Dopamine D1 receptor | DRD1 | P21728 |
| DS17 | | Methylenetanshinquinone | Muscarinic acetylcholine receptor M3 | CHRM3 | P20309 |
| DS17 | | Methylenetanshinquinone | Thrombin | F2 | P00734 |
| DS17 | | Methylenetanshinquinone | Muscarinic acetylcholine receptor M1 | CHRM1 | P11229 |
| DS17 | | Methylenetanshinquinone | Sodium channel protein type 5 subunit alpha | SCN5A | Q14524 |
| DS17 | | Methylenetanshinquinone | Muscarinic acetylcholine receptor M5 | CHRM5 | P08912 |
| DS17 | | Methylenetanshinquinone | Prostaglandin G/H synthase 2 | PTGS2 | P35354 |
| DS17 | | Methylenetanshinquinone | Carbonic anhydrase II | CA2 | P00918 |
| DS17 | | Methylenetanshinquinone | Retinoic acid receptor RXR-alpha | RXRA | P19793 |
| DS17 | | Methylenetanshinquinone | Delta-type opioid receptor | OPRD1 | P41143 |
| DS17 | | Methylenetanshinquinone | Acetylcholinesterase | ACHE | P22303 |
| DS17 | | Methylenetanshinquinone | 5-hydroxytryptamine 2A receptor | HTR2A | P28223 |
| DS17 | | Methylenetanshinquinone | Alpha-1A adrenergic receptor | ADRA1A | P35348 |
| DS17 | | Methylenetanshinquinone | Muscarinic acetylcholine receptor M2 | CHRM2 | P08172 |
| DS17 | | Methylenetanshinquinone | Beta-2 adrenergic receptor | ADRB2 | P07550 |
| DS17 | | Methylenetanshinquinone | Sodium-dependent serotonin transporter | SLC6A4 | P31645 |
| DS17 | | Methylenetanshinquinone | Mu-type opioid receptor | OPRM1 | P35372 |
| DS17 | | Methylenetanshinquinone | Gamma-aminobutyric acid receptor subunit alpha-1 | GABRA1 | P14867 |
| DS17 | | Methylenetanshinquinone | Dipeptidyl peptidase IV | DPP4 | P27487 |
| DS17 | | Methylenetanshinquinone | Heat shock protein HSP 90 | HSP90AA1 | P07900 |
| DS17 | | Methylenetanshinquinone | Neuronal acetylcholine receptor protein, alpha-7 chain | CHRNA7 | P36544 |
| DS17 | | Methylenetanshinquinone | Ig gamma-1 chain C region | IGHG1 | P01857 |
| DS17 | | Methylenetanshinquinone | Trypsin-1 | PRSS1 | P07477 |
| DS17 | | Methylenetanshinquinone | Nuclear receptor coactivator 1 | NCOA1 | Q15788 |
| DS18 | | przewalskin a | Mineralocorticoid receptor | NR3C2 | P08235 |
| DS18 | | przewalskin a | Glucocorticoid receptor | NR3C1 | P04150 |
| DS19 | | przewalskin b | Prostaglandin G/H synthase 2 | PTGS2 | P35354 |
| DS19 | | przewalskin b | Progesterone receptor | PGR | P06401 |
| DS19 | | przewalskin b | Mineralocorticoid receptor | NR3C2 | P08235 |
| DS19 | | przewalskin b | Glucocorticoid receptor | NR3C1 | P04150 |
| DS19 | | przewalskin b | Nuclear receptor coactivator 2 | NCOA2 | Q15596 |
| DS19 | | przewalskin b | Nuclear receptor coactivator 1 | NCOA1 | Q15788 |
| DS20 | | Przewaquinone B | Thrombin | F2 | P00734 |
| DS20 | | Przewaquinone B | Prostaglandin G/H synthase 2 | PTGS2 | P35354 |
| DS20 | | Przewaquinone B | Retinoic acid receptor RXR-alpha | RXRA | P19793 |
| DS20 | | Przewaquinone B | Dipeptidyl peptidase IV | DPP4 | P27487 |
| DS20 | | Przewaquinone B | Heat shock protein HSP 90 | HSP90AA1 | P07900 |
| DS20 | | Przewaquinone B | Phosphatidylinositol-4,5-bisphosphate 3-kinase catalytic subunit, gamma isoform | PIK3CG | P48736 |
| DS20 | | Przewaquinone B | Ig gamma-1 chain C region | IGHG1 | P01857 |
| DS20 | | Przewaquinone B | Trypsin-1 | PRSS1 | P07477 |
| DS20 | | Przewaquinone B | Nuclear receptor coactivator 1 | NCOA1 | Q15788 |
| DS21 | | przewaquinone c | Prostaglandin G/H synthase 1 | PTGS1 | P23219 |
| DS21 | | przewaquinone c | Dopamine D1 receptor | DRD1 | P21728 |
| DS21 | | przewaquinone c | Muscarinic acetylcholine receptor M3 | CHRM3 | P20309 |
| DS21 | | przewaquinone c | Thrombin | F2 | P00734 |
| DS21 | | przewaquinone c | Muscarinic acetylcholine receptor M1 | CHRM1 | P11229 |
| DS21 | | przewaquinone c | Sodium channel protein type 5 subunit alpha | SCN5A | Q14524 |
| DS21 | | przewaquinone c | Muscarinic acetylcholine receptor M5 | CHRM5 | P08912 |
| DS21 | | przewaquinone c | Prostaglandin G/H synthase 2 | PTGS2 | P35354 |
| DS21 | | przewaquinone c | Carbonic anhydrase II | CA2 | P00918 |
| DS21 | | przewaquinone c | Muscarinic acetylcholine receptor M4 | CHRM4 | P08173 |
| DS21 | | przewaquinone c | Delta-type opioid receptor | OPRD1 | P41143 |
| DS21 | | przewaquinone c | Acetylcholinesterase | ACHE | P22303 |
| DS21 | | przewaquinone c | Alpha-1A adrenergic receptor | ADRA1A | P35348 |
| DS21 | | przewaquinone c | Muscarinic acetylcholine receptor M2 | CHRM2 | P08172 |
| DS21 | | przewaquinone c | Beta-2 adrenergic receptor | ADRB2 | P07550 |
| DS21 | | przewaquinone c | Mu-type opioid receptor | OPRM1 | P35372 |
| DS21 | | przewaquinone c | Gamma-aminobutyric acid receptor subunit alpha-1 | GABRA1 | P14867 |
| DS21 | | przewaquinone c | Dipeptidyl peptidase IV | DPP4 | P27487 |
| DS21 | | przewaquinone c | Heat shock protein HSP 90 | HSP90AA1 | P07900 |
| DS21 | | przewaquinone c | Phosphatidylinositol-4,5-bisphosphate 3-kinase catalytic subunit, gamma isoform | PIK3CG | P48736 |
| DS21 | | przewaquinone c | Neuronal acetylcholine receptor protein, alpha-7 chain | CHRNA7 | P36544 |
| DS21 | | przewaquinone c | Nuclear receptor coactivator 1 | NCOA1 | Q15788 |
| DS22 | | (6S,7R)-6,7-dihydroxy-1,6-dimethyl-8,9-dihydro-7H-naphtho[8,7-g]benzofuran-10,11-dione | Thrombin | F2 | P00734 |
| DS22 | | (6S,7R)-6,7-dihydroxy-1,6-dimethyl-8,9-dihydro-7H-naphtho[8,7-g]benzofuran-10,11-dione | Prostaglandin G/H synthase 2 | PTGS2 | P35354 |
| DS22 | | (6S,7R)-6,7-dihydroxy-1,6-dimethyl-8,9-dihydro-7H-naphtho[8,7-g]benzofuran-10,11-dione | Carbonic anhydrase II | CA2 | P00918 |
| DS22 | | (6S,7R)-6,7-dihydroxy-1,6-dimethyl-8,9-dihydro-7H-naphtho[8,7-g]benzofuran-10,11-dione | Acetylcholinesterase | ACHE | P22303 |
| DS22 | | (6S,7R)-6,7-dihydroxy-1,6-dimethyl-8,9-dihydro-7H-naphtho[8,7-g]benzofuran-10,11-dione | Dipeptidyl peptidase IV | DPP4 | P27487 |
| DS22 | | (6S,7R)-6,7-dihydroxy-1,6-dimethyl-8,9-dihydro-7H-naphtho[8,7-g]benzofuran-10,11-dione | Heat shock protein HSP 90 | HSP90AA1 | P07900 |
| DS22 | | (6S,7R)-6,7-dihydroxy-1,6-dimethyl-8,9-dihydro-7H-naphtho[8,7-g]benzofuran-10,11-dione | Trypsin-1 | PRSS1 | P07477 |
| DS22 | | (6S,7R)-6,7-dihydroxy-1,6-dimethyl-8,9-dihydro-7H-naphtho[8,7-g]benzofuran-10,11-dione | Nuclear receptor coactivator 1 | NCOA1 | Q15788 |
| DS23 | | przewaquinone f | Thrombin | F2 | P00734 |
| DS23 | | przewaquinone f | Prostaglandin G/H synthase 2 | PTGS2 | P35354 |
| DS23 | | przewaquinone f | Dipeptidyl peptidase IV | DPP4 | P27487 |
| DS23 | | przewaquinone f | Trypsin-1 | PRSS1 | P07477 |
| DS23 | | przewaquinone f | Nuclear receptor coactivator 1 | NCOA1 | Q15788 |
| DS24 | | sclareol | Prostaglandin G/H synthase 2 | PTGS2 | P35354 |
| DS25 | | tanshinaldehyde | Dopamine D1 receptor | DRD1 | P21728 |
| DS25 | | tanshinaldehyde | Thrombin | F2 | P00734 |
| DS25 | | tanshinaldehyde | Muscarinic acetylcholine receptor M1 | CHRM1 | P11229 |
| DS25 | | tanshinaldehyde | Prostaglandin G/H synthase 2 | PTGS2 | P35354 |
| DS25 | | tanshinaldehyde | Delta-type opioid receptor | OPRD1 | P41143 |
| DS25 | | tanshinaldehyde | Acetylcholinesterase | ACHE | P22303 |
| DS25 | | tanshinaldehyde | 5-hydroxytryptamine 2A receptor | HTR2A | P28223 |
| DS25 | | tanshinaldehyde | Beta-2 adrenergic receptor | ADRB2 | P07550 |
| DS25 | | tanshinaldehyde | Mu-type opioid receptor | OPRM1 | P35372 |
| DS25 | | tanshinaldehyde | Dipeptidyl peptidase IV | DPP4 | P27487 |
| DS25 | | tanshinaldehyde | Neuronal acetylcholine receptor protein, alpha-7 chain | CHRNA7 | P36544 |
| DS25 | | tanshinaldehyde | Trypsin-1 | PRSS1 | P07477 |
| DS25 | | tanshinaldehyde | Nuclear receptor coactivator 1 | NCOA1 | Q15788 |
| DS26 | | Danshenol B | Prostaglandin G/H synthase 2 | PTGS2 | P35354 |
| DS26 | | Danshenol B | Carbonic anhydrase II | CA2 | P00918 |
| DS26 | | Danshenol B | Progesterone receptor | PGR | P06401 |
| DS26 | | Danshenol B | DNA topoisomerase II | TOP2A | P11388 |
| DS26 | | Danshenol B | Mu-type opioid receptor | OPRM1 | P35372 |
| DS26 | | Danshenol B | Glucocorticoid receptor | NR3C1 | P04150 |
| DS26 | | Danshenol B | Heat shock protein HSP 90 | HSP90AA1 | P07900 |
| DS26 | | Danshenol B | Nuclear receptor coactivator 1 | NCOA1 | Q15788 |
| DS27 | | Danshenol A | Prostaglandin G/H synthase 1 | PTGS1 | P23219 |
| DS27 | | Danshenol A | Potassium voltage-gated channel subfamily H member 2 | KCNH2 | Q12809 |
| DS27 | | Danshenol A | Sodium channel protein type 5 subunit alpha | SCN5A | Q14524 |
| DS27 | | Danshenol A | Coagulation factor Xa | F10 | P00742 |
| DS27 | | Danshenol A | Prostaglandin G/H synthase 2 | PTGS2 | P35354 |
| DS27 | | Danshenol A | Retinoic acid receptor RXR-alpha | RXRA | P19793 |
| DS27 | | Danshenol A | Phosphatidylinositol-4,5-bisphosphate 3-kinase catalytic subunit, gamma isoform | PIK3CG | P48736 |
| DS27 | | Danshenol A | Nuclear receptor coactivator 1 | NCOA1 | Q15788 |
| DS27 | | Danshenol A | Calcium-activated potassium channel subunit alpha 1 | KCNMA1 | Q12791 |
| DS28 | | Salvilenone | Prostaglandin G/H synthase 1 | PTGS1 | P23219 |
| DS28 | | Salvilenone | Estrogen receptor | ESR1 | P03372 |
| DS28 | | Salvilenone | Androgen receptor | AR | P10275 |
| DS28 | | Salvilenone | Muscarinic acetylcholine receptor M5 | CHRM5 | P08912 |
| DS28 | | Salvilenone | Prostaglandin G/H synthase 2 | PTGS2 | P35354 |
| DS28 | | Salvilenone | 5-hydroxytryptamine receptor 3A | HTR3A | P46098 |
| DS28 | | Salvilenone | Estrogen receptor beta | ESR2 | Q92731 |
| DS28 | | Salvilenone | Proto-oncogene serine/threonine-protein kinase Pim-1 | PIM1 | P11309 |
| DS29 | | cryptotanshinone | Prostaglandin G/H synthase 1 | PTGS1 | P23219 |
| DS29 | | cryptotanshinone | Dopamine D1 receptor | DRD1 | P21728 |
| DS29 | | cryptotanshinone | Muscarinic acetylcholine receptor M3 | CHRM3 | P20309 |
| DS29 | | cryptotanshinone | Muscarinic acetylcholine receptor M1 | CHRM1 | P11229 |
| DS29 | | cryptotanshinone | Sodium channel protein type 5 subunit alpha | SCN5A | Q14524 |
| DS29 | | cryptotanshinone | Muscarinic acetylcholine receptor M5 | CHRM5 | P08912 |
| DS29 | | cryptotanshinone | Prostaglandin G/H synthase 2 | PTGS2 | P35354 |
| DS29 | | cryptotanshinone | Carbonic anhydrase II | CA2 | P00918 |
| DS29 | | cryptotanshinone | Muscarinic acetylcholine receptor M4 | CHRM4 | P08173 |
| DS29 | | cryptotanshinone | Delta-type opioid receptor | OPRD1 | P41143 |
| DS29 | | cryptotanshinone | Alpha-1A adrenergic receptor | ADRA1A | P35348 |
| DS29 | | cryptotanshinone | Muscarinic acetylcholine receptor M2 | CHRM2 | P08172 |
| DS29 | | cryptotanshinone | Alpha-1B adrenergic receptor | ADRA1B | P35368 |
| DS29 | | cryptotanshinone | Beta-2 adrenergic receptor | ADRB2 | P07550 |
| DS29 | | cryptotanshinone | Alpha-1D adrenergic receptor | ADRA1D | P25100 |
| DS29 | | cryptotanshinone | DNA topoisomerase II | TOP2A | P11388 |
| DS29 | | cryptotanshinone | Mu-type opioid receptor | OPRM1 | P35372 |
| DS29 | | cryptotanshinone | Neuronal acetylcholine receptor protein, alpha-7 chain | CHRNA7 | P36544 |
| DS29 | | cryptotanshinone | Nuclear receptor coactivator 2 | NCOA2 | Q15596 |
| DS29 | | cryptotanshinone | Nuclear receptor coactivator 1 | NCOA1 | Q15788 |
| DS29 | | cryptotanshinone | Progesterone receptor | PGR | P06401 |
| DS29 | | cryptotanshinone | Gamma-aminobutyric acid receptor subunit alpha-1 | GABRA1 | P14867 |
| DS29 | | cryptotanshinone | Transcription factor p65 | RELA | Q04206 |
| DS29 | | cryptotanshinone | Signal transducer and activator of transcription 3 | STAT3 | P40763 |
| DS29 | | cryptotanshinone | G1/S-specific cyclin-D1 | CCND1 | P24385 |
| DS29 | | cryptotanshinone | Bcl-2-like protein 1 | BCL2L1 | Q07817 |
| DS29 | | cryptotanshinone | Tumor necrosis factor | TNF | P01375 |
| DS29 | | cryptotanshinone | Amyloid beta A4 protein | APP | P05067 |
| DS29 | | cryptotanshinone | Endothelin-1 | EDN1 | P05305 |
| DS29 | | cryptotanshinone | Baculoviral IAP repeat-containing protein 5 | BIRC5 | O15392 |
| DS30 | | dan-shexinkum d | Nitric oxide synthase, inducible | NOS2 | P35228 |
| DS30 | | dan-shexinkum d | Prostaglandin G/H synthase 1 | PTGS1 | P23219 |
| DS30 | | dan-shexinkum d | Thrombin | F2 | P00734 |
| DS30 | | dan-shexinkum d | Potassium voltage-gated channel subfamily H member 2 | KCNH2 | Q12809 |
| DS30 | | dan-shexinkum d | Muscarinic acetylcholine receptor M1 | CHRM1 | P11229 |
| DS30 | | dan-shexinkum d | Estrogen receptor | ESR1 | P03372 |
| DS30 | | dan-shexinkum d | Androgen receptor | AR | P10275 |
| DS30 | | dan-shexinkum d | Sodium channel protein type 5 subunit alpha | SCN5A | Q14524 |
| DS30 | | dan-shexinkum d | Peroxisome proliferator activated receptor gamma | PPARG | P37231 |
| DS30 | | dan-shexinkum d | Coagulation factor Xa | F10 | P00742 |
| DS30 | | dan-shexinkum d | Prostaglandin G/H synthase 2 | PTGS2 | P35354 |
| DS30 | | dan-shexinkum d | Carbonic anhydrase II | CA2 | P00918 |
| DS30 | | dan-shexinkum d | Retinoic acid receptor RXR-alpha | RXRA | P19793 |
| DS30 | | dan-shexinkum d | Acetylcholinesterase | ACHE | P22303 |
| DS30 | | dan-shexinkum d | Alpha-1B adrenergic receptor | ADRA1B | P35368 |
| DS30 | | dan-shexinkum d | Beta-2 adrenergic receptor | ADRB2 | P07550 |
| DS30 | | dan-shexinkum d | DNA topoisomerase II | TOP2A | P11388 |
| DS30 | | dan-shexinkum d | Estrogen receptor beta | ESR2 | Q92731 |
| DS30 | | dan-shexinkum d | Dipeptidyl peptidase IV | DPP4 | P27487 |
| DS30 | | dan-shexinkum d | Glycogen synthase kinase-3 beta | GSK3B | P49841 |
| DS30 | | dan-shexinkum d | Cell division protein kinase 2 | CDK2 | P24941 |
| DS30 | | dan-shexinkum d | Serine/threonine-protein kinase Chk1 | CHEK1 | O14757 |
| DS30 | | dan-shexinkum d | Ig gamma-1 chain C region | IGHG1 | P01857 |
| DS30 | | dan-shexinkum d | Trypsin-1 | PRSS1 | P07477 |
| DS30 | | dan-shexinkum d | Proto-oncogene serine/threonine-protein kinase Pim-1 | PIM1 | P11309 |
| DS30 | | dan-shexinkum d | Cyclin-A2 | CCNA2 | P20248 |
| DS30 | | dan-shexinkum d | Nuclear receptor coactivator 2 | NCOA2 | Q15596 |
| DS30 | | dan-shexinkum d | Nuclear receptor coactivator 1 | NCOA1 | Q15788 |
| DS30 | | dan-shexinkum d | Calmodulin | CALM1 | P0DP23 |
| DS31 | | danshenspiroketallactone | Prostaglandin G/H synthase 1 | PTGS1 | P23219 |
| DS31 | | danshenspiroketallactone | Dopamine D1 receptor | DRD1 | P21728 |
| DS31 | | danshenspiroketallactone | Muscarinic acetylcholine receptor M3 | CHRM3 | P20309 |
| DS31 | | danshenspiroketallactone | Thrombin | F2 | P00734 |
| DS31 | | danshenspiroketallactone | Muscarinic acetylcholine receptor M1 | CHRM1 | P11229 |
| DS31 | | danshenspiroketallactone | Estrogen receptor | ESR1 | P03372 |
| DS31 | | danshenspiroketallactone | Sodium channel protein type 5 subunit alpha | SCN5A | Q14524 |
| DS31 | | danshenspiroketallactone | Muscarinic acetylcholine receptor M5 | CHRM5 | P08912 |
| DS31 | | danshenspiroketallactone | Prostaglandin G/H synthase 2 | PTGS2 | P35354 |
| DS31 | | danshenspiroketallactone | Carbonic anhydrase II | CA2 | P00918 |
| DS31 | | danshenspiroketallactone | Muscarinic acetylcholine receptor M4 | CHRM4 | P08173 |
| DS31 | | danshenspiroketallactone | Retinoic acid receptor RXR-alpha | RXRA | P19793 |
| DS31 | | danshenspiroketallactone | Acetylcholinesterase | ACHE | P22303 |
| DS31 | | danshenspiroketallactone | Alpha-1A adrenergic receptor | ADRA1A | P35348 |
| DS31 | | danshenspiroketallactone | Muscarinic acetylcholine receptor M2 | CHRM2 | P08172 |
| DS31 | | danshenspiroketallactone | Alpha-1B adrenergic receptor | ADRA1B | P35368 |
| DS31 | | danshenspiroketallactone | Beta-2 adrenergic receptor | ADRB2 | P07550 |
| DS31 | | danshenspiroketallactone | Alpha-1D adrenergic receptor | ADRA1D | P25100 |
| DS31 | | danshenspiroketallactone | Neuronal acetylcholine receptor subunit alpha-2 | CHRNA2 | Q15822 |
| DS31 | | danshenspiroketallactone | Sodium-dependent serotonin transporter | SLC6A4 | P31645 |
| DS31 | | danshenspiroketallactone | Mu-type opioid receptor | OPRM1 | P35372 |
| DS31 | | danshenspiroketallactone | Gamma-aminobutyric acid receptor subunit alpha-1 | GABRA1 | P14867 |
| DS31 | | danshenspiroketallactone | Dipeptidyl peptidase IV | DPP4 | P27487 |
| DS31 | | danshenspiroketallactone | Heat shock protein HSP 90 | HSP90AA1 | P07900 |
| DS31 | | danshenspiroketallactone | Neuronal acetylcholine receptor protein, alpha-7 chain | CHRNA7 | P36544 |
| DS32 | | deoxyneocryptotanshinone | Prostaglandin G/H synthase 1 | PTGS1 | P23219 |
| DS32 | | deoxyneocryptotanshinone | Dopamine D1 receptor | DRD1 | P21728 |
| DS32 | | deoxyneocryptotanshinone | Muscarinic acetylcholine receptor M3 | CHRM3 | P20309 |
| DS32 | | deoxyneocryptotanshinone | Muscarinic acetylcholine receptor M1 | CHRM1 | P11229 |
| DS32 | | deoxyneocryptotanshinone | Estrogen receptor | ESR1 | P03372 |
| DS32 | | deoxyneocryptotanshinone | Androgen receptor | AR | P10275 |
| DS32 | | deoxyneocryptotanshinone | Sodium channel protein type 5 subunit alpha | SCN5A | Q14524 |
| DS32 | | deoxyneocryptotanshinone | Muscarinic acetylcholine receptor M5 | CHRM5 | P08912 |
| DS32 | | deoxyneocryptotanshinone | Prostaglandin G/H synthase 2 | PTGS2 | P35354 |
| DS32 | | deoxyneocryptotanshinone | Nitric-oxide synthase, endothelial | NOS3 | P29474 |
| DS32 | | deoxyneocryptotanshinone | Carbonic anhydrase II | CA2 | P00918 |
| DS32 | | deoxyneocryptotanshinone | Muscarinic acetylcholine receptor M4 | CHRM4 | P08173 |
| DS32 | | deoxyneocryptotanshinone | Retinoic acid receptor RXR-alpha | RXRA | P19793 |
| DS32 | | deoxyneocryptotanshinone | Delta-type opioid receptor | OPRD1 | P41143 |
| DS32 | | deoxyneocryptotanshinone | Alpha-1A adrenergic receptor | ADRA1A | P35348 |
| DS32 | | deoxyneocryptotanshinone | Muscarinic acetylcholine receptor M2 | CHRM2 | P08172 |
| DS32 | | deoxyneocryptotanshinone | Alpha-1B adrenergic receptor | ADRA1B | P35368 |
| DS32 | | deoxyneocryptotanshinone | Beta-2 adrenergic receptor | ADRB2 | P07550 |
| DS32 | | deoxyneocryptotanshinone | Alpha-1D adrenergic receptor | ADRA1D | P25100 |
| DS32 | | deoxyneocryptotanshinone | DNA topoisomerase II | TOP2A | P11388 |
| DS32 | | deoxyneocryptotanshinone | Mu-type opioid receptor | OPRM1 | P35372 |
| DS32 | | deoxyneocryptotanshinone | Glycogen synthase kinase-3 beta | GSK3B | P49841 |
| DS32 | | deoxyneocryptotanshinone | Cell division protein kinase 2 | CDK2 | P24941 |
| DS32 | | deoxyneocryptotanshinone | Neuronal acetylcholine receptor protein, alpha-7 chain | CHRNA7 | P36544 |
| DS32 | | deoxyneocryptotanshinone | Ig gamma-1 chain C region | IGHG1 | P01857 |
| DS32 | | deoxyneocryptotanshinone | Proto-oncogene serine/threonine-protein kinase Pim-1 | PIM1 | P11309 |
| DS32 | | deoxyneocryptotanshinone | Nuclear receptor coactivator 2 | NCOA2 | Q15596 |
| DS32 | | deoxyneocryptotanshinone | Nuclear receptor coactivator 1 | NCOA1 | Q15788 |
| DS33 | | dihydrotanshinlactone | Nitric oxide synthase, inducible | NOS2 | P35228 |
| DS33 | | dihydrotanshinlactone | Prostaglandin G/H synthase 1 | PTGS1 | P23219 |
| DS33 | | dihydrotanshinlactone | Dopamine D1 receptor | DRD1 | P21728 |
| DS33 | | dihydrotanshinlactone | Muscarinic acetylcholine receptor M3 | CHRM3 | P20309 |
| DS33 | | dihydrotanshinlactone | Thrombin | F2 | P00734 |
| DS33 | | dihydrotanshinlactone | Muscarinic acetylcholine receptor M1 | CHRM1 | P11229 |
| DS33 | | dihydrotanshinlactone | Estrogen receptor | ESR1 | P03372 |
| DS33 | | dihydrotanshinlactone | Androgen receptor | AR | P10275 |
| DS33 | | dihydrotanshinlactone | Sodium channel protein type 5 subunit alpha | SCN5A | Q14524 |
| DS33 | | dihydrotanshinlactone | Peroxisome proliferator activated receptor gamma | PPARG | P37231 |
| DS33 | | dihydrotanshinlactone | Muscarinic acetylcholine receptor M5 | CHRM5 | P08912 |
| DS33 | | dihydrotanshinlactone | Prostaglandin G/H synthase 2 | PTGS2 | P35354 |
| DS33 | | dihydrotanshinlactone | 5-hydroxytryptamine receptor 3A | HTR3A | P46098 |
| DS33 | | dihydrotanshinlactone | Carbonic anhydrase II | CA2 | P00918 |
| DS33 | | dihydrotanshinlactone | Retinoic acid receptor RXR-alpha | RXRA | P19793 |
| DS33 | | dihydrotanshinlactone | Acetylcholinesterase | ACHE | P22303 |
| DS33 | | dihydrotanshinlactone | CGMP-inhibited 3',5'-cyclic phosphodiesterase A | PDE3A | Q14432 |
| DS33 | | dihydrotanshinlactone | 5-hydroxytryptamine 2A receptor | HTR2A | P28223 |
| DS33 | | dihydrotanshinlactone | Alpha-1A adrenergic receptor | ADRA1A | P35348 |
| DS33 | | dihydrotanshinlactone | Alpha-1B adrenergic receptor | ADRA1B | P35368 |
| DS33 | | dihydrotanshinlactone | mRNA of Protein-tyrosine phosphatase, non-receptor type 1 | PTPN1 | P18031 |
| DS33 | | dihydrotanshinlactone | Sodium-dependent dopamine transporter | SLC6A3 | Q01959 |
| DS33 | | dihydrotanshinlactone | Beta-2 adrenergic receptor | ADRB2 | P07550 |
| DS33 | | dihydrotanshinlactone | Alpha-1D adrenergic receptor | ADRA1D | P25100 |
| DS33 | | dihydrotanshinlactone | Sodium-dependent serotonin transporter | SLC6A4 | P31645 |
| DS33 | | dihydrotanshinlactone | Mu-type opioid receptor | OPRM1 | P35372 |
| DS33 | | dihydrotanshinlactone | Gamma-aminobutyric acid receptor subunit alpha-1 | GABRA1 | P14867 |
| DS33 | | dihydrotanshinlactone | Dipeptidyl peptidase IV | DPP4 | P27487 |
| DS33 | | dihydrotanshinlactone | Glycogen synthase kinase-3 beta | GSK3B | P49841 |
| DS33 | | dihydrotanshinlactone | Phosphatidylinositol-4,5-bisphosphate 3-kinase catalytic subunit, gamma isoform | PIK3CG | P48736 |
| DS33 | | dihydrotanshinlactone | Neuronal acetylcholine receptor protein, alpha-7 chain | CHRNA7 | P36544 |
| DS33 | | dihydrotanshinlactone | mRNA of PKA Catalytic Subunit C-alpha | PRKACA | P17612 |
| DS33 | | dihydrotanshinlactone | Ig gamma-1 chain C region | IGHG1 | P01857 |
| DS33 | | dihydrotanshinlactone | Trypsin-1 | PRSS1 | P07477 |
| DS33 | | dihydrotanshinlactone | Proto-oncogene serine/threonine-protein kinase Pim-1 | PIM1 | P11309 |
| DS33 | | dihydrotanshinlactone | Cyclin-A2 | CCNA2 | P20248 |
| DS34 | | dihydrotanshinoneⅠ | Prostaglandin G/H synthase 1 | PTGS1 | P23219 |
| DS34 | | dihydrotanshinoneⅠ | Sodium channel protein type 5 subunit alpha | SCN5A | Q14524 |
| DS34 | | dihydrotanshinoneⅠ | Prostaglandin G/H synthase 2 | PTGS2 | P35354 |
| DS34 | | dihydrotanshinoneⅠ | 5-hydroxytryptamine receptor 3A | HTR3A | P46098 |
| DS34 | | dihydrotanshinoneⅠ | Retinoic acid receptor RXR-alpha | RXRA | P19793 |
| DS34 | | dihydrotanshinoneⅠ | Alpha-1A adrenergic receptor | ADRA1A | P35348 |
| DS34 | | dihydrotanshinoneⅠ | Alpha-1B adrenergic receptor | ADRA1B | P35368 |
| DS34 | | dihydrotanshinoneⅠ | Beta-2 adrenergic receptor | ADRB2 | P07550 |
| DS34 | | dihydrotanshinoneⅠ | Gamma-aminobutyric acid receptor subunit alpha-1 | GABRA1 | P14867 |
| DS34 | | dihydrotanshinoneⅠ | Heat shock protein HSP 90 | HSP90AA1 | P07900 |
| DS34 | | dihydrotanshinoneⅠ | Phosphatidylinositol-4,5-bisphosphate 3-kinase catalytic subunit, gamma isoform | PIK3CG | P48736 |
| DS34 | | dihydrotanshinoneⅠ | Neuronal acetylcholine receptor protein, alpha-7 chain | CHRNA7 | P36544 |
| DS34 | | dihydrotanshinoneⅠ | mRNA of PKA Catalytic Subunit C-alpha | PRKACA | P17612 |
| DS34 | | dihydrotanshinoneⅠ | Ig gamma-1 chain C region | IGHG1 | P01857 |
| DS34 | | dihydrotanshinoneⅠ | Nuclear receptor coactivator 2 | NCOA2 | Q15596 |
| DS34 | | dihydrotanshinoneⅠ | Nuclear receptor coactivator 1 | NCOA1 | Q15788 |
| DS34 | | dihydrotanshinoneⅠ | Calmodulin | CALM1 | P0DP23 |
| DS35 | | epidanshenspiroketallactone | Prostaglandin G/H synthase 1 | PTGS1 | P23219 |
| DS35 | | epidanshenspiroketallactone | Dopamine D1 receptor | DRD1 | P21728 |
| DS35 | | epidanshenspiroketallactone | Muscarinic acetylcholine receptor M3 | CHRM3 | P20309 |
| DS35 | | epidanshenspiroketallactone | Muscarinic acetylcholine receptor M1 | CHRM1 | P11229 |
| DS35 | | epidanshenspiroketallactone | Estrogen receptor | ESR1 | P03372 |
| DS35 | | epidanshenspiroketallactone | Sodium channel protein type 5 subunit alpha | SCN5A | Q14524 |
| DS35 | | epidanshenspiroketallactone | Muscarinic acetylcholine receptor M5 | CHRM5 | P08912 |
| DS35 | | epidanshenspiroketallactone | Prostaglandin G/H synthase 2 | PTGS2 | P35354 |
| DS35 | | epidanshenspiroketallactone | Nitric-oxide synthase, endothelial | NOS3 | P29474 |
| DS35 | | epidanshenspiroketallactone | Muscarinic acetylcholine receptor M4 | CHRM4 | P08173 |
| DS35 | | epidanshenspiroketallactone | Retinoic acid receptor RXR-alpha | RXRA | P19793 |
| DS35 | | epidanshenspiroketallactone | Delta-type opioid receptor | OPRD1 | P41143 |
| DS35 | | epidanshenspiroketallactone | CGMP-inhibited 3',5'-cyclic phosphodiesterase A | PDE3A | Q14432 |
| DS35 | | epidanshenspiroketallactone | 5-hydroxytryptamine 2A receptor | HTR2A | P28223 |
| DS35 | | epidanshenspiroketallactone | Alpha-1A adrenergic receptor | ADRA1A | P35348 |
| DS35 | | epidanshenspiroketallactone | Muscarinic acetylcholine receptor M2 | CHRM2 | P08172 |
| DS35 | | epidanshenspiroketallactone | Alpha-1B adrenergic receptor | ADRA1B | P35368 |
| DS35 | | epidanshenspiroketallactone | Beta-2 adrenergic receptor | ADRB2 | P07550 |
| DS35 | | epidanshenspiroketallactone | Alpha-1D adrenergic receptor | ADRA1D | P25100 |
| DS35 | | epidanshenspiroketallactone | Sodium-dependent serotonin transporter | SLC6A4 | P31645 |
| DS35 | | epidanshenspiroketallactone | Mu-type opioid receptor | OPRM1 | P35372 |
| DS35 | | epidanshenspiroketallactone | Gamma-aminobutyric acid receptor subunit alpha-1 | GABRA1 | P14867 |
| DS35 | | epidanshenspiroketallactone | Heat shock protein HSP 90 | HSP90AA1 | P07900 |
| DS35 | | epidanshenspiroketallactone | Cell division protein kinase 2 | CDK2 | P24941 |
| DS35 | | epidanshenspiroketallactone | Neuronal acetylcholine receptor protein, alpha-7 chain | CHRNA7 | P36544 |
| DS35 | | epidanshenspiroketallactone | Proto-oncogene serine/threonine-protein kinase Pim-1 | PIM1 | P11309 |
| DS36 | | C09092 | Muscarinic acetylcholine receptor M3 | CHRM3 | P20309 |
| DS36 | | C09092 | Thrombin | F2 | P00734 |
| DS36 | | C09092 | Muscarinic acetylcholine receptor M1 | CHRM1 | P11229 |
| DS36 | | C09092 | Sodium channel protein type 5 subunit alpha | SCN5A | Q14524 |
| DS36 | | C09092 | Carbonic anhydrase II | CA2 | P00918 |
| DS36 | | C09092 | Acetylcholinesterase | ACHE | P22303 |
| DS36 | | C09092 | Alpha-1A adrenergic receptor | ADRA1A | P35348 |
| DS36 | | C09092 | Muscarinic acetylcholine receptor M2 | CHRM2 | P08172 |
| DS36 | | C09092 | Alpha-1B adrenergic receptor | ADRA1B | P35368 |
| DS36 | | C09092 | Beta-2 adrenergic receptor | ADRB2 | P07550 |
| DS36 | | C09092 | Alpha-1D adrenergic receptor | ADRA1D | P25100 |
| DS36 | | C09092 | Mu-type opioid receptor | OPRM1 | P35372 |
| DS37 | | isocryptotanshi-none | Nitric oxide synthase, inducible | NOS2 | P35228 |
| DS37 | | isocryptotanshi-none | Prostaglandin G/H synthase 1 | PTGS1 | P23219 |
| DS37 | | isocryptotanshi-none | Dopamine D1 receptor | DRD1 | P21728 |
| DS37 | | isocryptotanshi-none | Muscarinic acetylcholine receptor M3 | CHRM3 | P20309 |
| DS37 | | isocryptotanshi-none | Muscarinic acetylcholine receptor M1 | CHRM1 | P11229 |
| DS37 | | isocryptotanshi-none | Estrogen receptor | ESR1 | P03372 |
| DS37 | | isocryptotanshi-none | Androgen receptor | AR | P10275 |
| DS37 | | isocryptotanshi-none | Sodium channel protein type 5 subunit alpha | SCN5A | Q14524 |
| DS37 | | isocryptotanshi-none | Coagulation factor Xa | F10 | P00742 |
| DS37 | | isocryptotanshi-none | Muscarinic acetylcholine receptor M5 | CHRM5 | P08912 |
| DS37 | | isocryptotanshi-none | Prostaglandin G/H synthase 2 | PTGS2 | P35354 |
| DS37 | | isocryptotanshi-none | Carbonic anhydrase II | CA2 | P00918 |
| DS37 | | isocryptotanshi-none | Muscarinic acetylcholine receptor M4 | CHRM4 | P08173 |
| DS37 | | isocryptotanshi-none | Retinoic acid receptor RXR-alpha | RXRA | P19793 |
| DS37 | | isocryptotanshi-none | Delta-type opioid receptor | OPRD1 | P41143 |
| DS37 | | isocryptotanshi-none | Acetylcholinesterase | ACHE | P22303 |
| DS37 | | isocryptotanshi-none | Alpha-1A adrenergic receptor | ADRA1A | P35348 |
| DS37 | | isocryptotanshi-none | Muscarinic acetylcholine receptor M2 | CHRM2 | P08172 |
| DS37 | | isocryptotanshi-none | Alpha-1B adrenergic receptor | ADRA1B | P35368 |
| DS37 | | isocryptotanshi-none | Beta-2 adrenergic receptor | ADRB2 | P07550 |
| DS37 | | isocryptotanshi-none | Alpha-1D adrenergic receptor | ADRA1D | P25100 |
| DS37 | | isocryptotanshi-none | DNA topoisomerase II | TOP2A | P11388 |
| DS37 | | isocryptotanshi-none | D(2) dopamine receptor | DRD2 | P14416 |
| DS37 | | isocryptotanshi-none | Mu-type opioid receptor | OPRM1 | P35372 |
| DS37 | | isocryptotanshi-none | Gamma-aminobutyric acid receptor subunit alpha-1 | GABRA1 | P14867 |
| DS37 | | isocryptotanshi-none | Cell division protein kinase 2 | CDK2 | P24941 |
| DS37 | | isocryptotanshi-none | Neuronal acetylcholine receptor protein, alpha-7 chain | CHRNA7 | P36544 |
| DS37 | | isocryptotanshi-none | Trypsin-1 | PRSS1 | P07477 |
| DS37 | | isocryptotanshi-none | Proto-oncogene serine/threonine-protein kinase Pim-1 | PIM1 | P11309 |
| DS37 | | isocryptotanshi-none | Nuclear receptor coactivator 2 | NCOA2 | Q15596 |
| DS37 | | isocryptotanshi-none | Nuclear receptor coactivator 1 | NCOA1 | Q15788 |
| DS38 | | Isotanshinone II | Nitric oxide synthase, inducible | NOS2 | P35228 |
| DS38 | | Isotanshinone II | Dopamine D1 receptor | DRD1 | P21728 |
| DS38 | | Isotanshinone II | Muscarinic acetylcholine receptor M3 | CHRM3 | P20309 |
| DS38 | | Isotanshinone II | Thrombin | F2 | P00734 |
| DS38 | | Isotanshinone II | Muscarinic acetylcholine receptor M1 | CHRM1 | P11229 |
| DS38 | | Isotanshinone II | Estrogen receptor | ESR1 | P03372 |
| DS38 | | Isotanshinone II | Androgen receptor | AR | P10275 |
| DS38 | | Isotanshinone II | Sodium channel protein type 5 subunit alpha | SCN5A | Q14524 |
| DS38 | | Isotanshinone II | Muscarinic acetylcholine receptor M5 | CHRM5 | P08912 |
| DS38 | | Isotanshinone II | Prostaglandin G/H synthase 2 | PTGS2 | P35354 |
| DS38 | | Isotanshinone II | Retinoic acid receptor RXR-alpha | RXRA | P19793 |
| DS38 | | Isotanshinone II | Delta-type opioid receptor | OPRD1 | P41143 |
| DS38 | | Isotanshinone II | Acetylcholinesterase | ACHE | P22303 |
| DS38 | | Isotanshinone II | Alpha-1A adrenergic receptor | ADRA1A | P35348 |
| DS38 | | Isotanshinone II | Muscarinic acetylcholine receptor M2 | CHRM2 | P08172 |
| DS38 | | Isotanshinone II | Beta-2 adrenergic receptor | ADRB2 | P07550 |
| DS38 | | Isotanshinone II | Mu-type opioid receptor | OPRM1 | P35372 |
| DS38 | | Isotanshinone II | Estrogen receptor beta | ESR2 | Q92731 |
| DS38 | | Isotanshinone II | Gamma-aminobutyric acid receptor subunit alpha-1 | GABRA1 | P14867 |
| DS38 | | Isotanshinone II | Dipeptidyl peptidase IV | DPP4 | P27487 |
| DS38 | | Isotanshinone II | Glycogen synthase kinase-3 beta | GSK3B | P49841 |
| DS38 | | Isotanshinone II | Cell division protein kinase 2 | CDK2 | P24941 |
| DS38 | | Isotanshinone II | Neuronal acetylcholine receptor protein, alpha-7 chain | CHRNA7 | P36544 |
| DS38 | | Isotanshinone II | Serine/threonine-protein kinase Chk1 | CHEK1 | O14757 |
| DS38 | | Isotanshinone II | Proto-oncogene serine/threonine-protein kinase Pim-1 | PIM1 | P11309 |
| DS38 | | Isotanshinone II | Cyclin-A2 | CCNA2 | P20248 |
| DS39 | | manool | Nuclear receptor coactivator 2 | NCOA2 | Q15596 |
| DS40 | | miltionone Ⅰ | Prostaglandin G/H synthase 1 | PTGS1 | P23219 |
| DS40 | | miltionone Ⅰ | Muscarinic acetylcholine receptor M3 | CHRM3 | P20309 |
| DS40 | | miltionone Ⅰ | Muscarinic acetylcholine receptor M1 | CHRM1 | P11229 |
| DS40 | | miltionone Ⅰ | Estrogen receptor | ESR1 | P03372 |
| DS40 | | miltionone Ⅰ | Androgen receptor | AR | P10275 |
| DS40 | | miltionone Ⅰ | Sodium channel protein type 5 subunit alpha | SCN5A | Q14524 |
| DS40 | | miltionone Ⅰ | Coagulation factor Xa | F10 | P00742 |
| DS40 | | miltionone Ⅰ | Prostaglandin G/H synthase 2 | PTGS2 | P35354 |
| DS40 | | miltionone Ⅰ | Carbonic anhydrase II | CA2 | P00918 |
| DS40 | | miltionone Ⅰ | Retinoic acid receptor RXR-alpha | RXRA | P19793 |
| DS40 | | miltionone Ⅰ | Delta-type opioid receptor | OPRD1 | P41143 |
| DS40 | | miltionone Ⅰ | 5-hydroxytryptamine 2A receptor | HTR2A | P28223 |
| DS40 | | miltionone Ⅰ | Alpha-1A adrenergic receptor | ADRA1A | P35348 |
| DS40 | | miltionone Ⅰ | Muscarinic acetylcholine receptor M2 | CHRM2 | P08172 |
| DS40 | | miltionone Ⅰ | Alpha-1B adrenergic receptor | ADRA1B | P35368 |
| DS40 | | miltionone Ⅰ | Beta-2 adrenergic receptor | ADRB2 | P07550 |
| DS40 | | miltionone Ⅰ | DNA topoisomerase II | TOP2A | P11388 |
| DS40 | | miltionone Ⅰ | Mu-type opioid receptor | OPRM1 | P35372 |
| DS40 | | miltionone Ⅰ | Glucocorticoid receptor | NR3C1 | P04150 |
| DS40 | | miltionone Ⅰ | Glycogen synthase kinase-3 beta | GSK3B | P49841 |
| DS40 | | miltionone Ⅰ | Cell division protein kinase 2 | CDK2 | P24941 |
| DS40 | | miltionone Ⅰ | Beta-lactamase | DPEP1 | P16444 |
| DS40 | | miltionone Ⅰ | Neuronal acetylcholine receptor protein, alpha-7 chain | CHRNA7 | P36544 |
| DS40 | | miltionone Ⅰ | Ig gamma-1 chain C region | IGHG1 | P01857 |
| DS40 | | miltionone Ⅰ | Proto-oncogene serine/threonine-protein kinase Pim-1 | PIM1 | P11309 |
| DS40 | | miltionone Ⅰ | Cyclin-A2 | CCNA2 | P20248 |
| DS40 | | miltionone Ⅰ | Nuclear receptor coactivator 2 | NCOA2 | Q15596 |
| DS40 | | miltionone Ⅰ | Nuclear receptor coactivator 1 | NCOA1 | Q15788 |
| DS41 | | miltionone Ⅱ | Thrombin | F2 | P00734 |
| DS41 | | miltionone Ⅱ | Prostaglandin G/H synthase 2 | PTGS2 | P35354 |
| DS41 | | miltionone Ⅱ | Carbonic anhydrase II | CA2 | P00918 |
| DS41 | | miltionone Ⅱ | Acetylcholinesterase | ACHE | P22303 |
| DS41 | | miltionone Ⅱ | Progesterone receptor | PGR | P06401 |
| DS41 | | miltionone Ⅱ | Glucocorticoid receptor | NR3C1 | P04150 |
| DS41 | | miltionone Ⅱ | Nuclear receptor coactivator 2 | NCOA2 | Q15596 |
| DS41 | | miltionone Ⅱ | Nuclear receptor coactivator 1 | NCOA1 | Q15788 |
| DS42 | | miltipolone | Estrogen receptor | ESR1 | P03372 |
| DS42 | | miltipolone | Acetylcholinesterase | ACHE | P22303 |
| DS43 | | Miltirone | Prostaglandin G/H synthase 1 | PTGS1 | P23219 |
| DS43 | | Miltirone | Dopamine D1 receptor | DRD1 | P21728 |
| DS43 | | Miltirone | Muscarinic acetylcholine receptor M3 | CHRM3 | P20309 |
| DS43 | | Miltirone | Muscarinic acetylcholine receptor M1 | CHRM1 | P11229 |
| DS43 | | Miltirone | Estrogen receptor | ESR1 | P03372 |
| DS43 | | Miltirone | Androgen receptor | AR | P10275 |
| DS43 | | Miltirone | D(1B) dopamine receptor | DRD5 | P21918 |
| DS43 | | Miltirone | Sodium channel protein type 5 subunit alpha | SCN5A | Q14524 |
| DS43 | | Miltirone | Muscarinic acetylcholine receptor M5 | CHRM5 | P08912 |
| DS43 | | Miltirone | Prostaglandin G/H synthase 2 | PTGS2 | P35354 |
| DS43 | | Miltirone | Nitric-oxide synthase, endothelial | NOS3 | P29474 |
| DS43 | | Miltirone | Carbonic anhydrase II | CA2 | P00918 |
| DS43 | | Miltirone | Alpha-2C adrenergic receptor | ADRA2C | P18825 |
| DS43 | | Miltirone | Muscarinic acetylcholine receptor M4 | CHRM4 | P08173 |
| DS43 | | Miltirone | Retinoic acid receptor RXR-alpha | RXRA | P19793 |
| DS43 | | Miltirone | Delta-type opioid receptor | OPRD1 | P41143 |
| DS43 | | Miltirone | Alpha-1A adrenergic receptor | ADRA1A | P35348 |
| DS43 | | Miltirone | Muscarinic acetylcholine receptor M2 | CHRM2 | P08172 |
| DS43 | | Miltirone | Alpha-1B adrenergic receptor | ADRA1B | P35368 |
| DS43 | | Miltirone | Sodium-dependent dopamine transporter | SLC6A3 | Q01959 |
| DS43 | | Miltirone | Beta-2 adrenergic receptor | ADRB2 | P07550 |
| DS43 | | Miltirone | Alpha-1D adrenergic receptor | ADRA1D | P25100 |
| DS43 | | Miltirone | DNA topoisomerase II | TOP2A | P11388 |
| DS43 | | Miltirone | Mu-type opioid receptor | OPRM1 | P35372 |
| DS43 | | Miltirone | Neuronal acetylcholine receptor protein, alpha-7 chain | CHRNA7 | P36544 |
| DS43 | | Miltirone | Nuclear receptor coactivator 2 | NCOA2 | Q15596 |
| DS44 | | neocryptotanshinone ii | Prostaglandin G/H synthase 1 | PTGS1 | P23219 |
| DS44 | | neocryptotanshinone ii | Dopamine D1 receptor | DRD1 | P21728 |
| DS44 | | neocryptotanshinone ii | Muscarinic acetylcholine receptor M3 | CHRM3 | P20309 |
| DS44 | | neocryptotanshinone ii | Muscarinic acetylcholine receptor M1 | CHRM1 | P11229 |
| DS44 | | neocryptotanshinone ii | Estrogen receptor | ESR1 | P03372 |
| DS44 | | neocryptotanshinone ii | Androgen receptor | AR | P10275 |
| DS44 | | neocryptotanshinone ii | Sodium channel protein type 5 subunit alpha | SCN5A | Q14524 |
| DS44 | | neocryptotanshinone ii | Prostaglandin G/H synthase 2 | PTGS2 | P35354 |
| DS44 | | neocryptotanshinone ii | Nitric-oxide synthase, endothelial | NOS3 | P29474 |
| DS44 | | neocryptotanshinone ii | Carbonic anhydrase II | CA2 | P00918 |
| DS44 | | neocryptotanshinone ii | Muscarinic acetylcholine receptor M4 | CHRM4 | P08173 |
| DS44 | | neocryptotanshinone ii | Retinoic acid receptor RXR-alpha | RXRA | P19793 |
| DS44 | | neocryptotanshinone ii | Delta-type opioid receptor | OPRD1 | P41143 |
| DS44 | | neocryptotanshinone ii | CGMP-inhibited 3',5'-cyclic phosphodiesterase A | PDE3A | Q14432 |
| DS44 | | neocryptotanshinone ii | Alpha-1A adrenergic receptor | ADRA1A | P35348 |
| DS44 | | neocryptotanshinone ii | Muscarinic acetylcholine receptor M2 | CHRM2 | P08172 |
| DS44 | | neocryptotanshinone ii | Alpha-1B adrenergic receptor | ADRA1B | P35368 |
| DS44 | | neocryptotanshinone ii | Sodium-dependent dopamine transporter | SLC6A3 | Q01959 |
| DS44 | | neocryptotanshinone ii | Beta-2 adrenergic receptor | ADRB2 | P07550 |
| DS44 | | neocryptotanshinone ii | Alpha-1D adrenergic receptor | ADRA1D | P25100 |
| DS44 | | neocryptotanshinone ii | Sodium-dependent serotonin transporter | SLC6A4 | P31645 |
| DS44 | | neocryptotanshinone ii | Mu-type opioid receptor | OPRM1 | P35372 |
| DS44 | | neocryptotanshinone ii | Gamma-aminobutyric acid receptor subunit alpha-1 | GABRA1 | P14867 |
| DS44 | | neocryptotanshinone ii | Glycogen synthase kinase-3 beta | GSK3B | P49841 |
| DS44 | | neocryptotanshinone ii | Heat shock protein HSP 90 | HSP90AA1 | P07900 |
| DS44 | | neocryptotanshinone ii | Cell division protein kinase 2 | CDK2 | P24941 |
| DS44 | | neocryptotanshinone ii | Neuronal acetylcholine receptor protein, alpha-7 chain | CHRNA7 | P36544 |
| DS44 | | neocryptotanshinone ii | Proto-oncogene serine/threonine-protein kinase Pim-1 | PIM1 | P11309 |
| DS44 | | neocryptotanshinone ii | Cyclin-A2 | CCNA2 | P20248 |
| DS45 | | neocryptotanshinone | Prostaglandin G/H synthase 1 | PTGS1 | P23219 |
| DS45 | | neocryptotanshinone | Muscarinic acetylcholine receptor M3 | CHRM3 | P20309 |
| DS45 | | neocryptotanshinone | Muscarinic acetylcholine receptor M1 | CHRM1 | P11229 |
| DS45 | | neocryptotanshinone | Sodium channel protein type 5 subunit alpha | SCN5A | Q14524 |
| DS45 | | neocryptotanshinone | Peroxisome proliferator activated receptor gamma | PPARG | P37231 |
| DS45 | | neocryptotanshinone | Prostaglandin G/H synthase 2 | PTGS2 | P35354 |
| DS45 | | neocryptotanshinone | Carbonic anhydrase II | CA2 | P00918 |
| DS45 | | neocryptotanshinone | Alpha-1B adrenergic receptor | ADRA1B | P35368 |
| DS45 | | neocryptotanshinone | Beta-2 adrenergic receptor | ADRB2 | P07550 |
| DS45 | | neocryptotanshinone | Alpha-1D adrenergic receptor | ADRA1D | P25100 |
| DS45 | | neocryptotanshinone | DNA topoisomerase II | TOP2A | P11388 |
| DS45 | | neocryptotanshinone | Mu-type opioid receptor | OPRM1 | P35372 |
| DS45 | | neocryptotanshinone | Neuronal acetylcholine receptor protein, alpha-7 chain | CHRNA7 | P36544 |
| DS45 | | neocryptotanshinone | Ig gamma-1 chain C region | IGHG1 | P01857 |
| DS45 | | neocryptotanshinone | Nuclear receptor coactivator 2 | NCOA2 | Q15596 |
| DS45 | | neocryptotanshinone | Nuclear receptor coactivator 1 | NCOA1 | Q15788 |
| DS46 | | 1-methyl-8,9-dihydro-7H-naphtho[5,6-g]benzofuran-6,10,11-trione | Prostaglandin G/H synthase 1 | PTGS1 | P23219 |
| DS46 | | 1-methyl-8,9-dihydro-7H-naphtho[5,6-g]benzofuran-6,10,11-trione | Dopamine D1 receptor | DRD1 | P21728 |
| DS46 | | 1-methyl-8,9-dihydro-7H-naphtho[5,6-g]benzofuran-6,10,11-trione | Muscarinic acetylcholine receptor M3 | CHRM3 | P20309 |
| DS46 | | 1-methyl-8,9-dihydro-7H-naphtho[5,6-g]benzofuran-6,10,11-trione | Thrombin | F2 | P00734 |
| DS46 | | 1-methyl-8,9-dihydro-7H-naphtho[5,6-g]benzofuran-6,10,11-trione | Sodium channel protein type 5 subunit alpha | SCN5A | Q14524 |
| DS46 | | 1-methyl-8,9-dihydro-7H-naphtho[5,6-g]benzofuran-6,10,11-trione | Muscarinic acetylcholine receptor M5 | CHRM5 | P08912 |
| DS46 | | 1-methyl-8,9-dihydro-7H-naphtho[5,6-g]benzofuran-6,10,11-trione | Prostaglandin G/H synthase 2 | PTGS2 | P35354 |
| DS46 | | 1-methyl-8,9-dihydro-7H-naphtho[5,6-g]benzofuran-6,10,11-trione | Carbonic anhydrase II | CA2 | P00918 |
| DS46 | | 1-methyl-8,9-dihydro-7H-naphtho[5,6-g]benzofuran-6,10,11-trione | Retinoic acid receptor RXR-alpha | RXRA | P19793 |
| DS46 | | 1-methyl-8,9-dihydro-7H-naphtho[5,6-g]benzofuran-6,10,11-trione | Acetylcholinesterase | ACHE | P22303 |
| DS46 | | 1-methyl-8,9-dihydro-7H-naphtho[5,6-g]benzofuran-6,10,11-trione | Alpha-1A adrenergic receptor | ADRA1A | P35348 |
| DS46 | | 1-methyl-8,9-dihydro-7H-naphtho[5,6-g]benzofuran-6,10,11-trione | Beta-2 adrenergic receptor | ADRB2 | P07550 |
| DS46 | | 1-methyl-8,9-dihydro-7H-naphtho[5,6-g]benzofuran-6,10,11-trione | Mu-type opioid receptor | OPRM1 | P35372 |
| DS46 | | 1-methyl-8,9-dihydro-7H-naphtho[5,6-g]benzofuran-6,10,11-trione | Gamma-aminobutyric acid receptor subunit alpha-1 | GABRA1 | P14867 |
| DS46 | | 1-methyl-8,9-dihydro-7H-naphtho[5,6-g]benzofuran-6,10,11-trione | Dipeptidyl peptidase IV | DPP4 | P27487 |
| DS46 | | 1-methyl-8,9-dihydro-7H-naphtho[5,6-g]benzofuran-6,10,11-trione | Heat shock protein HSP 90 | HSP90AA1 | P07900 |
| DS46 | | 1-methyl-8,9-dihydro-7H-naphtho[5,6-g]benzofuran-6,10,11-trione | Phosphatidylinositol-4,5-bisphosphate 3-kinase catalytic subunit, gamma isoform | PIK3CG | P48736 |
| DS46 | | 1-methyl-8,9-dihydro-7H-naphtho[5,6-g]benzofuran-6,10,11-trione | Neuronal acetylcholine receptor protein, alpha-7 chain | CHRNA7 | P36544 |
| DS46 | | 1-methyl-8,9-dihydro-7H-naphtho[5,6-g]benzofuran-6,10,11-trione | Ig gamma-1 chain C region | IGHG1 | P01857 |
| DS46 | | 1-methyl-8,9-dihydro-7H-naphtho[5,6-g]benzofuran-6,10,11-trione | Nuclear receptor coactivator 1 | NCOA1 | Q15788 |
| DS47 | | prolithospermic acid | Nitric oxide synthase, inducible | NOS2 | P35228 |
| DS47 | | prolithospermic acid | Prostaglandin G/H synthase 1 | PTGS1 | P23219 |
| DS47 | | prolithospermic acid | Thrombin | F2 | P00734 |
| DS47 | | prolithospermic acid | Estrogen receptor | ESR1 | P03372 |
| DS47 | | prolithospermic acid | Androgen receptor | AR | P10275 |
| DS47 | | prolithospermic acid | Prostaglandin G/H synthase 2 | PTGS2 | P35354 |
| DS47 | | prolithospermic acid | mRNA of Protein-tyrosine phosphatase, non-receptor type 1 | PTPN1 | P18031 |
| DS47 | | prolithospermic acid | Heat shock protein HSP 90 | HSP90AA1 | P07900 |
| DS47 | | prolithospermic acid | Trypsin-1 | PRSS1 | P07477 |
| DS47 | | prolithospermic acid | Calmodulin | CALM1 | P0DP23 |
| DS48 | | (2R)-3-(3,4-dihydroxyphenyl)-2-[(Z)-3-(3,4-dihydroxyphenyl)acryloyl]oxy-propionic acid | Thrombin | F2 | P00734 |
| DS48 | | (2R)-3-(3,4-dihydroxyphenyl)-2-[(Z)-3-(3,4-dihydroxyphenyl)acryloyl]oxy-propionic acid | Estrogen receptor | ESR1 | P03372 |
| DS48 | | (2R)-3-(3,4-dihydroxyphenyl)-2-[(Z)-3-(3,4-dihydroxyphenyl)acryloyl]oxy-propionic acid | Androgen receptor | AR | P10275 |
| DS48 | | (2R)-3-(3,4-dihydroxyphenyl)-2-[(Z)-3-(3,4-dihydroxyphenyl)acryloyl]oxy-propionic acid | Peroxisome proliferator activated receptor gamma | PPARG | P37231 |
| DS48 | | (2R)-3-(3,4-dihydroxyphenyl)-2-[(Z)-3-(3,4-dihydroxyphenyl)acryloyl]oxy-propionic acid | Prostaglandin G/H synthase 2 | PTGS2 | P35354 |
| DS48 | | (2R)-3-(3,4-dihydroxyphenyl)-2-[(Z)-3-(3,4-dihydroxyphenyl)acryloyl]oxy-propionic acid | Dipeptidyl peptidase IV | DPP4 | P27487 |
| DS48 | | (2R)-3-(3,4-dihydroxyphenyl)-2-[(Z)-3-(3,4-dihydroxyphenyl)acryloyl]oxy-propionic acid | Trypsin-1 | PRSS1 | P07477 |
| DS48 | | (2R)-3-(3,4-dihydroxyphenyl)-2-[(Z)-3-(3,4-dihydroxyphenyl)acryloyl]oxy-propionic acid | Cyclin-A2 | CCNA2 | P20248 |
| DS49 | | salvianolic acid g | Prostaglandin G/H synthase 2 | PTGS2 | P35354 |
| DS50 | | salvianolic acid j | Coagulation factor VII | F7 | P08709 |
| DS50 | | salvianolic acid j | mRNA of Protein-tyrosine phosphatase, non-receptor type 1 | PTPN1 | P18031 |
| DS50 | | salvianolic acid j | Trypsin-1 | PRSS1 | P07477 |
| DS51 | | salvilenone Ⅰ | Prostaglandin G/H synthase 2 | PTGS2 | P35354 |
| DS51 | | salvilenone Ⅰ | Retinoic acid receptor RXR-alpha | RXRA | P19793 |
| DS51 | | salvilenone Ⅰ | Acetylcholinesterase | ACHE | P22303 |
| DS51 | | salvilenone Ⅰ | Progesterone receptor | PGR | P06401 |
| DS51 | | salvilenone Ⅰ | mRNA of Protein-tyrosine phosphatase, non-receptor type 1 | PTPN1 | P18031 |
| DS51 | | salvilenone Ⅰ | Glucocorticoid receptor | NR3C1 | P04150 |
| DS51 | | salvilenone Ⅰ | Nuclear receptor coactivator 2 | NCOA2 | Q15596 |
| DS51 | | salvilenone Ⅰ | Nuclear receptor coactivator 1 | NCOA1 | Q15788 |
| DS52 | | salviolone | Prostaglandin G/H synthase 1 | PTGS1 | P23219 |
| DS52 | | salviolone | Dopamine D1 receptor | DRD1 | P21728 |
| DS52 | | salviolone | Muscarinic acetylcholine receptor M3 | CHRM3 | P20309 |
| DS52 | | salviolone | Thrombin | F2 | P00734 |
| DS52 | | salviolone | Muscarinic acetylcholine receptor M1 | CHRM1 | P11229 |
| DS52 | | salviolone | D(1B) dopamine receptor | DRD5 | P21918 |
| DS52 | | salviolone | Sodium channel protein type 5 subunit alpha | SCN5A | Q14524 |
| DS52 | | salviolone | Muscarinic acetylcholine receptor M5 | CHRM5 | P08912 |
| DS52 | | salviolone | Prostaglandin G/H synthase 2 | PTGS2 | P35354 |
| DS52 | | salviolone | Alpha-2A adrenergic receptor | ADRA2A | P08913 |
| DS52 | | salviolone | 5-hydroxytryptamine 1A receptor | HTR1A | P08908 |
| DS52 | | salviolone | 5-hydroxytryptamine receptor 3A | HTR3A | P46098 |
| DS52 | | salviolone | Gamma-aminobutyric-acid receptor alpha-2 subunit | GABRA2 | P47869 |
| DS52 | | salviolone | Muscarinic acetylcholine receptor M4 | CHRM4 | P08173 |
| DS52 | | salviolone | Delta-type opioid receptor | OPRD1 | P41143 |
| DS52 | | salviolone | Acetylcholinesterase | ACHE | P22303 |
| DS52 | | salviolone | CGMP-inhibited 3',5'-cyclic phosphodiesterase A | PDE3A | Q14432 |
| DS52 | | salviolone | 5-hydroxytryptamine 2A receptor | HTR2A | P28223 |
| DS52 | | salviolone | Gamma-aminobutyric-acid receptor alpha-5 subunit | GABRA5 | P31644 |
| DS52 | | salviolone | Sodium-dependent noradrenaline transporter | SLC6A2 | P23975 |
| DS52 | | salviolone | Alpha-1A adrenergic receptor | ADRA1A | P35348 |
| DS52 | | salviolone | Gamma-aminobutyric-acid receptor alpha-3 subunit | GABRA3 | P34903 |
| DS52 | | salviolone | 5-hydroxytryptamine 2C receptor | HTR2C | P28335 |
| DS52 | | salviolone | Muscarinic acetylcholine receptor M2 | CHRM2 | P08172 |
| DS52 | | salviolone | Alpha-2B adrenergic receptor | ADRA2B | P18089 |
| DS52 | | salviolone | Alpha-1B adrenergic receptor | ADRA1B | P35368 |
| DS52 | | salviolone | Sodium-dependent dopamine transporter | SLC6A3 | Q01959 |
| DS52 | | salviolone | Beta-2 adrenergic receptor | ADRB2 | P07550 |
| DS52 | | salviolone | Neuronal acetylcholine receptor subunit alpha-2 | CHRNA2 | Q15822 |
| DS52 | | salviolone | Sodium-dependent serotonin transporter | SLC6A4 | P31645 |
| DS52 | | salviolone | D(2) dopamine receptor | DRD2 | P14416 |
| DS52 | | salviolone | Mu-type opioid receptor | OPRM1 | P35372 |
| DS52 | | salviolone | Gamma-aminobutyric acid receptor subunit alpha-1 | GABRA1 | P14867 |
| DS52 | | salviolone | 5-hydroxytryptamine 1B receptor | HTR1B | P28222 |
| DS52 | | salviolone | Neuronal acetylcholine receptor protein, alpha-7 chain | CHRNA7 | P36544 |
| DS52 | | salviolone | Gamma-aminobutyric-acid receptor subunit alpha-6 | GABRA6 | Q16445 |
| DS52 | | salviolone | Gamma-aminobutyric acid receptor subunit gamma-3 | GABRG3 | Q99928 |
| DS52 | | salviolone | Gamma-aminobutyric acid receptor subunit epsilon | GABRE | P78334 |
| DS53 | | (6S)-6-hydroxy-1-methyl-6-methylol-8,9-dihydro-7H-naphtho[8,7-g]benzofuran-10,11-quinone | Thrombin | F2 | P00734 |
| DS53 | | (6S)-6-hydroxy-1-methyl-6-methylol-8,9-dihydro-7H-naphtho[8,7-g]benzofuran-10,11-quinone | Prostaglandin G/H synthase 2 | PTGS2 | P35354 |
| DS53 | | (6S)-6-hydroxy-1-methyl-6-methylol-8,9-dihydro-7H-naphtho[8,7-g]benzofuran-10,11-quinone | Carbonic anhydrase II | CA2 | P00918 |
| DS53 | | (6S)-6-hydroxy-1-methyl-6-methylol-8,9-dihydro-7H-naphtho[8,7-g]benzofuran-10,11-quinone | Acetylcholinesterase | ACHE | P22303 |
| DS53 | | (6S)-6-hydroxy-1-methyl-6-methylol-8,9-dihydro-7H-naphtho[8,7-g]benzofuran-10,11-quinone | Dipeptidyl peptidase IV | DPP4 | P27487 |
| DS53 | | (6S)-6-hydroxy-1-methyl-6-methylol-8,9-dihydro-7H-naphtho[8,7-g]benzofuran-10,11-quinone | Heat shock protein HSP 90 | HSP90AA1 | P07900 |
| DS53 | | (6S)-6-hydroxy-1-methyl-6-methylol-8,9-dihydro-7H-naphtho[8,7-g]benzofuran-10,11-quinone | Trypsin-1 | PRSS1 | P07477 |
| DS53 | | (6S)-6-hydroxy-1-methyl-6-methylol-8,9-dihydro-7H-naphtho[8,7-g]benzofuran-10,11-quinone | Nuclear receptor coactivator 1 | NCOA1 | Q15788 |
| DS54 | | Tanshindiol B | Thrombin | F2 | P00734 |
| DS54 | | Tanshindiol B | Prostaglandin G/H synthase 2 | PTGS2 | P35354 |
| DS54 | | Tanshindiol B | Carbonic anhydrase II | CA2 | P00918 |
| DS54 | | Tanshindiol B | Acetylcholinesterase | ACHE | P22303 |
| DS54 | | Tanshindiol B | Dipeptidyl peptidase IV | DPP4 | P27487 |
| DS54 | | Tanshindiol B | Heat shock protein HSP 90 | HSP90AA1 | P07900 |
| DS54 | | Tanshindiol B | Nuclear receptor coactivator 1 | NCOA1 | Q15788 |
| DS55 | | Przewaquinone E | Thrombin | F2 | P00734 |
| DS55 | | Przewaquinone E | Prostaglandin G/H synthase 2 | PTGS2 | P35354 |
| DS55 | | Przewaquinone E | Carbonic anhydrase II | CA2 | P00918 |
| DS55 | | Przewaquinone E | Acetylcholinesterase | ACHE | P22303 |
| DS55 | | Przewaquinone E | Dipeptidyl peptidase IV | DPP4 | P27487 |
| DS55 | | Przewaquinone E | Heat shock protein HSP 90 | HSP90AA1 | P07900 |
| DS55 | | Przewaquinone E | Nuclear receptor coactivator 1 | NCOA1 | Q15788 |
| DS56 | | tanshinone iia | Dopamine D1 receptor | DRD1 | P21728 |
| DS56 | | tanshinone iia | Muscarinic acetylcholine receptor M3 | CHRM3 | P20309 |
| DS56 | | tanshinone iia | Thrombin | F2 | P00734 |
| DS56 | | tanshinone iia | Muscarinic acetylcholine receptor M1 | CHRM1 | P11229 |
| DS56 | | tanshinone iia | Sodium channel protein type 5 subunit alpha | SCN5A | Q14524 |
| DS56 | | tanshinone iia | Muscarinic acetylcholine receptor M5 | CHRM5 | P08912 |
| DS56 | | tanshinone iia | Prostaglandin G/H synthase 2 | PTGS2 | P35354 |
| DS56 | | tanshinone iia | Muscarinic acetylcholine receptor M4 | CHRM4 | P08173 |
| DS56 | | tanshinone iia | Delta-type opioid receptor | OPRD1 | P41143 |
| DS56 | | tanshinone iia | Acetylcholinesterase | ACHE | P22303 |
| DS56 | | tanshinone iia | Alpha-1A adrenergic receptor | ADRA1A | P35348 |
| DS56 | | tanshinone iia | Muscarinic acetylcholine receptor M2 | CHRM2 | P08172 |
| DS56 | | tanshinone iia | Beta-2 adrenergic receptor | ADRB2 | P07550 |
| DS56 | | tanshinone iia | Mu-type opioid receptor | OPRM1 | P35372 |
| DS56 | | tanshinone iia | Dipeptidyl peptidase IV | DPP4 | P27487 |
| DS56 | | tanshinone iia | Neuronal acetylcholine receptor protein, alpha-7 chain | CHRNA7 | P36544 |
| DS56 | | tanshinone iia | Nuclear receptor coactivator 1 | NCOA1 | Q15788 |
| DS56 | | tanshinone iia | Retinoic acid receptor RXR-alpha | RXRA | P19793 |
| DS56 | | tanshinone iia | Transcription factor p65 | RELA | Q04206 |
| DS56 | | tanshinone iia | Apoptosis regulator Bcl-2 | BCL2 | P10415 |
| DS56 | | tanshinone iia | Proto-oncogene c-Fos | FOS | P01100 |
| DS56 | | tanshinone iia | Cyclin-dependent kinase inhibitor 1 | CDKN1A | P38936 |
| DS56 | | tanshinone iia | Matrix metalloproteinase-9 | MMP9 | P14780 |
| DS56 | | tanshinone iia | Transcription factor AP-1 | JUN | P05412 |
| DS56 | | tanshinone iia | Activator of 90 kDa heat shock protein ATPase homolog 1 | AHSA1 | O95433 |
| DS56 | | tanshinone iia | Caspase-3 | CASP3 | P42574 |
| DS56 | | tanshinone iia | Cellular tumor antigen p53 | TP53 | P04637 |
| DS56 | | tanshinone iia | NF-kappa-B inhibitor alpha | NFKBIA | P25963 |
| DS56 | | tanshinone iia | Fatty acid synthase | FASN | P49327 |
| DS56 | | tanshinone iia | Endothelin-1 receptor | EDNRA | P25101 |
| DS56 | | tanshinone iia | Endothelin-1 | EDN1 | P05305 |
| DS56 | | tanshinone iia | Cytochrome P450 3A4 | CYP3A4 | P08684 |
| DS56 | | tanshinone iia | Cytochrome P450 1A2 | CYP1A2 | P05177 |
| DS56 | | tanshinone iia | Myc proto-oncogene protein | MYC | P01106 |
| DS56 | | tanshinone iia | Cytochrome P450 1A1 | CYP1A1 | P04798 |
| DS56 | | tanshinone iia | Nuclear receptor subfamily 1 group I member 2 | NR1I2 | O75469 |
| DS56 | | tanshinone iia | Nucleophosmin | NPM1 | P06748 |
| DS56 | | tanshinone iia | Endothelin-converting enzyme 1 | ECE1 | P42892 |
| DS56 | | tanshinone iia | Poly [ADP-ribose] polymerase 4 | PARP4 | Q9UKK3 |
| DS56 | | tanshinone iia | Calcitonin receptor | CALCR | P30988 |
| DS56 | | tanshinone iia | Integrin beta-3 | ITGB3 | P05106 |
| DS57 | | (6S)-6-(hydroxymethyl)-1,6-dimethyl-8,9-dihydro-7H-naphtho[8,7-g]benzofuran-10,11-dione | Thrombin | F2 | P00734 |
| DS57 | | (6S)-6-(hydroxymethyl)-1,6-dimethyl-8,9-dihydro-7H-naphtho[8,7-g]benzofuran-10,11-dione | Muscarinic acetylcholine receptor M1 | CHRM1 | P11229 |
| DS57 | | (6S)-6-(hydroxymethyl)-1,6-dimethyl-8,9-dihydro-7H-naphtho[8,7-g]benzofuran-10,11-dione | Sodium channel protein type 5 subunit alpha | SCN5A | Q14524 |
| DS57 | | (6S)-6-(hydroxymethyl)-1,6-dimethyl-8,9-dihydro-7H-naphtho[8,7-g]benzofuran-10,11-dione | Prostaglandin G/H synthase 2 | PTGS2 | P35354 |
| DS57 | | (6S)-6-(hydroxymethyl)-1,6-dimethyl-8,9-dihydro-7H-naphtho[8,7-g]benzofuran-10,11-dione | Delta-type opioid receptor | OPRD1 | P41143 |
| DS57 | | (6S)-6-(hydroxymethyl)-1,6-dimethyl-8,9-dihydro-7H-naphtho[8,7-g]benzofuran-10,11-dione | Acetylcholinesterase | ACHE | P22303 |
| DS57 | | (6S)-6-(hydroxymethyl)-1,6-dimethyl-8,9-dihydro-7H-naphtho[8,7-g]benzofuran-10,11-dione | Alpha-1A adrenergic receptor | ADRA1A | P35348 |
| DS57 | | (6S)-6-(hydroxymethyl)-1,6-dimethyl-8,9-dihydro-7H-naphtho[8,7-g]benzofuran-10,11-dione | Beta-2 adrenergic receptor | ADRB2 | P07550 |
| DS57 | | (6S)-6-(hydroxymethyl)-1,6-dimethyl-8,9-dihydro-7H-naphtho[8,7-g]benzofuran-10,11-dione | Mu-type opioid receptor | OPRM1 | P35372 |
| DS57 | | (6S)-6-(hydroxymethyl)-1,6-dimethyl-8,9-dihydro-7H-naphtho[8,7-g]benzofuran-10,11-dione | Dipeptidyl peptidase IV | DPP4 | P27487 |
| DS57 | | (6S)-6-(hydroxymethyl)-1,6-dimethyl-8,9-dihydro-7H-naphtho[8,7-g]benzofuran-10,11-dione | Neuronal acetylcholine receptor protein, alpha-7 chain | CHRNA7 | P36544 |
| DS57 | | (6S)-6-(hydroxymethyl)-1,6-dimethyl-8,9-dihydro-7H-naphtho[8,7-g]benzofuran-10,11-dione | Trypsin-1 | PRSS1 | P07477 |
| DS57 | | (6S)-6-(hydroxymethyl)-1,6-dimethyl-8,9-dihydro-7H-naphtho[8,7-g]benzofuran-10,11-dione | Nuclear receptor coactivator 1 | NCOA1 | Q15788 |
| DS58 | | tanshinone Ⅵ | Prostaglandin G/H synthase 1 | PTGS1 | P23219 |
| DS58 | | tanshinone Ⅵ | Estrogen receptor | ESR1 | P03372 |
| DS58 | | tanshinone Ⅵ | Androgen receptor | AR | P10275 |
| DS58 | | tanshinone Ⅵ | Sodium channel protein type 5 subunit alpha | SCN5A | Q14524 |
| DS58 | | tanshinone Ⅵ | Peroxisome proliferator activated receptor gamma | PPARG | P37231 |
| DS58 | | tanshinone Ⅵ | Coagulation factor Xa | F10 | P00742 |
| DS58 | | tanshinone Ⅵ | Prostaglandin G/H synthase 2 | PTGS2 | P35354 |
| DS58 | | tanshinone Ⅵ | Heat shock protein HSP 90 | HSP90AA1 | P07900 |
| DS58 | | tanshinone Ⅵ | Beta-lactamase | DPEP1 | P16444 |
| DS58 | | tanshinone Ⅵ | Ig gamma-1 chain C region | IGHG1 | P01857 |
| DS58 | | tanshinone Ⅵ | Nuclear receptor coactivator 2 | NCOA2 | Q15596 |
| DS58 | | tanshinone Ⅵ | Nuclear receptor coactivator 1 | NCOA1 | Q15788 |
| DS58 | | tanshinone Ⅵ | Calmodulin | CALM1 | P0DP23 |
| J1 | | hederagenin | Progesterone receptor | PGR | P06401 |
| J1 | | hederagenin | Nuclear receptor coactivator 2 | NCOA2 | Q15596 |
| J1 | | hederagenin | Muscarinic acetylcholine receptor M3 | CHRM3 | P20309 |
| J1 | | hederagenin | Muscarinic acetylcholine receptor M1 | CHRM1 | P11229 |
| J1 | | hederagenin | Gamma-aminobutyric-acid receptor alpha-2 subunit | GABRA2 | P47869 |
| J1 | | hederagenin | Gamma-aminobutyric-acid receptor alpha-3 subunit | GABRA3 | P34903 |
| J1 | | hederagenin | Muscarinic acetylcholine receptor M2 | CHRM2 | P08172 |
| J1 | | hederagenin | Alpha-1B adrenergic receptor | ADRA1B | P35368 |
| J1 | | hederagenin | Gamma-aminobutyric acid receptor subunit alpha-1 | GABRA1 | P14867 |
| J1 | | hederagenin | Glutamate receptor 2 | GRIA2 | P42262 |
| J1 | | hederagenin | Gamma-aminobutyric-acid receptor subunit alpha-6 | GABRA6 | Q16445 |
| J1 | | hederagenin | Gamma-aminobutyric-acid receptor alpha-5 subunit | GABRA5 | P31644 |
| J1 | | hederagenin | Ig gamma-1 chain C region | IGHG1 | P01857 |
| J1 | | hederagenin | Alcohol dehydrogenase 1B | ADH1B | P00325 |
| J1 | | hederagenin | Alcohol dehydrogenase 1C | ADH1C | P00326 |
| J1 | | hederagenin | Prostaglandin G/H synthase 1 | PTGS1 | P23219 |
| J1 | | hederagenin | Sodium channel protein type 5 subunit alpha | SCN5A | Q14524 |
| J1 | | hederagenin | Prostaglandin G/H synthase 2 | PTGS2 | P35354 |
| J1 | | hederagenin | Retinoic acid receptor RXR-alpha | RXRA | P19793 |
| J1 | | hederagenin | CGMP-inhibited 3',5'-cyclic phosphodiesterase A | PDE3A | Q14432 |
| J1 | | hederagenin | Sodium-dependent noradrenaline transporter | SLC6A2 | P23975 |
| EZ1 | | bisdemethoxycurcumin | Beta-secretase 1 | BACE1 | P56817 |
| I1 | | Mandenol | Prostaglandin G/H synthase 1 | PTGS1 | P23219 |
| I1 | | Mandenol | Prostaglandin G/H synthase 2 | PTGS2 | P35354 |
| I1 | | Mandenol | Nuclear receptor coactivator 2 | NCOA2 | Q15596 |
| GL1 | | Diosmetin | Nitric oxide synthase, inducible | NOS2 | P35228 |
| GL1 | | Diosmetin | Prostaglandin G/H synthase 1 | PTGS1 | P23219 |
| GL1 | | Diosmetin | Prostaglandin G/H synthase 2 | PTGS2 | P35354 |
| GL1 | | Diosmetin | Dipeptidyl peptidase IV | DPP4 | P27487 |
| GL1 | | Diosmetin | Heat shock protein HSP 90 | HSP90AA1 | P07900 |
| GL1 | | Diosmetin | mRNA of PKA Catalytic Subunit C-alpha | PRKACA | P17612 |
| GL1 | | Diosmetin | Trypsin-1 | PRSS1 | P07477 |
| GL1 | | Diosmetin | Nuclear receptor coactivator 2 | NCOA2 | Q15596 |
| GL1 | | Diosmetin | Nuclear receptor coactivator 1 | NCOA1 | Q15788 |
| GL1 | | Diosmetin | Calmodulin | CALM1 | P0DP23 |
| GL2 | | Spinasterol | Progesterone receptor | PGR | P06401 |
| GL2 | | Spinasterol | Mineralocorticoid receptor | NR3C2 | P08235 |
| GL2 | | Spinasterol | Nuclear receptor coactivator 2 | NCOA2 | Q15596 |
| GL3 | | Hydroxygenkwanin | Nitric oxide synthase, inducible | NOS2 | P35228 |
| GL3 | | Hydroxygenkwanin | Prostaglandin G/H synthase 1 | PTGS1 | P23219 |
| GL3 | | Hydroxygenkwanin | Prostaglandin G/H synthase 2 | PTGS2 | P35354 |
| GL3 | | Hydroxygenkwanin | Dipeptidyl peptidase IV | DPP4 | P27487 |
| GL3 | | Hydroxygenkwanin | Heat shock protein HSP 90 | HSP90AA1 | P07900 |
| GL3 | | Hydroxygenkwanin | mRNA of PKA Catalytic Subunit C-alpha | PRKACA | P17612 |
| GL3 | | Hydroxygenkwanin | Trypsin-1 | PRSS1 | P07477 |
| GL3 | | Hydroxygenkwanin | Nuclear receptor coactivator 2 | NCOA2 | Q15596 |
| GL3 | | Hydroxygenkwanin | Calmodulin | CALM1 | P0DP23 |
| GL3 | | Hydroxygenkwanin | Phosphatidylinositol-4,5-bisphosphate 3-kinase catalytic subunit, gamma isoform | PIK3CG | P48736 |
| GL4 | | Schottenol | Progesterone receptor | PGR | P06401 |
| GL4 | | Schottenol | Nuclear receptor coactivator 2 | NCOA2 | Q15596 |
| GL5 | | 10α-cucurbita-5,24-diene-3β-ol | Progesterone receptor | PGR | P06401 |
| GL5 | | 10α-cucurbita-5,24-diene-3β-ol | Mineralocorticoid receptor | NR3C2 | P08235 |
| GL6 | | 5-dehydrokarounidiol | Glucocorticoid receptor | NR3C1 | P04150 |
| GL7 | | 7-oxo-dihydrokaro-unidiol | Mineralocorticoid receptor | NR3C2 | P08235 |
| GL8 | | Linolenic acid ethyl ester | Prostaglandin G/H synthase 1 | PTGS1 | P23219 |
| GL8 | | Linolenic acid ethyl ester | Prostaglandin G/H synthase 2 | PTGS2 | P35354 |
| GL9 | | vitamin-e | Coagulation factor Xa | F10 | P00742 |
| PFZ1 | | 11,14-eicosadienoic acid | Nuclear receptor coactivator 2 | NCOA2 | Q15596 |
| PFZ2 | | Delphin_qt | Prostaglandin G/H synthase 1 | PTGS1 | P23219 |
| PFZ2 | | Delphin_qt | Prostaglandin G/H synthase 2 | PTGS2 | P35354 |
| PFZ2 | | Delphin_qt | Carbonic anhydrase II | CA2 | P00918 |
| PFZ2 | | Delphin_qt | Heat shock protein HSP 90 | HSP90AA1 | P07900 |
| PFZ2 | | Delphin_qt | Phosphatidylinositol-4,5-bisphosphate 3-kinase catalytic subunit, gamma isoform | PIK3CG | P48736 |
| PFZ2 | | Delphin_qt | Nuclear receptor coactivator 2 | NCOA2 | Q15596 |
| PFZ3 | | Deltoin | Prostaglandin G/H synthase 1 | PTGS1 | P23219 |
| PFZ3 | | Deltoin | Thrombin | F2 | P00734 |
| PFZ3 | | Deltoin | Sodium channel protein type 5 subunit alpha | SCN5A | Q14524 |
| PFZ3 | | Deltoin | Coagulation factor Xa | F10 | P00742 |
| PFZ3 | | Deltoin | Prostaglandin G/H synthase 2 | PTGS2 | P35354 |
| PFZ3 | | Deltoin | Acetylcholinesterase | ACHE | P22303 |
| PFZ3 | | Deltoin | Alpha-1B adrenergic receptor | ADRA1B | P35368 |
| PFZ3 | | Deltoin | Beta-2 adrenergic receptor | ADRB2 | P07550 |
| PFZ3 | | Deltoin | Dipeptidyl peptidase IV | DPP4 | P27487 |
| PFZ3 | | Deltoin | Trypsin-1 | PRSS1 | P07477 |
| PFZ3 | | Deltoin | Calmodulin | CALM1 | P0DP23 |
| PFZ4 | | Deoxyandrographolide | Prostaglandin G/H synthase 2 | PTGS2 | P35354 |
| PFZ4 | | Deoxyandrographolide | Progesterone receptor | PGR | P06401 |
| PFZ4 | | Deoxyandrographolide | Nuclear receptor coactivator 2 | NCOA2 | Q15596 |
| PFZ4 | | Deoxyandrographolide | Nuclear receptor coactivator 1 | NCOA1 | Q15788 |
| PFZ5 | | Karanjin | Prostaglandin G/H synthase 1 | PTGS1 | P23219 |
| PFZ5 | | Karanjin | Estrogen receptor | ESR1 | P03372 |
| PFZ5 | | Karanjin | Prostaglandin G/H synthase 2 | PTGS2 | P35354 |
| PFZ5 | | Karanjin | Phosphatidylinositol-4,5-bisphosphate 3-kinase catalytic subunit, gamma isoform | PIK3CG | P48736 |
| PFZ5 | | Karanjin | Serine/threonine-protein kinase Chk1 | CHEK1 | O14757 |
| G1 | | sitosterol | Progesterone receptor | PGR | P06401 |
| G1 | | sitosterol | Nuclear receptor coactivator 2 | NCOA2 | Q15596 |
| G1 | | sitosterol | Mineralocorticoid receptor | NR3C2 | P08235 |
| PFZ6 | | (R)-Norcoclaurine | Dopamine D2 receptor | DRD2 | P14416 |
| PFZ6 | | (R)-Norcoclaurine | Dopamine D4 receptor | DRD4 | P21917 |
| E1 | | Mairin | Progesterone receptor | PGR | P06401 |
| HJS1 | | 3'-methyleriodictyol | Prostaglandin G/H synthase 1 | PTGS1 | P23219 |
| HJS1 | | 3'-methyleriodictyol | Prostaglandin G/H synthase 2 | PTGS2 | P35354 |
| HJS1 | | 3'-methyleriodictyol | Heat shock protein HSP 90 | HSP90AA1 | P07900 |
| HJS1 | | 3'-methyleriodictyol | mRNA of PKA Catalytic Subunit C-alpha | PRKACA | P17612 |
| HJS1 | | 3'-methyleriodictyol | Calmodulin | CALM1 | P0DP23 |
| HJS2 | | Rhamnazin | Nitric oxide synthase, inducible | NOS2 | P35228 |
| HJS2 | | Rhamnazin | Prostaglandin G/H synthase 1 | PTGS1 | P23219 |
| HJS2 | | Rhamnazin | Estrogen receptor | ESR1 | P03372 |
| HJS2 | | Rhamnazin | Androgen receptor | AR | P10275 |
| HJS2 | | Rhamnazin | Sodium channel protein type 5 subunit alpha | SCN5A | Q14524 |
| HJS2 | | Rhamnazin | Peroxisome proliferator activated receptor gamma | PPARG | P37231 |
| HJS2 | | Rhamnazin | Prostaglandin G/H synthase 2 | PTGS2 | P35354 |
| HJS2 | | Rhamnazin | Estrogen receptor beta | ESR2 | Q92731 |
| HJS2 | | Rhamnazin | Dipeptidyl peptidase IV | DPP4 | P27487 |
| HJS2 | | Rhamnazin | Mitogen-activated protein kinase 14 | MAPK14 | Q16539 |
| HJS2 | | Rhamnazin | Glycogen synthase kinase-3 beta | GSK3B | P49841 |
| HJS2 | | Rhamnazin | Heat shock protein HSP 90 | HSP90AA1 | P07900 |
| HJS2 | | Rhamnazin | Cell division protein kinase 2 | CDK2 | P24941 |
| HJS2 | | Rhamnazin | Trypsin-1 | PRSS1 | P07477 |
| HJS2 | | Rhamnazin | Proto-oncogene serine/threonine-protein kinase Pim-1 | PIM1 | P11309 |
| HJS2 | | Rhamnazin | Cyclin-A2 | CCNA2 | P20248 |
| HJS2 | | Rhamnazin | Nuclear receptor coactivator 2 | NCOA2 | Q15596 |
| HJS2 | | Rhamnazin | Calmodulin | CALM1 | P0DP23 |
| HJS2 | | Rhamnazin | Peroxisome proliferator activated receptor delta | PPARD | Q03181 |
| HJS2 | | Rhamnazin | Phosphatidylinositol-4,5-bisphosphate 3-kinase catalytic subunit, gamma isoform | PIK3CG | P48736 |
| HJS2 | | Rhamnazin | Coagulation factor Xa | F10 | P00742 |
| HJS2 | | Rhamnazin | DNA topoisomerase II | TOP2A | P11388 |
| HJS2 | | Rhamnazin | Serine/threonine-protein kinase Chk1 | CHEK1 | O14757 |
| F1 | | isorhamnetin | Nitric oxide synthase, inducible | NOS2 | P35228 |
| F1 | | isorhamnetin | Prostaglandin G/H synthase 1 | PTGS1 | P23219 |
| F1 | | isorhamnetin | Estrogen receptor | ESR1 | P03372 |
| F1 | | isorhamnetin | Androgen receptor | AR | P10275 |
| F1 | | isorhamnetin | Peroxisome proliferator activated receptor gamma | PPARG | P37231 |
| F1 | | isorhamnetin | Prostaglandin G/H synthase 2 | PTGS2 | P35354 |
| F1 | | isorhamnetin | mRNA of Protein-tyrosine phosphatase, non-receptor type 1 | PTPN1 | P18031 |
| F1 | | isorhamnetin | Estrogen receptor beta | ESR2 | Q92731 |
| F1 | | isorhamnetin | Dipeptidyl peptidase IV | DPP4 | P27487 |
| F1 | | isorhamnetin | Mitogen-activated protein kinase 14 | MAPK14 | Q16539 |
| F1 | | isorhamnetin | Glycogen synthase kinase-3 beta | GSK3B | P49841 |
| F1 | | isorhamnetin | Heat shock protein HSP 90 | HSP90AA1 | P07900 |
| F1 | | isorhamnetin | Cell division protein kinase 2 | CDK2 | P24941 |
| F1 | | isorhamnetin | Phosphatidylinositol-4,5-bisphosphate 3-kinase catalytic subunit, gamma isoform | PIK3CG | P48736 |
| F1 | | isorhamnetin | mRNA of PKA Catalytic Subunit C-alpha | PRKACA | P17612 |
| H1 | | beta-sitosterol | Progesterone receptor | PGR | P06401 |
| H1 | | beta-sitosterol | Nuclear receptor coactivator 2 | NCOA2 | Q15596 |
| H1 | | beta-sitosterol | Prostaglandin G/H synthase 1 | PTGS1 | P23219 |
| H1 | | beta-sitosterol | Prostaglandin G/H synthase 2 | PTGS2 | P35354 |
| H1 | | beta-sitosterol | Heat shock protein HSP 90 | HSP90AA1 | P07900 |
| H1 | | beta-sitosterol | Phosphatidylinositol-4,5-bisphosphate 3-kinase catalytic subunit, gamma isoform | PIK3CG | P48736 |
| H1 | | beta-sitosterol | Potassium voltage-gated channel subfamily H member 2 | KCNH2 | Q12809 |
| H1 | | beta-sitosterol | mRNA of PKA Catalytic Subunit C-alpha | PRKACA | P17612 |
| H1 | | beta-sitosterol | Dopamine D1 receptor | DRD1 | P21728 |
| H1 | | beta-sitosterol | Muscarinic acetylcholine receptor M3 | CHRM3 | P20309 |
| H1 | | beta-sitosterol | Muscarinic acetylcholine receptor M1 | CHRM1 | P11229 |
| H1 | | beta-sitosterol | Sodium channel protein type 5 subunit alpha | SCN5A | Q14524 |
| H1 | | beta-sitosterol | Gamma-aminobutyric-acid receptor alpha-2 subunit | GABRA2 | P47869 |
| H1 | | beta-sitosterol | Muscarinic acetylcholine receptor M4 | CHRM4 | P08173 |
| H1 | | beta-sitosterol | CGMP-inhibited 3',5'-cyclic phosphodiesterase A | PDE3A | Q14432 |
| H1 | | beta-sitosterol | 5-hydroxytryptamine 2A receptor | HTR2A | P28223 |
| H1 | | beta-sitosterol | Gamma-aminobutyric-acid receptor alpha-5 subunit | GABRA5 | P31644 |
| H1 | | beta-sitosterol | Alpha-1A adrenergic receptor | ADRA1A | P35348 |
| H1 | | beta-sitosterol | Gamma-aminobutyric-acid receptor alpha-3 subunit | GABRA3 | P34903 |
| H1 | | beta-sitosterol | Muscarinic acetylcholine receptor M2 | CHRM2 | P08172 |
| H1 | | beta-sitosterol | Alpha-1B adrenergic receptor | ADRA1B | P35368 |
| H1 | | beta-sitosterol | Beta-2 adrenergic receptor | ADRB2 | P07550 |
| H1 | | beta-sitosterol | Neuronal acetylcholine receptor subunit alpha-2 | CHRNA2 | Q15822 |
| H1 | | beta-sitosterol | Sodium-dependent serotonin transporter | SLC6A4 | P31645 |
| H1 | | beta-sitosterol | Mu-type opioid receptor | OPRM1 | P35372 |
| H1 | | beta-sitosterol | Gamma-aminobutyric acid receptor subunit alpha-1 | GABRA1 | P14867 |
| H1 | | beta-sitosterol | Neuronal acetylcholine receptor protein, alpha-7 chain | CHRNA7 | P36544 |
| H1 | | beta-sitosterol | Apoptosis regulator Bcl-2 | BCL2 | P10415 |
| H1 | | beta-sitosterol | Apoptosis regulator BAX | BAX | Q07812 |
| H1 | | beta-sitosterol | Caspase-9 | CASP9 | P55211 |
| H1 | | beta-sitosterol | Transcription factor AP-1 | JUN | P05412 |
| H1 | | beta-sitosterol | Caspase-3 | CASP3 | P42574 |
| H1 | | beta-sitosterol | Caspase-8 | CASP8 | Q14790 |
| H1 | | beta-sitosterol | Protein kinase C alpha type | PRKCA | P17252 |
| H1 | | beta-sitosterol | Transforming growth factor beta-1 | TGFB1 | P01137 |
| H1 | | beta-sitosterol | Serum paraoxonase/arylesterase 1 | PON1 | P27169 |
| H1 | | beta-sitosterol | Microtubule-associated protein 2 | MAP2 | P11137 |
| HQ1 | | (3S,8S,9S,10R,13R,14S,17R)-10,13-dimethyl-17-[(2R,5S)-5-propan-2-yloctan-2-yl]-2,3,4,7,8,9,11,12,14,15,16,17-dodecahydro-1H-cyclopenta[a]phenanthren-3-ol | Progesterone receptor | PGR | P06401 |
| D1 | | quercetin | 26S proteasome non-ATPase regulatory subunit 3 | PSMD3 | O43242 |
| D1 | | quercetin | 72 kDa type IV collagenase | MMP2 | P08253 |
| D1 | | quercetin | 78 kDa glucose-regulated protein | HSPA5 | P11021 |
| D1 | | quercetin | Acetylcholinesterase | ACHE | P22303 |
| D1 | | quercetin | Acetyl-CoA carboxylase 1 | ACACA | Q13085 |
| D1 | | quercetin | Activator of 90 kDa heat shock protein ATPase homolog 1 | AHSA1 | O95433 |
| D1 | | quercetin | Aldose reductase | AKR1B1 | P15121 |
| D1 | | quercetin | Amine oxidase [flavin-containing] B | MAOB | P27338 |
| D1 | | quercetin | Androgen receptor | AR | P10275 |
| D1 | | quercetin | Apoptosis regulator BAX | BAX | Q07812 |
| D1 | | quercetin | Apoptosis regulator Bcl-2 | BCL2 | P10415 |
| D1 | | quercetin | Arachidonate 5-lipoxygenase | ALOX5 | P09917 |
| D1 | | quercetin | Aryl hydrocarbon receptor | AHR | P35869 |
| D1 | | quercetin | ATP-binding cassette sub-family G member 2 | ABCG2 | Q9UNQ0 |
| D1 | | quercetin | Baculoviral IAP repeat-containing protein 5 | BIRC5 | O15392 |
| D1 | | quercetin | Bcl-2-like protein 1 | BCL2L1 | Q07817 |
| D1 | | quercetin | Beta-2 adrenergic receptor | ADRB2 | P07550 |
| D1 | | quercetin | Caspase-3 | CASP3 | P42574 |
| D1 | | quercetin | Caspase-8 | CASP8 | Q14790 |
| D1 | | quercetin | Caspase-9 | CASP9 | P55211 |
| D1 | | quercetin | Cathepsin D | CTSD | P07339 |
| D1 | | quercetin | Caveolin-1 | CAV1 | Q03135 |
| D1 | | quercetin | C-C motif chemokine 2 | CCL2 | P13500 |
| D1 | | quercetin | CD40 ligand | CD40LG | P29965 |
| D1 | | quercetin | Cellular tumor antigen p53 | TP53 | P04637 |
| D1 | | quercetin | Claudin-4 | CLDN4 | O14493 |
| D1 | | quercetin | Coagulation factor VII | F7 | P08709 |
| D1 | | quercetin | Coagulation factor Xa | F10 | P00742 |
| D1 | | quercetin | Collagen alpha-1(I) chain | COL1A1 | P02452 |
| D1 | | quercetin | Collagen alpha-1(III) chain | COL3A1 | P02461 |
| D1 | | quercetin | C-reactive protein | CRP | P02741 |
| D1 | | quercetin | C-X-C motif chemokine 10 | CXCL10 | P02778 |
| D1 | | quercetin | C-X-C motif chemokine 11 | CXCL11 | O14625 |
| D1 | | quercetin | C-X-C motif chemokine 2 | CXCL2 | P19875 |
| D1 | | quercetin | Cyclin-dependent kinase inhibitor 1 | CDKN1A | P38936 |
| D1 | | quercetin | Cyclin-dependent kinase inhibitor 2A, isoforms 1/2/3 | CDKN2A | Q8N726 |
| D1 | | quercetin | Cytochrome P450 1A1 | CYP1A1 | P04798 |
| D1 | | quercetin | Cytochrome P450 1A2 | CYP1A2 | P05177 |
| D1 | | quercetin | Cytochrome P450 1B1 | CYP1B1 | Q16678 |
| D1 | | quercetin | Cytochrome P450 3A4 | CYP3A4 | P08684 |
| D1 | | quercetin | DDB1- and CUL4-associated factor 5 | DCAF5 | Q96JK2 |
| D1 | | quercetin | Dipeptidyl peptidase IV | DPP4 | P27487 |
| D1 | | quercetin | DNA topoisomerase 1 | TOP1 | P11387 |
| D1 | | quercetin | DNA topoisomerase 2-alpha | TOP2A | P11388 |
| D1 | | quercetin | DNA topoisomerase II | TOP2A | P11388 |
| D1 | | quercetin | Dual oxidase 2 | DUOX2 | Q9NRD8 |
| D1 | | quercetin | Epidermal growth factor receptor | EGFR | P00533 |
| D1 | | quercetin | E-selectin | SELE | P16581 |
| D1 | | quercetin | Estrogen sulfotransferase | SULT1E1 | P49888 |
| D1 | | quercetin | ETS domain-containing protein Elk-1 | ELK1 | P19419 |
| D1 | | quercetin | Eukaryotic translation initiation factor 6 | EIF6 | P56537 |
| D1 | | quercetin | G1/S-specific cyclin-D1 | CCND1 | P24385 |
| D1 | | quercetin | G2/mitotic-specific cyclin-B1 | CCNB1 | P14635 |
| D1 | | quercetin | Gamma-aminobutyric acid receptor subunit alpha-1 | GABRA1 | P14867 |
| D1 | | quercetin | Gap junction alpha-1 protein | GJA1 | P17302 |
| D1 | | quercetin | Glutathione S-transferase Mu 1 | GSTM1 | P09488 |
| D1 | | quercetin | Glutathione S-transferase Mu 2 | GSTM2 | P28161 |
| D1 | | quercetin | Glutathione S-transferase P | GSTP1 | P09211 |
| D1 | | quercetin | Heat shock factor protein 1 | HSF1 | Q00613 |
| D1 | | quercetin | Heat shock protein beta-1 | HSPB1 | P04792 |
| D1 | | quercetin | Heat shock protein HSP 90 | HSP90AA1 | P07900 |
| D1 | | quercetin | Heme oxygenase 1 | HMOX1 | P09601 |
| D1 | | quercetin | Hexokinase-2 | HK2 | P52789 |
| D1 | | quercetin | Homeobox protein Nkx-3.1 | NKX3-1 | Q99801 |
| D1 | | quercetin | Hyaluronan synthase 2 | HAS2 | Q92819 |
| D1 | | quercetin | Hypoxia-inducible factor 1-alpha | HIF1A | Q16665 |
| D1 | | quercetin | Inhibitor of nuclear factor kappa-B kinase subunit alpha | CHUK | O15111 |
| D1 | | quercetin | Insulin receptor | INSR | P06213 |
| D1 | | quercetin | Insulin-like growth factor II | IGF2 | P01344 |
| D1 | | quercetin | Insulin-like growth factor-binding protein 3 | IGFBP3 | P17936 |
| D1 | | quercetin | Intercellular adhesion molecule 1 | ICAM1 | P05362 |
| D1 | | quercetin | Interferon gamma | IFNG | P01579 |
| D1 | | quercetin | Interferon regulatory factor 1 | IRF1 | P10914 |
| D1 | | quercetin | Interleukin-1 alpha | IL1A | P01583 |
| D1 | | quercetin | Interleukin-1 beta | IL1B | P01584 |
| D1 | | quercetin | Interleukin-10 | IL10 | P22301 |
| D1 | | quercetin | Interleukin-2 | IL2 | P60568 |
| D1 | | quercetin | Interleukin-6 | IL6 | P05231 |
| D1 | | quercetin | Interleukin-8 | CXCL8 | P10145 |
| D1 | | quercetin | Interstitial collagenase | MMP1 | P03956 |
| D1 | | quercetin | Maltase-glucoamylase, intestinal | MGAM | O43451 |
| D1 | | quercetin | Matrix metalloproteinase-9 | MMP9 | P14780 |
| D1 | | quercetin | Mitogen-activated protein kinase 1 | MAPK1 | P28482 |
| D1 | | quercetin | mRNA of PKA Catalytic Subunit C-alpha | PRKACA | P17612 |
| D1 | | quercetin | Myc proto-oncogene protein | MYC | P01106 |
| D1 | | quercetin | Myeloperoxidase | MPO | P05164 |
| D1 | | quercetin | NAD(P)H dehydrogenase [quinone] 1 | NQO1 | P15559 |
| D1 | | quercetin | NADPH--cytochrome P450 reductase | POR | P16435 |
| D1 | | quercetin | Neutrophil cytosol factor 1 | NCF1 | P14598 |
| D1 | | quercetin | NF-kappa-B inhibitor alpha | NFKBIA | P25963 |
| D1 | | quercetin | Nitric oxide synthase, endothelial | NOS3 | P29474 |
| D1 | | quercetin | Nitric-oxide synthase, endothelial | NOS3 | P29474 |
| D1 | | quercetin | Nuclear factor erythroid 2-related factor 2 | NFE2L2 | Q16236 |
| D1 | | quercetin | Nuclear receptor coactivator 2 | NCOA2 | Q15596 |
| D1 | | quercetin | Nuclear receptor subfamily 1 group I member 2 | NR1I2 | O75469 |
| D1 | | quercetin | Nuclear receptor subfamily 1 group I member 3 | NR1I3 | Q14994 |
| D1 | | quercetin | Ornithine decarboxylase | ODC1 | P11926 |
| D1 | | quercetin | Osteopontin | SPP1 | P10451 |
| D1 | | quercetin | Peroxisome proliferator activated receptor gamma | PPARG | P37231 |
| D1 | | quercetin | Peroxisome proliferator-activated receptor alpha | PPARA | Q07869 |
| D1 | | quercetin | Peroxisome proliferator-activated receptor delta | PPARD | Q03181 |
| D1 | | quercetin | Peroxisome proliferator-activated receptor gamma | PPARG | P37231 |
| D1 | | quercetin | Phosphatidylinositol-3,4,5-trisphosphate 3-phosphatase and dual-specificity protein phosphatase PTEN | PTEN | P60484 |
| D1 | | quercetin | Phosphatidylinositol-4,5-bisphosphate 3-kinase catalytic subunit, gamma isoform | PIK3CG | P48736 |
| D1 | | quercetin | Plasminogen activator inhibitor 1 | SERPINE1 | P05121 |
| D1 | | quercetin | Poly [ADP-ribose] polymerase 1 | PARP1 | P09874 |
| D1 | | quercetin | Potassium voltage-gated channel subfamily H member 2 | KCNH2 | Q12809 |
| D1 | | quercetin | Probable E3 ubiquitin-protein ligase HERC5 | HERC5 | Q9UII4 |
| D1 | | quercetin | Procollagen C-endopeptidase enhancer 1 | PCOLCE | Q15113 |
| D1 | | quercetin | Pro-epidermal growth factor | EGF | P01133 |
| D1 | | quercetin | Prostaglandin E2 receptor EP3 subtype | PTGER3 | P43115 |
| D1 | | quercetin | Prostaglandin G/H synthase 1 | PTGS1 | P23219 |
| D1 | | quercetin | Prostaglandin G/H synthase 2 | PTGS2 | P35354 |
| D1 | | quercetin | Prostatic acid phosphatase | ACP3 | P15309 |
| D1 | | quercetin | Protein CBFA2T1 | RUNX1T1 | Q06455 |
| D1 | | quercetin | Protein kinase C alpha type | PRKCA | P17252 |
| D1 | | quercetin | Protein kinase C beta type | PRKCB | P05771 |
| D1 | | quercetin | Proto-oncogene c-Fos | FOS | P01100 |
| D1 | | quercetin | Puromycin-sensitive aminopeptidase | NPEPPS | P55786 |
| D1 | | quercetin | RAC-alpha serine/threonine-protein kinase | AKT1 | P31749 |
| D1 | | quercetin | RAF proto-oncogene serine/threonine-protein kinase | RAF1 | P04049 |
| D1 | | quercetin | Ras association domain-containing protein 1 | RASSF1 | Q9NS23 |
| D1 | | quercetin | Ras GTPase-activating protein 1 | RASA1 | P20936 |
| D1 | | quercetin | Receptor tyrosine-protein kinase erbB-2 | ERBB2 | P04626 |
| D1 | | quercetin | Receptor tyrosine-protein kinase erbB-3 | ERBB3 | P21860 |
| D1 | | quercetin | Retinoblastoma-associated protein | RB1 | P06400 |
| D1 | | quercetin | Retinoic acid receptor RXR-alpha | RXRA | P19793 |
| D1 | | quercetin | Runt-related transcription factor 2 | RUNX2 | Q13950 |
| D1 | | quercetin | Serine/threonine-protein kinase Chk2 | CHEK2 | O96017 |
| D1 | | quercetin | Serum paraoxonase/arylesterase 1 | PON1 | P27169 |
| D1 | | quercetin | Signal transducer and activator of transcription 1-alpha/beta | STAT1 | P42224 |
| D1 | | quercetin | Sodium channel protein type 5 subunit alpha | SCN5A | Q14524 |
| D1 | | quercetin | Solute carrier family 2, facilitated glucose transporter member 4 | SLC2A4 | P14672 |
| D1 | | quercetin | Stromelysin-1 | MMP3 | P08254 |
| D1 | | quercetin | Superoxide dismutase [Cu-Zn] | SOD1 | P00441 |
| D1 | | quercetin | Thrombin | F2 | P00734 |
| D1 | | quercetin | Thrombomodulin | THBD | P07204 |
| D1 | | quercetin | Tissue factor | F3 | P13726 |
| D1 | | quercetin | Tissue-type plasminogen activator | PLAT | P00750 |
| D1 | | quercetin | Transcription factor AP-1 | JUN | P05412 |
| D1 | | quercetin | Transcription factor E2F1 | E2F1 | Q01094 |
| D1 | | quercetin | Transcription factor E2F2 | E2F2 | Q14209 |
| D1 | | quercetin | Transcription factor p65 | RELA | Q04206 |
| D1 | | quercetin | Transforming growth factor beta-1 | TGFB1 | P01137 |
| D1 | | quercetin | Trypsin-1 | PRSS1 | P07477 |
| D1 | | quercetin | Tumor necrosis factor | TNF | P01375 |
| D1 | | quercetin | Type I iodothyronine deiodinase | DIO1 | P49895 |
| D1 | | quercetin | Urokinase-type plasminogen activator | PLAU | P00749 |
| D1 | | quercetin | Vascular cell adhesion protein 1 | VCAM1 | P19320 |
| D1 | | quercetin | Vascular endothelial growth factor A | VEGFA | P15692 |
| D1 | | quercetin | Xanthine dehydrogenase/oxidase | XDH | P47989 |
| HQ2 | | Jaranol | Androgen receptor | AR | P10275 |
| HQ2 | | Jaranol | Calmodulin | CALM1 | P0DP23 |
| HQ2 | | Jaranol | Cell division protein kinase 2 | CDK2 | P24941 |
| HQ2 | | Jaranol | Dipeptidyl peptidase IV | DPP4 | P27487 |
| HQ2 | | Jaranol | Estrogen receptor beta | ESR2 | Q92731 |
| HQ2 | | Jaranol | Heat shock protein HSP 90 | HSP90AA1 | P07900 |
| HQ2 | | Jaranol | Nitric oxide synthase, inducible | NOS2 | P35228 |
| HQ2 | | Jaranol | Nuclear receptor coactivator 2 | NCOA2 | Q15596 |
| HQ2 | | Jaranol | Prostaglandin G/H synthase 1 | PTGS1 | P23219 |
| HQ2 | | Jaranol | Prostaglandin G/H synthase 2 | PTGS2 | P35354 |
| HQ2 | | Jaranol | Serine/threonine-protein kinase Chk1 | CHEK1 | O14757 |
| HQ2 | | Jaranol | Sodium channel protein type 5 subunit alpha | SCN5A | Q14524 |
| HQ2 | | Jaranol | Trypsin-1 | PRSS1 | P07477 |
| J1 | | hederagenin | Progesterone receptor | PGR | P06401 |
| F1 | | isorhamnetin | Acetylcholinesterase | ACHE | P22303 |
| F1 | | isorhamnetin | Aldose reductase | AKR1B1 | P15121 |
| F1 | | isorhamnetin | Amine oxidase [flavin-containing] B | MAOB | P27338 |
| F1 | | isorhamnetin | Calmodulin | CALM1 | P0DP23 |
| F1 | | isorhamnetin | Coagulation factor VII | F7 | P08709 |
| F1 | | isorhamnetin | Cyclin-A2 | CCNA2 | P20248 |
| F1 | | isorhamnetin | Gamma-aminobutyric acid receptor subunit alpha-1 | GABRA1 | P14867 |
| F1 | | isorhamnetin | Glutamate receptor 2 | GRIA2 | P42262 |
| F1 | | isorhamnetin | Glycogen phosphorylase, muscle form | PYGM | P11217 |
| F1 | | isorhamnetin | Neutrophil cytosol factor 1 | NCF1 | P14598 |
| F1 | | isorhamnetin | Nitric-oxide synthase, endothelial | NOS3 | P29474 |
| F1 | | isorhamnetin | Nuclear receptor coactivator 1 | NCOA1 | Q15788 |
| F1 | | isorhamnetin | Nuclear receptor coactivator 2 | NCOA2 | Q15596 |
| F1 | | isorhamnetin | Oxidized low-density lipoprotein receptor 1 | OLR1 | P78380 |
| F1 | | isorhamnetin | Peroxisome proliferator activated receptor delta | PPARD | Q03181 |
| F1 | | isorhamnetin | Proto-oncogene serine/threonine-protein kinase Pim-1 | PIM1 | P11309 |
| F1 | | isorhamnetin | Serine/threonine-protein kinase Chk1 | CHEK1 | O14757 |
| F1 | | isorhamnetin | Thrombin | F2 | P00734 |
| F1 | | isorhamnetin | Transcription factor p65 | RELA | Q04206 |
| F1 | | isorhamnetin | Trypsin-1 | PRSS1 | P07477 |
| F1 | | isorhamnetin | Xanthine dehydrogenase/oxidase | XDH | P47989 |
| HQ3 | | 3,9-di-O-methylnissolin | 5-hydroxytryptamine receptor 3A | HTR3A | P46098 |
| HQ3 | | 3,9-di-O-methylnissolin | Acetylcholinesterase | ACHE | P22303 |
| HQ3 | | 3,9-di-O-methylnissolin | Alpha-1B adrenergic receptor | ADRA1B | P35368 |
| HQ3 | | 3,9-di-O-methylnissolin | Alpha-1D adrenergic receptor | ADRA1D | P25100 |
| HQ3 | | 3,9-di-O-methylnissolin | Alpha-2C adrenergic receptor | ADRA2C | P18825 |
| HQ3 | | 3,9-di-O-methylnissolin | Beta-1 adrenergic receptor | ADRB1 | P08588 |
| HQ3 | | 3,9-di-O-methylnissolin | Beta-2 adrenergic receptor | ADRB2 | P07550 |
| HQ3 | | 3,9-di-O-methylnissolin | Calmodulin | CALM1 | P0DP23 |
| HQ3 | | 3,9-di-O-methylnissolin | CGMP-inhibited 3',5'-cyclic phosphodiesterase A | PDE3A | Q14432 |
| HQ3 | | 3,9-di-O-methylnissolin | Estrogen receptor | ESR1 | P03372 |
| HQ3 | | 3,9-di-O-methylnissolin | Gamma-aminobutyric acid receptor subunit alpha-1 | GABRA1 | P14867 |
| HQ3 | | 3,9-di-O-methylnissolin | Muscarinic acetylcholine receptor M1 | CHRM1 | P11229 |
| HQ3 | | 3,9-di-O-methylnissolin | Muscarinic acetylcholine receptor M3 | CHRM3 | P20309 |
| HQ3 | | 3,9-di-O-methylnissolin | Mu-type opioid receptor | OPRM1 | P35372 |
| HQ3 | | 3,9-di-O-methylnissolin | Nitric oxide synthase, inducible | NOS2 | P35228 |
| HQ3 | | 3,9-di-O-methylnissolin | Nitric-oxide synthase, endothelial | NOS3 | P29474 |
| HQ3 | | 3,9-di-O-methylnissolin | Nuclear receptor coactivator 2 | NCOA2 | Q15596 |
| HQ3 | | 3,9-di-O-methylnissolin | Prostaglandin G/H synthase 1 | PTGS1 | P23219 |
| HQ3 | | 3,9-di-O-methylnissolin | Prostaglandin G/H synthase 2 | PTGS2 | P35354 |
| HQ3 | | 3,9-di-O-methylnissolin | Retinoic acid receptor RXR-alpha | RXRA | P19793 |
| HQ3 | | 3,9-di-O-methylnissolin | Sodium channel protein type 5 subunit alpha | SCN5A | Q14524 |
| HQ3 | | 3,9-di-O-methylnissolin | Thrombin | F2 | P00734 |
| HQ3 | | 3,9-di-O-methylnissolin | Trypsin-1 | PRSS1 | P07477 |
| HQ4 | | 7-O-methylisomucronulatol | 5-hydroxytryptamine 2A receptor | HTR2A | P28223 |
| HQ4 | | 7-O-methylisomucronulatol | Alpha-1A adrenergic receptor | ADRA1A | P35348 |
| HQ4 | | 7-O-methylisomucronulatol | Alpha-1B adrenergic receptor | ADRA1B | P35368 |
| HQ4 | | 7-O-methylisomucronulatol | Alpha-1D adrenergic receptor | ADRA1D | P25100 |
| HQ4 | | 7-O-methylisomucronulatol | Alpha-2C adrenergic receptor | ADRA2C | P18825 |
| HQ4 | | 7-O-methylisomucronulatol | Androgen receptor | AR | P10275 |
| HQ4 | | 7-O-methylisomucronulatol | Beta-1 adrenergic receptor | ADRB1 | P08588 |
| HQ4 | | 7-O-methylisomucronulatol | Beta-2 adrenergic receptor | ADRB2 | P07550 |
| HQ4 | | 7-O-methylisomucronulatol | Calcium-activated potassium channel subunit alpha 1 | KCNMA1 | Q12791 |
| HQ4 | | 7-O-methylisomucronulatol | Calmodulin | CALM1 | P0DP23 |
| HQ4 | | 7-O-methylisomucronulatol | Cell division protein kinase 2 | CDK2 | P24941 |
| HQ4 | | 7-O-methylisomucronulatol | CGMP-inhibited 3',5'-cyclic phosphodiesterase A | PDE3A | Q14432 |
| HQ4 | | 7-O-methylisomucronulatol | Coagulation factor Xa | F10 | P00742 |
| HQ4 | | 7-O-methylisomucronulatol | Cyclin-A2 | CCNA2 | P20248 |
| HQ4 | | 7-O-methylisomucronulatol | Delta-type opioid receptor | OPRD1 | P41143 |
| HQ4 | | 7-O-methylisomucronulatol | Dipeptidyl peptidase IV | DPP4 | P27487 |
| HQ4 | | 7-O-methylisomucronulatol | Dopamine D1 receptor | DRD1 | P21728 |
| HQ4 | | 7-O-methylisomucronulatol | Estrogen receptor | ESR1 | P03372 |
| HQ4 | | 7-O-methylisomucronulatol | Estrogen receptor beta | ESR2 | Q92731 |
| HQ4 | | 7-O-methylisomucronulatol | Gamma-aminobutyric acid receptor subunit alpha-1 | GABRA1 | P14867 |
| HQ4 | | 7-O-methylisomucronulatol | Glycogen synthase kinase-3 beta | GSK3B | P49841 |
| HQ4 | | 7-O-methylisomucronulatol | Heat shock protein HSP 90 | HSP90AA1 | P07900 |
| HQ4 | | 7-O-methylisomucronulatol | Mitogen-activated protein kinase 14 | MAPK14 | Q16539 |
| HQ4 | | 7-O-methylisomucronulatol | mRNA of PKA Catalytic Subunit C-alpha | PRKACA | P17612 |
| HQ4 | | 7-O-methylisomucronulatol | Muscarinic acetylcholine receptor M1 | CHRM1 | P11229 |
| HQ4 | | 7-O-methylisomucronulatol | Muscarinic acetylcholine receptor M2 | CHRM2 | P08172 |
| HQ4 | | 7-O-methylisomucronulatol | Muscarinic acetylcholine receptor M3 | CHRM3 | P20309 |
| HQ4 | | 7-O-methylisomucronulatol | Muscarinic acetylcholine receptor M4 | CHRM4 | P08173 |
| HQ4 | | 7-O-methylisomucronulatol | Muscarinic acetylcholine receptor M5 | CHRM5 | P08912 |
| HQ4 | | 7-O-methylisomucronulatol | Nitric oxide synthase, inducible | NOS2 | P35228 |
| HQ4 | | 7-O-methylisomucronulatol | Nitric-oxide synthase, endothelial | NOS3 | P29474 |
| HQ4 | | 7-O-methylisomucronulatol | Nuclear receptor coactivator 2 | NCOA2 | Q15596 |
| HQ4 | | 7-O-methylisomucronulatol | Peroxisome proliferator activated receptor gamma | PPARG | P37231 |
| HQ4 | | 7-O-methylisomucronulatol | Potassium voltage-gated channel subfamily H member 2 | KCNH2 | Q12809 |
| HQ4 | | 7-O-methylisomucronulatol | Prostaglandin G/H synthase 1 | PTGS1 | P23219 |
| HQ4 | | 7-O-methylisomucronulatol | Prostaglandin G/H synthase 2 | PTGS2 | P35354 |
| HQ4 | | 7-O-methylisomucronulatol | Proto-oncogene serine/threonine-protein kinase Pim-1 | PIM1 | P11309 |
| HQ4 | | 7-O-methylisomucronulatol | Retinoic acid receptor RXR-alpha | RXRA | P19793 |
| HQ4 | | 7-O-methylisomucronulatol | Retinoic acid receptor RXR-beta | RXRB | P28702 |
| HQ4 | | 7-O-methylisomucronulatol | Serine/threonine-protein kinase Chk1 | CHEK1 | O14757 |
| HQ4 | | 7-O-methylisomucronulatol | Sodium channel protein type 5 subunit alpha | SCN5A | Q14524 |
| HQ4 | | 7-O-methylisomucronulatol | Sodium-dependent dopamine transporter | SLC6A3 | Q01959 |
| HQ4 | | 7-O-methylisomucronulatol | Sodium-dependent serotonin transporter | SLC6A4 | P31645 |
| HQ4 | | 7-O-methylisomucronulatol | Thrombin | F2 | P00734 |
| HQ4 | | 7-O-methylisomucronulatol | Trypsin-1 | PRSS1 | P07477 |
| HQ5 | | 9,10-dimethoxypterocarpan-3-O-β-D-glucoside | DNA topoisomerase II | TOP2A | P11388 |
| HQ5 | | 9,10-dimethoxypterocarpan-3-O-β-D-glucoside | Nuclear receptor coactivator 2 | NCOA2 | Q15596 |
| HQ5 | | 9,10-dimethoxypterocarpan-3-O-β-D-glucoside | Prostaglandin G/H synthase 2 | PTGS2 | P35354 |
| HQ6 | | (6aR,11aR)-9,10-dimethoxy-6a,11a-dihydro-6H-benzofurano[3,2-c]chromen-3-ol | 5-hydroxytryptamine receptor 3A | HTR3A | P46098 |
| HQ6 | | (6aR,11aR)-9,10-dimethoxy-6a,11a-dihydro-6H-benzofurano[3,2-c]chromen-3-ol | Acetylcholinesterase | ACHE | P22303 |
| HQ6 | | (6aR,11aR)-9,10-dimethoxy-6a,11a-dihydro-6H-benzofurano[3,2-c]chromen-3-ol | Alpha-1B adrenergic receptor | ADRA1B | P35368 |
| HQ6 | | (6aR,11aR)-9,10-dimethoxy-6a,11a-dihydro-6H-benzofurano[3,2-c]chromen-3-ol | Alpha-1D adrenergic receptor | ADRA1D | P25100 |
| HQ6 | | (6aR,11aR)-9,10-dimethoxy-6a,11a-dihydro-6H-benzofurano[3,2-c]chromen-3-ol | Beta-2 adrenergic receptor | ADRB2 | P07550 |
| HQ6 | | (6aR,11aR)-9,10-dimethoxy-6a,11a-dihydro-6H-benzofurano[3,2-c]chromen-3-ol | Calmodulin | CALM1 | P0DP23 |
| HQ6 | | (6aR,11aR)-9,10-dimethoxy-6a,11a-dihydro-6H-benzofurano[3,2-c]chromen-3-ol | Estrogen receptor | ESR1 | P03372 |
| HQ6 | | (6aR,11aR)-9,10-dimethoxy-6a,11a-dihydro-6H-benzofurano[3,2-c]chromen-3-ol | Gamma-aminobutyric acid receptor subunit alpha-1 | GABRA1 | P14867 |
| HQ6 | | (6aR,11aR)-9,10-dimethoxy-6a,11a-dihydro-6H-benzofurano[3,2-c]chromen-3-ol | Heat shock protein HSP 90 | HSP90AA1 | P07900 |
| HQ6 | | (6aR,11aR)-9,10-dimethoxy-6a,11a-dihydro-6H-benzofurano[3,2-c]chromen-3-ol | Muscarinic acetylcholine receptor M1 | CHRM1 | P11229 |
| HQ6 | | (6aR,11aR)-9,10-dimethoxy-6a,11a-dihydro-6H-benzofurano[3,2-c]chromen-3-ol | Muscarinic acetylcholine receptor M3 | CHRM3 | P20309 |
| HQ6 | | (6aR,11aR)-9,10-dimethoxy-6a,11a-dihydro-6H-benzofurano[3,2-c]chromen-3-ol | Muscarinic acetylcholine receptor M4 | CHRM4 | P08173 |
| HQ6 | | (6aR,11aR)-9,10-dimethoxy-6a,11a-dihydro-6H-benzofurano[3,2-c]chromen-3-ol | Neuronal acetylcholine receptor protein, alpha-7 chain | CHRNA7 | P36544 |
| HQ6 | | (6aR,11aR)-9,10-dimethoxy-6a,11a-dihydro-6H-benzofurano[3,2-c]chromen-3-ol | Nitric oxide synthase, inducible | NOS2 | P35228 |
| HQ6 | | (6aR,11aR)-9,10-dimethoxy-6a,11a-dihydro-6H-benzofurano[3,2-c]chromen-3-ol | Nuclear receptor coactivator 1 | NCOA1 | Q15788 |
| HQ6 | | (6aR,11aR)-9,10-dimethoxy-6a,11a-dihydro-6H-benzofurano[3,2-c]chromen-3-ol | Nuclear receptor coactivator 2 | NCOA2 | Q15596 |
| HQ6 | | (6aR,11aR)-9,10-dimethoxy-6a,11a-dihydro-6H-benzofurano[3,2-c]chromen-3-ol | Prostaglandin G/H synthase 1 | PTGS1 | P23219 |
| HQ6 | | (6aR,11aR)-9,10-dimethoxy-6a,11a-dihydro-6H-benzofurano[3,2-c]chromen-3-ol | Prostaglandin G/H synthase 2 | PTGS2 | P35354 |
| HQ6 | | (6aR,11aR)-9,10-dimethoxy-6a,11a-dihydro-6H-benzofurano[3,2-c]chromen-3-ol | Retinoic acid receptor RXR-alpha | RXRA | P19793 |
| HQ6 | | (6aR,11aR)-9,10-dimethoxy-6a,11a-dihydro-6H-benzofurano[3,2-c]chromen-3-ol | Sodium channel protein type 5 subunit alpha | SCN5A | Q14524 |
| HQ6 | | (6aR,11aR)-9,10-dimethoxy-6a,11a-dihydro-6H-benzofurano[3,2-c]chromen-3-ol | Thrombin | F2 | P00734 |
| HQ6 | | (6aR,11aR)-9,10-dimethoxy-6a,11a-dihydro-6H-benzofurano[3,2-c]chromen-3-ol | Trypsin-1 | PRSS1 | P07477 |
| HQ7 | | Bifendate | Calcium-activated potassium channel subunit alpha 1 | KCNMA1 | Q12791 |
| HQ7 | | Bifendate | DNA topoisomerase II | TOP2A | P11388 |
| HQ7 | | Bifendate | Heat shock protein HSP 90 | HSP90AA1 | P07900 |
| HQ7 | | Bifendate | Hepatocyte growth factor receptor | MET | P08581 |
| HQ7 | | Bifendate | Prostaglandin G/H synthase 1 | PTGS1 | P23219 |
| HQ7 | | Bifendate | Prostaglandin G/H synthase 2 | PTGS2 | P35354 |
| HQ7 | | Bifendate | Vascular endothelial growth factor receptor 2 | KDR | P35968 |
| HQ8 | | formononetin | 3 beta-hydroxysteroid dehydrogenase/Delta 5-->4-isomerase type 1 | HSD3B1 | P14060 |
| HQ8 | | formononetin | 3 beta-hydroxysteroid dehydrogenase/Delta 5-->4-isomerase type 2 | HSD3B2 | P26439 |
| HQ8 | | formononetin | Acetylcholinesterase | ACHE | P22303 |
| HQ8 | | formononetin | Alpha-1A adrenergic receptor | ADRA1A | P35348 |
| HQ8 | | formononetin | Amine oxidase [flavin-containing] B | MAOB | P27338 |
| HQ8 | | formononetin | Androgen receptor | AR | P10275 |
| HQ8 | | formononetin | ATP synthase subunit beta, mitochondrial | ATP5F1B | P06576 |
| HQ8 | | formononetin | Beta-2 adrenergic receptor | ADRB2 | P07550 |
| HQ8 | | formononetin | Beta-lactamase | DPEP1 | P16444 |
| HQ8 | | formononetin | Calmodulin | CALM1 | P0DP23 |
| HQ8 | | formononetin | cAMP-dependent protein kinase inhibitor alpha | PKIA | P61925 |
| HQ8 | | formononetin | Cell division protein kinase 2 | CDK2 | P24941 |
| HQ8 | | formononetin | CGMP-inhibited 3',5'-cyclic phosphodiesterase A | PDE3A | Q14432 |
| HQ8 | | formononetin | Cyclin-A2 | CCNA2 | P20248 |
| HQ8 | | formononetin | Dipeptidyl peptidase IV | DPP4 | P27487 |
| HQ8 | | formononetin | Estrogen receptor | ESR1 | P03372 |
| HQ8 | | formononetin | Estrogen receptor beta | ESR2 | Q92731 |
| HQ8 | | formononetin | Glycogen synthase kinase-3 beta | GSK3B | P49841 |
| HQ8 | | formononetin | Heat shock protein HSP 90 | HSP90AA1 | P07900 |
| HQ8 | | formononetin | Interleukin-4 | IL4 | P05112 |
| HQ8 | | formononetin | Mitogen-activated protein kinase 14 | MAPK14 | Q16539 |
| HQ8 | | formononetin | mRNA of PKA Catalytic Subunit C-alpha | PRKACA | P17612 |
| HQ8 | | formononetin | Muscarinic acetylcholine receptor M1 | CHRM1 | P11229 |
| HQ8 | | formononetin | NAD-dependent deacetylase sirtuin-1 | SIRT1 | Q96EB6 |
| HQ8 | | formononetin | NADH-ubiquinone oxidoreductase chain 6 | MT-ND6 | P03923 |
| HQ8 | | formononetin | Nitric oxide synthase, inducible | NOS2 | P35228 |
| HQ8 | | formononetin | Nitric-oxide synthase, endothelial | NOS3 | P29474 |
| HQ8 | | formononetin | Peroxisome proliferator activated receptor gamma | PPARG | P37231 |
| HQ8 | | formononetin | Peroxisome proliferator-activated receptor gamma | PPARG | P37231 |
| HQ8 | | formononetin | Prostaglandin G/H synthase 1 | PTGS1 | P23219 |
| HQ8 | | formononetin | Prostaglandin G/H synthase 2 | PTGS2 | P35354 |
| HQ8 | | formononetin | Proto-oncogene serine/threonine-protein kinase Pim-1 | PIM1 | P11309 |
| HQ8 | | formononetin | Retinoic acid receptor RXR-alpha | RXRA | P19793 |
| HQ8 | | formononetin | Serine/threonine-protein kinase Chk1 | CHEK1 | O14757 |
| HQ8 | | formononetin | Sodium-dependent dopamine transporter | SLC6A3 | Q01959 |
| HQ8 | | formononetin | Sodium-dependent serotonin transporter | SLC6A4 | P31645 |
| HQ8 | | formononetin | Thrombin | F2 | P00734 |
| HQ8 | | formononetin | Transcription factor AP-1 | JUN | P05412 |
| HQ8 | | formononetin | Trypsin-1 | PRSS1 | P07477 |
| HQ9 | | Calycosin | Androgen receptor | AR | P10275 |
| HQ9 | | Calycosin | Beta-2 adrenergic receptor | ADRB2 | P07550 |
| HQ9 | | Calycosin | Calmodulin | CALM1 | P0DP23 |
| HQ9 | | Calycosin | Cell division protein kinase 2 | CDK2 | P24941 |
| HQ9 | | Calycosin | CGMP-inhibited 3',5'-cyclic phosphodiesterase A | PDE3A | Q14432 |
| HQ9 | | Calycosin | Cyclin-A2 | CCNA2 | P20248 |
| HQ9 | | Calycosin | Dipeptidyl peptidase IV | DPP4 | P27487 |
| HQ9 | | Calycosin | Estrogen receptor | ESR1 | P03372 |
| HQ9 | | Calycosin | Estrogen receptor beta | ESR2 | Q92731 |
| HQ9 | | Calycosin | Glycogen synthase kinase-3 beta | GSK3B | P49841 |
| HQ9 | | Calycosin | Heat shock protein HSP 90 | HSP90AA1 | P07900 |
| HQ9 | | Calycosin | Mitogen-activated protein kinase 14 | MAPK14 | Q16539 |
| HQ9 | | Calycosin | mRNA of PKA Catalytic Subunit C-alpha | PRKACA | P17612 |
| HQ9 | | Calycosin | Nitric oxide synthase, inducible | NOS2 | P35228 |
| HQ9 | | Calycosin | Nuclear receptor coactivator 2 | NCOA2 | Q15596 |
| HQ9 | | Calycosin | Peroxisome proliferator activated receptor gamma | PPARG | P37231 |
| HQ9 | | Calycosin | Prostaglandin G/H synthase 1 | PTGS1 | P23219 |
| HQ9 | | Calycosin | Prostaglandin G/H synthase 2 | PTGS2 | P35354 |
| HQ9 | | Calycosin | Proto-oncogene serine/threonine-protein kinase Pim-1 | PIM1 | P11309 |
| HQ9 | | Calycosin | Retinoic acid receptor RXR-alpha | RXRA | P19793 |
| HQ9 | | Calycosin | Serine/threonine-protein kinase Chk1 | CHEK1 | O14757 |
| HQ9 | | Calycosin | Trypsin-1 | PRSS1 | P07477 |
| C1 | | kaempferol | 26S proteasome non-ATPase regulatory subunit 3 | PSMD3 | O43242 |
| C1 | | kaempferol | Acetylcholinesterase | ACHE | P22303 |
| C1 | | kaempferol | Activator of 90 kDa heat shock protein ATPase homolog 1 | AHSA1 | O95433 |
| C1 | | kaempferol | Aldo-keto reductase family 1 member C3 | AKR1C3 | P42330 |
| C1 | | kaempferol | Alpha-1B adrenergic receptor | ADRA1B | P35368 |
| C1 | | kaempferol | Androgen receptor | AR | P10275 |
| C1 | | kaempferol | Antileukoproteinase | SLPI | P03973 |
| C1 | | kaempferol | Apoptosis regulator BAX | BAX | Q07812 |
| C1 | | kaempferol | Apoptosis regulator Bcl-2 | BCL2 | P10415 |
| C1 | | kaempferol | Arachidonate 5-lipoxygenase | ALOX5 | P09917 |
| C1 | | kaempferol | Aryl hydrocarbon receptor | AHR | P35869 |
| C1 | | kaempferol | Calmodulin | CALM1 | P0DP23 |
| C1 | | kaempferol | Caspase-3 | CASP3 | P42574 |
| C1 | | kaempferol | Coagulation factor VII | F7 | P08709 |
| C1 | | kaempferol | Cytochrome P450 1A1 | CYP1A1 | P04798 |
| C1 | | kaempferol | Cytochrome P450 1A2 | CYP1A2 | P05177 |
| C1 | | kaempferol | Cytochrome P450 1B1 | CYP1B1 | Q16678 |
| C1 | | kaempferol | Cytochrome P450 3A4 | CYP3A4 | P08684 |
| C1 | | kaempferol | Dipeptidyl peptidase IV | DPP4 | P27487 |
| C1 | | kaempferol | DNA topoisomerase II | TOP2A | P11388 |
| C1 | | kaempferol | E-selectin | SELE | P16581 |
| C1 | | kaempferol | Gamma-aminobutyric acid receptor subunit alpha-1 | GABRA1 | P14867 |
| C1 | | kaempferol | Gamma-aminobutyric-acid receptor alpha-2 subunit | GABRA2 | P47869 |
| C1 | | kaempferol | Glutathione S-transferase Mu 1 | GSTM1 | P09488 |
| C1 | | kaempferol | Glutathione S-transferase Mu 2 | GSTM2 | P28161 |
| C1 | | kaempferol | Glutathione S-transferase P | GSTP1 | P09211 |
| C1 | | kaempferol | Heat shock protein HSP 90 | HSP90AA1 | P07900 |
| C1 | | kaempferol | Heme oxygenase 1 | HMOX1 | P09601 |
| C1 | | kaempferol | Hyaluronan synthase 2 | HAS2 | Q92819 |
| C1 | | kaempferol | Inhibitor of nuclear factor kappa-B kinase subunit beta | IKBKB | O14920 |
| C1 | | kaempferol | Insulin receptor | INSR | P06213 |
| C1 | | kaempferol | Intercellular adhesion molecule 1 | ICAM1 | P05362 |
| C1 | | kaempferol | Interstitial collagenase | MMP1 | P03956 |
| C1 | | kaempferol | Mitogen-activated protein kinase 8 | MAPK8 | P45983 |
| C1 | | kaempferol | mRNA of PKA Catalytic Subunit C-alpha | PRKACA | P17612 |
| C1 | | kaempferol | Muscarinic acetylcholine receptor M1 | CHRM1 | P11229 |
| C1 | | kaempferol | Muscarinic acetylcholine receptor M2 | CHRM2 | P08172 |
| C1 | | kaempferol | Nitric oxide synthase, inducible | NOS2 | P35228 |
| C1 | | kaempferol | Nitric-oxide synthase, endothelial | NOS3 | P29474 |
| C1 | | kaempferol | Nuclear receptor coactivator 2 | NCOA2 | Q15596 |
| C1 | | kaempferol | Nuclear receptor subfamily 1 group I member 2 | NR1I2 | O75469 |
| C1 | | kaempferol | Nuclear receptor subfamily 1 group I member 3 | NR1I3 | Q14994 |
| C1 | | kaempferol | Peroxisome proliferator activated receptor gamma | PPARG | P37231 |
| C1 | | kaempferol | Peroxisome proliferator-activated receptor gamma | PPARG | P37231 |
| C1 | | kaempferol | Phosphatidylinositol-4,5-bisphosphate 3-kinase catalytic subunit, gamma isoform | PIK3CG | P48736 |
| C1 | | kaempferol | Progesterone receptor | PGR | P06401 |
| C1 | | kaempferol | Prostaglandin G/H synthase 1 | PTGS1 | P23219 |
| C1 | | kaempferol | Prostaglandin G/H synthase 2 | PTGS2 | P35354 |
| C1 | | kaempferol | RAC-alpha serine/threonine-protein kinase | AKT1 | P31749 |
| C1 | | kaempferol | Serine/threonine-protein phosphatase 2B catalytic subunit alpha isoform | PPP3CA | Q08209 |
| C1 | | kaempferol | Signal transducer and activator of transcription 1-alpha/beta | STAT1 | P42224 |
| C1 | | kaempferol | Sodium-dependent noradrenaline transporter | SLC6A2 | P23975 |
| C1 | | kaempferol | Solute carrier family 2, facilitated glucose transporter member 4 | SLC2A4 | P14672 |
| C1 | | kaempferol | Thrombin | F2 | P00734 |
| C1 | | kaempferol | Transcription factor AP-1 | JUN | P05412 |
| C1 | | kaempferol | Transcription factor p65 | RELA | Q04206 |
| C1 | | kaempferol | Trypsin-1 | PRSS1 | P07477 |
| C1 | | kaempferol | Tumor necrosis factor | TNF | P01375 |
| C1 | | kaempferol | Type I iodothyronine deiodinase | DIO1 | P49895 |
| C1 | | kaempferol | Vascular cell adhesion protein 1 | VCAM1 | P19320 |
| C1 | | kaempferol | Xanthine dehydrogenase/oxidase | XDH | P47989 |
| HQ10 | | FA | Cell division protein kinase 2 | CDK2 | P24941 |
| HQ10 | | FA | Glycogen synthase kinase-3 beta | GSK3B | P49841 |
| HQ10 | | FA | Thrombin | F2 | P00734 |
| HQ11 | | isomucronulatol-7,2'-di-O-glucosiole | DNA topoisomerase II | TOP2A | P11388 |
| HQ12 | | 1,7-Dihydroxy-3,9-dimethoxy pterocarpene | Heat shock protein HSP 90 | HSP90AA1 | P07900 |
| HQ12 | | 1,7-Dihydroxy-3,9-dimethoxy pterocarpene | Prostaglandin G/H synthase 2 | PTGS2 | P35354 |
| HQ12 | | 1,7-Dihydroxy-3,9-dimethoxy pterocarpene | Retinoic acid receptor RXR-alpha | RXRA | P19793 |
| HQ12 | | 1,7-Dihydroxy-3,9-dimethoxy pterocarpene | Trypsin-1 | PRSS1 | P07477 |
| JQC1 | | acacetin | Nitric oxide synthase, inducible | NOS2 | P35228 |
| JQC1 | | acacetin | Prostaglandin G/H synthase 1 | PTGS1 | P23219 |
| JQC1 | | acacetin | Androgen receptor | AR | P10275 |
| JQC1 | | acacetin | Prostaglandin G/H synthase 2 | PTGS2 | P35354 |
| JQC1 | | acacetin | Dipeptidyl peptidase IV | DPP4 | P27487 |
| JQC1 | | acacetin | Heat shock protein HSP 90 | HSP90AA1 | P07900 |
| JQC1 | | acacetin | Cell division protein kinase 2 | CDK2 | P24941 |
| JQC1 | | acacetin | mRNA of PKA Catalytic Subunit C-alpha | PRKACA | P17612 |
| JQC1 | | acacetin | Trypsin-1 | PRSS1 | P07477 |
| JQC1 | | acacetin | Nuclear receptor coactivator 2 | NCOA2 | Q15596 |
| JQC1 | | acacetin | Nuclear receptor coactivator 1 | NCOA1 | Q15788 |
| JQC1 | | acacetin | Calmodulin | CALM1 | P0DP23 |
| JQC1 | | acacetin | Phosphatidylinositol-4,5-bisphosphate 3-kinase catalytic subunit, gamma isoform | PIK3CG | P48736 |
| JQC1 | | acacetin | Serine/threonine-protein kinase Chk1 | CHEK1 | O14757 |
| JQC1 | | acacetin | Beta-2 adrenergic receptor | ADRB2 | P07550 |
| JQC1 | | acacetin | CGMP-inhibited 3',5'-cyclic phosphodiesterase A | PDE3A | Q14432 |
| JQC1 | | acacetin | Transcription factor p65 | RELA | Q04206 |
| JQC1 | | acacetin | Apoptosis regulator Bcl-2 | BCL2 | P10415 |
| JQC1 | | acacetin | Cyclin-dependent kinase inhibitor 1 | CDKN1A | P38936 |
| JQC1 | | acacetin | Apoptosis regulator BAX | BAX | Q07812 |
| JQC1 | | acacetin | Caspase-3 | CASP3 | P42574 |
| JQC1 | | acacetin | Cellular tumor antigen p53 | TP53 | P04637 |
| JQC1 | | acacetin | Caspase-8 | CASP8 | Q14790 |
| JQC1 | | acacetin | Fatty acid synthase | FASN | P49327 |
| JQC1 | | acacetin | Tumor necrosis factor ligand superfamily member 6 | FASLG | P48023 |
| JQC1 | | acacetin | Cytochrome P450 19A1 | CYP19A1 | P11511 |
| JQC2 | | Linarin | DNA topoisomerase II | TOP2A | P11388 |
| JQC3 | | Hesperetin | Prostaglandin G/H synthase 1 | PTGS1 | P23219 |
| JQC3 | | Hesperetin | Sodium channel protein type 5 subunit alpha | SCN5A | Q14524 |
| JQC3 | | Hesperetin | Prostaglandin G/H synthase 2 | PTGS2 | P35354 |
| JQC3 | | Hesperetin | Heat shock protein HSP 90 | HSP90AA1 | P07900 |
| JQC3 | | Hesperetin | Phosphatidylinositol-4,5-bisphosphate 3-kinase catalytic subunit, gamma isoform | PIK3CG | P48736 |
| JQC3 | | Hesperetin | mRNA of PKA Catalytic Subunit C-alpha | PRKACA | P17612 |
| JQC3 | | Hesperetin | Nuclear receptor coactivator 2 | NCOA2 | Q15596 |
| JQC3 | | Hesperetin | Nuclear receptor coactivator 1 | NCOA1 | Q15788 |
| JQC3 | | Hesperetin | Calmodulin | CALM1 | P0DP23 |
| JQC4 | | daucostero_qt | Progesterone receptor | PGR | P06401 |
| JQC4 | | daucostero_qt | Nuclear receptor coactivator 2 | NCOA2 | Q15596 |
| JQC5 | | ent-Epicatechin | Prostaglandin G/H synthase 1 | PTGS1 | P23219 |
| JQC5 | | ent-Epicatechin | Estrogen receptor | ESR1 | P03372 |
| JQC5 | | ent-Epicatechin | Prostaglandin G/H synthase 2 | PTGS2 | P35354 |
| JQC5 | | ent-Epicatechin | Heat shock protein HSP 90 | HSP90AA1 | P07900 |
| JQC5 | | ent-Epicatechin | Beta-lactamase | DPEP1 | P16444 |
| JQC5 | | ent-Epicatechin | mRNA of PKA Catalytic Subunit C-alpha | PRKACA | P17612 |
| JQC6 | | rhamnocitrin-3,4'-diglucOside | DNA topoisomerase II | TOP2A | P11388 |
| KWYXZ1 | | astragalin | Coagulation factor Xa | F10 | P00742 |
| KWYXZ1 | | astragalin | Prostaglandin G/H synthase 2 | PTGS2 | P35354 |
| KWYXZ1 | | astragalin | DNA topoisomerase II | TOP2A | P11388 |
| KWYXZ1 | | astragalin | Trypsin-1 | PRSS1 | P07477 |
| KWYXZ1 | | astragalin | Nitric oxide synthase, inducible | NOS2 | P35228 |
| KWYXZ1 | | astragalin | Prostaglandin G/H synthase 1 | PTGS1 | P23219 |
| KWYXZ1 | | astragalin | Heat shock protein HSP 90 | HSP90AA1 | P07900 |
| KWYXZ1 | | astragalin | Phosphatidylinositol-4,5-bisphosphate 3-kinase catalytic subunit, gamma isoform | PIK3CG | P48736 |
| KWYXZ1 | | astragalin | mRNA of PKA Catalytic Subunit C-alpha | PRKACA | P17612 |
| KWYXZ1 | | astragalin | Nuclear receptor coactivator 2 | NCOA2 | Q15596 |
| KWYXZ1 | | astragalin | Thrombin | F2 | P00734 |
| KWYXZ1 | | astragalin | Potassium voltage-gated channel subfamily H member 2 | KCNH2 | Q12809 |
| KWYXZ1 | | astragalin | Coagulation factor VII | F7 | P08709 |
| KWYXZ1 | | astragalin | Vascular endothelial growth factor receptor 2 | KDR | P35968 |
| KWYXZ1 | | astragalin | Dipeptidyl peptidase IV | DPP4 | P27487 |
| KWYXZ1 | | astragalin | Calmodulin | CALM1 | P0DP23 |
| KWYXZ2 | | rutin | DNA topoisomerase II | TOP2A | P11388 |
| KWYXZ2 | | rutin | Transcription factor p65 | RELA | Q04206 |
| KWYXZ2 | | rutin | Tumor necrosis factor | TNF | P01375 |
| KWYXZ2 | | rutin | Interleukin-6 | IL6 | P05231 |
| KWYXZ2 | | rutin | Caspase-3 | CASP3 | P42574 |
| KWYXZ2 | | rutin | NADPH--cytochrome P450 reductase | POR | P16435 |
| KWYXZ2 | | rutin | Superoxide dismutase [Cu-Zn] | SOD1 | P00441 |
| KWYXZ2 | | rutin | Catalase | CAT | P04040 |
| KWYXZ2 | | rutin | Interleukin-1 beta | IL1B | P01584 |
| KWYXZ2 | | rutin | Interleukin-8 | CXCL8 | P10145 |
| KWYXZ2 | | rutin | Protein kinase C beta type | PRKCB | P05771 |
| KWYXZ2 | | rutin | Arachidonate 5-lipoxygenase | ALOX5 | P09917 |
| KWYXZ2 | | rutin | 3-hydroxy-3-methylglutaryl-coenzyme A reductase | HMGCR | P04035 |
| KWYXZ2 | | rutin | Hyaluronan synthase 2 | HAS2 | Q92819 |
| KWYXZ2 | | rutin | Glutathione S-transferase P | GSTP1 | P09211 |
| KWYXZ2 | | rutin | Type I iodothyronine deiodinase | DIO1 | P49895 |
| KWYXZ2 | | rutin | C5a anaphylatoxin chemotactic receptor | C5AR1 | P21730 |
| KWYXZ2 | | rutin | Insulin | INS | P01308 |
| KWYXZ2 | | rutin | Low affinity immunoglobulin epsilon Fc receptor | FCER2 | P06734 |
| KWYXZ2 | | rutin | Integrin beta-2 | ITGB2 | P05107 |
| KWYXZ2 | | rutin | Thromboxane A2 receptor | TBXA2R | P21731 |
| KWYXZ3 | | quercitrin | Coagulation factor Xa | F10 | P00742 |
| KWYXZ3 | | quercitrin | mRNA of Protein-tyrosine phosphatase, non-receptor type 1 | PTPN1 | P18031 |
| KWYXZ3 | | quercitrin | DNA topoisomerase II | TOP2A | P11388 |
| KWYXZ3 | | quercitrin | Prostaglandin G/H synthase 2 | PTGS2 | P35354 |
| KWYXZ3 | | quercitrin | Trypsin-1 | PRSS1 | P07477 |
| KWYXZ3 | | quercitrin | Cytochrome P450 3A4 | CYP3A4 | P08684 |
| KWYXZ3 | | quercitrin | Hyaluronan synthase 2 | HAS2 | Q92819 |
| KWYXZ3 | | quercitrin | Glutathione S-transferase P | GSTP1 | P09211 |
| KWYXZ3 | | quercitrin | Aldose reductase | AKR1B1 | P15121 |
| KWYXZ4 | | ellagic acid | Cell division protein kinase 2 | CDK2 | P24941 |
| KWYXZ4 | | ellagic acid | Estrogen receptor | ESR1 | P03372 |
| KWYXZ4 | | ellagic acid | Androgen receptor | AR | P10275 |
| KWYXZ4 | | ellagic acid | Progesterone receptor | PGR | P06401 |
| KWYXZ4 | | ellagic acid | Heat shock protein HSP 90 | HSP90AA1 | P07900 |
| KWYXZ4 | | ellagic acid | Transcription factor p65 | RELA | Q04206 |
| KWYXZ4 | | ellagic acid | Vascular endothelial growth factor A | VEGFA | P15692 |
| KWYXZ4 | | ellagic acid | Cyclin-dependent kinase inhibitor 1 | CDKN1A | P38936 |
| KWYXZ4 | | ellagic acid | 72 kDa type IV collagenase | MMP2 | P08253 |
| KWYXZ4 | | ellagic acid | Matrix metalloproteinase-9 | MMP9 | P14780 |
| KWYXZ4 | | ellagic acid | NF-kappa-B inhibitor alpha | NFKBIA | P25963 |
| KWYXZ4 | | ellagic acid | Interleukin-8 | CXCL8 | P10145 |
| KWYXZ4 | | ellagic acid | Protein kinase C beta type | PRKCB | P05771 |
| KWYXZ4 | | ellagic acid | Glutathione S-transferase P | GSTP1 | P09211 |
| KWYXZ4 | | ellagic acid | Insulin-like growth factor II | IGF2 | P01344 |
| KWYXZ4 | | ellagic acid | Glutathione S-transferase Mu 1 | GSTM1 | P09488 |
| KWYXZ4 | | ellagic acid | Glutathione S-transferase Mu 2 | GSTM2 | P28161 |
| KWYXZ4 | | ellagic acid | Glutathione S-transferase A1 | GSTA1 | P08263 |
| KWYXZ4 | | ellagic acid | Glutathione S-transferase A2 | GSTA2 | P09210 |
| KWYXZ5 | | Gallic acid | Prostaglandin G/H synthase 1 | PTGS1 | P23219 |
| KWYXZ5 | | Gallic acid | Prostaglandin G/H synthase 2 | PTGS2 | P35354 |
| KWYXZ5 | | Gallic acid | Amine oxidase [flavin-containing] B | MAOB | P27338 |
| KWYXZ5 | | Gallic acid | Progesterone receptor | PGR | P06401 |
| KWYXZ5 | | Gallic acid | mRNA of Protein-tyrosine phosphatase, non-receptor type 1 | PTPN1 | P18031 |
| KWYXZ5 | | Gallic acid | DNA topoisomerase II | TOP2A | P11388 |
| KWYXZ5 | | Gallic acid | Heat shock protein HSP 90 | HSP90AA1 | P07900 |
| KWYXZ5 | | Gallic acid | Phosphatidylinositol-4,5-bisphosphate 3-kinase catalytic subunit, gamma isoform | PIK3CG | P48736 |
| KWYXZ5 | | Gallic acid | Caspase-9 | CASP9 | P55211 |
| KWYXZ5 | | Gallic acid | Caspase-3 | CASP3 | P42574 |
| KWYXZ5 | | Gallic acid | Cellular tumor antigen p53 | TP53 | P04637 |
| KWYXZ5 | | Gallic acid | Fatty acid synthase | FASN | P49327 |
| KWYXZ5 | | Gallic acid | Tumor necrosis factor ligand superfamily member 6 | FASLG | P48023 |
| KWYXZ5 | | Gallic acid | Microsomal glutathione S-transferase 1 | MGST1 | P10620 |
| KWYXZ5 | | Gallic acid | Cytochrome P450 3A43 | CYP3A43 | Q9HB55 |
| KWYXZ6 | | gallocatechin | Prostaglandin G/H synthase 1 | PTGS1 | P23219 |
| KWYXZ6 | | gallocatechin | Estrogen receptor | ESR1 | P03372 |
| KWYXZ6 | | gallocatechin | Prostaglandin G/H synthase 2 | PTGS2 | P35354 |
| KWYXZ6 | | gallocatechin | Heat shock protein HSP 90 | HSP90AA1 | P07900 |
| KWYXZ6 | | gallocatechin | Nuclear receptor coactivator 2 | NCOA2 | Q15596 |
| KWYXZ6 | | gallocatechin | Matrix metalloproteinase-9 | MMP9 | P14780 |
| KWYXZ7 | | corilagin | Transcription factor p65 | RELA | Q04206 |
| KWYXZ7 | | corilagin | Interleukin-10 | IL10 | P22301 |
| KWYXZ7 | | corilagin | Tumor necrosis factor | TNF | P01375 |
| KWYXZ7 | | corilagin | Interleukin-6 | IL6 | P05231 |
| KWYXZ7 | | corilagin | Prostaglandin G/H synthase 2 | PTGS2 | P35354 |
| KWYXZ7 | | corilagin | Heme oxygenase 1 | HMOX1 | P09601 |
| KWYXZ7 | | corilagin | Interleukin-1 beta | IL1B | P01584 |
| KWYXZ7 | | corilagin | Prolyl endopeptidase | PREP | P48147 |
| KWYXZ8 | | lupeol | Progesterone receptor | PGR | P06401 |
| KWYXZ8 | | lupeol | Apoptosis regulator Bcl-2 | BCL2 | P10415 |
| KWYXZ8 | | lupeol | Apoptosis regulator BAX | BAX | Q07812 |
| KWYXZ8 | | lupeol | Caspase-9 | CASP9 | P55211 |
| KWYXZ8 | | lupeol | Caspase-3 | CASP3 | P42574 |
| KWYXZ8 | | lupeol | Superoxide dismutase [Cu-Zn] | SOD1 | P00441 |
| KWYXZ8 | | lupeol | G2/mitotic-specific cyclin-B1 | CCNB1 | P14635 |
| KWYXZ8 | | lupeol | M-phase inducer phosphatase 3 | CDC25C | P30307 |
| KWYXZ8 | | lupeol | Apoptotic protease-activating factor 1 | APAF1 | O14727 |
| KWYXZ8 | | lupeol | Serine/threonine-protein kinase PLK1 | PLK1 | P53350 |
| KWYXZ8 | | lupeol | 14-3-3 protein sigma | SFN | P31947 |
| KWYXZ8 | | lupeol | Carnitine O-acetyltransferase | CRAT | P43155 |
| A2 | | oleanolic acid | Caspase-9 | CASP9 | P55211 |
| A2 | | oleanolic acid | Caspase-3 | CASP3 | P42574 |
| A2 | | oleanolic acid | Heme oxygenase 1 | HMOX1 | P09601 |
| A2 | | oleanolic acid | Intercellular adhesion molecule 1 | ICAM1 | P05362 |
| A2 | | oleanolic acid | NAD(P)H dehydrogenase [quinone] 1 | NQO1 | P15559 |
| A2 | | oleanolic acid | Pancreatic alpha-amylase | AMY2A | P04746 |
| KWYXZ9 | | ursolic acid | Urokinase-type plasminogen activator | PLAU | P00749 |
| KWYXZ9 | | ursolic acid | Cathepsin B | CTSB | P07858 |
| KWYXZ9 | | ursolic acid | Transcription factor p65 | RELA | Q04206 |
| KWYXZ9 | | ursolic acid | Signal transducer and activator of transcription 3 | STAT3 | P40763 |
| KWYXZ9 | | ursolic acid | Vascular endothelial growth factor A | VEGFA | P15692 |
| KWYXZ9 | | ursolic acid | G1/S-specific cyclin-D1 | CCND1 | P24385 |
| KWYXZ9 | | ursolic acid | Apoptosis regulator Bcl-2 | BCL2 | P10415 |
| KWYXZ9 | | ursolic acid | Bcl-2-like protein 1 | BCL2L1 | Q07817 |
| KWYXZ9 | | ursolic acid | Proto-oncogene c-Fos | FOS | P01100 |
| KWYXZ9 | | ursolic acid | Cyclin-dependent kinase inhibitor 1 | CDKN1A | P38936 |
| KWYXZ9 | | ursolic acid | Apoptosis regulator BAX | BAX | Q07812 |
| KWYXZ9 | | ursolic acid | Caspase-9 | CASP9 | P55211 |
| KWYXZ9 | | ursolic acid | 72 kDa type IV collagenase | MMP2 | P08253 |
| KWYXZ9 | | ursolic acid | Matrix metalloproteinase-9 | MMP9 | P14780 |
| KWYXZ9 | | ursolic acid | Cell division protein kinase 4 | CDK4 | P11802 |
| KWYXZ9 | | ursolic acid | Tumor necrosis factor | TNF | P01375 |
| KWYXZ9 | | ursolic acid | Transcription factor AP-1 | JUN | P05412 |
| KWYXZ9 | | ursolic acid | Interleukin-6 | IL6 | P05231 |
| KWYXZ9 | | ursolic acid | Cell division protein kinase 6 | Cdk6 | Q00534 |
| KWYXZ9 | | ursolic acid | Caspase-3 | CASP3 | P42574 |
| KWYXZ9 | | ursolic acid | Cellular tumor antigen p53 | TP53 | P04637 |
| KWYXZ9 | | ursolic acid | Mitogen-activated protein kinase 8 | MAPK8 | P45983 |
| KWYXZ9 | | ursolic acid | Prostaglandin G/H synthase 2 | PTGS2 | P35354 |
| KWYXZ9 | | ursolic acid | NF-kappa-B inhibitor alpha | NFKBIA | P25963 |
| KWYXZ9 | | ursolic acid | Caspase-8 | CASP8 | Q14790 |
| KWYXZ9 | | ursolic acid | Fatty acid synthase | FASN | P49327 |
| KWYXZ9 | | ursolic acid | Interstitial collagenase | MMP1 | P03956 |
| KWYXZ9 | | ursolic acid | Stromelysin-1 | MMP3 | P08254 |
| KWYXZ9 | | ursolic acid | Probable E3 ubiquitin-protein ligase HERC5 | HERC5 | Q9UII4 |
| KWYXZ9 | | ursolic acid | Heparin-binding growth factor 2 | FGF2 | P09038 |
| KWYXZ9 | | ursolic acid | Stromelysin-2 | MMP10 | P09238 |
| KWYXZ9 | | ursolic acid | Intercellular adhesion molecule 1 | ICAM1 | P05362 |
| KWYXZ9 | | ursolic acid | Interleukin-1 beta | IL1B | P01584 |
| KWYXZ9 | | ursolic acid | Cyclic AMP-responsive element-binding protein 1 | CREB1 | P16220 |
| KWYXZ9 | | ursolic acid | E-selectin | SELE | P16581 |
| KWYXZ9 | | ursolic acid | Prostaglandin E2 receptor EP3 subtype | PTGER3 | P43115 |
| KWYXZ9 | | ursolic acid | Prostaglandin G/H synthase 1 | PTGS1 | P23219 |
| KWYXZ9 | | ursolic acid | Induced myeloid leukemia cell differentiation protein Mcl-1 | MCL1 | Q07820 |
| KWYXZ9 | | ursolic acid | Protein kinase C gamma type | PRKCG | P05129 |
| KWYXZ9 | | ursolic acid | Cyclic AMP-dependent transcription factor ATF-2 | ATF2 | P15336 |
| KWYXZ9 | | ursolic acid | Granulocyte-macrophage colony-stimulating factor | CSF2 | P04141 |
| KWYXZ9 | | ursolic acid | Platelet endothelial cell adhesion molecule | PECAM1 | P16284 |
| KWYXZ9 | | ursolic acid | C-Jun-amino-terminal kinase-interacting protein 2 | MAPK8IP2 | Q13387 |
| KWYXZ9 | | ursolic acid | Baculoviral IAP repeat-containing protein 5 | BIRC5 | O15392 |
| KWYXZ9 | | ursolic acid | Tyrosine-protein phosphatase non-receptor type 6 | PTPN6 | P29350 |
| KWYXZ9 | | ursolic acid | Neuromodulin | GAP43 | P17677 |
| KWYXZ9 | | ursolic acid | Dual oxidase 2 | DUOX2 | Q9NRD8 |
| KWYXZ9 | | ursolic acid | Nitric oxide synthase, endothelial | NOS3 | P29474 |
| KWYXZ9 | | ursolic acid | Tyrosine-protein phosphatase non-receptor type 1 | PTPN1 | P18031 |
| KWYXZ9 | | ursolic acid | Phosphatidylinositol-3,4,5-trisphosphate 5-phosphatase 2 | INPPL1 | O15357 |
| KWYXZ9 | | ursolic acid | Lipopolysaccharide-induced tumor necrosis factor-alpha factor | LITAF | Q99732 |
| KWYXZ9 | | ursolic acid | G1/S-specific cyclin-D2 | CCND2 | P30279 |
| KWYXZ9 | | ursolic acid | Tumor necrosis factor ligand superfamily member 6 | FASLG | P48023 |
| KWYXZ9 | | ursolic acid | Caspase-1 | CASP1 | P29466 |
| KWYXZ9 | | ursolic acid | Ectonucleotide pyrophosphatase/phosphodiesterase family member 7 | ENPP7 | Q6UWV6 |
| KWYXZ10 | | linalool | Muscarinic acetylcholine receptor M1 | CHRM1 | P11229 |
| KWYXZ10 | | linalool | Gamma-aminobutyric-acid receptor alpha-2 subunit | GABRA2 | P47869 |
| KWYXZ10 | | linalool | Muscarinic acetylcholine receptor M2 | CHRM2 | P08172 |
| KWYXZ10 | | linalool | Gamma-aminobutyric-acid receptor alpha-3 subunit | GABRA3 | P34903 |
| KWYXZ10 | | linalool | Gamma-aminobutyric-acid receptor subunit alpha-6 | GABRA6 | Q16445 |
| KWYXZ10 | | linalool | Prostaglandin G/H synthase 1 | PTGS1 | P23219 |
| KWYXZ10 | | linalool | Potassium voltage-gated channel subfamily H member 2 | KCNH2 | Q12809 |
| KWYXZ10 | | linalool | Sodium channel protein type 5 subunit alpha | SCN5A | Q14524 |
| KWYXZ10 | | linalool | Coagulation factor Xa | F10 | P00742 |
| KWYXZ10 | | linalool | Prostaglandin G/H synthase 2 | PTGS2 | P35354 |
| KWYXZ10 | | linalool | Vascular endothelial growth factor receptor 2 | KDR | P35968 |
| KWYXZ10 | | linalool | DNA topoisomerase II | TOP2A | P11388 |
| KWYXZ10 | | linalool | Heat shock protein HSP 90 | HSP90AA1 | P07900 |
| KWYXZ10 | | linalool | Nuclear receptor coactivator 2 | NCOA2 | Q15596 |
| KWYXZ10 | | linalool | Calcium-activated potassium channel subunit alpha 1 | KCNMA1 | Q12791 |
| KWYXZ10 | | linalool | Muscarinic acetylcholine receptor M3 | CHRM3 | P20309 |
| KWYXZ10 | | linalool | Gamma-aminobutyric-acid receptor alpha-5 subunit | GABRA5 | P31644 |
| KWYXZ10 | | linalool | Gamma-aminobutyric acid receptor subunit alpha-1 | GABRA1 | P14867 |
| KWYXZ10 | | linalool | Sodium-dependent noradrenaline transporter | SLC6A2 | P23975 |
| KWYXZ10 | | linalool | Progesterone receptor | PGR | P06401 |
| KWYXZ11 | | phytol | Prostaglandin G/H synthase 2 | PTGS2 | P35354 |
| KWYXZ11 | | phytol | Retinoic acid receptor RXR-alpha | RXRA | P19793 |
| KWYXZ11 | | phytol | Muscarinic acetylcholine receptor M3 | CHRM3 | P20309 |
| KWYXZ11 | | phytol | Muscarinic acetylcholine receptor M1 | CHRM1 | P11229 |
| KWYXZ11 | | phytol | Gamma-aminobutyric acid receptor subunit alpha-1 | GABRA1 | P14867 |
| KWYXZ11 | | phytol | Peroxisome proliferator-activated receptor alpha | PPARA | Q07869 |
| KWYXZ12 | | palmitic acid | Cathepsin D | CTSD | P07339 |
| KWYXZ12 | | palmitic acid | Alcohol dehydrogenase 1B | ADH1B | P00325 |
| KWYXZ12 | | palmitic acid | Alcohol dehydrogenase 1C | ADH1C | P00326 |
| KWYXZ12 | | palmitic acid | Prostaglandin G/H synthase 1 | PTGS1 | P23219 |
| KWYXZ12 | | palmitic acid | Prostaglandin G/H synthase 2 | PTGS2 | P35354 |
| KWYXZ12 | | palmitic acid | Rhodopsin | RHO | P08100 |
| KWYXZ12 | | palmitic acid | Ig gamma-1 chain C region | IGHG1 | P01857 |
| KWYXZ12 | | palmitic acid | Nuclear receptor coactivator 2 | NCOA2 | Q15596 |
| KWYXZ12 | | palmitic acid | Apoptosis regulator Bcl-2 | BCL2 | P10415 |
| KWYXZ12 | | palmitic acid | Interleukin-10 | IL10 | P22301 |
| KWYXZ12 | | palmitic acid | Tumor necrosis factor | TNF | P01375 |
| KWYXZ12 | | palmitic acid | Collagen alpha-1(I) chain | COL1A1 | P02452 |
| KWYXZ12 | | palmitic acid | Phosphatidylinositol-3,4,5-trisphosphate 3-phosphatase and dual-specificity protein phosphatase PTEN | PTEN | P60484 |
| KWYXZ12 | | palmitic acid | Putative beta-glucuronidase-like protein SMA3 | GUSBP1 | Q15486 |
| KWYXZ12 | | palmitic acid | Solute carrier family 22 member 5 | SLC22A5 | O76082 |
| KWYXZ12 | | palmitic acid | Choline-phosphate cytidylyltransferase A | PCYT1A | P49585 |
| KWYXZ13 | | 4-hydroxybenzaldehyde | Alcohol dehydrogenase 1B | ADH1B | P00325 |
| KWYXZ13 | | 4-hydroxybenzaldehyde | Alcohol dehydrogenase 1C | ADH1C | P00326 |
| A1 | | vanillic acid | Prostaglandin G/H synthase 2 | PTGS2 | P35354 |
| A1 | | vanillic acid | Amine oxidase [flavin-containing] B | MAOB | P27338 |
| A1 | | vanillic acid | Amine oxidase [flavin-containing] A | MAOA | P21397 |
| A1 | | vanillic acid | Alpha-2A adrenergic receptor | ADRA2A | P08913 |
| A1 | | vanillic acid | Prostaglandin G/H synthase 1 | PTGS1 | P23219 |
| A1 | | vanillic acid | Nitric oxide synthase, endothelial | NOS3 | P29474 |
| A1 | | vanillic acid | 5,6-dihydroxyindole-2-carboxylic acid oxidase | TYRP1 | P17643 |
| KWYXZ14 | | apigenin | Prostaglandin G/H synthase 1 | PTGS1 | P23219 |
| KWYXZ14 | | apigenin | Androgen receptor | AR | P10275 |
| KWYXZ14 | | apigenin | Prostaglandin G/H synthase 2 | PTGS2 | P35354 |
| KWYXZ14 | | apigenin | Heat shock protein HSP 90 | HSP90AA1 | P07900 |
| KWYXZ14 | | apigenin | Trypsin-1 | PRSS1 | P07477 |
| KWYXZ14 | | apigenin | Nuclear receptor coactivator 2 | NCOA2 | Q15596 |
| KWYXZ14 | | apigenin | Phosphatidylinositol-4,5-bisphosphate 3-kinase catalytic subunit, gamma isoform | PIK3CG | P48736 |
| KWYXZ14 | | apigenin | mRNA of PKA Catalytic Subunit C-alpha | PRKACA | P17612 |
| KWYXZ14 | | apigenin | Sodium channel protein type 5 subunit alpha | SCN5A | Q14524 |
| KWYXZ14 | | apigenin | Coagulation factor Xa | F10 | P00742 |
| KWYXZ14 | | apigenin | Coagulation factor VII | F7 | P08709 |
| KWYXZ14 | | apigenin | DNA topoisomerase II | TOP2A | P11388 |
| KWYXZ14 | | apigenin | Dipeptidyl peptidase IV | DPP4 | P27487 |
| KWYXZ14 | | apigenin | Calmodulin | CALM1 | P0DP23 |
| KWYXZ14 | | apigenin | Transcription factor p65 | RELA | Q04206 |
| KWYXZ14 | | apigenin | RAC-alpha serine/threonine-protein kinase | AKT1 | P31749 |
| KWYXZ14 | | apigenin | Vascular endothelial growth factor A | VEGFA | P15692 |
| KWYXZ14 | | apigenin | G1/S-specific cyclin-D1 | CCND1 | P24385 |
| KWYXZ14 | | apigenin | Apoptosis regulator Bcl-2 | BCL2 | P10415 |
| KWYXZ14 | | apigenin | Bcl-2-like protein 1 | BCL2L1 | Q07817 |
| KWYXZ14 | | apigenin | Proto-oncogene c-Fos | FOS | P01100 |
| KWYXZ14 | | apigenin | Cyclin-dependent kinase inhibitor 1 | CDKN1A | P38936 |
| KWYXZ14 | | apigenin | Eukaryotic translation initiation factor 6 | EIF6 | P56537 |
| KWYXZ14 | | apigenin | Apoptosis regulator BAX | BAX | Q07812 |
| KWYXZ14 | | apigenin | Caspase-9 | CASP9 | P55211 |
| KWYXZ14 | | apigenin | Urokinase-type plasminogen activator | PLAU | P00749 |
| KWYXZ14 | | apigenin | Matrix metalloproteinase-9 | MMP9 | P14780 |
| KWYXZ14 | | apigenin | Retinoblastoma-associated protein | RB1 | P06400 |
| KWYXZ14 | | apigenin | Cell division protein kinase 4 | CDK4 | P11802 |
| KWYXZ14 | | apigenin | Tumor necrosis factor | TNF | P01375 |
| KWYXZ14 | | apigenin | Transcription factor AP-1 | JUN | P05412 |
| KWYXZ14 | | apigenin | Cell division protein kinase 6 | Cdk6 | Q00534 |
| KWYXZ14 | | apigenin | Cyclin-dependent kinase inhibitor 2A, isoforms 1/2/3 | CDKN2A | Q8N726 |
| KWYXZ14 | | apigenin | Eukaryotic translation elongation factor 1 epsilon-1 | EEF1E1 | O43324 |
| KWYXZ14 | | apigenin | Activator of 90 kDa heat shock protein ATPase homolog 1 | AHSA1 | O95433 |
| KWYXZ14 | | apigenin | Caspase-3 | CASP3 | P42574 |
| KWYXZ14 | | apigenin | Cellular tumor antigen p53 | TP53 | P04637 |
| KWYXZ14 | | apigenin | NF-kappa-B inhibitor alpha | NFKBIA | P25963 |
| KWYXZ14 | | apigenin | Ornithine decarboxylase | ODC1 | P11926 |
| KWYXZ14 | | apigenin | E3 ubiquitin-protein ligase Mdm2 | MDM2 | Q00987 |
| KWYXZ14 | | apigenin | Bcl2 antagonist of cell death | BAD | Q92934 |
| KWYXZ14 | | apigenin | Interstitial collagenase | MMP1 | P03956 |
| KWYXZ14 | | apigenin | Hypoxia-inducible factor 1-alpha | HIF1A | Q16665 |
| KWYXZ14 | | apigenin | Insulin-like growth factor 1 receptor | IGF1R | P08069 |
| KWYXZ14 | | apigenin | Protein CBFA2T1 | RUNX1T1 | Q06455 |
| KWYXZ14 | | apigenin | Probable E3 ubiquitin-protein ligase HERC5 | HERC5 | Q9UII4 |
| KWYXZ14 | | apigenin | Acetyl-CoA carboxylase 1 | ACACA | Q13085 |
| KWYXZ14 | | apigenin | Heme oxygenase 1 | HMOX1 | P09601 |
| KWYXZ14 | | apigenin | Intercellular adhesion molecule 1 | ICAM1 | P05362 |
| KWYXZ14 | | apigenin | Induced myeloid leukemia cell differentiation protein Mcl-1 | MCL1 | Q07820 |
| KWYXZ14 | | apigenin | G1/S-specific cyclin-D2 | CCND2 | P30279 |
| KWYXZ14 | | apigenin | Interleukin-2 | IL2 | P60568 |
| KWYXZ14 | | apigenin | G2/mitotic-specific cyclin-B1 | CCNB1 | P14635 |
| KWYXZ14 | | apigenin | Plasminogen activator inhibitor 1 | SERPINE1 | P05121 |
| KWYXZ14 | | apigenin | Interferon gamma | IFNG | P01579 |
| KWYXZ14 | | apigenin | Interleukin-4 | IL4 | P05112 |
| KWYXZ14 | | apigenin | NF-kappa-B essential modulator | IKBKG | Q9Y6K9 |
| KWYXZ14 | | apigenin | Cytochrome P450 19A1 | CYP19A1 | P11511 |
| KWYXZ14 | | apigenin | Baculoviral IAP repeat-containing protein 4 | XIAP | P98170 |
| KWYXZ14 | | apigenin | 26S proteasome non-ATPase regulatory subunit 3 | PSMD3 | O43242 |
| KWYXZ14 | | apigenin | Solute carrier family 2, facilitated glucose transporter member 4 | SLC2A4 | P14672 |
| KWYXZ14 | | apigenin | Insulin receptor | INSR | P06213 |
| KWYXZ14 | | apigenin | CD40 ligand | CD40LG | P29965 |
| KWYXZ14 | | apigenin | Cytochrome c | CYCS | P99999 |
| KWYXZ14 | | apigenin | CASP8 and FADD-like apoptosis regulator | CFLAR | O15519 |
| KWYXZ14 | | apigenin | Alpha- and gamma-adaptin-binding protein p34 | AAGAB | Q6PD74 |
| KWYXZ14 | | apigenin | Insulin | INS | P01308 |
| KWYXZ14 | | apigenin | Low affinity immunoglobulin epsilon Fc receptor | FCER2 | P06734 |
| KWYXZ14 | | apigenin | Interleukin-13 | IL13 | P35225 |
| KWYXZ14 | | apigenin | High affinity immunoglobulin epsilon receptor subunit beta | MS4A2 | Q01362 |
| KWYXZ14 | | apigenin | Intestinal-type alkaline phosphatase | ALPI | P09923 |
| KWYXZ14 | | apigenin | Proteasome activator complex subunit 3 | PSME3 | P61289 |
| KWYXZ14 | | apigenin | Glucose-6-phosphatase | G6PC | P35575 |
| KWYXZ14 | | apigenin | Adenomatous polyposis coli protein | APC | P25054 |
| KWYXZ14 | | apigenin | Transient receptor potential cation channel subfamily M member 2 | TRPM2 | O94759 |
| KWYXZ14 | | apigenin | Aldo-keto reductase family 1 member C3 | AKR1C3 | P42330 |
| KWYXZ14 | | apigenin | Sodium/iodide cotransporter | SLC5A5 | Q92911 |
| KWYXZ14 | | apigenin | Sodium/potassium-transporting ATPase subunit gamma | FXYD2 | P54710 |
| KWYXZ14 | | apigenin | Dolichyl-phosphate beta-glucosyltransferase | ALG5 | Q9Y673 |
| KWYXZ15 | | Hinokinin | Norepinephrine transporter | SLC6A2 | P23975 |
| KWYXZ15 | | Hinokinin | Dopamine transporter | SLC6A3 | Q01959 |
| SQ1 | | DFV | Prostaglandin G/H synthase 1 | PTGS1 | P23219 |
| SQ1 | | DFV | Estrogen receptor | ESR1 | P03372 |
| SQ1 | | DFV | Prostaglandin G/H synthase 2 | PTGS2 | P35354 |
| SQ1 | | DFV | Retinoic acid receptor RXR-alpha | RXRA | P19793 |
| SQ1 | | DFV | Beta-2 adrenergic receptor | ADRB2 | P07550 |
| SQ1 | | DFV | Heat shock protein HSP 90 | HSP90AA1 | P07900 |
| SQ1 | | DFV | Phosphatidylinositol-4,5-bisphosphate 3-kinase catalytic subunit, gamma isoform | PIK3CG | P48736 |
| SQ1 | | DFV | mRNA of PKA Catalytic Subunit C-alpha | PRKACA | P17612 |
| SQ1 | | DFV | Beta-lactamase | DPEP1 | P16444 |
| SQ1 | | DFV | Amine oxidase [flavin-containing] B | MAOB | P27338 |
| SQ1 | | DFV | Sodium-dependent serotonin transporter | SLC6A4 | P31645 |
| SQ1 | | DFV | cAMP-dependent protein kinase inhibitor alpha | PKIA | P61925 |
| SQ2 | | Mandenol | Prostaglandin G/H synthase 1 | PTGS1 | P23219 |
| SQ2 | | Mandenol | Prostaglandin G/H synthase 2 | PTGS2 | P35354 |
| SQ2 | | Mandenol | Nuclear receptor coactivator 2 | NCOA2 | Q15596 |
| SQ2 | | Diop | Sodium channel protein type 5 subunit alpha | SCN5A | Q14524 |
| SQ2 | | Diop | Beta-2 adrenergic receptor | ADRB2 | P07550 |
| SQ2 | | Diop | Muscarinic acetylcholine receptor M3 | CHRM3 | P20309 |
| SQ3 | | ginsenoside rh2 | Apoptosis regulator BAX | BAX | Q07812 |
| SQ3 | | ginsenoside rh2 | Tumor necrosis factor | TNF | P01375 |
| SQ3 | | ginsenoside rh2 | Caspase-3 | CASP3 | P42574 |
| SQ3 | | ginsenoside rh2 | Prostaglandin G/H synthase 2 | PTGS2 | P35354 |
| SQ3 | | ginsenoside rh2 | NF-kappa-B inhibitor alpha | NFKBIA | P25963 |
| SQ3 | | ginsenoside rh2 | Interleukin-1 beta | IL1B | P01584 |
| SQ3 | | ginsenoside rh2 | Caspase-1 | CASP1 | P29466 |
| SQ3 | | ginsenoside rh2 | Interferon gamma | IFNG | P01579 |
| SQ3 | | ginsenoside rh2 | Pituitary adenylate cyclase-activating polypeptide | ADCYAP1 | P18509 |
| SQ3 | | ginsenoside rh2 | Proteasome assembly chaperone 1 | PSMG1 | O95456 |
| SQ3 | | ginsenoside rh2 | Dual specificity mitogen-activated protein kinase kinase 4 | MAP2K4 | P45985 |
| SQ3 | | ginsenoside rh2 | Solute carrier family 2, facilitated glucose transporter member 4 | SLC2A4 | P14672 |
| SQ4 | | Stigmasterol | Progesterone receptor | PGR | P06401 |
| SQ4 | | Stigmasterol | Mineralocorticoid receptor | NR3C2 | P08235 |
| SQ4 | | Stigmasterol | Nuclear receptor coactivator 2 | NCOA2 | Q15596 |
| SQ4 | | Stigmasterol | Alcohol dehydrogenase 1C | ADH1C | P00326 |
| SQ4 | | Stigmasterol | Ig gamma-1 chain C region | IGHG1 | P01857 |
| SQ4 | | Stigmasterol | Retinoic acid receptor RXR-alpha | RXRA | P19793 |
| SQ4 | | Stigmasterol | Nuclear receptor coactivator 1 | NCOA1 | Q15788 |
| SQ4 | | Stigmasterol | Prostaglandin G/H synthase 1 | PTGS1 | P23219 |
| SQ4 | | Stigmasterol | Prostaglandin G/H synthase 2 | PTGS2 | P35354 |
| SQ4 | | Stigmasterol | Alpha-2A adrenergic receptor | ADRA2A | P08913 |
| SQ4 | | Stigmasterol | Sodium-dependent noradrenaline transporter | SLC6A2 | P23975 |
| SQ4 | | Stigmasterol | Sodium-dependent dopamine transporter | SLC6A3 | Q01959 |
| SQ4 | | Stigmasterol | Beta-2 adrenergic receptor | ADRB2 | P07550 |
| SQ4 | | Stigmasterol | Aldose reductase | AKR1B1 | P15121 |
| SQ4 | | Stigmasterol | Urokinase-type plasminogen activator | PLAU | P00749 |
| SQ4 | | Stigmasterol | Leukotriene A-4 hydrolase | LTA4H | P09960 |
| SQ4 | | Stigmasterol | Amine oxidase [flavin-containing] B | MAOB | P27338 |
| SQ4 | | Stigmasterol | Amine oxidase [flavin-containing] A | MAOA | P21397 |
| SQ4 | | Stigmasterol | mRNA of PKA Catalytic Subunit C-alpha | PRKACA | P17612 |
| SQ4 | | Stigmasterol | Chymotrypsinogen B | CTRB1 | P17538 |
| SQ4 | | Stigmasterol | Muscarinic acetylcholine receptor M3 | CHRM3 | P20309 |
| SQ4 | | Stigmasterol | Muscarinic acetylcholine receptor M1 | CHRM1 | P11229 |
| SQ4 | | Stigmasterol | Beta-1 adrenergic receptor | ADRB1 | P08588 |
| SQ4 | | Stigmasterol | Sodium channel protein type 5 subunit alpha | SCN5A | Q14524 |
| SQ4 | | Stigmasterol | 5-hydroxytryptamine 2A receptor | HTR2A | P28223 |
| SQ4 | | Stigmasterol | Alpha-1A adrenergic receptor | ADRA1A | P35348 |
| SQ4 | | Stigmasterol | Gamma-aminobutyric-acid receptor alpha-3 subunit | GABRA3 | P34903 |
| SQ4 | | Stigmasterol | Muscarinic acetylcholine receptor M2 | CHRM2 | P08172 |
| SQ4 | | Stigmasterol | Alpha-1B adrenergic receptor | ADRA1B | P35368 |
| SQ4 | | Stigmasterol | Gamma-aminobutyric acid receptor subunit alpha-1 | GABRA1 | P14867 |
| SQ4 | | Stigmasterol | Neuronal acetylcholine receptor protein, alpha-7 chain | CHRNA7 | P36544 |
| SDH1 | | Catalpol | Dipeptidyl peptidase IV | DPP4 | P27487 |
| SDH1 | | Catalpol | Apoptosis regulator Bcl-2 | BCL2 | P10415 |
| SDH1 | | Catalpol | Caspase-3 | CASP3 | P42574 |
| SDH2 | | Gamma-Aminobutyric Acid | Cathepsin D | CTSD | P07339 |
| SDH2 | | Gamma-Aminobutyric Acid | Cholinesterase | BCHE | P06276 |
| SDH2 | | Gamma-Aminobutyric Acid | Alcohol dehydrogenase 1B | ADH1B | P00325 |
| SDH2 | | Gamma-Aminobutyric Acid | Alcohol dehydrogenase 1C | ADH1C | P00326 |
| SDH2 | | Gamma-Aminobutyric Acid | 1-aminocyclopropane-1-carboxylate deaminase | ACCS | Q96QU6 |
| SDH2 | | Gamma-Aminobutyric Acid | Glycine amidinotransferase, mitochondrial | GATM | P50440 |
| SDH2 | | Gamma-Aminobutyric Acid | Trypsin-3 | PRSS3 | P35030 |
| SDH2 | | Gamma-Aminobutyric Acid | 4-aminobutyrate aminotransferase, mitochondrial | ABAT | P80404 |
| SDH2 | | Gamma-Aminobutyric Acid | mRNA of PKA Catalytic Subunit C-alpha | PRKACA | P17612 |
| SDH2 | | Gamma-Aminobutyric Acid | Alcohol dehydrogenase 1A | ADH1A | P07327 |
| SDH2 | | Gamma-Aminobutyric Acid | Interleukin-6 | IL6 | P05231 |
| SDH2 | | Gamma-Aminobutyric Acid | Transitional endoplasmic reticulum ATPase | VCP | P55072 |
| SDH2 | | Gamma-Aminobutyric Acid | Sodium- and chloride-dependent GABA transporter 1 | SLC6A1 | P30531 |
| SDH3 | | [Acteoside](http://www.megabionet.org/tcmid/ingredient/23064/) | Intercellular adhesion molecule 1 | ICAM1 | P05362 |
| SDH4 | | Rehmaglutin D | Gamma-aminobutyric acid receptor subunit alpha-1 | GABRA1 | P14867 |
| SDH5 | | gentistic acid | Prostaglandin G/H synthase 1 | PTGS1 | P23219 |
| SDH5 | | gentistic acid | Arachidonate 5-lipoxygenase | ALOX5 | P09917 |
| SDH5 | | gentistic acid | Prostaglandin G/H synthase 2 | PTGS2 | P35354 |
| SDH5 | | gentistic acid | Amine oxidase [flavin-containing] B | MAOB | P27338 |
| SDH5 | | gentistic acid | Alcohol dehydrogenase 1C | ADH1C | P00326 |
| SDH5 | | gentistic acid | Chymotrypsinogen B | CTRB1 | P17538 |
| SDH5 | | gentistic acid | Carbonic anhydrase II | CA2 | P00918 |
| SDH5 | | gentistic acid | Carbonic anhydrase I | CA1 | P00915 |
| SDH5 | | gentistic acid | Carbonic anhydrase XII | CA12 | O43570 |
| SDH5 | | gentistic acid | Carbonic anhydrase IX | CA9 | Q16790 |
| SDH6 | | versulin | Prostaglandin G/H synthase 1 | PTGS1 | P23219 |
| SDH6 | | versulin | Androgen receptor | AR | P10275 |
| SDH6 | | versulin | Prostaglandin G/H synthase 2 | PTGS2 | P35354 |
| SDH6 | | versulin | Heat shock protein HSP 90 | HSP90AA1 | P07900 |
| SDH6 | | versulin | Trypsin-1 | PRSS1 | P07477 |
| SDH6 | | versulin | Nuclear receptor coactivator 2 | NCOA2 | Q15596 |
| SDH6 | | versulin | Phosphatidylinositol-4,5-bisphosphate 3-kinase catalytic subunit, gamma isoform | PIK3CG | P48736 |
| SDH6 | | versulin | mRNA of PKA Catalytic Subunit C-alpha | PRKACA | P17612 |
| SDH6 | | versulin | Sodium channel protein type 5 subunit alpha | SCN5A | Q14524 |
| SDH6 | | versulin | Coagulation factor Xa | F10 | P00742 |
| SDH6 | | versulin | Coagulation factor VII | F7 | P08709 |
| SDH6 | | versulin | DNA topoisomerase II | TOP2A | P11388 |
| SDH6 | | versulin | Dipeptidyl peptidase IV | DPP4 | P27487 |
| SDH6 | | versulin | Calmodulin | CALM1 | P0DP23 |
| SDH6 | | versulin | Transcription factor p65 | RELA | Q04206 |
| SDH6 | | versulin | RAC-alpha serine/threonine-protein kinase | AKT1 | P31749 |
| SDH6 | | versulin | Vascular endothelial growth factor A | VEGFA | P15692 |
| SDH6 | | versulin | G1/S-specific cyclin-D1 | CCND1 | P24385 |
| SDH6 | | versulin | Apoptosis regulator Bcl-2 | BCL2 | P10415 |
| SDH6 | | versulin | Bcl-2-like protein 1 | BCL2L1 | Q07817 |
| SDH6 | | versulin | Proto-oncogene c-Fos | FOS | P01100 |
| SDH6 | | versulin | Cyclin-dependent kinase inhibitor 1 | CDKN1A | P38936 |
| SDH6 | | versulin | Eukaryotic translation initiation factor 6 | EIF6 | P56537 |
| SDH6 | | versulin | Apoptosis regulator BAX | BAX | Q07812 |
| SDH6 | | versulin | Caspase-9 | CASP9 | P55211 |
| SDH6 | | versulin | Urokinase-type plasminogen activator | PLAU | P00749 |
| SDH6 | | versulin | Matrix metalloproteinase-9 | MMP9 | P14780 |
| SDH6 | | versulin | Retinoblastoma-associated protein | RB1 | P06400 |
| SDH6 | | versulin | Cell division protein kinase 4 | CDK4 | P11802 |
| SDH6 | | versulin | Tumor necrosis factor | TNF | P01375 |
| SDH6 | | versulin | Transcription factor AP-1 | JUN | P05412 |
| SDH6 | | versulin | Cell division protein kinase 6 | Cdk6 | Q00534 |
| SDH6 | | versulin | Cyclin-dependent kinase inhibitor 2A, isoforms 1/2/3 | CDKN2A | Q8N726 |
| SDH6 | | versulin | Eukaryotic translation elongation factor 1 epsilon-1 | EEF1E1 | O43324 |
| SDH6 | | versulin | Activator of 90 kDa heat shock protein ATPase homolog 1 | AHSA1 | O95433 |
| SDH6 | | versulin | Caspase-3 | CASP3 | P42574 |
| SDH6 | | versulin | Cellular tumor antigen p53 | TP53 | P04637 |
| SDH6 | | versulin | NF-kappa-B inhibitor alpha | NFKBIA | P25963 |
| SDH6 | | versulin | Ornithine decarboxylase | ODC1 | P11926 |
| SDH6 | | versulin | E3 ubiquitin-protein ligase Mdm2 | MDM2 | Q00987 |
| SDH6 | | versulin | Bcl2 antagonist of cell death | BAD | Q92934 |
| SDH6 | | versulin | Interstitial collagenase | MMP1 | P03956 |
| SDH6 | | versulin | Hypoxia-inducible factor 1-alpha | HIF1A | Q16665 |
| SDH6 | | versulin | Insulin-like growth factor 1 receptor | IGF1R | P08069 |
| SDH6 | | versulin | Protein CBFA2T1 | RUNX1T1 | Q06455 |
| SDH6 | | versulin | Probable E3 ubiquitin-protein ligase HERC5 | HERC5 | Q9UII4 |
| SDH6 | | versulin | Acetyl-CoA carboxylase 1 | ACACA | Q13085 |
| SDH6 | | versulin | Heme oxygenase 1 | HMOX1 | P09601 |
| SDH6 | | versulin | Intercellular adhesion molecule 1 | ICAM1 | P05362 |
| SDH6 | | versulin | Induced myeloid leukemia cell differentiation protein Mcl-1 | MCL1 | Q07820 |
| SDH6 | | versulin | G1/S-specific cyclin-D2 | CCND2 | P30279 |
| SDH6 | | versulin | Interleukin-2 | IL2 | P60568 |
| SDH6 | | versulin | G2/mitotic-specific cyclin-B1 | CCNB1 | P14635 |
| SDH6 | | versulin | Plasminogen activator inhibitor 1 | SERPINE1 | P05121 |
| SDH6 | | versulin | Interferon gamma | IFNG | P01579 |
| SDH6 | | versulin | Interleukin-4 | IL4 | P05112 |
| SDH6 | | versulin | NF-kappa-B essential modulator | IKBKG | Q9Y6K9 |
| SDH6 | | versulin | Cytochrome P450 19A1 | CYP19A1 | P11511 |
| SDH6 | | versulin | Baculoviral IAP repeat-containing protein 4 | XIAP | P98170 |
| SDH6 | | versulin | 26S proteasome non-ATPase regulatory subunit 3 | PSMD3 | O43242 |
| SDH6 | | versulin | Solute carrier family 2, facilitated glucose transporter member 4 | SLC2A4 | P14672 |
| SDH6 | | versulin | Insulin receptor | INSR | P06213 |
| SDH6 | | versulin | CD40 ligand | CD40LG | P29965 |
| SDH6 | | versulin | Cytochrome c | CYCS | P99999 |
| SDH6 | | versulin | CASP8 and FADD-like apoptosis regulator | CFLAR | O15519 |
| SDH6 | | versulin | Alpha- and gamma-adaptin-binding protein p34 | AAGAB | Q6PD74 |
| SDH6 | | versulin | Insulin | INS | P01308 |
| SDH6 | | versulin | Low affinity immunoglobulin epsilon Fc receptor | FCER2 | P06734 |
| SDH6 | | versulin | Interleukin-13 | IL13 | P35225 |
| SDH6 | | versulin | High affinity immunoglobulin epsilon receptor subunit beta | MS4A2 | Q01362 |
| SDH6 | | versulin | Intestinal-type alkaline phosphatase | ALPI | P09923 |
| SDH6 | | versulin | Proteasome activator complex subunit 3 | PSME3 | P61289 |
| SDH6 | | versulin | Glucose-6-phosphatase | G6PC | P35575 |
| SDH6 | | versulin | Adenomatous polyposis coli protein | APC | P25054 |
| SDH6 | | versulin | Transient receptor potential cation channel subfamily M member 2 | TRPM2 | O94759 |
| SDH6 | | versulin | Aldo-keto reductase family 1 member C3 | AKR1C3 | P42330 |
| SDH6 | | versulin | Sodium/iodide cotransporter | SLC5A5 | Q92911 |
| SDH6 | | versulin | Sodium/potassium-transporting ATPase subunit gamma | FXYD2 | P54710 |
| SDH6 | | versulin | Dolichyl-phosphate beta-glucosyltransferase | ALG5 | Q9Y673 |
| SDH6 | | versulin | NADPH oxidase 4 | NOX4 | Q9NPH5 |
| SDH6 | | versulin | Aldose reductase (by homology) | AKR1B1 | P15121 |
| SDH6 | | versulin | Cyclin-dependent kinase 5/CDK5 activator 1 | CDK5R1 | Q15078 |
| SDH6 | | versulin | Xanthine dehydrogenase | XDH | P47989 |
| SDH6 | | versulin | Monoamine oxidase A | MAOA | P21397 |
| SDH6 | | versulin | Tyrosine-protein kinase receptor FLT3 | Flt3 | P36888 |
| SDH6 | | versulin | Estrogen receptor alpha | ESR1 | P03372 |
| SDH6 | | versulin | Cyclin-dependent kinase 1/cyclin B | CCNB3 | Q8WWL7 |
| SDH6 | | versulin | Acetylcholinesterase | ACHE | P22303 |
| SDH6 | | versulin | Adenosine A1 receptor (by homology) | ADORA1 | P30542 |
| SDH6 | | versulin | Estrogen receptor beta | ESR2 | Q92731 |
| SDH6 | | versulin | Adenosine A2a receptor (by homology) | ADORA2A | P29274 |
| SDH6 | | versulin | Tyrosine-protein kinase SYK | SYK | P43405 |
| SDH6 | | versulin | Glycogen synthase kinase-3 beta | GSK3B | P49841 |
| SDH6 | | versulin | Multidrug resistance-associated protein 1 | ABCC1 | P33527 |
| SDH6 | | versulin | Estradiol 17-beta-dehydrogenase 1 | HSD17B1 | P14061 |
| SDH6 | | versulin | Transthyretin | TTR | P02766 |
| SDH6 | | versulin | Casein kinase II alpha | CSNK2A1 | P68400 |
| SDH6 | | versulin | Cystic fibrosis transmembrane conductance regulator | CFTR | P13569 |
| SDH6 | | versulin | Cytochrome P450 1B1 | CYP1B1 | Q16678 |
| SDH6 | | versulin | ATP-binding cassette sub-family G member 2 | ABCG2 | Q9UNQ0 |
| SDH6 | | versulin | Aldo-keto reductase family 1 member B10 | AKR1B10 | O60218 |
| SDH6 | | versulin | Tankyrase-2 | TNKS2 | Q9H2K2 |
| SDH6 | | versulin | Tankyrase-1 | TNKS | O95271 |
| SDH7 | | Rehmaglutin A | Heat shock protein HSP 90-alpha | HSP90AA1 | P07900 |
| SDH8 | | Verbascoside | Protein kinase C alpha | PRKCA | P17252 |
| SDH8 | | Verbascoside | Matrix metalloproteinase 2 | MMP2 | P08253 |
| SDH8 | | Verbascoside | Matrix metalloproteinase 12 | MMP12 | P39900 |
