# Supplementary material for: Network Pharmacology Approach to Explore the Potential Mechanisms of Jieduan-Niwan Formula Treating Acute-on-Chronic Liver Failure
Source: Evid Based Complement Alternat Med. 2020 Dec 30;2020:1041307. doi: 10.1155/2020/1041307 (PMC7787753; doi:10.1155/2020/1041307)
Supplement: Supplementary Materials — Supplementary Material 1: Table S1: information of potentially bioactive compounds of Jieduan-Niwan Formula. Supplementary Material 2: Table S2: the details of targets from compounds in JDNW Formula. Supplementary Material 3: Table S3: 1471 known ACLF-related targets. Supplementary Material 4: Table S4: 168 potential targets shared in JDNW Formula and ACLF. Supplementary Material 5: Table S5: information of potentially bioactive compounds of 168 common targets. Supplementary Material 6: Table S6: GO cellular component enrichment analysis of key targets of JDNW Formula in the treatment of ACLF. Supplementary Material 7: Table S7: KEGG pathway enrichment analysis of key targets of JDNW Formula in the treatment of ACLF. [file 1041307.f1.zip › 1041307.f1/Table S3.1041307.v2.docx]

| **1471 Known ACLF-related targets** | | | |
| --- | --- | --- | --- |
| **Gene Symbol** | **Description** | **Uniprot ID** | **Source** |
| AFP | Alpha-fetoprotein | P02771 | DisGeNET,GeneCard |
| ALB | Serum albumin | P02768 | DisGeNET,GeneCard |
| ARNTL2 | Aryl hydrocarbon receptor nuclear translocator-like protein 2 | Q8WYA1 | DisGeNET |
| IL6 | Interleukin-6 | P05231 | DisGeNET,OMIM,GeneCard |
| APEX1 | DNA-(apurinic or apyrimidinic site) endonuclease | P27695 | DisGeNET,GeneCard |
| APOA5 | Apolipoprotein A-V | Q6Q788 | DisGeNET,GeneCard |
| IL23A | Interleukin-23 subunit alpha | Q9NPF7 | DisGeNET |
| AVP | Vasopressin-neurophysin 2-copeptin | P01185 | DisGeNET,GeneCard |
| BCHE | Cholinesterase | P06276 | DisGeNET,GeneCard |
| BCL2 | Apoptosis regulator Bcl-2 | P10415 | DisGeNET,GeneCard |
| BRAF | Serine/threonine-protein kinase B-raf | P15056 | OMIM,GeneCard |
| GTF2H1 | General transcription factor IIH subunit 1 | P32780 | DisGeNET |
| KCTD9 | BTB/POZ domain-containing protein KCTD9 | Q7L273 | DisGeNET |
| CASP1 | Caspase-1 | P29466 | DisGeNET,GeneCard |
| DCTN4 | Dynactin subunit 4 | Q9UJW0 | DisGeNET |
| IL22 | Interleukin-22 | Q9GZX6 | DisGeNET |
| CCL4 | C-C motif chemokine 4 | P13236 | DisGeNET,GeneCard |
| CCR6 | C-C chemokine receptor type 6 | P51684 | DisGeNET,GeneCard |
| NUP62 | Nuclear pore glycoprotein p62 | P37198 | DisGeNET |
| HGF | Hepatocyte growth factor | P14210 | DisGeNET,OMIM,GeneCard |
| CD163 | Scavenger receptor cysteine-rich type 1 protein M130 | Q86VB7 | DisGeNET,GeneCard |
| IL23R | Interleukin-23 receptor | Q5VWK5 | DisGeNET |
| KHDRBS1 | KH domain-containing, RNA-binding, signal transduction-associated protein 1 | Q07666 | DisGeNET |
| SQSTM1 | Sequestosome-1 | Q13501 | DisGeNET |
| CD34 | Hematopoietic progenitor cell antigen CD34 | P28906 | DisGeNET,GeneCard |
| MIR542 | Not Available | Not Available | DisGeNET |
| MSC | Musculin | O60682 | DisGeNET |
| SPRR2A | Small proline-rich protein 2A | P35326 | DisGeNET |
| SSRP1 | FACT complex subunit SSRP1 | Q08945 | DisGeNET |
| TEAD1 | Transcriptional enhancer factor TEF-1 | P28347 | DisGeNET |
| CPT2 | Carnitine O-palmitoyltransferase 2, mitochondrial | P23786 | OMIM,GeneCard |
| BNIP3 | BCL2/adenovirus E1B 19 kDa protein-interacting protein 3 | Q12983 | DisGeNET |
| SEMA6A | Semaphorin-6A | Q9H2E6 | DisGeNET |
| TLR3 | Toll-like receptor 3 | O15455 | DisGeNET,OMIM,GeneCard |
| CRP | C-reactive protein [Cleaved into: C-reactive protein | P02741 | DisGeNET,GeneCard |
| CSF2 | Granulocyte-macrophage colony-stimulating factor | P04141 | DisGeNET,GeneCard |
| RARRES2 | Retinoic acid receptor responder protein 2 | Q99969 | DisGeNET |
| ATG5 | Autophagy protein 5 | Q9H1Y0 | DisGeNET |
| RORA | Nuclear receptor ROR-alpha | P35398 | DisGeNET |
| OPN1SW | Short-wave-sensitive opsin 1 | P03999 | DisGeNET |
| CSF3 | Granulocyte colony-stimulating factor | P09919 | DisGeNET,GeneCard |
| SAMSN1 | SAM domain-containing protein SAMSN-1 | Q9NSI8 | DisGeNET |
| CD247 | T-cell surface glycoprotein CD3 zeta chain | P20963 | DisGeNET |
| NMI | N-myc-interactor | Q13287 | DisGeNET |
| CDR3 | Not Available | Not Available | DisGeNET |
| H4C9 | Not Available | Not Available | DisGeNET |
| CST3 | Cystatin-C | P01034 | DisGeNET,GeneCard |
| H4C1 | Not Available | Not Available | DisGeNET |
| H4C4 | Not Available | Not Available | DisGeNET |
| H4C6 | Not Available | Not Available | DisGeNET |
| H4C5 | Not Available | Not Available | DisGeNET |
| H4C2 | Not Available | Not Available | DisGeNET |
| H4C11 | Not Available | Not Available | DisGeNET |
| H4C8 | Not Available | Not Available | DisGeNET |
| TFPI2 | Tissue factor pathway inhibitor 2 | P48307 | DisGeNET |
| H4C13 | Not Available | Not Available | DisGeNET |
| H4C14 | Not Available | Not Available | DisGeNET |
| RGN | Regucalcin | Q15493 | DisGeNET |
| CXCL10 | C-X-C motif chemokine 10 | P02778 | DisGeNET,GeneCard |
| TRAF6 | TNF receptor-associated factor 6 | Q9Y4K3 | DisGeNET |
| SELENBP1 | Methanethiol oxidase | Q13228 | DisGeNET |
| H4C12 | Not Available | Not Available | DisGeNET |
| CXCL12 | Stromal cell-derived factor 1 | P48061 | DisGeNET,GeneCard |
| FSD1 | Fibronectin type III and SPRY domain-containing protein 1 | Q9BTV5 | DisGeNET |
| FSD1L | FSD1-like protein | Q9BXM9 | DisGeNET |
| H4C3 | Not Available | Not Available | DisGeNET |
| HIF1A-AS1 | Not Available | Not Available | DisGeNET |
| GLA | Alpha-galactosidase A | P06280 | DisGeNET |
| IL37 | Interleukin-37 | Q9NZH6 | DisGeNET |
| GLUL | Glutamine synthetase | P15104 | DisGeNET,OMIM,GeneCard |
| STON1-GTF2A1L | Not Available | Not Available | DisGeNET |
| CXCL8 | Interleukin-8 | P10145 | DisGeNET,GeneCard |
| KLRC4-KLRK1 | Not Available | Not Available | DisGeNET |
| CXCR1 | C-X-C chemokine receptor type 1 | P25024 | DisGeNET,GeneCard |
| HINT1 | Histidine triad nucleotide-binding protein 1 | P49773 | DisGeNET |
| IL27 | Interleukin-27 subunit alpha | Q8NEV9 | DisGeNET |
| CXCR2 | C-X-C chemokine receptor type 2 | P25025 | DisGeNET,GeneCard |
| EZH2 | Histone-lysine N-methyltransferase EZH2 | Q15910 | DisGeNET,OMIM,GeneCard |
| GTF2A1L | TFIIA-alpha and beta-like factor | Q9UNN4 | DisGeNET |
| STON1 | Stonin-1 | Q9Y6Q2 | DisGeNET |
| H4-16 | Not Available | Not Available | DisGeNET |
| CYP2B6 | Cytochrome P450 2B6 | P20813 | DisGeNET,GeneCard |
| DNMT3A | DNA | Q9Y6K1 | OMIM,GeneCard |
| DEFB1 | Beta-defensin 1 | P60022 | DisGeNET |
| AFM | Afamin | P43652 | DisGeNET |
| E2F1 | Transcription factor E2F1 | Q01094 | DisGeNET |
| EIF4E | Eukaryotic translation initiation factor 4E | P06730 | OMIM,GeneCard |
| ID1 | DNA-binding protein inhibitor ID-1 | P41134 | DisGeNET |
| IFI16 | Gamma-interferon-inducible protein 16 | Q16666 | DisGeNET |
| GDE1 | Glycerophosphodiester phosphodiesterase 1 | Q9NZC3 | DisGeNET |
| PIK3CB | Phosphatidylinositol 4,5-bisphosphate 3-kinase catalytic subunit beta isoform | P42338 | DisGeNET |
| PIK3CD | Phosphatidylinositol 4,5-bisphosphate 3-kinase catalytic subunit delta isoform | O00329 | DisGeNET |
| F2 | Prothrombin | P00734 | DisGeNET,GeneCard |
| IL17D | Interleukin-17D | Q8TAD2 | DisGeNET |
| CCHCR1 | Coiled-coil alpha-helical rod protein 1 | Q8TD31 | DisGeNET |
| FCGR2A | Low affinity immunoglobulin gamma Fc region receptor II-a | P12318 | OMIM,GeneCard |
| H4C15 | Not Available | Not Available | DisGeNET |
| MYDGF | Myeloid-derived growth factor | Q969H8 | DisGeNET |
| FGF21 | Fibroblast growth factor 21 | Q9NSA1 | DisGeNET,GeneCard |
| LEF1 | Lymphoid enhancer-binding factor 1 | Q9UJU2 | DisGeNET |
| FOXP3 | Forkhead box protein P3 | Q9BZS1 | DisGeNET,GeneCard |
| KLRC1 | NKG2-A/NKG2-B type II integral membrane protein | P26715 | DisGeNET |
| LAMC2 | Laminin subunit gamma-2 | Q13753 | DisGeNET |
| GATA2 | Endothelial transcription factor GATA-2 | P23769 | OMIM,GeneCard |
| MRC1 | Macrophage mannose receptor 1 | P22897 | DisGeNET |
| MIR328 | Not Available | Not Available | DisGeNET |
| MIR374A | Not Available | Not Available | DisGeNET |
| GJA1 | Gap junction alpha-1 protein | P17302 | OMIM,GeneCard |
| ACKR3 | Atypical chemokine receptor 3 | P25106 | DisGeNET |
| EKVP3 | Not Available | Not Available | OMIM |
| CMDR | Not Available | Not Available | OMIM |
| HLHS1 | Not Available | Not Available | OMIM |
| AVSD3 | Not Available | Not Available | OMIM |
| CMM9 | Not Available | Not Available | OMIM |
| SALMY | Not Available | Not Available | OMIM |
| HSS | Not Available | Not Available | OMIM |
| PFBMFT1 | Not Available | Not Available | OMIM |
| HMERF | Not Available | Not Available | OMIM |
| BFNIS | Not Available | Not Available | OMIM |
| CILD37 | Not Available | Not Available | OMIM |
| KOGS | Not Available | Not Available | OMIM |
| SCA43 | Not Available | Not Available | OMIM |
| BFIS3 | Not Available | Not Available | OMIM |
| SPGF18 | Not Available | Not Available | OMIM |
| MPRM | Not Available | Not Available | OMIM |
| ODOD | Not Available | Not Available | OMIM |
| PENTT | Not Available | Not Available | OMIM |
| DKCB4 | Not Available | Not Available | OMIM |
| CMT2T | Not Available | Not Available | OMIM |
| ASD8 | Not Available | Not Available | OMIM |
| DEL1q21.1 | Not Available | Not Available | OMIM |
| EKNS | Not Available | Not Available | OMIM |
| EPM4 | Not Available | Not Available | OMIM |
| LKENP | Not Available | Not Available | OMIM |
| MC1DN10 | Not Available | Not Available | OMIM |
| PGL5 | Not Available | Not Available | OMIM |
| POF3 | Not Available | Not Available | OMIM |
| TASR2 | Not Available | Not Available | OMIM |
| VSD2 | Not Available | Not Available | OMIM |
| EIEE11 | Not Available | Not Available | OMIM |
| NEP | Not Available | Not Available | OMIM |
| C1DELq21.1 | Not Available | Not Available | OMIM |
| DKCA2 | Not Available | Not Available | OMIM |
| PFE | Not Available | Not Available | OMIM |
| AMRF | Not Available | Not Available | OMIM |
| HSF | Not Available | Not Available | OMIM |
| COXPD8 | Not Available | Not Available | OMIM |
| B17.2L | Not Available | Not Available | OMIM |
| LGMDR10 | Not Available | Not Available | OMIM |
| SDTY3 | Not Available | Not Available | OMIM |
| IMF1 | Not Available | Not Available | OMIM |
| CMD1GG | Not Available | Not Available | OMIM |
| PFRK | Not Available | Not Available | OMIM |
| HDHC7 | Not Available | Not Available | OMIM |
| TASR1 | Not Available | Not Available | OMIM |
| BMFS4 | Not Available | Not Available | OMIM |
| CAID | Not Available | Not Available | OMIM |
| IIAE3 | Not Available | Not Available | OMIM |
| ILFS1 | Not Available | Not Available | OMIM |
| ILFS2 | Not Available | Not Available | OMIM |
| IMD21 | Not Available | Not Available | OMIM |
| MKS7 | Not Available | Not Available | OMIM |
| MMIHS | Not Available | Not Available | OMIM |
| NKEFA | Not Available | Not Available | OMIM |
| OCP2 | Not Available | Not Available | OMIM |
| PARP | Not Available | Not Available | OMIM |
| PKD4 | Not Available | Not Available | OMIM |
| PSN1 | Not Available | Not Available | OMIM |
| SPGF43 | Not Available | Not Available | OMIM |
| VODI | Not Available | Not Available | OMIM |
| SCARB2 | Lysosome membrane protein 2 | Q14108 | OMIM |
| NDUFAF2 | NADH dehydrogenase [ubiquinone] 1 alpha subcomplex assembly factor 2 | Q8N183 | OMIM |
| MYSM1 | Histone H2A deubiquitinase MYSM1 | Q5VVJ2 | OMIM |
| RBM8A | RNA-binding protein 8A | Q9Y5S9 | OMIM |
| SGOL1 | Not Available | Not Available | OMIM |
| SDHA | Succinate dehydrogenase [ubiquinone] flavoprotein subunit, mitochondrial | P31040 | OMIM |
| AARS2 | Alanine--tRNA ligase, mitochondrial | Q5JTZ9 | OMIM |
| SCN2A | Sodium channel protein type 2 subunit alpha | Q99250 | OMIM |
| DNAH1 | Dynein heavy chain 1, axonemal | Q9P2D7 | OMIM |
| RANBP2 | E3 SUMO-protein ligase RanBP2 | P49792 | OMIM |
| LARS1 | Leucine--tRNA ligase, cytoplasmic | Q9P2J5 | OMIM |
| NBAS | Neuroblastoma-amplified sequence | A2RRP1 | OMIM |
| GOLM1 | Golgi membrane protein 1 | Q8NBJ4 | DisGeNET,GeneCard |
| GPT | Alanine aminotransferase 1 | P24298 | DisGeNET,GeneCard |
| GSTP1 | Glutathione S-transferase P | P09211 | DisGeNET,GeneCard |
| PRDX1 | Peroxiredoxin-1 | Q06830 | OMIM |
| SKP1 | S-phase kinase-associated protein 1 | P63208 | OMIM |
| HADHA | Trifunctional enzyme subunit alpha, mitochondrial | P40939 | OMIM,GeneCard |
| PTH1R | Parathyroid hormone/parathyroid hormone-related peptide receptor | Q03431 | OMIM |
| FOXL2 | Forkhead box protein L2 | P58012 | OMIM |
| FCYT | Not Available | Not Available | OMIM |
| NT5C3A | Cytosolic 5'-nucleotidase 3A | Q9H0P0 | OMIM |
| SPEF2 | Sperm flagellar protein 2 | Q9C093 | OMIM |
| SRSF10 | Serine/arginine-rich splicing factor 10 | O75494 | OMIM |
| HAMP | Hepcidin | P81172 | OMIM |
| CITED2 | Cbp/p300-interacting transactivator 2 | Q99967 | OMIM |
| ACSL6 | Long-chain-fatty-acid--CoA ligase 6 | Q9UKU0 | OMIM |
| AKR7A2 | Aflatoxin B1 aldehyde reductase member 2 | O43488 | OMIM |
| TARDBP | TAR DNA-binding protein 43 | Q13148 | OMIM |
| STRADB | STE20-related kinase adapter protein beta | Q9C0K7 | OMIM |
| DNAJC21 | DnaJ homolog subfamily C member 21 | Q5F1R6 | OMIM |
| CYP21A2 | Steroid 21-hydroxylase | P08686 | OMIM |
| SLC25A20 | Mitochondrial carnitine/acylcarnitine carrier protein | O43772 | OMIM |
| HAVCR2 | Hepatitis A virus cellular receptor 2 | Q8TDQ0 | DisGeNET,GeneCard |
| SLC9A3 | Sodium/hydrogen exchanger 3 | P48764 | OMIM |
| DRD5 | D(1B) dopamine receptor | P21918 | OMIM |
| MAD2L2 | Mitotic spindle assembly checkpoint protein MAD2B | Q9UI95 | OMIM |
| GNAI2 | Guanine nucleotide-binding protein G | P04899 | OMIM |
| GINGF2 | Not Available | Not Available | OMIM |
| HIF1A | Hypoxia-inducible factor 1-alpha | Q16665 | DisGeNET,GeneCard |
| IHPK3 | Not Available | Not Available | OMIM |
| IMPDH1 | Inosine-5'-monophosphate dehydrogenase 1 | P20839 | OMIM |
| CEP19 | Centrosomal protein of 19 kDa | Q96LK0 | OMIM |
| HLA-B | HLA class I histocompatibility antigen, B alpha chain | P01889 | OMIM,GeneCard |
| RBM15 | RNA-binding protein 15 | Q96T37 | OMIM |
| HFM1 | Probable ATP-dependent DNA helicase HFM1 | A2PYH4 | OMIM |
| TRNAM2 | Not Available | Not Available | OMIM |
| TRNT1 | CCA tRNA nucleotidyltransferase 1, mitochondrial | Q96Q11 | OMIM |
| WASF2 | Wiskott-Aldrich syndrome protein family member 2 | Q9Y6W5 | OMIM |
| PPA2 | Inorganic pyrophosphatase 2, mitochondrial | Q9H2U2 | OMIM |
| TAL1 | T-cell acute lymphocytic leukemia protein 1 | P17542 | OMIM |
| GFI1 | Zinc finger protein Gfi-1 | Q99684 | OMIM |
| CFAP44 | Cilia- and flagella-associated protein 44 | Q96MT7 | OMIM |
| CFAP69 | Cilia- and flagella-associated protein 69 | A5D8W1 | OMIM |
| SLC26A8 | Testis anion transporter 1 | Q96RN1 | OMIM |
| TTC21A | Tetratricopeptide repeat protein 21A | Q8NDW8 | OMIM |
| CFAP65 | Cilia- and flagella-associated protein 65 | Q6ZU64 | OMIM |
| DAZL | Deleted in azoospermia-like | Q92904 | OMIM |
| PROS1 | Vitamin K-dependent protein S | P07225 | OMIM |
| EIF3I | Eukaryotic translation initiation factor 3 subunit I | Q13347 | OMIM |
| ADRA2C | Alpha-2C adrenergic receptor | P18825 | OMIM |
| RFH1 | Not Available | Not Available | OMIM |
| SPGF2 | Not Available | Not Available | OMIM |
| DBT | Lipoamide acyltransferase component of branched-chain alpha-keto acid dehydrogenase complex, mitochondrial | P11182 | OMIM |
| HLA-DPB1 | HLA class II histocompatibility antigen, DP beta 1 chain | P04440 | OMIM,GeneCard |
| MDM4 | Protein Mdm4 | O15151 | OMIM |
| CHIC2 | Cysteine-rich hydrophobic domain-containing protein 2 | Q9UKJ5 | OMIM |
| PBX1 | Pre-B-cell leukemia transcription factor 1 | P40424 | OMIM |
| S100A10 | Protein S100-A10 | P60903 | OMIM |
| CD8B1 | Not Available | Not Available | OMIM |
| CDH12 | Cadherin-12 | P55289 | OMIM |
| CENPC1 | Not Available | Not Available | OMIM |
| CTRC | Chymotrypsin-C | Q99895 | OMIM |
| RFWD2 | Not Available | Not Available | OMIM |
| DEK | Protein DEK | P35659 | OMIM |
| SGCE | Epsilon-sarcoglycan | O43556 | OMIM |
| EEF1B2 | Elongation factor 1-beta | P24534 | OMIM |
| FSHD1 | Not Available | Not Available | OMIM |
| GPX1 | Glutathione peroxidase 1 | P07203 | OMIM |
| HIST1H3J | Not Available | Not Available | OMIM |
| HIST1H3H | Not Available | Not Available | OMIM |
| HIST1H3B | Not Available | Not Available | OMIM |
| H4FN | Not Available | Not Available | OMIM |
| IMMT | MICOS complex subunit MIC60 | Q16891 | OMIM |
| RINT1 | RAD50-interacting protein 1 | Q6NUQ1 | OMIM |
| PPP1R2 | Protein phosphatase inhibitor 2 | P41236 | OMIM |
| NBPF1 | Neuroblastoma breakpoint family member 1 | Q3BBV0 | OMIM |
| RAP1A | Ras-related protein Rap-1A | P62834 | OMIM |
| HLA-DRB1 | HLA class II histocompatibility antigen, DRB1 beta chain | P01911 | DisGeNET,GeneCard |
| NDUFB3 | NADH dehydrogenase [ubiquinone] 1 beta subcomplex subunit 3 | O43676 | OMIM |
| MIR101-1 | Not Available | Not Available | OMIM |
| GNB1 | Guanine nucleotide-binding protein G | P62873 | OMIM |
| MRPS18B | 28S ribosomal protein S18b, mitochondrial | Q9Y676 | OMIM |
| ABCB10 | ATP-binding cassette sub-family B member 10, mitochondrial | Q9NRK6 | OMIM |
| HMGB1 | High mobility group protein B1 | P09429 | DisGeNET,GeneCard |
| RNASEH1 | Ribonuclease H1 | O60930 | OMIM |
| TREM2 | Triggering receptor expressed on myeloid cells 2 | Q9NZC2 | OMIM |
| MSH5 | MutS protein homolog 5 | O43196 | OMIM |
| GDF9 | Growth/differentiation factor 9 | O60383 | OMIM |
| NOBOX | Homeobox protein NOBOX | O60393 | OMIM |
| FIGLA | Factor in the germline alpha | Q6QHK4 | OMIM |
| STAG3 | Cohesin subunit SA-3 | Q9UJ98 | OMIM |
| NEK2 | Serine/threonine-protein kinase Nek2 | P51955 | OMIM |
| SHISA5 | Protein shisa-5 | Q8N114 | OMIM |
| BRDT | Bromodomain testis-specific protein | Q58F21 | OMIM |
| TEX15 | Testis-expressed protein 15 | Q9BXT5 | OMIM |
| TSGA10 | Testis-specific gene 10 protein | Q9BZW7 | OMIM |
| SPINK2 | Serine protease inhibitor Kazal-type 2 | P20155 | OMIM |
| FSIP2 | Fibrous sheath-interacting protein 2 | Q5CZC0 | OMIM |
| ARMC2 | Armadillo repeat-containing protein 2 | Q8NEN0 | OMIM |
| TTC29 | Tetratricopeptide repeat protein 29 | Q8NA56 | OMIM |
| SPATA16 | Spermatogenesis-associated protein 16 | Q9BXB7 | OMIM |
| SRM | Spermidine synthase | P19623 | OMIM |
| TSSK1 | Not Available | Not Available | OMIM |
| C1D | Nuclear nucleic acid-binding protein C1D | Q13901 | OMIM |
| AOMS1 | Not Available | Not Available | OMIM |
| TREML1 | Trem-like transcript 1 protein | Q86YW5 | OMIM |
| TREML2 | Trem-like transcript 2 protein | Q5T2D2 | OMIM |
| TREML3 | Not Available | Not Available | OMIM |
| TREML4 | Trem-like transcript 4 protein | Q6UXN2 | OMIM |
| PRSS2 | Trypsin-2 | P07478 | OMIM |
| ACAT2 | Acetyl-CoA acetyltransferase, cytosolic | Q9BWD1 | OMIM |
| AIR | Not Available | Not Available | OMIM |
| ALL2 | Not Available | Not Available | OMIM |
| AMD1 | S-adenosylmethionine decarboxylase proenzyme | P17707 | OMIM |
| ARGFX | Arginine-fifty homeobox | A6NJG6 | OMIM |
| BLACE | B-cell acute lymphoblastic leukemia-expressed protein | A4D250 | OMIM |
| C7orf13 | Not Available | Not Available | OMIM |
| CCND3 | G1/S-specific cyclin-D3 | P30281 | OMIM |
| CDC20 | Cell division cycle protein 20 homolog | Q12834 | OMIM |
| CDCA8 | Borealin | Q53HL2 | OMIM |
| CDYL | Chromodomain Y-like protein | Q9Y232 | OMIM |
| CLLS4 | Not Available | Not Available | OMIM |
| COPD | Not Available | Not Available | OMIM |
| COX17 | Cytochrome c oxidase copper chaperone | Q14061 | OMIM |
| COX5B | Cytochrome c oxidase subunit 5B, mitochondrial | P10606 | OMIM |
| COX7A2 | Cytochrome c oxidase subunit 7A2, mitochondrial | P14406 | OMIM |
| COX7C | Cytochrome c oxidase subunit 7C, mitochondrial | P15954 | OMIM |
| CTEPH1 | Not Available | Not Available | OMIM |
| DBI | Acyl-CoA-binding protein | P07108 | OMIM |
| FCER1G | High affinity immunoglobulin epsilon receptor subunit gamma | P30273 | OMIM |
| FUCA1 | Tissue alpha-L-fucosidase | P04066 | OMIM |
| GM2A | Ganglioside GM2 activator | P17900 | OMIM |
| GPD2 | Glycerol-3-phosphate dehydrogenase, mitochondrial | P43304 | OMIM |
| GSTM5 | Glutathione S-transferase Mu 5 | P46439 | OMIM |
| IFNG | Interferon gamma | P01579 | DisGeNET,GeneCard |
| LPIN1 | Phosphatidate phosphatase LPIN1 | Q14693 | OMIM |
| LPP | Lipoma-preferred partner | Q93052 | OMIM |
| MIR1258 | Not Available | Not Available | OMIM |
| MPD3 | Not Available | Not Available | OMIM |
| MRPL35 | 39S ribosomal protein L35, mitochondrial | Q9NZE8 | OMIM |
| MRPL53 | 39S ribosomal protein L53, mitochondrial | Q96EL3 | OMIM |
| MRPS10 | 28S ribosomal protein S10, mitochondrial | P82664 | OMIM |
| MYB | Transcriptional activator Myb | P10242 | OMIM |
| IL10 | Interleukin-10 | P22301 | DisGeNET,GeneCard |
| NICN1 | Nicolin-1 | Q9BSH3 | OMIM |
| NPM1 | Nucleophosmin | P06748 | OMIM |
| IL17A | Interleukin-17A | Q16552 | DisGeNET,GeneCard |
| ORMDL1 | ORM1-like protein 1 | Q9P0S3 | OMIM |
| PARK21 | Not Available | Not Available | OMIM |
| PARTICL | Not Available | Not Available | OMIM |
| PGK2 | Phosphoglycerate kinase 2 | P07205 | OMIM |
| PHKG1 | Phosphorylase b kinase gamma catalytic chain, skeletal muscle/heart isoform | Q16816 | OMIM |
| PIGF | Phosphatidylinositol-glycan biosynthesis class F protein | Q07326 | OMIM |
| PPP2CB | Serine/threonine-protein phosphatase 2A catalytic subunit beta isoform | P62714 | OMIM |
| PRKCI | Protein kinase C iota type | P41743 | OMIM |
| RAP1GDS1 | Rap1 GTPase-GDP dissociation stimulator 1 | P52306 | OMIM |
| RPL9 | Not Available | Not Available | OMIM |
| RRM2 | Ribonucleoside-diphosphate reductase subunit M2 | P31350 | OMIM |
| SM2 | Not Available | Not Available | OMIM |
| SRD5A1 | 3-oxo-5-alpha-steroid 4-dehydrogenase 1 | P18405 | OMIM |
| TREM1 | Triggering receptor expressed on myeloid cells 1 | Q9NP99 | OMIM |
| TTC4 | Tetratricopeptide repeat protein 4 | O95801 | OMIM |
| TWISTNB | DNA-directed RNA polymerase I subunit RPA43 | Q3B726 | OMIM |
| TYHAQ | Not Available | Not Available | OMIM |
| UQCRH | Cytochrome b-c1 complex subunit 6, mitochondrial | P07919 | OMIM |
| VAMAS6 | Not Available | Not Available | OMIM |
| WAKMAR2 | Not Available | Not Available | OMIM |
| ZNF92 | Zinc finger protein 92 | Q03936 | OMIM |
| 2ADUB | Not Available | Not Available | OMIM |
| ADPRT | Not Available | Not Available | OMIM |
| ADRA2L2 | Not Available | Not Available | OMIM |
| AKR7 | Not Available | Not Available | OMIM |
| AORF | Not Available | Not Available | OMIM |
| ASG | Not Available | Not Available | OMIM |
| BCATE2 | Not Available | Not Available | OMIM |
| BMFS1 | Not Available | Not Available | OMIM |
| BMFS6 | Not Available | Not Available | OMIM |
| BPES | Not Available | Not Available | OMIM |
| BTL | Not Available | Not Available | OMIM |
| C3orf34 | Not Available | Not Available | OMIM |
| C7orf63 | Not Available | Not Available | OMIM |
| CACT | Not Available | Not Available | OMIM |
| CAKUHED | Not Available | Not Available | OMIM |
| CAL1L | Not Available | Not Available | OMIM |
| CCDC108 | Not Available | Not Available | OMIM |
| CD10 | Not Available | Not Available | OMIM |
| CD36L2 | Not Available | Not Available | OMIM |
| CD8B | T-cell surface glycoprotein CD8 beta chain | P10966 | OMIM |
| CDHB | Not Available | Not Available | OMIM |
| CENPC | Centromere protein C | Q03188 | OMIM |
| CLCR | Not Available | Not Available | OMIM |
| CMD1G | Not Available | Not Available | OMIM |
| COP1 | E3 ubiquitin-protein ligase COP1 | Q8NHY2 | OMIM |
| CX43 | Not Available | Not Available | OMIM |
| CYP21 | Not Available | Not Available | OMIM |
| D6S231E | Not Available | Not Available | OMIM |
| DAZH | Not Available | Not Available | OMIM |
| DCML | Not Available | Not Available | OMIM |
| DFNB39 | Not Available | Not Available | OMIM |
| DNAJA5 | Not Available | Not Available | OMIM |
| DRD1B | Not Available | Not Available | OMIM |
| DYT11 | Not Available | Not Available | OMIM |
| EEF1B1 | Not Available | Not Available | OMIM |
| EIF3S2 | Not Available | Not Available | OMIM |
| EIF4EL1 | Not Available | Not Available | OMIM |
| EZH1 | Histone-lysine N-methyltransferase EZH1 | Q92800 | OMIM |
| FACL6 | Not Available | Not Available | OMIM |
| FSHD1A | Not Available | Not Available | OMIM |
| FUSIP1 | Not Available | Not Available | OMIM |
| GGF2 | Not Available | Not Available | OMIM |
| GLNS | Not Available | Not Available | OMIM |
| GNAI2B | Not Available | Not Available | OMIM |
| GPXD | Not Available | Not Available | OMIM |
| H3FJ | Not Available | Not Available | OMIM |
| H3FK | Not Available | Not Available | OMIM |
| H3FL | Not Available | Not Available | OMIM |
| H4F2 | Not Available | Not Available | OMIM |
| HL11 | Not Available | Not Available | OMIM |
| HMP | Not Available | Not Available | OMIM |
| IFI41 | Not Available | Not Available | OMIM |
| IFNB2 | Not Available | Not Available | OMIM |
| IGFR2 | Not Available | Not Available | OMIM |
| IIAE2 | Not Available | Not Available | OMIM |
| IIAE4 | Not Available | Not Available | OMIM |
| IL6RQ | Not Available | Not Available | OMIM |
| ILFS3 | Not Available | Not Available | OMIM |
| ILPIP | Not Available | Not Available | OMIM |
| INSP6K3 | Not Available | Not Available | OMIM |
| IPP2 | Not Available | Not Available | OMIM |
| KIAA1270 | Not Available | Not Available | OMIM |
| KIAA1693 | Not Available | Not Available | OMIM |
| KPL2 | Not Available | Not Available | OMIM |
| KREV1 | Not Available | Not Available | OMIM |
| LARS | Not Available | Not Available | OMIM |
| MAD2B | Not Available | Not Available | OMIM |
| MAR | Not Available | Not Available | OMIM |
| MC1DN25 | Not Available | Not Available | OMIM |
| MER3 | Not Available | Not Available | OMIM |
| MIRN101-1 | Not Available | Not Available | OMIM |
| MLCK | Not Available | Not Available | OMIM |
| MRD42 | Not Available | Not Available | OMIM |
| MRG1 | Not Available | Not Available | OMIM |
| MRPS18-2 | Not Available | Not Available | OMIM |
| MTABC2 | Not Available | Not Available | OMIM |
| MTPA | Not Available | Not Available | OMIM |
| NAG | Not Available | Not Available | OMIM |
| NDUFA12L | Not Available | Not Available | OMIM |
| NHE3 | Not Available | Not Available | OMIM |
| NPH3 | Not Available | Not Available | OMIM |
| NS7 | Not Available | Not Available | OMIM |
| NT5C3 | Not Available | Not Available | OMIM |
| NUP358 | Not Available | Not Available | OMIM |
| ON | Not Available | Not Available | OMIM |
| OTF3 | Not Available | Not Available | OMIM |
| PBT | Not Available | Not Available | OMIM |
| PDGFR | Not Available | Not Available | OMIM |
| PEOB2 | Not Available | Not Available | OMIM |
| IL18 | Interleukin-18 | Q14116 | DisGeNET,GeneCard |
| PLOSL2 | Not Available | Not Available | OMIM |
| POF13 | Not Available | Not Available | OMIM |
| POF14 | Not Available | Not Available | OMIM |
| POF5 | Not Available | Not Available | OMIM |
| POF6 | Not Available | Not Available | OMIM |
| POF8 | Not Available | Not Available | OMIM |
| PRXI | Not Available | Not Available | OMIM |
| PTHR1 | Not Available | Not Available | OMIM |
| RBM8B | Not Available | Not Available | OMIM |
| RP10 | Not Available | Not Available | OMIM |
| RP67 | Not Available | Not Available | OMIM |
| SCFI | Not Available | Not Available | OMIM |
| SCN2A1 | Not Available | Not Available | OMIM |
| SCOTIN | Not Available | Not Available | OMIM |
| SDH1 | Not Available | Not Available | OMIM |
| SGO | Not Available | Not Available | OMIM |
| SIFD | Not Available | Not Available | OMIM |
| SKP1A | Not Available | Not Available | OMIM |
| SPDA1 | Not Available | Not Available | OMIM |
| SPEN | Msx2-interacting protein | Q96T58 | OMIM |
| SPGF21 | Not Available | Not Available | OMIM |
| SPGF25 | Not Available | Not Available | OMIM |
| SPGF26 | Not Available | Not Available | OMIM |
| SPGF29 | Not Available | Not Available | OMIM |
| SPGF34 | Not Available | Not Available | OMIM |
| SPGF38 | Not Available | Not Available | OMIM |
| SPGF42 | Not Available | Not Available | OMIM |
| SPGF6 | Not Available | Not Available | OMIM |
| SPS1 | Not Available | Not Available | OMIM |
| STI2 | Not Available | Not Available | OMIM |
| STK22D | Not Available | Not Available | OMIM |
| SUNCOR | Not Available | Not Available | OMIM |
| SYNX | Not Available | Not Available | OMIM |
| TAT1 | Not Available | Not Available | OMIM |
| TBRS | Not Available | Not Available | OMIM |
| TCL5 | Not Available | Not Available | OMIM |
| TCS1 | Not Available | Not Available | OMIM |
| TDP43 | Not Available | Not Available | OMIM |
| THPH5 | Not Available | Not Available | OMIM |
| TLT1 | Not Available | Not Available | OMIM |
| TLT2 | Not Available | Not Available | OMIM |
| TLT3 | Not Available | Not Available | OMIM |
| TLT4 | Not Available | Not Available | OMIM |
| TRNAMI2 | Not Available | Not Available | OMIM |
| TRY2 | Not Available | Not Available | OMIM |
| WAVE2 | Not Available | Not Available | OMIM |
| WDR52 | Not Available | Not Available | OMIM |
| ZNF163 | Not Available | Not Available | OMIM |
| AAT7 | Not Available | Not Available | OMIM |
| ACS2 | Not Available | Not Available | OMIM |
| AFAR | Not Available | Not Available | OMIM |
| ALS10 | Not Available | Not Available | OMIM |
| ALS2CR2 | Not Available | Not Available | OMIM |
| ANE1 | Not Available | Not Available | OMIM |
| ARPKD | Not Available | Not Available | OMIM |
| AUTS19 | Not Available | Not Available | OMIM |
| BFIC3 | Not Available | Not Available | OMIM |
| BMFS3 | Not Available | Not Available | OMIM |
| BPES1 | Not Available | Not Available | OMIM |
| BSF2 | Not Available | Not Available | OMIM |
| CA21H | Not Available | Not Available | OMIM |
| CAC | Not Available | Not Available | OMIM |
| CALLA | Not Available | Not Available | OMIM |
| CD32 | Not Available | Not Available | OMIM |
| DIAR8 | Not Available | Not Available | OMIM |
| DNAHC1 | Not Available | Not Available | OMIM |
| DRD1L2 | Not Available | Not Available | OMIM |
| EST2 | Not Available | Not Available | OMIM |
| FANCV | Not Available | Not Available | OMIM |
| GIP | Gastric inhibitory polypeptide | P09681 | OMIM |
| HESJAS | Not Available | Not Available | OMIM |
| HGF2 | Not Available | Not Available | OMIM |
| IBGC4 | Not Available | Not Available | OMIM |
| IFI75 | Not Available | Not Available | OMIM |
| IL6Q | Not Available | Not Available | OMIM |
| IP6K3 | Inositol hexakisphosphate kinase 3 | Q96PC2 | OMIM |
| KIAA1770 | Not Available | Not Available | OMIM |
| KIAA1915 | Not Available | Not Available | OMIM |
| LCA11 | Not Available | Not Available | OMIM |
| LFIS | Not Available | Not Available | OMIM |
| LIMPII | Not Available | Not Available | OMIM |
| MASTC | Not Available | Not Available | OMIM |
| MMTN | Not Available | Not Available | OMIM |
| MONOMAC | Not Available | Not Available | OMIM |
| MOSPGF | Not Available | Not Available | OMIM |
| MTALARS | Not Available | Not Available | OMIM |
| ODDD | Not Available | Not Available | OMIM |
| OI17 | Not Available | Not Available | OMIM |
| OTT | Not Available | Not Available | OMIM |
| P35SRJ | Not Available | Not Available | OMIM |
| PAGA | Not Available | Not Available | OMIM |
| POF9 | Not Available | Not Available | OMIM |
| PPOL | Not Available | Not Available | OMIM |
| PTHR | Not Available | Not Available | OMIM |
| RHPD1 | Not Available | Not Available | OMIM |
| RNTMI2 | Not Available | Not Available | OMIM |
| RPEM | Not Available | Not Available | OMIM |
| SCAR2 | Not Available | Not Available | OMIM |
| SCFAI | Not Available | Not Available | OMIM |
| SCL | Not Available | Not Available | OMIM |
| SCN2 | Not Available | Not Available | OMIM |
| SDHF | Not Available | Not Available | OMIM |
| SGO1 | Shugoshin 1 | Q5FBB7 | OMIM |
| SOPH | Not Available | Not Available | OMIM |
| SPGF20 | Not Available | Not Available | OMIM |
| SPGF24 | Not Available | Not Available | OMIM |
| SPGF3 | Not Available | Not Available | OMIM |
| SPGF37 | Not Available | Not Available | OMIM |
| SPGF40 | Not Available | Not Available | OMIM |
| SPGYLA | Not Available | Not Available | OMIM |
| TAR | Not Available | Not Available | OMIM |
| TASR | Not Available | Not Available | OMIM |
| TCEB1L | Not Available | Not Available | OMIM |
| THPH6 | Not Available | Not Available | OMIM |
| TMD | Not Available | Not Available | OMIM |
| TRIP1 | Not Available | Not Available | OMIM |
| UMPH1 | Not Available | Not Available | OMIM |
| WVS | Not Available | Not Available | OMIM |
| INS | Insulin [Cleaved into: Insulin B chain; Insulin A chain] | P01308 | GeneCard |
| IL1A | Interleukin-1 alpha | P01583 | DisGeNET,GeneCard |
| IL1B | Interleukin-1 beta | P01584 | DisGeNET,GeneCard |
| ACE | Angiotensin-converting enzyme | P12821 | GeneCard |
| TP53 | Cellular tumor antigen p53 | P04637 | GeneCard |
| SLC17A5 | Sialin | Q9NRA2 | GeneCard |
| TGFB1 | Transforming growth factor beta-1 proprotein [Cleaved into: Latency-associated peptide | P01137 | GeneCard |
| CD40LG | CD40 ligand | P29965 | GeneCard |
| SERPINA1 | Alpha-1-antitrypsin | P01009 | GeneCard |
| HFE | Hereditary hemochromatosis protein | Q30201 | GeneCard |
| FAS | Tumor necrosis factor receptor superfamily member 6 | P25445 | GeneCard |
| CFTR | Cystic fibrosis transmembrane conductance regulator | P13569 | GeneCard |
| IL1RN | Interleukin-1 receptor antagonist protein | P18510 | DisGeNET,GeneCard |
| LEP | Leptin | P41159 | GeneCard |
| REN | Renin | P00797 | GeneCard |
| PPARG | Peroxisome proliferator-activated receptor gamma | P37231 | GeneCard |
| NPPA | Natriuretic peptides A | P01160 | GeneCard |
| CP | Ceruloplasmin | P00450 | GeneCard |
| CCL2 | C-C motif chemokine 2 | P13500 | GeneCard |
| IL2 | Interleukin-2 | P60568 | DisGeNET,GeneCard |
| CTLA4 | Cytotoxic T-lymphocyte protein 4 | P16410 | GeneCard |
| SOD1 | Superoxide dismutase [Cu-Zn] | P00441 | GeneCard |
| TTR | Transthyretin | P02766 | GeneCard |
| IL21 | Interleukin-21 | Q9HBE4 | DisGeNET,GeneCard |
| ICAM1 | Intercellular adhesion molecule 1 | P05362 | GeneCard |
| IL33 | Interleukin-33 | O95760 | DisGeNET,GeneCard |
| TF | Serotransferrin | P02787 | GeneCard |
| STAT3 | Signal transducer and activator of transcription 3 | P40763 | GeneCard |
| IL6R | Interleukin-6 receptor subunit alpha | P08887 | OMIM,GeneCard |
| EGF | Pro-epidermal growth factor | P01133 | GeneCard |
| PKD1 | Polycystin-1 | P98161 | GeneCard |
| THBD | Thrombomodulin | P07204 | GeneCard |
| IRF1 | Interferon regulatory factor 1 | P10914 | OMIM,GeneCard |
| EGFR | Epidermal growth factor receptor | P00533 | GeneCard |
| FASLG | Tumor necrosis factor ligand superfamily member 6 | P48023 | GeneCard |
| NOS2 | Nitric oxide synthase, inducible | P35228 | GeneCard |
| IGF1 | Insulin-like growth factor I | P05019 | GeneCard |
| JAK1 | Tyrosine-protein kinase JAK1 | P23458 | OMIM,GeneCard |
| MYC | Myc proto-oncogene protein | P01106 | GeneCard |
| GGT1 | Glutathione hydrolase 1 proenzyme | P19440 | GeneCard |
| EPO | Erythropoietin | P01588 | GeneCard |
| STAT1 | Signal transducer and activator of transcription 1-alpha/beta | P42224 | GeneCard |
| NPPB | Natriuretic peptides B | P16860 | GeneCard |
| CDH1 | Cadherin-1 | P12830 | GeneCard |
| CD79A | B-cell antigen receptor complex-associated protein alpha chain | P11912 | GeneCard |
| KRAS | GTPase KRas | P01116 | GeneCard |
| NOS3 | Nitric oxide synthase, endothelial | P29474 | GeneCard |
| POMC | Pro-opiomelanocortin | P01189 | GeneCard |
| KIT | Mast/stem cell growth factor receptor Kit | P10721 | OMIM,GeneCard |
| FBN1 | Fibrillin-1 [Cleaved into: Asprosin] | P35555 | GeneCard |
| VEGFA | Vascular endothelial growth factor A | P15692 | GeneCard |
| FN1 | Fibronectin | P02751 | GeneCard |
| MBL2 | Mannose-binding protein C | P11226 | GeneCard |
| ACADVL | Very long-chain specific acyl-CoA dehydrogenase, mitochondrial | P49748 | GeneCard |
| WT1 | Wilms tumor protein | P19544 | GeneCard |
| CAT | Catalase | P04040 | GeneCard |
| TMEM67 | Meckelin | Q5HYA8 | GeneCard |
| HLA-DQB1 | HLA class II histocompatibility antigen, DQ beta 1 chain | P01920 | GeneCard |
| MUC1 | Mucin-1 | P15941 | GeneCard |
| AGTR1 | Type-1 angiotensin II receptor | P30556 | GeneCard |
| MIR21 | Not Available | Not Available | GeneCard |
| PKD2 | Polycystin-2 | Q13563 | GeneCard |
| KLRK1 | NKG2-D type II integral membrane protein | P26718 | DisGeNET,GeneCard |
| LPL | Lipoprotein lipase | P06858 | GeneCard |
| SEC63 | Translocation protein SEC63 homolog | Q9UGP8 | GeneCard |
| VDR | Vitamin D3 receptor | P11473 | GeneCard |
| THPO | Thrombopoietin | P40225 | GeneCard |
| TFRC | Transferrin receptor protein 1 | P02786 | GeneCard |
| HADHB | Trifunctional enzyme subunit beta, mitochondrial | P55084 | GeneCard |
| EDN1 | Endothelin-1 | P05305 | GeneCard |
| KRT18 | Keratin, type I cytoskeletal 18 | P05783 | DisGeNET,GeneCard |
| SMAD4 | Mothers against decapentaplegic homolog 4 | Q13485 | GeneCard |
| CDKN2A | Tumor suppressor ARF | Q8N726 | GeneCard |
| HLA-A | HLA class I histocompatibility antigen, A alpha chain | P04439 | GeneCard |
| BMP6 | Bone morphogenetic protein 6 | P22004 | GeneCard |
| DMD | Dystrophin | P11532 | GeneCard |
| PRKCSH | Glucosidase 2 subunit beta | P14314 | GeneCard |
| GAA | Lysosomal alpha-glucosidase | P10253 | GeneCard |
| NOTCH1 | Neurogenic locus notch homolog protein 1 | P46531 | GeneCard |
| LCN2 | Neutrophil gelatinase-associated lipocalin | P80188 | DisGeNET,GeneCard |
| ADIPOQ | Adiponectin | Q15848 | GeneCard |
| TERC | Not Available | Not Available | GeneCard |
| B2M | Beta-2-microglobulin [Cleaved into: Beta-2-microglobulin form pI 5.3] | P61769 | GeneCard |
| CASP8 | Caspase-8 | Q14790 | GeneCard |
| MFN2 | Mitofusin-2 | O95140 | DisGeNET,GeneCard |
| RTEL1 | Regulator of telomere elongation helicase 1 | Q9NZ71 | GeneCard |
| ERCC6 | Chimeric ERCC6-PGBD3 protein | P0DP91 | GeneCard |
| INVS | Inversin | Q9Y283 | GeneCard |
| CYP3A4 | Cytochrome P450 3A4 | P08684 | GeneCard |
| CCN2 | CCN family member 2 | P29279 | GeneCard |
| IL4 | Interleukin-4 | P05112 | GeneCard |
| HNF1B | Hepatocyte nuclear factor 1-beta | P35680 | GeneCard |
| CCR5 | C-C chemokine receptor type 5 | P51681 | GeneCard |
| AKT1 | RAC-alpha serine/threonine-protein kinase | P31749 | GeneCard |
| TGFBR2 | TGF-beta receptor type-2 | P37173 | GeneCard |
| ARG1 | Arginase-1 | P05089 | GeneCard |
| APOB | Apolipoprotein B-100 | P04114 | GeneCard |
| TNFRSF1B | Tumor necrosis factor receptor superfamily member 1B | P20333 | GeneCard |
| HRAS | GTPase HRas | P01112 | GeneCard |
| PON1 | Serum paraoxonase/arylesterase 1 | P27169 | GeneCard |
| PPARA | Peroxisome proliferator-activated receptor alpha | Q07869 | GeneCard |
| NEK8 | Serine/threonine-protein kinase Nek8 | Q86SG6 | GeneCard |
| C3 | Complement C3 | P01024 | GeneCard |
| ABCB4 | Phosphatidylcholine translocator ABCB4 | P21439 | GeneCard |
| PDCD1 | Programmed cell death protein 1 | Q15116 | GeneCard |
| TSC2 | Tuberin | P49815 | GeneCard |
| XDH | Xanthine dehydrogenase/oxidase [Includes: Xanthine dehydrogenase | P47989 | GeneCard |
| MECP2 | Methyl-CpG-binding protein 2 | P51608 | GeneCard |
| FGA | Fibrinogen alpha chain [Cleaved into: Fibrinopeptide A; Fibrinogen alpha chain] | P02671 | GeneCard |
| PRTN3 | Myeloblastin | P24158 | GeneCard |
| ADA | Adenosine deaminase | P00813 | GeneCard |
| ALPL | Alkaline phosphatase, tissue-nonspecific isozyme | P05186 | GeneCard |
| NOD2 | Nucleotide-binding oligomerization domain-containing protein 2 | Q9HC29 | GeneCard |
| G6PD | Glucose-6-phosphate 1-dehydrogenase | P11413 | GeneCard |
| APOE | Apolipoprotein E | P02649 | GeneCard |
| BRCA2 | Breast cancer type 2 susceptibility protein | P51587 | GeneCard |
| MPO | Myeloperoxidase | P05164 | GeneCard |
| CD28 | T-cell-specific surface glycoprotein CD28 | P10747 | GeneCard |
| ELANE | Neutrophil elastase | P08246 | GeneCard |
| JAK2 | Tyrosine-protein kinase JAK2 | O60674 | GeneCard |
| UMOD | Uromodulin | P07911 | GeneCard |
| TIMP1 | Metalloproteinase inhibitor 1 | P01033 | GeneCard |
| NLRP3 | NACHT, LRR and PYD domains-containing protein 3 | Q96P20 | GeneCard |
| MYH7 | Myosin-7 | P12883 | GeneCard |
| APOA1 | Apolipoprotein A-I | P02647 | GeneCard |
| SLC4A1 | Band 3 anion transport protein | P02730 | GeneCard |
| CYP2E1 | Cytochrome P450 2E1 | P05181 | GeneCard |
| GATA4 | Transcription factor GATA-4 | P43694 | GeneCard |
| ABCB1 | ATP-dependent translocase ABCB1 | P08183 | GeneCard |
| INPP5E | Phosphatidylinositol polyphosphate 5-phosphatase type IV | Q9NRR6 | GeneCard |
| AQP2 | Aquaporin-2 | P41181 | GeneCard |
| WRAP53 | Telomerase Cajal body protein 1 | Q9BUR4 | GeneCard |
| CD81 | CD81 antigen | P60033 | GeneCard |
| MIR126 | Not Available | Not Available | GeneCard |
| TSC1 | Hamartin | Q92574 | GeneCard |
| F3 | Tissue factor | P13726 | GeneCard |
| IGF2 | Insulin-like growth factor II | P01344 | GeneCard |
| CFH | Complement factor H | P08603 | GeneCard |
| HLA-DQA1 | HLA class II histocompatibility antigen, DQ alpha 1 chain | P01909 | GeneCard |
| ABCC2 | Canalicular multispecific organic anion transporter 1 | Q92887 | GeneCard |
| MIR17 | Not Available | Not Available | GeneCard |
| NRAS | GTPase NRas | P01111 | GeneCard |
| TGFA | Protransforming growth factor alpha [Cleaved into: Transforming growth factor alpha | P01135 | GeneCard |
| CD4 | T-cell surface glycoprotein CD4 | P01730 | GeneCard |
| POLG | DNA polymerase subunit gamma-1 | P54098 | GeneCard |
| MIR122 | Not Available | Not Available | DisGeNET,GeneCard |
| COMT | Catechol O-methyltransferase | P21964 | GeneCard |
| CTNNB1 | Catenin beta-1 | P35222 | GeneCard |
| ATP8B1 | Phospholipid-transporting ATPase IC | O43520 | GeneCard |
| MIR146A | Not Available | Not Available | DisGeNET,GeneCard |
| MIR155 | Not Available | Not Available | GeneCard |
| ALG9 | Alpha-1,2-mannosyltransferase ALG9 | Q9H6U8 | GeneCard |
| NFE2L2 | Nuclear factor erythroid 2-related factor 2 | Q16236 | GeneCard |
| ENPP1 | Ectonucleotide pyrophosphatase/phosphodiesterase family member 1 | P22413 | GeneCard |
| RYR1 | Ryanodine receptor 1 | P21817 | GeneCard |
| PTGS2 | Prostaglandin G/H synthase 2 | P35354 | GeneCard |
| CCL5 | C-C motif chemokine 5 | P13501 | GeneCard |
| FAH | Fumarylacetoacetase | P16930 | GeneCard |
| HP | Haptoglobin | P00738 | GeneCard |
| TINF2 | TERF1-interacting nuclear factor 2 | Q9BSI4 | GeneCard |
| RETN | Resistin | Q9HD89 | GeneCard |
| DKC1 | H/ACA ribonucleoprotein complex subunit DKC1 | O60832 | GeneCard |
| HNF4A | Hepatocyte nuclear factor 4-alpha | P41235 | GeneCard |
| SERPINA3 | Alpha-1-antichymotrypsin | P01011 | GeneCard |
| CASR | Extracellular calcium-sensing receptor | P41180 | GeneCard |
| SERPINC1 | Antithrombin-III | P01008 | GeneCard |
| SERPINE1 | Plasminogen activator inhibitor 1 | P05121 | GeneCard |
| PRF1 | Perforin-1 | P14222 | GeneCard |
| IFNA1 | Not Available | Not Available | GeneCard |
| TNFSF11 | Tumor necrosis factor ligand superfamily member 11 | O14788 | GeneCard |
| SFTPC | Pulmonary surfactant-associated protein C | P11686 | GeneCard |
| MAPK8 | Mitogen-activated protein kinase 8 | P45983 | GeneCard |
| MME | Neprilysin | P08473 | OMIM,GeneCard |
| HNF1A | Hepatocyte nuclear factor 1-alpha | P20823 | GeneCard |
| G6PC | Glucose-6-phosphatase | P35575 | GeneCard |
| HBB | Hemoglobin subunit beta | P68871 | GeneCard |
| MMP2 | 72 kDa type IV collagenase | P08253 | GeneCard |
| TRPV4 | Transient receptor potential cation channel subfamily V member 4 | Q9HBA0 | GeneCard |
| INSR | Insulin receptor | P06213 | GeneCard |
| NHP2 | H/ACA ribonucleoprotein complex subunit 2 | Q9NX24 | GeneCard |
| MAPK1 | Mitogen-activated protein kinase 1 | P28482 | GeneCard |
| F5 | Coagulation factor V | P12259 | GeneCard |
| IL12RB1 | Interleukin-12 receptor subunit beta-1 | P42701 | GeneCard |
| PTEN | Phosphatidylinositol 3,4,5-trisphosphate 3-phosphatase and dual-specificity protein phosphatase PTEN | P60484 | GeneCard |
| ITGB3 | Integrin beta-3 | P05106 | GeneCard |
| PARN | Poly | O95453 | GeneCard |
| TK2 | Thymidine kinase 2, mitochondrial | O00142 | GeneCard |
| HPRT1 | Hypoxanthine-guanine phosphoribosyltransferase | P00492 | GeneCard |
| ATP7B | Copper-transporting ATPase 2 | P35670 | GeneCard |
| MMP9 | Matrix metalloproteinase-9 | P14780 | DisGeNET,GeneCard |
| IL2RA | Interleukin-2 receptor subunit alpha | P01589 | GeneCard |
| DCTN1 | Dynactin subunit 1 | Q14203 | GeneCard |
| MEFV | Pyrin | O15553 | GeneCard |
| HMOX1 | Heme oxygenase 1 | P09601 | GeneCard |
| LMNA | Prelamin-A/C [Cleaved into: Lamin-A/C | P02545 | GeneCard |
| ITGAM | Integrin alpha-M | P11215 | GeneCard |
| MIR34A | Not Available | Not Available | GeneCard |
| TLR5 | Toll-like receptor 5 | O60602 | GeneCard |
| EPHX1 | Epoxide hydrolase 1 | P07099 | GeneCard |
| ESR1 | Estrogen receptor | P03372 | GeneCard |
| TRIP4 | Activating signal cointegrator 1 | Q15650 | GeneCard |
| MTOR | Serine/threonine-protein kinase mTOR | P42345 | DisGeNET,GeneCard |
| SST | Somatostatin | P61278 | GeneCard |
| PMS2 | Mismatch repair endonuclease PMS2 | P54278 | GeneCard |
| CAV1 | Caveolin-1 | Q03135 | GeneCard |
| HBA2 | Not Available | Not Available | GeneCard |
| GNRH1 | Progonadoliberin-1 | P01148 | GeneCard |
| IL5 | Interleukin-5 | P05113 | GeneCard |
| MT-CYB | Cytochrome b | P00156 | GeneCard |
| IBA57 | Putative transferase CAF17, mitochondrial | Q5T440 | GeneCard |
| PPARGC1A | Peroxisome proliferator-activated receptor gamma coactivator 1-alpha | Q9UBK2 | GeneCard |
| CLDN1 | Claudin-1 | O95832 | GeneCard |
| CYP2C9 | Cytochrome P450 2C9 | P11712 | GeneCard |
| NOP10 | H/ACA ribonucleoprotein complex subunit 3 | Q9NPE3 | GeneCard |
| BDNF | Brain-derived neurotrophic factor | P23560 | GeneCard |
| SH2D1A | SH2 domain-containing protein 1A | O60880 | GeneCard |
| NKX2-1 | Homeobox protein Nkx-2.1 | P43699 | GeneCard |
| NFKB1 | Nuclear factor NF-kappa-B p105 subunit | P19838 | GeneCard |
| FKRP | Fukutin-related protein | Q9H9S5 | GeneCard |
| MIR144 | Not Available | Not Available | GeneCard |
| TNFRSF13B | Tumor necrosis factor receptor superfamily member 13B | O14836 | GeneCard |
| MYD88 | Myeloid differentiation primary response protein MyD88 | Q99836 | GeneCard |
| SDHB | Succinate dehydrogenase [ubiquinone] iron-sulfur subunit, mitochondrial | P21912 | GeneCard |
| CFI | Complement factor I | P05156 | GeneCard |
| PRSS1 | Trypsin-1 | P07477 | GeneCard |
| JUN | Transcription factor AP-1 | P05412 | GeneCard |
| SOD2 | Superoxide dismutase [Mn], mitochondrial | P04179 | GeneCard |
| GUSB | Beta-glucuronidase | P08236 | GeneCard |
| GH1 | Somatotropin | P01241 | GeneCard |
| MPL | Thrombopoietin receptor | P40238 | GeneCard |
| PIK3C2A | Phosphatidylinositol 4-phosphate 3-kinase C2 domain-containing subunit alpha | O00443 | GeneCard |
| KRT8 | Keratin, type II cytoskeletal 8 | P05787 | GeneCard |
| VCAM1 | Vascular cell adhesion protein 1 | P19320 | GeneCard |
| MVK | Mevalonate kinase | Q03426 | GeneCard |
| LCAT | Phosphatidylcholine-sterol acyltransferase | P04180 | GeneCard |
| IL12A | Interleukin-12 subunit alpha | P29459 | GeneCard |
| MYLK | Myosin light chain kinase, smooth muscle | Q15746 | OMIM,GeneCard |
| FECH | Ferrochelatase, mitochondrial | P22830 | GeneCard |
| FOS | Proto-oncogene c-Fos | P01100 | GeneCard |
| UGT1A1 | UDP-glucuronosyltransferase 1-1 | P22309 | GeneCard |
| FGF2 | Fibroblast growth factor 2 | P09038 | GeneCard |
| CCND1 | G1/S-specific cyclin-D1 | P24385 | GeneCard |
| TRMT10C | tRNA methyltransferase 10 homolog C | Q7L0Y3 | GeneCard |
| FANCM | Fanconi anemia group M protein | Q8IYD8 | GeneCard |
| CHAT | Choline O-acetyltransferase | P28329 | GeneCard |
| TPI1 | Triosephosphate isomerase | P60174 | GeneCard |
| ENG | Endoglin | P17813 | GeneCard |
| MYPN | Myopalladin | Q86TC9 | GeneCard |
| TCIRG1 | V-type proton ATPase 116 kDa subunit a isoform 3 | Q13488 | GeneCard |
| INPPL1 | Phosphatidylinositol 3,4,5-trisphosphate 5-phosphatase 2 | O15357 | GeneCard |
| MIR148A | Not Available | Not Available | GeneCard |
| IL13 | Interleukin-13 | P35225 | GeneCard |
| CALCA | Calcitonin gene-related peptide 1 | P06881 | GeneCard |
| MUSK | Muscle, skeletal receptor tyrosine-protein kinase | O15146 | GeneCard |
| ADM | ADM [Cleaved into: Adrenomedullin | P35318 | GeneCard |
| IGFBP3 | Insulin-like growth factor-binding protein 3 | P17936 | GeneCard |
| SBDS | Ribosome maturation protein SBDS | Q9Y3A5 | GeneCard |
| NFU1 | NFU1 iron-sulfur cluster scaffold homolog, mitochondrial | Q9UMS0 | GeneCard |
| CA2 | Carbonic anhydrase 2 | P00918 | GeneCard |
| MET | Hepatocyte growth factor receptor | P08581 | GeneCard |
| CD46 | Membrane cofactor protein | P15529 | GeneCard |
| FLT1 | Vascular endothelial growth factor receptor 1 | P17948 | GeneCard |
| NCF1 | Neutrophil cytosol factor 1 | P14598 | OMIM,GeneCard |
| CSF2RA | Granulocyte-macrophage colony-stimulating factor receptor subunit alpha | P15509 | GeneCard |
| SIRT1 | NAD-dependent protein deacetylase sirtuin-1 | Q96EB6 | GeneCard |
| OTC | Ornithine carbamoyltransferase, mitochondrial | P00480 | GeneCard |
| H2AC18 | Not Available | Not Available | GeneCard |
| ACTA1 | Actin, alpha skeletal muscle | P68133 | GeneCard |
| AGXT | Serine--pyruvate aminotransferase | P21549 | GeneCard |
| ISCA2 | Iron-sulfur cluster assembly 2 homolog, mitochondrial | Q86U28 | GeneCard |
| SLC12A1 | Solute carrier family 12 member 1 | Q13621 | GeneCard |
| ADRB2 | Beta-2 adrenergic receptor | P07550 | GeneCard |
| TNFRSF1A | Tumor necrosis factor receptor superfamily member 1A | P19438 | GeneCard |
| NPHS2 | Podocin | Q9NP85 | GeneCard |
| U2AF1 | Splicing factor U2AF 35 kDa subunit | Q01081 | GeneCard |
| CLCN5 | H | P51795 | GeneCard |
| MLXIPL | Carbohydrate-responsive element-binding protein | Q9NP71 | GeneCard |
| NPHP1 | Nephrocystin-1 | O15259 | GeneCard |
| ALDH2 | Aldehyde dehydrogenase, mitochondrial | P05091 | GeneCard |
| NBN | Nibrin | O60934 | GeneCard |
| ANXA5 | Annexin A5 | P08758 | GeneCard |
| NCF2 | Neutrophil cytosol factor 2 | P19878 | OMIM,GeneCard |
| FTH1 | Ferritin heavy chain | P02794 | GeneCard |
| PAX2 | Paired box protein Pax-2 | Q02962 | GeneCard |
| FLNA | Filamin-A | P21333 | GeneCard |
| AGT | Angiotensinogen | P01019 | GeneCard |
| ASS1 | Argininosuccinate synthase | P00966 | GeneCard |
| GNAS | Protein ALEX | P84996 | GeneCard |
| ELN | Elastin | P15502 | GeneCard |
| GATA1 | Erythroid transcription factor | P15976 | GeneCard |
| FANCD2 | Fanconi anemia group D2 protein | Q9BXW9 | GeneCard |
| FHL1 | Four and a half LIM domains protein 1 | Q13642 | GeneCard |
| ABCA3 | ATP-binding cassette sub-family A member 3 | Q99758 | GeneCard |
| NPHS1 | Nephrin | O60500 | GeneCard |
| BGLAP | Osteocalcin | P02818 | GeneCard |
| RAF1 | RAF proto-oncogene serine/threonine-protein kinase | P04049 | GeneCard |
| MIRLET7D | Not Available | Not Available | GeneCard |
| PTPN11 | Tyrosine-protein phosphatase non-receptor type 11 | Q06124 | GeneCard |
| GALNS | N-acetylgalactosamine-6-sulfatase | P34059 | GeneCard |
| TTN-AS1 | Not Available | Not Available | GeneCard |
| GATA3 | Trans-acting T-cell-specific transcription factor GATA-3 | P23771 | GeneCard |
| NPHP3 | Nephrocystin-3 | Q7Z494 | OMIM,GeneCard |
| FANCA | Fanconi anemia group A protein | O15360 | GeneCard |
| FANCI | Fanconi anemia group I protein | Q9NVI1 | GeneCard |
| FTL | Ferritin light chain | P02792 | GeneCard |
| CXCR4 | C-X-C chemokine receptor type 4 | P61073 | GeneCard |
| NOS1 | Nitric oxide synthase, brain | P29475 | GeneCard |
| TET2 | Methylcytosine dioxygenase TET2 | Q6N021 | GeneCard |
| PNLIP | Pancreatic triacylglycerol lipase | P16233 | GeneCard |
| C4A | Complement C4-A | P0C0L4 | GeneCard |
| CYP3A5 | Cytochrome P450 3A5 | P20815 | GeneCard |
| CASP3 | Caspase-3 | P42574 | GeneCard |
| TLR9 | Toll-like receptor 9 | Q9NR96 | GeneCard |
| SELP | P-selectin | P16109 | GeneCard |
| KNG1 | Kininogen-1 | P01042 | GeneCard |
| LMOD3 | Leiomodin-3 | Q0VAK6 | GeneCard |
| PLAT | Tissue-type plasminogen activator | P00750 | GeneCard |
| MMP1 | Interstitial collagenase | P03956 | GeneCard |
| NR3C1 | Glucocorticoid receptor | P04150 | DisGeNET,GeneCard |
| SLPI | Antileukoproteinase | P03973 | GeneCard |
| BMP4 | Bone morphogenetic protein 4 | P12644 | GeneCard |
| IL1R1 | Interleukin-1 receptor type 1 | P14778 | GeneCard |
| MAPK14 | Mitogen-activated protein kinase 14 | Q16539 | GeneCard |
| IFNB1 | Interferon beta | P01574 | GeneCard |
| IFNA2 | Interferon alpha-2 | P01563 | GeneCard |
| IL3 | Interleukin-3 | P08700 | GeneCard |
| AR | Androgen receptor | P10275 | GeneCard |
| FLT3 | Receptor-type tyrosine-protein kinase FLT3 | P36888 | GeneCard |
| FANCC | Fanconi anemia group C protein | Q00597 | GeneCard |
| SLC52A3 | Solute carrier family 52, riboflavin transporter, member 3 | Q9NQ40 | GeneCard |
| STAT4 | Signal transducer and activator of transcription 4 | Q14765 | GeneCard |
| ALDOB | Fructose-bisphosphate aldolase B | P05062 | GeneCard |
| H19 | Not Available | Not Available | GeneCard |
| TCF4 | Transcription factor 4 | P15884 | GeneCard |
| SLC2A1 | Solute carrier family 2, facilitated glucose transporter member 1 | P11166 | GeneCard |
| ACD | Adrenocortical dysplasia protein homolog | Q96AP0 | GeneCard |
| MYH9 | Myosin-9 | P35579 | GeneCard |
| RPS19 | 40S ribosomal protein S19 | P39019 | GeneCard |
| TMPO | Lamina-associated polypeptide 2, isoforms beta/gamma | P42167 | GeneCard |
| GHRL | Appetite-regulating hormone | Q9UBU3 | GeneCard |
| SEPSECS | O-phosphoseryl-tRNA | Q9HD40 | GeneCard |
| GDNF | Glial cell line-derived neurotrophic factor | P39905 | GeneCard |
| C4B | Not Available | Not Available | GeneCard |
| CYP1A2 | Cytochrome P450 1A2 | P05177 | GeneCard |
| TBK1 | Serine/threonine-protein kinase TBK1 | Q9UHD2 | GeneCard |
| CYP2C19 | Cytochrome P450 2C19 | P33261 | GeneCard |
| SMPD1 | Sphingomyelin phosphodiesterase | P17405 | GeneCard |
| PTF1A | Pancreas transcription factor 1 subunit alpha | Q7RTS3 | GeneCard |
| GHR | Growth hormone receptor | P10912 | GeneCard |
| GCG | Glucagon [Cleaved into: Glicentin; Glicentin-related polypeptide | P01275 | GeneCard |
| SPRTN | SprT-like domain-containing protein Spartan | Q9H040 | GeneCard |
| RNASE3 | Eosinophil cationic protein | P12724 | GeneCard |
| SELE | E-selectin | P16581 | GeneCard |
| FABP1 | Fatty acid-binding protein, liver | P07148 | GeneCard |
| COL4A3 | Collagen alpha-3 | Q01955 | GeneCard |
| PMM2 | Phosphomannomutase 2 | O15305 | GeneCard |
| XIAP | E3 ubiquitin-protein ligase XIAP | P98170 | GeneCard |
| ERBB2 | Receptor tyrosine-protein kinase erbB-2 | P04626 | GeneCard |
| CYP2D6 | Cytochrome P450 2D6 | P10635 | GeneCard |
| ANGPT2 | Angiopoietin-2 | O15123 | GeneCard |
| ODC1 | Ornithine decarboxylase | P11926 | OMIM,GeneCard |
| CCK | Cholecystokinin | P06307 | GeneCard |
| LTA | Lymphotoxin-alpha | P01374 | GeneCard |
| STAT6 | Signal transducer and activator of transcription 6 | P42226 | GeneCard |
| FGFR1 | Fibroblast growth factor receptor 1 | P11362 | GeneCard |
| BCL11A | B-cell lymphoma/leukemia 11A | Q9H165 | GeneCard |
| PTPRC | Receptor-type tyrosine-protein phosphatase C | P08575 | GeneCard |
| NPY | Pro-neuropeptide Y [Cleaved into: Neuropeptide Y | P01303 | GeneCard |
| SLC10A2 | Ileal sodium/bile acid cotransporter | Q12908 | GeneCard |
| FANCG | Fanconi anemia group G protein | O15287 | GeneCard |
| PARP1 | Poly [ADP-ribose] polymerase 1 | P09874 | OMIM,GeneCard |
| CD3D | T-cell surface glycoprotein CD3 delta chain | P04234 | GeneCard |
| SOCS3 | Suppressor of cytokine signaling 3 | O14543 | GeneCard |
| CCL11 | Eotaxin | P51671 | GeneCard |
| ALOX5 | Arachidonate 5-lipoxygenase | P09917 | GeneCard |
| MUC5AC | Mucin-5AC | P98088 | GeneCard |
| TNFSF10 | Tumor necrosis factor ligand superfamily member 10 | P50591 | GeneCard |
| PLAU | Urokinase-type plasminogen activator | P00749 | GeneCard |
| HYDIN | Hydrocephalus-inducing protein homolog | Q4G0P3 | GeneCard |
| FLNC | Filamin-C | Q14315 | GeneCard |
| SLC40A1 | Solute carrier family 40 member 1 | Q9NP59 | GeneCard |
| PDGFRB | Platelet-derived growth factor receptor beta | P09619 | OMIM,GeneCard |
| LBR | Delta | Q14739 | GeneCard |
| CCL3 | C-C motif chemokine 3 | P10147 | GeneCard |
| IGF1R | Insulin-like growth factor 1 receptor | P08069 | GeneCard |
| PDGFB | Platelet-derived growth factor subunit B | P01127 | GeneCard |
| LDLR | Low-density lipoprotein receptor | P01130 | GeneCard |
| CYP19A1 | Aromatase | P11511 | GeneCard |
| SFTPB | Pulmonary surfactant-associated protein B | P07988 | GeneCard |
| IL2RB | Interleukin-2 receptor subunit beta | P14784 | GeneCard |
| PKD1L1 | Polycystic kidney disease protein 1-like 1 | Q8TDX9 | GeneCard |
| PIK3CA | Phosphatidylinositol 4,5-bisphosphate 3-kinase catalytic subunit alpha isoform | P42336 | DisGeNET,GeneCard |
| SOCS1 | Suppressor of cytokine signaling 1 | O15524 | GeneCard |
| CD44 | CD44 antigen | P16070 | GeneCard |
| GAPDH | Glyceraldehyde-3-phosphate dehydrogenase | P04406 | GeneCard |
| CR1 | Complement receptor type 1 | P17927 | GeneCard |
| HLA-DPA1 | HLA class II histocompatibility antigen, DP alpha 1 chain | P20036 | GeneCard |
| PITX2 | Pituitary homeobox 2 | Q99697 | GeneCard |
| IFNL3 | Interferon lambda-3 | Q8IZI9 | GeneCard |
| ANPEP | Aminopeptidase N | P15144 | GeneCard |
| HMGCR | 3-hydroxy-3-methylglutaryl-coenzyme A reductase | P04035 | GeneCard |
| CXCL9 | C-X-C motif chemokine 9 | Q07325 | GeneCard |
| RELA | Transcription factor p65 | Q04206 | GeneCard |
| DNAH8 | Dynein heavy chain 8, axonemal | Q96JB1 | GeneCard |
| PCSK1 | Neuroendocrine convertase 1 | P29120 | GeneCard |
| NR3C2 | Mineralocorticoid receptor | P08235 | GeneCard |
| OPRM1 | Mu-type opioid receptor | P35372 | GeneCard |
| CYP1A1 | Cytochrome P450 1A1 | P04798 | GeneCard |
| APRT | Adenine phosphoribosyltransferase | P07741 | GeneCard |
| TNNT2 | Troponin T, cardiac muscle | P45379 | GeneCard |
| NTRK1 | High affinity nerve growth factor receptor | P04629 | GeneCard |
| SCT | Secretin | P09683 | GeneCard |
| CEACAM5 | Carcinoembryonic antigen-related cell adhesion molecule 5 | P06731 | GeneCard |
| ACHE | Acetylcholinesterase | P22303 | GeneCard |
| PIK3R1 | Phosphatidylinositol 3-kinase regulatory subunit alpha | P27986 | GeneCard |
| MB | Myoglobin | P02144 | GeneCard |
| RPL5 | 60S ribosomal protein L5 | P46777 | GeneCard |
| CFHR5 | Complement factor H-related protein 5 | Q9BXR6 | GeneCard |
| MAT1A | S-adenosylmethionine synthase isoform type-1 | Q00266 | GeneCard |
| DES | Desmin | P17661 | GeneCard |
| DPYD | Dihydropyrimidine dehydrogenase [NADP | Q12882 | GeneCard |
| CD40 | Tumor necrosis factor receptor superfamily member 5 | P25942 | GeneCard |
| PF4 | Platelet factor 4 | P02776 | GeneCard |
| PPBP | Platelet basic protein | P02775 | GeneCard |
| GSR | Glutathione reductase, mitochondrial | P00390 | GeneCard |
| KCNN4 | Intermediate conductance calcium-activated potassium channel protein 4 | O15554 | GeneCard |
| CD8A | T-cell surface glycoprotein CD8 alpha chain | P01732 | GeneCard |
| GSTM1 | Glutathione S-transferase Mu 1 | P09488 | GeneCard |
| LGALS3 | Galectin-3 | P17931 | GeneCard |
| PTX3 | Pentraxin-related protein PTX3 | P26022 | GeneCard |
| GDF15 | Growth/differentiation factor 15 | Q99988 | GeneCard |
| TGFB2 | Transforming growth factor beta-2 proprotein | P61812 | GeneCard |
| UBR1 | E3 ubiquitin-protein ligase UBR1 | Q8IWV7 | GeneCard |
| SP1 | Transcription factor Sp1 | P08047 | GeneCard |
| MAPK3 | Mitogen-activated protein kinase 3 | P27361 | GeneCard |
| PRL | Prolactin | P01236 | GeneCard |
| IFNL4 | Interferon lambda-4 | K9M1U5 | GeneCard |
| MAP2K1 | Dual specificity mitogen-activated protein kinase kinase 1 | Q02750 | GeneCard |
| UCP2 | Mitochondrial uncoupling protein 2 | P55851 | GeneCard |
| FGF23 | Fibroblast growth factor 23 | Q9GZV9 | GeneCard |
| KRT19 | Keratin, type I cytoskeletal 19 | P08727 | GeneCard |
| PTGS1 | Prostaglandin G/H synthase 1 | P23219 | GeneCard |
| HSPD1 | 60 kDa heat shock protein, mitochondrial | P10809 | GeneCard |
| IGHE | Immunoglobulin heavy constant epsilon | P01854 | GeneCard |
| SCN4A | Sodium channel protein type 4 subunit alpha | P35499 | GeneCard |
| GZMB | Granzyme B | P10144 | GeneCard |
| ABCC1 | Multidrug resistance-associated protein 1 | P33527 | GeneCard |
| CYCS | Cytochrome c | P99999 | GeneCard |
| CREB1 | Cyclic AMP-responsive element-binding protein 1 | P16220 | GeneCard |
| SCN5A | Sodium channel protein type 5 subunit alpha | Q14524 | GeneCard |
| TNFRSF11B | Tumor necrosis factor receptor superfamily member 11B | O00300 | GeneCard |
| PIK3CG | Phosphatidylinositol 4,5-bisphosphate 3-kinase catalytic subunit gamma isoform | P48736 | DisGeNET,GeneCard |
| MDM2 | E3 ubiquitin-protein ligase Mdm2 | Q00987 | GeneCard |
| CHKB | Choline/ethanolamine kinase | Q9Y259 | GeneCard |
| LIPC | Hepatic triacylglycerol lipase | P11150 | GeneCard |
| KCNJ11 | ATP-sensitive inward rectifier potassium channel 11 | Q14654 | GeneCard |
| CSF3R | Granulocyte colony-stimulating factor receptor | Q99062 | GeneCard |
| NR1H2 | Oxysterols receptor LXR-beta | P55055 | GeneCard |
| LEPR | Leptin receptor | P48357 | GeneCard |
| VIP | VIP peptides [Cleaved into: Intestinal peptide PHV-42 | P01282 | GeneCard |
| MTHFR | Methylenetetrahydrofolate reductase | P42898 | GeneCard |
| CSF1 | Macrophage colony-stimulating factor 1 | P09603 | GeneCard |
| NAGLU | Alpha-N-acetylglucosaminidase | P54802 | GeneCard |
| PRKAG2 | 5'-AMP-activated protein kinase subunit gamma-2 | Q9UGJ0 | GeneCard |
| CCL17 | C-C motif chemokine 17 | Q92583 | GeneCard |
| FARSB | Phenylalanine--tRNA ligase beta subunit | Q9NSD9 | GeneCard |
| CD14 | Monocyte differentiation antigen CD14 | P08571 | GeneCard |
| PCK2 | Phosphoenolpyruvate carboxykinase [GTP], mitochondrial | Q16822 | GeneCard |
| SCGB1A1 | Uteroglobin | P11684 | GeneCard |
| PKHD1 | Fibrocystin | P08F94 | OMIM,GeneCard |
| ATP7A | Copper-transporting ATPase 1 | Q04656 | GeneCard |
| S100A9 | Protein S100-A9 | P06702 | GeneCard |
| ALAD | Delta-aminolevulinic acid dehydratase | P13716 | GeneCard |
| ACTC1 | Actin, alpha cardiac muscle 1 | P68032 | GeneCard |
| CTSG | Cathepsin G | P08311 | GeneCard |
| MAPT | Microtubule-associated protein tau | P10636 | GeneCard |
| ALPP | Alkaline phosphatase, placental type | P05187 | GeneCard |
| IGHMBP2 | DNA-binding protein SMUBP-2 | P38935 | GeneCard |
| PLG | Plasminogen | P00747 | DisGeNET,GeneCard |
| SERPINA6 | Corticosteroid-binding globulin | P08185 | GeneCard |
| IL7R | Interleukin-7 receptor subunit alpha | P16871 | GeneCard |
| LAMA2 | Laminin subunit alpha-2 | P24043 | GeneCard |
| AGPAT2 | 1-acyl-sn-glycerol-3-phosphate acyltransferase beta | O15120 | GeneCard |
| SPINK1 | Serine protease inhibitor Kazal-type 1 | P00995 | GeneCard |
| CRH | Corticoliberin | P06850 | GeneCard |
| BOLA3 | BolA-like protein 3 | Q53S33 | GeneCard |
| LTF | Lactotransferrin | P02788 | GeneCard |
| IL15 | Interleukin-15 | P40933 | GeneCard |
| F7 | Coagulation factor VII | P08709 | GeneCard |
| EARS2 | Probable glutamate--tRNA ligase, mitochondrial | Q5JPH6 | GeneCard |
| MIF | Macrophage migration inhibitory factor | P14174 | GeneCard |
| KITLG | Kit ligand | P21583 | GeneCard |
| DNMT3B | DNA | Q9UBC3 | GeneCard |
| UROD | Uroporphyrinogen decarboxylase | P06132 | GeneCard |
| NR1I2 | Nuclear receptor subfamily 1 group I member 2 | O75469 | GeneCard |
| ANGPT1 | Angiopoietin-1 | Q15389 | GeneCard |
| GAST | Gastrin [Cleaved into: Gastrin-71 | P01350 | GeneCard |
| FCGR3B | Low affinity immunoglobulin gamma Fc region receptor III-B | O75015 | GeneCard |
| ADA2 | Adenosine deaminase 2 | Q9NZK5 | GeneCard |
| IL11 | Interleukin-11 | P20809 | GeneCard |
| CDKN3 | Cyclin-dependent kinase inhibitor 3 | Q16667 | GeneCard |
| PDGFRA | Platelet-derived growth factor receptor alpha | P16234 | GeneCard |
| HBG2 | Hemoglobin subunit gamma-2 | P69892 | GeneCard |
| PGK1 | Phosphoglycerate kinase 1 | P00558 | GeneCard |
| TGFB3 | Transforming growth factor beta-3 proprotein [Cleaved into: Latency-associated peptide | P10600 | GeneCard |
| POU5F1 | POU domain, class 5, transcription factor 1 | Q01860 | OMIM,GeneCard |
| SLCO1B3 | Solute carrier organic anion transporter family member 1B3 | Q9NPD5 | GeneCard |
| AHCY | Adenosylhomocysteinase | P23526 | GeneCard |
| SFTPD | Pulmonary surfactant-associated protein D | P35247 | GeneCard |
| ASXL1 | Polycomb group protein ASXL1 | Q8IXJ9 | GeneCard |
| IGFBP1 | Insulin-like growth factor-binding protein 1 | P08833 | GeneCard |
| SHBG | Sex hormone-binding globulin | P04278 | DisGeNET,GeneCard |
| ITGA2B | Integrin alpha-IIb | P08514 | GeneCard |
| SLCO1B1 | Solute carrier organic anion transporter family member 1B1 | Q9Y6L6 | GeneCard |
| MMP8 | Neutrophil collagenase | P22894 | GeneCard |
| APOH | Beta-2-glycoprotein 1 | P02749 | GeneCard |
| IL9 | Interleukin-9 | P15248 | GeneCard |
| COX5A | Cytochrome c oxidase subunit 5A, mitochondrial | P20674 | GeneCard |
| HSPA4 | Heat shock 70 kDa protein 4 | P34932 | GeneCard |
| TAC1 | Protachykinin-1 | P20366 | GeneCard |
| SLC10A1 | Sodium/bile acid cotransporter | Q14973 | DisGeNET,GeneCard |
| SERPINA7 | Thyroxine-binding globulin | P05543 | GeneCard |
| ACTB | Actin, cytoplasmic 1 | P60709 | GeneCard |
| F10 | Coagulation factor X | P00742 | GeneCard |
| SELENON | Selenoprotein N | Q9NZV5 | GeneCard |
| AVPR2 | Vasopressin V2 receptor | P30518 | GeneCard |
| IRF5 | Interferon regulatory factor 5 | Q13568 | GeneCard |
| ADCY10 | Adenylate cyclase type 10 | Q96PN6 | GeneCard |
| DPP4 | Dipeptidyl peptidase 4 | P27487 | GeneCard |
| APOC2 | Apolipoprotein C-II | P02655 | GeneCard |
| SP110 | Sp110 nuclear body protein | Q9HB58 | OMIM,GeneCard |
| CNR1 | Cannabinoid receptor 1 | P21554 | GeneCard |
| HLA-G | HLA class I histocompatibility antigen, alpha chain G | P17693 | GeneCard |
| IL7 | Interleukin-7 | P13232 | GeneCard |
| MYH6 | Myosin-6 | P13533 | GeneCard |
| CD55 | Complement decay-accelerating factor | P08174 | GeneCard |
| CETP | Cholesteryl ester transfer protein | P11597 | GeneCard |
| NR1I3 | Nuclear receptor subfamily 1 group I member 3 | Q14994 | GeneCard |
| TNFAIP3 | Tumor necrosis factor alpha-induced protein 3 | P21580 | GeneCard |
| NGF | Beta-nerve growth factor | P01138 | GeneCard |
| CPOX | Oxygen-dependent coproporphyrinogen-III oxidase, mitochondrial | P36551 | GeneCard |
| TPMT | Thiopurine S-methyltransferase | P51580 | GeneCard |
| ITGAL | Integrin alpha-L | P20701 | GeneCard |
| CXCR3 | C-X-C chemokine receptor type 3 | P49682 | GeneCard |
| HAVCR1 | Hepatitis A virus cellular receptor 1 | Q96D42 | GeneCard |
| ITGA4 | Integrin alpha-4 | P13612 | GeneCard |
| S100A8 | Protein S100-A8 | P05109 | GeneCard |
| CD86 | T-lymphocyte activation antigen CD86 | P42081 | GeneCard |
| GFER | FAD-linked sulfhydryl oxidase ALR | P55789 | GeneCard |
| SLC2A4 | Solute carrier family 2, facilitated glucose transporter member 4 | P14672 | GeneCard |
| KRT7 | Keratin, type II cytoskeletal 7 | P08729 | GeneCard |
| SMAD2 | Mothers against decapentaplegic homolog 2 | Q15796 | GeneCard |
| F9 | Coagulation factor IX | P00740 | GeneCard |
| LRPPRC | Leucine-rich PPR motif-containing protein, mitochondrial | P42704 | GeneCard |
| SPINK5 | Serine protease inhibitor Kazal-type 5 | Q9NQ38 | GeneCard |
| FASN | Fatty acid synthase | P49327 | GeneCard |
| BAX | Apoptosis regulator BAX | Q07812 | GeneCard |
| CD59 | CD59 glycoprotein | P13987 | GeneCard |
| ADAMTS13 | A disintegrin and metalloproteinase with thrombospondin motifs 13 | Q76LX8 | GeneCard |
| AKT2 | RAC-beta serine/threonine-protein kinase | P31751 | GeneCard |
| MMP12 | Macrophage metalloelastase | P39900 | GeneCard |
| C1S | Complement C1s subcomponent | P09871 | GeneCard |
| AGER | Advanced glycosylation end product-specific receptor | Q15109 | GeneCard |
| MBTPS2 | Membrane-bound transcription factor site-2 protease | O43462 | GeneCard |
| GC | Vitamin D-binding protein | P02774 | GeneCard |
| HMBS | Porphobilinogen deaminase | P08397 | GeneCard |
| CHGA | Chromogranin-A | P10645 | GeneCard |
| CDKN1A | Cyclin-dependent kinase inhibitor 1 | P38936 | GeneCard |
| SPARC | SPARC | P09486 | OMIM,GeneCard |
| AHSG | Alpha-2-HS-glycoprotein | P02765 | GeneCard |
| GLUD1 | Glutamate dehydrogenase 1, mitochondrial | P00367 | GeneCard |
| CHIT1 | Chitotriosidase-1 | Q13231 | GeneCard |
| MEGF10 | Multiple epidermal growth factor-like domains protein 10 | Q96KG7 | GeneCard |
| MMUT | Methylmalonyl-CoA mutase, mitochondrial | P22033 | GeneCard |
| HOTAIR | Not Available | Not Available | GeneCard |
| IREB2 | Iron-responsive element-binding protein 2 | P48200 | GeneCard |
| SPP1 | Osteopontin | P10451 | DisGeNET,GeneCard |
| SAA1 | Serum amyloid A-1 protein | P0DJI8 | GeneCard |
| MATR3 | Matrin-3 | P43243 | GeneCard |
| PLA2G2A | Phospholipase A2, membrane associated | P14555 | GeneCard |
| CHI3L1 | Chitinase-3-like protein 1 | P36222 | GeneCard |
| ICOSLG | ICOS ligand | O75144 | GeneCard |
| LAMP2 | Lysosome-associated membrane glycoprotein 2 | P13473 | GeneCard |
| HNRNPK | Heterogeneous nuclear ribonucleoprotein K | P61978 | GeneCard |
| ITK | Tyrosine-protein kinase ITK/TSK | Q08881 | GeneCard |
| SELL | L-selectin | P14151 | GeneCard |
| MX1 | Interferon-induced GTP-binding protein Mx1 | P20591 | GeneCard |
| CD80 | T-lymphocyte activation antigen CD80 | P33681 | GeneCard |
| SCN1A | Sodium channel protein type 1 subunit alpha | P35498 | GeneCard |
| CDK4 | Cyclin-dependent kinase 4 | P11802 | GeneCard |
| SRP72 | Signal recognition particle subunit SRP72 | O76094 | OMIM,GeneCard |
| ABCB7 | ATP-binding cassette sub-family B member 7, mitochondrial | O75027 | GeneCard |
| RYR2 | Ryanodine receptor 2 | Q92736 | GeneCard |
| TAT | Tyrosine aminotransferase | P17735 | GeneCard |
| PRKCD | Protein kinase C delta type | Q05655 | GeneCard |
| PDE4A | cAMP-specific 3',5'-cyclic phosphodiesterase 4A | P27815 | GeneCard |
| CHRM3 | Muscarinic acetylcholine receptor M3 | P20309 | GeneCard |
| JAK3 | Tyrosine-protein kinase JAK3 | P52333 | GeneCard |
| ADRB1 | Beta-1 adrenergic receptor | P08588 | GeneCard |
| YAP1 | Transcriptional coactivator YAP1 | P46937 | GeneCard |
| DNASE1 | Deoxyribonuclease-1 | P24855 | GeneCard |
| SUMO1 | Small ubiquitin-related modifier 1 | P63165 | GeneCard |
| CTSB | Cathepsin B | P07858 | GeneCard |
| EDNRB | Endothelin receptor type B | P24530 | GeneCard |
| ERBB3 | Receptor tyrosine-protein kinase erbB-3 | P21860 | GeneCard |
| IFT88 | Intraflagellar transport protein 88 homolog | Q13099 | GeneCard |
| GLE1 | Nucleoporin GLE1 | Q53GS7 | GeneCard |
| CYBB | Cytochrome b-245 heavy chain | P04839 | GeneCard |
| F2R | Proteinase-activated receptor 1 | P25116 | GeneCard |
| PCNA | Proliferating cell nuclear antigen | P12004 | GeneCard |
| NQO1 | NAD | P15559 | GeneCard |
| CD209 | CD209 antigen | Q9NNX6 | GeneCard |
| ZMPSTE24 | CAAX prenyl protease 1 homolog | O75844 | GeneCard |
| VIM | Vimentin | P08670 | GeneCard |
| POSTN | Periostin | Q15063 | GeneCard |
| PRKD1 | Serine/threonine-protein kinase D1 | Q15139 | GeneCard |
| TRPV1 | Transient receptor potential cation channel subfamily V member 1 | Q8NER1 | GeneCard |
| COL4A4 | Collagen alpha-4 | P53420 | GeneCard |
| KDR | Vascular endothelial growth factor receptor 2 | P35968 | GeneCard |
| STAR | Steroidogenic acute regulatory protein, mitochondrial | P49675 | OMIM,GeneCard |
| SLC25A4 | ADP/ATP translocase 1 | P12235 | GeneCard |
| GSTT1 | Glutathione S-transferase theta-1 | P30711 | GeneCard |
| MBP | Myelin basic protein | P02686 | GeneCard |
| CCR7 | C-C chemokine receptor type 7 | P32248 | GeneCard |
| PRKCB | Protein kinase C beta type | P05771 | GeneCard |
| TLR7 | Toll-like receptor 7 | Q9NYK1 | GeneCard |
| MT-CO2 | Cytochrome c oxidase subunit 2 | P00403 | GeneCard |
| NEU1 | Sialidase-1 | Q99519 | GeneCard |
| PLAUR | Urokinase plasminogen activator surface receptor | Q03405 | GeneCard |
| SDC1 | Syndecan-1 | P18827 | GeneCard |
| TERT | Telomerase reverse transcriptase | O14746 | OMIM,GeneCard |
| GRB2 | Growth factor receptor-bound protein 2 | P62993 | GeneCard |
| MLH1 | DNA mismatch repair protein Mlh1 | P40692 | GeneCard |
| POT1 | Protection of telomeres protein 1 | Q9NUX5 | GeneCard |
| SCARB1 | Scavenger receptor class B member 1 | Q8WTV0 | GeneCard |
| TLR2 | Toll-like receptor 2 | O60603 | DisGeNET,GeneCard |
| PRNP | Major prion protein | P04156 | GeneCard |
| SUOX | Sulfite oxidase, mitochondrial | P51687 | GeneCard |
| EDN3 | Endothelin-3 | P14138 | GeneCard |
| ATAD1 | ATPase family AAA domain-containing protein 1 | Q8NBU5 | GeneCard |
| KIF3A | Kinesin-like protein KIF3A | Q9Y496 | GeneCard |
| GFAP | Glial fibrillary acidic protein | P14136 | GeneCard |
| IFNAR2 | Interferon alpha/beta receptor 2 | P48551 | GeneCard |
| COL4A5 | Collagen alpha-5 | P29400 | GeneCard |
| IFI27 | Interferon alpha-inducible protein 27, mitochondrial | P40305 | GeneCard |
| BIRC5 | Baculoviral IAP repeat-containing protein 5 | O15392 | GeneCard |
| TNFRSF10B | Tumor necrosis factor receptor superfamily member 10B | O14763 | GeneCard |
| EPRS1 | Bifunctional glutamate/proline--tRNA ligase | P07814 | GeneCard |
| ARID1B | AT-rich interactive domain-containing protein 1B | Q8NFD5 | GeneCard |
| MIR29A | Not Available | Not Available | GeneCard |
| USH2A | Usherin | O75445 | GeneCard |
| IFNAR1 | Interferon alpha/beta receptor 1 | P17181 | GeneCard |
| LEPQTL1 | Not Available | Not Available | GeneCard |
| FGFR2 | Fibroblast growth factor receptor 2 | P21802 | GeneCard |
| AKR1B1 | Aldo-keto reductase family 1 member B1 | P15121 | GeneCard |
| IDH2 | Isocitrate dehydrogenase [NADP], mitochondrial | P48735 | GeneCard |
| CD274 | Programmed cell death 1 ligand 1 | Q9NZQ7 | GeneCard |
| CDKN1B | Cyclin-dependent kinase inhibitor 1B | P46527 | GeneCard |
| CCR3 | C-C chemokine receptor type 3 | P51677 | GeneCard |
| IFNGR1 | Interferon gamma receptor 1 | P15260 | GeneCard |
| MUC4 | Mucin-4 | Q99102 | GeneCard |
| MIR142 | Not Available | Not Available | GeneCard |
| SCN8A | Sodium channel protein type 8 subunit alpha | Q9UQD0 | GeneCard |
| SULT1A3 | Sulfotransferase 1A3 | P0DMM9 | GeneCard |
| DDX58 | Antiviral innate immune response receptor RIG-I | O95786 | GeneCard |
| CD69 | Early activation antigen CD69 | Q07108 | GeneCard |
| MKKS | McKusick-Kaufman/Bardet-Biedl syndromes putative chaperonin | Q9NPJ1 | GeneCard |
| ITGB1 | Integrin beta-1 | P05556 | GeneCard |
| PCSK9 | Proprotein convertase subtilisin/kexin type 9 | Q8NBP7 | GeneCard |
| TLR8 | Toll-like receptor 8 | Q9NR97 | GeneCard |
| MIR152 | Not Available | Not Available | GeneCard |
| GSN | Gelsolin | P06396 | GeneCard |
| MS4A1 | B-lymphocyte antigen CD20 | P11836 | GeneCard |
| TNFRSF10A | Tumor necrosis factor receptor superfamily member 10A | O00220 | GeneCard |
| KCNJ5 | G protein-activated inward rectifier potassium channel 4 | P48544 | GeneCard |
| LIF | Leukemia inhibitory factor | P15018 | GeneCard |
| BMPR2 | Bone morphogenetic protein receptor type-2 | Q13873 | GeneCard |
| KLK3 | Prostate-specific antigen | P07288 | GeneCard |
| MYL1 | Myosin light chain 1/3, skeletal muscle isoform | P05976 | GeneCard |
| AKR1A1 | Aldo-keto reductase family 1 member A1 | P14550 | GeneCard |
| LACTB | Serine beta-lactamase-like protein LACTB, mitochondrial | P83111 | GeneCard |
| BCL2L1 | Bcl-2-like protein 1 | Q07817 | GeneCard |
| ABL1 | Tyrosine-protein kinase ABL1 | P00519 | GeneCard |
| PGF | Placenta growth factor | P49763 | GeneCard |
| CXCL1 | Growth-regulated alpha protein | P09341 | GeneCard |
| VIPR1 | Vasoactive intestinal polypeptide receptor 1 | P32241 | GeneCard |
| MAP3K5 | Mitogen-activated protein kinase kinase kinase 5 | Q99683 | GeneCard |
| GYPB | Glycophorin-B | P06028 | GeneCard |
| EIF2AK2 | Interferon-induced, double-stranded RNA-activated protein kinase | P19525 | GeneCard |
| CASP9 | Caspase-9 | P55211 | GeneCard |
| VCL | Vinculin | P18206 | GeneCard |
| TYK2 | Non-receptor tyrosine-protein kinase TYK2 | P29597 | GeneCard |
| EIF2S1 | Eukaryotic translation initiation factor 2 subunit 1 | P05198 | GeneCard |
| DDIT3 | DNA damage-inducible transcript 3 protein | P35638 | GeneCard |
| MIR200C | Not Available | Not Available | GeneCard |
| ADIPOR1 | Adiponectin receptor protein 1 | Q96A54 | GeneCard |
| DLL4 | Delta-like protein 4 | Q9NR61 | GeneCard |
| HCRT | Orexin | O43612 | GeneCard |
| HSPB1 | Heat shock protein beta-1 | P04792 | GeneCard |
| CHGB | Secretogranin-1 | P05060 | GeneCard |
| TIMP2 | Metalloproteinase inhibitor 2 | P16035 | GeneCard |
| MMAA | Methylmalonic aciduria type A protein, mitochondrial | Q8IVH4 | GeneCard |
| MIR140 | Not Available | Not Available | GeneCard |
| TCN2 | Transcobalamin-2 | P20062 | GeneCard |
| HSPG2 | Basement membrane-specific heparan sulfate proteoglycan core protein | P98160 | GeneCard |
| SERPINB1 | Leukocyte elastase inhibitor | P30740 | GeneCard |
| DBH | Dopamine beta-hydroxylase | P09172 | GeneCard |
| AKT3 | RAC-gamma serine/threonine-protein kinase | Q9Y243 | GeneCard |
| CS | Citrate synthase, mitochondrial | O75390 | GeneCard |
| RB1 | Retinoblastoma-associated protein | P06400 | GeneCard |
| ADH1B | All-trans-retinol dehydrogenase [NAD | P00325 | GeneCard |
| MMP13 | Collagenase 3 | P45452 | GeneCard |
| SLC6A4 | Sodium-dependent serotonin transporter | P31645 | GeneCard |
| BPI | Bactericidal permeability-increasing protein | P17213 | GeneCard |
| AMBP | Protein AMBP [Cleaved into: Alpha-1-microglobulin | P02760 | GeneCard |
| ACP5 | Tartrate-resistant acid phosphatase type 5 | P13686 | GeneCard |
| CLEC4M | C-type lectin domain family 4 member M | Q9H2X3 | GeneCard |
| IFT20 | Intraflagellar transport protein 20 homolog | Q8IY31 | GeneCard |
| MPZ | Myelin protein P0 | P25189 | GeneCard |
| SLC7A9 | b | P82251 | GeneCard |
| IRF3 | Interferon regulatory factor 3 | Q14653 | GeneCard |
| STAT5A | Signal transducer and activator of transcription 5A | P42229 | GeneCard |
| IL17F | Interleukin-17F | Q96PD4 | GeneCard |
| TLR4 | Toll-like receptor 4 | O00206 | DisGeNET,GeneCard |
| IGFBP2 | Insulin-like growth factor-binding protein 2 | P18065 | GeneCard |
| PIGA | Phosphatidylinositol N-acetylglucosaminyltransferase subunit A | P37287 | GeneCard |
| ENO2 | Gamma-enolase | P09104 | GeneCard |
| NR5A2 | Nuclear receptor subfamily 5 group A member 2 | O00482 | GeneCard |
| TLR1 | Toll-like receptor 1 | Q15399 | GeneCard |
| SHH | Sonic hedgehog protein | Q15465 | GeneCard |
| SRC | Proto-oncogene tyrosine-protein kinase Src | P12931 | GeneCard |
| TBX21 | T-box transcription factor TBX21 | Q9UL17 | GeneCard |
| EDNRA | Endothelin-1 receptor | P25101 | GeneCard |
| VTN | Vitronectin | P04004 | GeneCard |
| DNM1L | Dynamin-1-like protein | O00429 | GeneCard |
| WNT5A | Protein Wnt-5a | P41221 | GeneCard |
| F8 | Coagulation factor VIII | P00451 | GeneCard |
| RXRA | Retinoic acid receptor RXR-alpha | P19793 | GeneCard |
| LPA | Apolipoprotein | P08519 | GeneCard |
| RAC1 | Ras-related C3 botulinum toxin substrate 1 | P63000 | GeneCard |
| PRKN | E3 ubiquitin-protein ligase parkin | O60260 | GeneCard |
| MMAB | Corrinoid adenosyltransferase | Q96EY8 | GeneCard |
| ACP1 | Low molecular weight phosphotyrosine protein phosphatase | P24666 | GeneCard |
| ITGB2 | Integrin beta-2 | P05107 | GeneCard |
| GRHPR | Glyoxylate reductase/hydroxypyruvate reductase | Q9UBQ7 | GeneCard |
| TYMS | Thymidylate synthase | P04818 | GeneCard |
| MIR30D | Not Available | Not Available | GeneCard |
| LRP2 | Low-density lipoprotein receptor-related protein 2 | P98164 | GeneCard |
| MAVS | Mitochondrial antiviral-signaling protein | Q7Z434 | GeneCard |
| HLA-C | HLA class I histocompatibility antigen, C alpha chain | P10321 | GeneCard |
| TPO | Thyroid peroxidase | P07202 | GeneCard |
| NGFR | Tumor necrosis factor receptor superfamily member 16 | P08138 | GeneCard |
| TNF | Tumor necrosis factor | P01375 | DisGeNET,GeneCard |
| WRN | Werner syndrome ATP-dependent helicase | Q14191 | GeneCard |
| MCL1 | Induced myeloid leukemia cell differentiation protein Mcl-1 | Q07820 | GeneCard |
| FCGR1A | High affinity immunoglobulin gamma Fc receptor I | P12314 | GeneCard |
| PNOC | Prepronociceptin [Cleaved into: Nocistatin; Nociceptin | Q13519 | GeneCard |
| HTR3A | 5-hydroxytryptamine receptor 3A | P46098 | GeneCard |
| TSPO | Putative peripheral benzodiazepine receptor-related protein | B1AH88 | GeneCard |
| PVT1 | Not Available | Not Available | GeneCard |
| SLC1A2 | Excitatory amino acid transporter 2 | P43004 | GeneCard |
| BCR | Breakpoint cluster region protein | P11274 | GeneCard |
| PSEN1 | Presenilin-1 | P49768 | GeneCard |
| GSK3B | Glycogen synthase kinase-3 beta | P49841 | GeneCard |
| RASSF1 | Ras association domain-containing protein 1 | Q9NS23 | GeneCard |
| CD27 | CD27 antigen | P26842 | GeneCard |
| GPD1 | Glycerol-3-phosphate dehydrogenase [NAD | P21695 | GeneCard |
| DNMT1 | DNA | P26358 | GeneCard |
| LOX | Protein-lysine 6-oxidase | P28300 | GeneCard |
| CRYAB | Alpha-crystallin B chain | P02511 | GeneCard |
| SMAD3 | Mothers against decapentaplegic homolog 3 | P84022 | GeneCard |
| ERCC2 | General transcription and DNA repair factor IIH helicase subunit XPD | P18074 | GeneCard |
| MMP3 | Stromelysin-1 | P08254 | GeneCard |
| ADCY6 | Adenylate cyclase type 6 | O43306 | GeneCard |
| GAL | Galanin peptides [Cleaved into: Galanin; Galanin message-associated peptide | P22466 | GeneCard |
| TNFRSF6B | Tumor necrosis factor receptor superfamily member 6B | O95407 | DisGeNET,GeneCard |
| NID1 | Nidogen-1 | P14543 | GeneCard |
| TSLP | Thymic stromal lymphopoietin | Q969D9 | GeneCard |
| ECI2 | Enoyl-CoA delta isomerase 2, mitochondrial | O75521 | GeneCard |
| NEUROD1 | Neurogenic differentiation factor 1 | Q13562 | GeneCard |
| IRF7 | Interferon regulatory factor 7 | Q92985 | GeneCard |
| RUNX1 | Runt-related transcription factor 1 | Q01196 | GeneCard |
| MGP | Matrix Gla protein | P08493 | GeneCard |
| CIITA | MHC class II transactivator | P33076 | GeneCard |
| ADORA2A | Adenosine receptor A2a | P29274 | GeneCard |
| MUC6 | Mucin-6 | Q6W4X9 | GeneCard |
| TNFSF13B | Tumor necrosis factor ligand superfamily member 13B | Q9Y275 | GeneCard |
| SLC11A1 | Natural resistance-associated macrophage protein 1 | P49279 | GeneCard |
| PROM1 | Prominin-1 | O43490 | GeneCard |
| ACACA | Acetyl-CoA carboxylase 1 | Q13085 | GeneCard |
| MIR93 | Not Available | Not Available | GeneCard |
| CYBA | Cytochrome b-245 light chain | P13498 | GeneCard |
| PLA2G4A | Cytosolic phospholipase A2 | P47712 | GeneCard |
| ADRB3 | Beta-3 adrenergic receptor | P13945 | GeneCard |
| ADAMTSL1 | ADAMTS-like protein 1 | Q8N6G6 | GeneCard |
| CCR4 | C-C chemokine receptor type 4 | P51679 | GeneCard |
| NDUFA5 | NADH dehydrogenase [ubiquinone] 1 alpha subcomplex subunit 5 | Q16718 | GeneCard |
| ADORA1 | Adenosine receptor A1 | P30542 | GeneCard |
| CXCL5 | C-X-C motif chemokine 5 | P42830 | GeneCard |
| CCL21 | C-C motif chemokine 21 | O00585 | GeneCard |
| ADH1C | Alcohol dehydrogenase 1C | P00326 | GeneCard |
| PRKAA1 | 5'-AMP-activated protein kinase catalytic subunit alpha-1 | Q13131 | GeneCard |
| FABP3 | Fatty acid-binding protein, heart | P05413 | GeneCard |
| LAT | Linker for activation of T-cells family member 1 | O43561 | GeneCard |
| TNFRSF11A | Tumor necrosis factor receptor superfamily member 11A | Q9Y6Q6 | GeneCard |
| HBG1 | Hemoglobin subunit gamma-1 | P69891 | GeneCard |
| USP18 | Ubl carboxyl-terminal hydrolase 18 | Q9UMW8 | GeneCard |
| FGB | Fibrinogen beta chain [Cleaved into: Fibrinopeptide B; Fibrinogen beta chain] | P02675 | GeneCard |
| SNCA | Alpha-synuclein | P37840 | GeneCard |
| IL1RAPL2 | X-linked interleukin-1 receptor accessory protein-like 2 | Q9NP60 | GeneCard |
| ICOS | Inducible T-cell costimulator | Q9Y6W8 | GeneCard |
| TTN | Titin | Q8WZ42 | OMIM,GeneCard |
| KL | Klotho | Q9UEF7 | GeneCard |
| CD3E | T-cell surface glycoprotein CD3 epsilon chain | P07766 | GeneCard |
| IL18R1 | Interleukin-18 receptor 1 | Q13478 | GeneCard |
| NAMPT | Nicotinamide phosphoribosyltransferase | P43490 | GeneCard |
| ITGA2 | Integrin alpha-2 | P17301 | GeneCard |
| PLA2G7 | Platelet-activating factor acetylhydrolase | Q13093 | GeneCard |
| CALB2 | Calretinin | P22676 | GeneCard |
| IL4R | Interleukin-4 receptor subunit alpha | P24394 | GeneCard |
| GHRH | Somatoliberin | P01286 | GeneCard |
| RPS6 | 40S ribosomal protein S6 | P62753 | GeneCard |
| MAOA | Amine oxidase [flavin-containing] A | P21397 | GeneCard |
| MYL2 | Myosin regulatory light chain 2, ventricular/cardiac muscle isoform | P10916 | GeneCard |
| CCS | Copper chaperone for superoxide dismutase | O14618 | GeneCard |
| HLA-DRA | HLA class II histocompatibility antigen, DR alpha chain | P01903 | GeneCard |
| ENO1 | Alpha-enolase | P06733 | GeneCard |
| JUP | Junction plakoglobin | P14923 | GeneCard |
| MMP7 | Matrilysin | P09237 | GeneCard |
| REG3A | Regenerating islet-derived protein 3-alpha | Q06141 | GeneCard |
| MIR148B | Not Available | Not Available | GeneCard |
| PRKAB1 | 5'-AMP-activated protein kinase subunit beta-1 | Q9Y478 | GeneCard |
| IKBKB | Inhibitor of nuclear factor kappa-B kinase subunit beta | O14920 | GeneCard |
| HRH2 | Histamine H2 receptor | P25021 | GeneCard |
| TMPRSS15 | Enteropeptidase | P98073 | GeneCard |
| PTPN3 | Tyrosine-protein phosphatase non-receptor type 3 | P26045 | GeneCard |
| TLR6 | Toll-like receptor 6 | Q9Y2C9 | GeneCard |
| APP | Amyloid-beta precursor protein | P05067 | GeneCard |
| F13A1 | Coagulation factor XIII A chain | P00488 | GeneCard |
| CCKAR | Cholecystokinin receptor type A | P32238 | GeneCard |
| C5AR1 | C5a anaphylatoxin chemotactic receptor 1 | P21730 | GeneCard |
| BSG | Basigin | P35613 | GeneCard |
| PLP1 | Myelin proteolipid protein | P60201 | GeneCard |
| SF3B1 | Splicing factor 3B subunit 1 | O75533 | GeneCard |
| PTK2 | Focal adhesion kinase 1 | Q05397 | GeneCard |
| STAT5B | Signal transducer and activator of transcription 5B | P51692 | GeneCard |
| GCDH | Glutaryl-CoA dehydrogenase, mitochondrial | Q92947 | GeneCard |
| PRKCA | Protein kinase C alpha type | P17252 | GeneCard |
| MIR34C | Not Available | Not Available | GeneCard |
| TIMP3 | Metalloproteinase inhibitor 3 | P35625 | GeneCard |
| CTSK | Cathepsin K | P43235 | GeneCard |
| AOC3 | Membrane primary amine oxidase | Q16853 | GeneCard |
| VAPB | Vesicle-associated membrane protein-associated protein B/C | O95292 | GeneCard |
| TERF1 | Telomeric repeat-binding factor 1 | P54274 | GeneCard |
| ASGR2 | Asialoglycoprotein receptor 2 | P07307 | GeneCard |
| VWF | von Willebrand factor | P04275 | DisGeNET,GeneCard |
| CDH2 | Cadherin-2 | P19022 | GeneCard |
| IL1RL1 | Interleukin-1 receptor-like 1 | Q01638 | GeneCard |
| UGT1A | Not Available | Not Available | GeneCard |
| UTS2 | Urotensin-2 | O95399 | GeneCard |
| C5 | Complement C5 | P01031 | GeneCard |
| MGMT | Methylated-DNA--protein-cysteine methyltransferase | P16455 | GeneCard |
| MAPK10 | Mitogen-activated protein kinase 10 | P53779 | GeneCard |
| COL2A1 | Collagen alpha-1 | P02458 | GeneCard |
| TLR10 | Toll-like receptor 10 | Q9BXR5 | GeneCard |
| FCER2 | Low affinity immunoglobulin epsilon Fc receptor | P06734 | GeneCard |
| ADCYAP1 | Pituitary adenylate cyclase-activating polypeptide | P18509 | GeneCard |
| CDX2 | Homeobox protein CDX-2 | Q99626 | GeneCard |
| FOXO1 | Forkhead box protein O1 | Q12778 | GeneCard |
| MYCN | N-myc proto-oncogene protein | P04198 | GeneCard |
| CD33 | Myeloid cell surface antigen CD33 | P20138 | GeneCard |
| SERPINB2 | Plasminogen activator inhibitor 2 | P05120 | GeneCard |
| RSAD2 | Radical S-adenosyl methionine domain-containing protein 2 | Q8WXG1 | GeneCard |
| CA1 | Carbonic anhydrase 1 | P00915 | GeneCard |
| P4HB | Protein disulfide-isomerase | P07237 | GeneCard |
| LBP | Lipopolysaccharide-binding protein | P18428 | GeneCard |
| NCAM1 | Neural cell adhesion molecule 1 | P13591 | GeneCard |
| CTNNA1 | Catenin alpha-1 | P35221 | GeneCard |
| NTS | Neurotensin/neuromedin N [Cleaved into: Large neuromedin N | P30990 | GeneCard |
| HELLS | Lymphoid-specific helicase | Q9NRZ9 | GeneCard |
| CEBPB | CCAAT/enhancer-binding protein beta | P17676 | GeneCard |
| TH | Tyrosine 3-monooxygenase | P07101 | GeneCard |
| IL12RB2 | Interleukin-12 receptor subunit beta-2 | Q99665 | GeneCard |
| F2RL1 | Proteinase-activated receptor 2 | P55085 | GeneCard |
| IRF9 | Interferon regulatory factor 9 | Q00978 | GeneCard |
| CFHR1 | Complement factor H-related protein 1 | Q03591 | GeneCard |
| TNFSF12 | Tumor necrosis factor ligand superfamily member 12 | O43508 | GeneCard |
| IL12B | Interleukin-12 subunit beta | P29460 | GeneCard |
| FOXM1 | Forkhead box protein M1 | Q08050 | GeneCard |
| HOGA1 | 4-hydroxy-2-oxoglutarate aldolase, mitochondrial | Q86XE5 | GeneCard |
| HBS1L | HBS1-like protein | Q9Y450 | GeneCard |
| PECAM1 | Platelet endothelial cell adhesion molecule | P16284 | GeneCard |
| SLC22A4 | Solute carrier family 22 member 4 | Q9H015 | GeneCard |
| EGR1 | Early growth response protein 1 | P18146 | GeneCard |
| KRT14 | Keratin, type I cytoskeletal 14 | P02533 | GeneCard |
| GSDMB | Gasdermin-B | Q8TAX9 | GeneCard |
| ORMDL3 | ORM1-like protein 3 | Q8N138 | GeneCard |
| ANXA2 | Annexin A2 | P07355 | GeneCard |
| CLU | Clusterin | P10909 | GeneCard |
| DAPK1 | Death-associated protein kinase 1 | P53355 | GeneCard |
| ITGAX | Integrin alpha-X | P20702 | GeneCard |
| PLA2G6 | 85/88 kDa calcium-independent phospholipase A2 | O60733 | GeneCard |
| NFKBIA | NF-kappa-B inhibitor alpha | P25963 | GeneCard |
| DOK7 | Protein Dok-7 | Q18PE1 | GeneCard |
| S100B | Protein S100-B | P04271 | GeneCard |
| IL21R | Interleukin-21 receptor | Q9HBE5 | GeneCard |
| PKD2L1 | Polycystic kidney disease 2-like 1 protein | Q9P0L9 | GeneCard |
| FKBP5 | Peptidyl-prolyl cis-trans isomerase FKBP5 | Q13451 | GeneCard |
| SCD | Acyl-CoA desaturase | O00767 | GeneCard |
| CHUK | Inhibitor of nuclear factor kappa-B kinase subunit alpha | O15111 | GeneCard |
| GPX3 | Glutathione peroxidase 3 | P22352 | GeneCard |
| RSF1 | Remodeling and spacing factor 1 | Q96T23 | GeneCard |
| TEK | Angiopoietin-1 receptor | Q02763 | GeneCard |
| CABIN1 | Calcineurin-binding protein cabin-1 | Q9Y6J0 | GeneCard |
