# Supplementary material for: Network Pharmacology Approach to Explore the Potential Mechanisms of Jieduan-Niwan Formula Treating Acute-on-Chronic Liver Failure
Source: Evid Based Complement Alternat Med. 2020 Dec 30;2020:1041307. doi: 10.1155/2020/1041307 (PMC7787753; doi:10.1155/2020/1041307)
Supplement: Supplementary Materials — Supplementary Material 1: Table S1: information of potentially bioactive compounds of Jieduan-Niwan Formula. Supplementary Material 2: Table S2: the details of targets from compounds in JDNW Formula. Supplementary Material 3: Table S3: 1471 known ACLF-related targets. Supplementary Material 4: Table S4: 168 potential targets shared in JDNW Formula and ACLF. Supplementary Material 5: Table S5: information of potentially bioactive compounds of 168 common targets. Supplementary Material 6: Table S6: GO cellular component enrichment analysis of key targets of JDNW Formula in the treatment of ACLF. Supplementary Material 7: Table S7: KEGG pathway enrichment analysis of key targets of JDNW Formula in the treatment of ACLF. [file 1041307.f1.zip › 1041307.f1/Table S4.1041307.v2.docx]

| **168 potential targets shared in JDNW formula and ACLF** | | |
| --- | --- | --- |
| **Gene Symbol** | **Target Protein Name** | **Uniprot ID** |
| MMP2 | 72 kDa type IV collagenase | P08253 |
| XDH | Xanthine dehydrogenase/oxidase [Includes: Xanthine dehydrogenase | P47989 |
| EDN1 | Endothelin-1 | P05305 |
| ADRA2C | Alpha-2C adrenergic receptor | P18825 |
| HSPB1 | Heat shock protein beta-1 | P04792 |
| PLAU | Urokinase-type plasminogen activator | P00749 |
| PTEN | Phosphatidylinositol 3,4,5-trisphosphate 3-phosphatase and dual-specificity protein phosphatase PTEN | P60484 |
| CYCS | Cytochrome c | P99999 |
| NOS2 | Nitric oxide synthase, inducible | P35228 |
| SLC6A4 | Sodium-dependent serotonin transporter | P31645 |
| MET | Hepatocyte growth factor receptor | P08581 |
| IRF1 | Interferon regulatory factor 1 | P10914 |
| ALOX5 | Arachidonate 5-lipoxygenase | P09917 |
| GJA1 | Gap junction alpha-1 protein | P17302 |
| FASN | Fatty acid synthase | P49327 |
| PRKCB | Protein kinase C beta type | P05771 |
| RXRA | Retinoic acid receptor RXR-alpha | P19793 |
| C5AR1 | C5a anaphylatoxin chemotactic receptor 1 | P21730 |
| ACACA | Acetyl-CoA carboxylase 1 | Q13085 |
| BIRC5 | Baculoviral IAP repeat-containing protein 5 | O15392 |
| DPP4 | Dipeptidyl peptidase 4 | P27487 |
| DRD5 | D(1B) dopamine receptor | P21918 |
| MAOA | Amine oxidase [flavin-containing] A | P21397 |
| PON1 | Serum paraoxonase/arylesterase 1 | P27169 |
| BCHE | Cholinesterase | P06276 |
| BCL2 | Apoptosis regulator Bcl-2 | P10415 |
| NPM1 | Nucleophosmin | P06748 |
| F7 | Coagulation factor VII | P08709 |
| ADORA2A | Adenosine receptor A2a | P29274 |
| EDNRA | Endothelin-1 receptor | P25101 |
| CYP1A1 | Cytochrome P450 1A1 | P04798 |
| E2F1 | Transcription factor E2F1 | Q01094 |
| HTR3A | 5-hydroxytryptamine receptor 3A | P46098 |
| CASP8 | Caspase-8 | Q14790 |
| PPARG | Peroxisome proliferator-activated receptor gamma | P37231 |
| CRP | C-reactive protein [Cleaved into: C-reactive protein | P02741 |
| GSTP1 | Glutathione S-transferase P | P09211 |
| CXCL8 | Interleukin-8 | P10145 |
| SELE | E-selectin | P16581 |
| NFE2L2 | Nuclear factor erythroid 2-related factor 2 | Q16236 |
| NR3C1 | Glucocorticoid receptor | P04150 |
| NR1I3 | Nuclear receptor subfamily 1 group I member 3 | Q14994 |
| THBD | Thrombomodulin | P07204 |
| CYP19A1 | Aromatase | P11511 |
| MAPK14 | Mitogen-activated protein kinase 14 | Q16539 |
| ADRB1 | Beta-1 adrenergic receptor | P08588 |
| CTSB | Cathepsin B | P07858 |
| TNF | Tumor necrosis factor | P01375 |
| RAF1 | RAF proto-oncogene serine/threonine-protein kinase | P04049 |
| EGF | Pro-epidermal growth factor | P01133 |
| IL1A | Interleukin-1 alpha | P01583 |
| MPO | Myeloperoxidase | P05164 |
| PCNA | Proliferating cell nuclear antigen | P12004 |
| CHUK | Inhibitor of nuclear factor kappa-B kinase subunit alpha | O15111 |
| STAT3 | Signal transducer and activator of transcription 3 | P40763 |
| CASP1 | Caspase-1 | P29466 |
| SPP1 | Osteopontin | P10451 |
| F2 | Prothrombin | P00734 |
| XIAP | E3 ubiquitin-protein ligase XIAP | P98170 |
| PTGS2 | Prostaglandin G/H synthase 2 | P35354 |
| SLPI | Antileukoproteinase | P03973 |
| NCF1 | Neutrophil cytosol factor 1 | P14598 |
| ADH1C | Alcohol dehydrogenase 1C | P00326 |
| CCND1 | G1/S-specific cyclin-D1 | P24385 |
| ESR1 | Estrogen receptor | P03372 |
| CDK4 | Cyclin-dependent kinase 4 | P11802 |
| ITGB2 | Integrin beta-2 | P05107 |
| ADRB2 | Beta-2 adrenergic receptor | P07550 |
| VEGFA | Vascular endothelial growth factor A | P15692 |
| TGFB1 | Transforming growth factor beta-1 proprotein [Cleaved into: Latency-associated peptide | P01137 |
| MYC | Myc proto-oncogene protein | P01106 |
| IL13 | Interleukin-13 | P35225 |
| ACHE | Acetylcholinesterase | P22303 |
| GSK3B | Glycogen synthase kinase-3 beta | P49841 |
| MCL1 | Induced myeloid leukemia cell differentiation protein Mcl-1 | Q07820 |
| CCL2 | C-C motif chemokine 2 | P13500 |
| MMP1 | Interstitial collagenase | P03956 |
| STAT1 | Signal transducer and activator of transcription 1-alpha/beta | P42224 |
| IL6 | Interleukin-6 | P05231 |
| MMP12 | Macrophage metalloelastase | P39900 |
| CASP3 | Caspase-3 | P42574 |
| PARP1 | Poly [ADP-ribose] polymerase 1 | P09874 |
| HMGCR | 3-hydroxy-3-methylglutaryl-coenzyme A reductase | P04035 |
| NR3C2 | Mineralocorticoid receptor | P08235 |
| INPPL1 | Phosphatidylinositol 3,4,5-trisphosphate 5-phosphatase 2 | O15357 |
| FASLG | Tumor necrosis factor ligand superfamily member 6 | P48023 |
| KDR | Vascular endothelial growth factor receptor 2 | P35968 |
| HMOX1 | Heme oxygenase 1 | P09601 |
| INS | Insulin [Cleaved into: Insulin B chain; Insulin A chain] | P01308 |
| PECAM1 | Platelet endothelial cell adhesion molecule | P16284 |
| MMP3 | Stromelysin-1 | P08254 |
| PPARA | Peroxisome proliferator-activated receptor alpha | Q07869 |
| TTR | Transthyretin | P02766 |
| CYP1A2 | Cytochrome P450 1A2 | P05177 |
| ADCYAP1 | Pituitary adenylate cyclase-activating polypeptide | P18509 |
| GSTM1 | Glutathione S-transferase Mu 1 | P09488 |
| IL10 | Interleukin-10 | P22301 |
| MAPK1 | Mitogen-activated protein kinase 1 | P28482 |
| IGF2 | Insulin-like growth factor II | P01344 |
| PIK3CG | Phosphatidylinositol 4,5-bisphosphate 3-kinase catalytic subunit gamma isoform | P48736 |
| PLAT | Tissue-type plasminogen activator | P00750 |
| MDM2 | E3 ubiquitin-protein ligase Mdm2 | Q00987 |
| EGFR | Epidermal growth factor receptor | P00533 |
| IKBKB | Inhibitor of nuclear factor kappa-B kinase subunit beta | O14920 |
| NQO1 | NAD | P15559 |
| SOD1 | Superoxide dismutase [Cu-Zn] | P00441 |
| FLT3 | Receptor-type tyrosine-protein kinase FLT3 | P36888 |
| ADORA1 | Adenosine receptor A1 | P30542 |
| IL2 | Interleukin-2 | P60568 |
| ERBB3 | Receptor tyrosine-protein kinase erbB-3 | P21860 |
| ERBB2 | Receptor tyrosine-protein kinase erbB-2 | P04626 |
| CREB1 | Cyclic AMP-responsive element-binding protein 1 | P16220 |
| FOS | Proto-oncogene c-Fos | P01100 |
| IFNG | Interferon gamma | P01579 |
| IL4 | Interleukin-4 | P05112 |
| FGF2 | Fibroblast growth factor 2 | P09038 |
| MAPK8 | Mitogen-activated protein kinase 8 | P45983 |
| IGF1R | Insulin-like growth factor 1 receptor | P08069 |
| F10 | Coagulation factor X | P00742 |
| ICAM1 | Intercellular adhesion molecule 1 | P05362 |
| CAV1 | Caveolin-1 | Q03135 |
| BCL2L1 | Bcl-2-like protein 1 | Q07817 |
| AKR1B1 | Aldo-keto reductase family 1 member B1 | P15121 |
| CA2 | Carbonic anhydrase 2 | P00918 |
| HIF1A | Hypoxia-inducible factor 1-alpha | Q16665 |
| RELA | Transcription factor p65 | Q04206 |
| SLC2A4 | Solute carrier family 2, facilitated glucose transporter member 4 | P14672 |
| NOS3 | Nitric oxide synthase, endothelial | P29474 |
| RB1 | Retinoblastoma-associated protein | P06400 |
| CFTR | Cystic fibrosis transmembrane conductance regulator | P13569 |
| ODC1 | Ornithine decarboxylase | P11926 |
| OPRM1 | Mu-type opioid receptor | P35372 |
| CXCL10 | C-X-C motif chemokine 10 | P02778 |
| SERPINE1 | Plasminogen activator inhibitor 1 | P05121 |
| VCAM1 | Vascular cell adhesion protein 1 | P19320 |
| TP53 | Cellular tumor antigen p53 | P04637 |
| INSR | Insulin receptor | P06213 |
| CASP9 | Caspase-9 | P55211 |
| ADH1B | All-trans-retinol dehydrogenase [NAD | P00325 |
| CDKN1A | Cyclin-dependent kinase inhibitor 1 | P38936 |
| CAT | Catalase | P04040 |
| FCER2 | Low affinity immunoglobulin epsilon Fc receptor | P06734 |
| AKT1 | RAC-alpha serine/threonine-protein kinase | P31749 |
| SIRT1 | NAD-dependent protein deacetylase sirtuin-1 | Q96EB6 |
| ABCC1 | Multidrug resistance-associated protein 1 | P33527 |
| ITGB3 | Integrin beta-3 | P05106 |
| IL1B | Interleukin-1 beta | P01584 |
| G6PC | Glucose-6-phosphatase | P35575 |
| CHRM3 | Muscarinic acetylcholine receptor M3 | P20309 |
| NFKBIA | NF-kappa-B inhibitor alpha | P25963 |
| IGFBP3 | Insulin-like growth factor-binding protein 3 | P17936 |
| PTGS1 | Prostaglandin G/H synthase 1 | P23219 |
| F3 | Tissue factor | P13726 |
| NR1I2 | Nuclear receptor subfamily 1 group I member 2 | O75469 |
| CDKN2A | Tumor suppressor ARF | Q8N726 |
| CA1 | Carbonic anhydrase 1 | P00915 |
| JUN | Transcription factor AP-1 | P05412 |
| AR | Androgen receptor | P10275 |
| BAX | Apoptosis regulator BAX | Q07812 |
| PRKCA | Protein kinase C alpha type | P17252 |
| CD40LG | CD40 ligand | P29965 |
| RASSF1 | Ras association domain-containing protein 1 | Q9NS23 |
| CYP3A4 | Cytochrome P450 3A4 | P08684 |
| SCN5A | Sodium channel protein type 5 subunit alpha | Q14524 |
| APP | Amyloid-beta precursor protein | P05067 |
| PRSS1 | Trypsin-1 | P07477 |
| CSF2 | Granulocyte-macrophage colony-stimulating factor | P04141 |
| MMP9 | Matrix metalloproteinase-9 | P14780 |
