# Supplementary material for: Network Pharmacology Approach to Explore the Potential Mechanisms of Jieduan-Niwan Formula Treating Acute-on-Chronic Liver Failure
Source: Evid Based Complement Alternat Med. 2020 Dec 30;2020:1041307. doi: 10.1155/2020/1041307 (PMC7787753; doi:10.1155/2020/1041307)
Supplement: Supplementary Materials — Supplementary Material 1: Table S1: information of potentially bioactive compounds of Jieduan-Niwan Formula. Supplementary Material 2: Table S2: the details of targets from compounds in JDNW Formula. Supplementary Material 3: Table S3: 1471 known ACLF-related targets. Supplementary Material 4: Table S4: 168 potential targets shared in JDNW Formula and ACLF. Supplementary Material 5: Table S5: information of potentially bioactive compounds of 168 common targets. Supplementary Material 6: Table S6: GO cellular component enrichment analysis of key targets of JDNW Formula in the treatment of ACLF. Supplementary Material 7: Table S7: KEGG pathway enrichment analysis of key targets of JDNW Formula in the treatment of ACLF. [file 1041307.f1.zip › 1041307.f1/Table S5.1041307.v2.docx]

| **Information of potential bioactive compounds of 168 common targets** | | |
| --- | --- | --- |
| **ID** | **Molecule name** | **Degree** |
| D1 | quercetin | 105 |
| SDH6 | versulin | 64 |
| KWYXZ14 | apigenin | 52 |
| B1 | luteolin | 46 |
| KWYXZ9 | ursolic acid | 41 |
| C1 | kaempferol | 40 |
| DS56 | tanshinone iia | 28 |
| HQ8 | formononetin | 20 |
| HQ4 | 7-O-methylisomucronulatol | 20 |
| DS33 | dihydrotanshinlactone | 20 |
| F1 | isorhamnetin | 19 |
| JQC1 | acacetin | 18 |
| H1 | beta-sitosterol | 17 |
| KWYXZ2 | rutin | 16 |
| HQ3 | 3,9-di-O-methylnissolin | 16 |
| DS30 | dan-shexinkum d | 16 |
| DS29 | cryptotanshinone | 15 |
| DS37 | isocryptotanshi-none | 14 |
| DS13 | 4-methylenemiltirone | 14 |
| DS10 | 2-isopropyl-8-methylphenanthrene-3,4-dione | 14 |
| KWYXZ4 | ellagic acid | 13 |
| HJS2 | Rhamnazin | 13 |
| DS44 | neocryptotanshinone ii | 13 |
| DS43 | Miltirone | 13 |
| DS40 | miltionone Ⅰ | 13 |
| DS38 | Isotanshinone II | 13 |
| DS31 | danshenspiroketallactone | 13 |
| SQ4 | Stigmasterol | 12 |
| HQ9 | Calycosin | 12 |
| HQ6 | (6aR,11aR)-9,10-dimethoxy-6a,11a-dihydro-6H-benzofurano[3,2-c]chromen-3-ol | 12 |
| DS46 | 1-methyl-8,9-dihydro-7H-naphtho[5,6-g]benzofuran-6,10,11-trione | 12 |
| DS32 | deoxyneocryptotanshinone | 12 |
| DS17 | Methylenetanshinquinone | 12 |
| DS52 | salviolone | 11 |
| DS21 | przewaquinone c | 11 |
| DS6 | Dehydrotanshinone II A | 11 |
| SQ3 | ginsenoside rh2 | 10 |
| KWYXZ1 | astragalin | 10 |
| DS35 | epidanshenspiroketallactone | 10 |
| DS1 | 1,2,5,6-tetrahydrotanshinone | 10 |
| PFZ3 | Deltoin | 9 |
| DS16 | 3-beta-Hydroxymethyllenetanshiquinone | 9 |
| DS9 | 5,6-dihydroxy-7-isopropyl-1,1-dimethyl-2,3-dihydrophenanthren-4-one | 9 |
| KWYXZ12 | palmitic acid | 8 |
| KWYXZ5 | Gallic acid | 8 |
| DS57 | (6S)-6-(hydroxymethyl)-1,6-dimethyl-8,9-dihydro-7H-naphtho[8,7-g]benzofuran-10,11-dione | 8 |
| DS45 | neocryptotanshinone | 8 |
| DS11 | 3α-hydroxytanshinoneⅡa | 8 |
| SQ1 | DFV | 7 |
| KWYXZ7 | corilagin | 7 |
| HQ2 | Jaranol | 7 |
| J1 | hederagenin | 7 |
| DS58 | tanshinone Ⅵ | 7 |
| DS48 | (2R)-3-(3,4-dihydroxyphenyl)-2-[(Z)-3-(3,4-dihydroxyphenyl)acryloyl]oxy-propionic acid | 7 |
| DS47 | prolithospermic acid | 7 |
| DS36 | C09092 | 7 |
| DS34 | dihydrotanshinoneⅠ | 7 |
| DS25 | tanshinaldehyde | 7 |
| DS14 | 2-(4-hydroxy-3-methoxyphenyl)-5-(3-hydroxypropyl)-7-methoxy-3-benzofurancarboxaldehyde | 7 |
| DS5 | sugiol | 7 |
| SDH5 | gentistic acid | 6 |
| KWYXZ10 | linalool | 6 |
| KWYXZ3 | quercitrin | 6 |
| GL3 | Hydroxygenkwanin | 6 |
| DS53 | (6S)-6-hydroxy-1-methyl-6-methylol-8,9-dihydro-7H-naphtho[8,7-g]benzofuran-10,11-quinone | 6 |
| DS27 | Danshenol A | 6 |
| DS22 | (6S,7R)-6,7-dihydroxy-1,6-dimethyl-8,9-dihydro-7H-naphtho[8,7-g]benzofuran-10,11-dione | 6 |
| DS20 | Przewaquinone B | 6 |
| DS15 | formyltanshinone | 6 |
| SQ2 | Diop | 5 |
| A2 | oleanolic acid | 5 |
| KWYXZ8 | lupeol | 5 |
| GL1 | Diosmetin | 5 |
| DS55 | Przewaquinone E | 5 |
| DS54 | Tanshindiol B | 5 |
| DS41 | miltionone Ⅱ | 5 |
| DS28 | Salvilenone | 5 |
| SDH2 | Gamma-Aminobutyric Acid | 4 |
| A1 | vanillic acid | 4 |
| KWYXZ11 | phytol | 4 |
| KWYXZ6 | gallocatechin | 4 |
| JQC3 | Hesperetin | 4 |
| HQ7 | Bifendate | 4 |
| PFZ5 | Karanjin | 4 |
| PFZ2 | Delphin_qt | 4 |
| DS51 | salvilenone Ⅰ | 4 |
| DS26 | Danshenol B | 4 |
| DS23 | przewaquinone f | 4 |
| SDH8 | Verbascoside | 3 |
| SDH1 | Catalpol | 3 |
| JQC5 | ent-Epicatechin | 3 |
| HQ12 | 1,7-Dihydroxy-3,9-dimethoxy pterocarpene | 3 |
| DS19 | przewalskin b | 3 |
| KWYXZ13 | 4-hydroxybenzaldehyde | 2 |
| HQ10 | FA | 2 |
| HJS1 | 3'-methyleriodictyol | 2 |
| GL8 | Linolenic acid ethyl ester | 2 |
| I1 | Mandenol | 2 |
| DS50 | salvianolic acid j | 2 |
| DS42 | miltipolone | 2 |
| DS18 | przewalskin a | 2 |
| DS8 | digallate | 2 |
| SDH3 | Acteoside | 1 |
| HQ5 | 9,10-dimethoxypterocarpan-3-O-β-D-glucoside | 1 |
| G1 | sitosterol | 1 |
| PFZ4 | Deoxyandrographolide | 1 |
| GL9 | vitamin-e | 1 |
| GL7 | 7-oxo-dihydrokaro-unidiol | 1 |
| GL6 | 5-dehydrokarounidiol | 1 |
| GL5 | 10α-cucurbita-5,24-diene-3β-ol | 1 |
| GL2 | Spinasterol | 1 |
| DS49 | salvianolic acid g | 1 |
| DS24 | sclareol | 1 |
| DS12 | (E)-3-[2-(3,4-dihydroxyphenyl)-7-hydroxy-benzofuran-4-yl]acrylic acid | 1 |
| DS7 | Baicalin | 1 |
| DS4 | isoimperatorin | 1 |
| DS2 | Poriferasterol | 1 |
