# Supplementary material for: Network Pharmacology Approach to Explore the Potential Mechanisms of Jieduan-Niwan Formula Treating Acute-on-Chronic Liver Failure
Source: Evid Based Complement Alternat Med. 2020 Dec 30;2020:1041307. doi: 10.1155/2020/1041307 (PMC7787753; doi:10.1155/2020/1041307)
Supplement: Supplementary Materials — Supplementary Material 1: Table S1: information of potentially bioactive compounds of Jieduan-Niwan Formula. Supplementary Material 2: Table S2: the details of targets from compounds in JDNW Formula. Supplementary Material 3: Table S3: 1471 known ACLF-related targets. Supplementary Material 4: Table S4: 168 potential targets shared in JDNW Formula and ACLF. Supplementary Material 5: Table S5: information of potentially bioactive compounds of 168 common targets. Supplementary Material 6: Table S6: GO cellular component enrichment analysis of key targets of JDNW Formula in the treatment of ACLF. Supplementary Material 7: Table S7: KEGG pathway enrichment analysis of key targets of JDNW Formula in the treatment of ACLF. [file 1041307.f1.zip › 1041307.f1/Table S6.1041307.v2.docx]

| **GO Cellular Components enrichment analysis of key targets of JDNW Formula in the treatment of ACLF** | | | |
| --- | --- | --- | --- |
| **Term** | **Description** | **LogP** | **Count** |
| GO:0009636 | response to toxic substance | -47.88018785 | 52 |
| GO:0009611 | response to wounding | -43.63746554 | 54 |
| GO:0010035 | response to inorganic substance | -43.27465622 | 50 |
| GO:0032496 | response to lipopolysaccharide | -42.61766805 | 42 |
| GO:0002237 | response to molecule of bacterial origin | -41.42140459 | 42 |
| GO:0097190 | apoptotic signaling pathway | -40.35470075 | 49 |
| GO:1901652 | response to peptide | -38.97442353 | 46 |
| GO:1901699 | cellular response to nitrogen compound | -38.5840217 | 49 |
| GO:0042060 | wound healing | -38.40996958 | 47 |
| GO:0030335 | positive regulation of cell migration | -38.00882075 | 46 |
| GO:0072593 | reactive oxygen species metabolic process | -37.74790982 | 37 |
| GO:2000147 | positive regulation of cell motility | -37.12564675 | 46 |
| GO:0071417 | cellular response to organonitrogen compound | -36.79725869 | 46 |
| GO:0006979 | response to oxidative stress | -36.65339761 | 42 |
| GO:0051272 | positive regulation of cellular component movement | -36.53883834 | 46 |
| GO:0040017 | positive regulation of locomotion | -36.22100687 | 46 |
| GO:0034599 | cellular response to oxidative stress | -34.97815479 | 36 |
| GO:0009991 | response to extracellular stimulus | -34.79920302 | 43 |
| GO:0071396 | cellular response to lipid | -34.69301289 | 44 |
| GO:0046677 | response to antibiotic | -34.27942017 | 36 |
| GO:0071407 | cellular response to organic cyclic compound | -33.40032553 | 41 |
| GO:0031667 | response to nutrient levels | -33.36625706 | 41 |
| GO:2001233 | regulation of apoptotic signaling pathway | -33.20494304 | 38 |
| GO:0008285 | negative regulation of cell proliferation | -32.76904586 | 47 |
| GO:0001568 | blood vessel development | -32.62087745 | 47 |
| GO:0010942 | positive regulation of cell death | -32.59067057 | 46 |
| GO:0048514 | blood vessel morphogenesis | -32.51393075 | 45 |
| GO:0010038 | response to metal ion | -32.45215411 | 36 |
| GO:0032870 | cellular response to hormone stimulus | -32.05675701 | 44 |
| GO:0000302 | response to reactive oxygen species | -31.82420697 | 31 |
| GO:0001525 | angiogenesis | -31.59696253 | 42 |
| GO:0070997 | neuron death | -31.35112898 | 35 |
| GO:0007169 | transmembrane receptor protein tyrosine kinase signaling pathway | -31.33147212 | 45 |
| GO:0009617 | response to bacterium | -31.30666538 | 45 |
| GO:0050673 | epithelial cell proliferation | -31.26399099 | 38 |
| GO:2001234 | negative regulation of apoptotic signaling pathway | -30.51638362 | 30 |
| GO:0070482 | response to oxygen levels | -29.90611167 | 35 |
| GO:0043410 | positive regulation of MAPK cascade | -29.58358146 | 39 |
| GO:2000377 | regulation of reactive oxygen species metabolic process | -29.51799473 | 28 |
| GO:0097191 | extrinsic apoptotic signaling pathway | -29.2176291 | 29 |
| GO:0070848 | response to growth factor | -29.15298424 | 43 |
| GO:1901214 | regulation of neuron death | -28.96142501 | 32 |
| GO:0043408 | regulation of MAPK cascade | -28.94099084 | 43 |
| GO:0033002 | muscle cell proliferation | -28.45502472 | 29 |
| GO:0050678 | regulation of epithelial cell proliferation | -28.28033877 | 34 |
| GO:0043068 | positive regulation of programmed cell death | -28.17078581 | 41 |
| GO:0051347 | positive regulation of transferase activity | -28.17078581 | 41 |
| GO:0071900 | regulation of protein serine/threonine kinase activity | -27.88396594 | 37 |
| GO:0048545 | response to steroid hormone | -27.83940534 | 32 |
| GO:0050727 | regulation of inflammatory response | -27.65182849 | 37 |
| GO:0001666 | response to hypoxia | -27.40954964 | 32 |
| GO:0080135 | regulation of cellular response to stress | -27.37956422 | 42 |
| GO:0043065 | positive regulation of apoptotic process | -27.26170987 | 40 |
| GO:1901653 | cellular response to peptide | -27.11500644 | 33 |
| GO:0036293 | response to decreased oxygen levels | -26.9568916 | 32 |
| GO:0032103 | positive regulation of response to external stimulus | -26.88201833 | 31 |
| GO:0043434 | response to peptide hormone | -26.78545752 | 34 |
| GO:0033674 | positive regulation of kinase activity | -26.7824358 | 38 |
| GO:0048660 | regulation of smooth muscle cell proliferation | -26.70378338 | 25 |
| GO:0051090 | regulation of DNA-binding transcription factor activity | -26.5942336 | 34 |
| GO:0003018 | vascular process in circulatory system | -26.57377145 | 25 |
| GO:0048659 | smooth muscle cell proliferation | -26.57377145 | 25 |
| GO:0071363 | cellular response to growth factor stimulus | -26.51159217 | 40 |
| GO:0002521 | leukocyte differentiation | -26.49695224 | 36 |
| GO:0007568 | aging | -26.36743954 | 30 |
| GO:0008015 | blood circulation | -26.24761553 | 36 |
| GO:0040008 | regulation of growth | -26.09972986 | 39 |
| GO:0030155 | regulation of cell adhesion | -26.08368399 | 40 |
| GO:0097305 | response to alcohol | -26.07539079 | 27 |
| GO:0050878 | regulation of body fluid levels | -25.9674567 | 35 |
| GO:0003013 | circulatory system process | -25.94898141 | 36 |
| GO:0048732 | gland development | -25.72063512 | 33 |
| GO:0035296 | regulation of tube diameter | -25.69368689 | 23 |
| GO:0050880 | regulation of blood vessel size | -25.69368689 | 23 |
| GO:0097746 | regulation of blood vessel diameter | -25.69368689 | 23 |
| GO:0035150 | regulation of tube size | -25.62027932 | 23 |
| GO:0034614 | cellular response to reactive oxygen species | -25.38386901 | 24 |
| GO:0045596 | negative regulation of cell differentiation | -25.34795657 | 40 |
| GO:0032102 | negative regulation of response to external stimulus | -24.75143154 | 31 |
| GO:0050900 | leukocyte migration | -24.74059331 | 34 |
| GO:0051046 | regulation of secretion | -24.68221906 | 39 |
| GO:0035690 | cellular response to drug | -24.21598762 | 30 |
| GO:0071216 | cellular response to biotic stimulus | -24.15250884 | 26 |
| GO:0001101 | response to acid chemical | -24.00700892 | 29 |
| GO:0001819 | positive regulation of cytokine production | -23.94775258 | 32 |
| GO:1903530 | regulation of secretion by cell | -23.85133871 | 37 |
| GO:2000379 | positive regulation of reactive oxygen species metabolic process | -23.84350351 | 20 |
| GO:0070201 | regulation of establishment of protein localization | -23.78603958 | 37 |
| GO:2001237 | negative regulation of extrinsic apoptotic signaling pathway | -23.75583703 | 20 |
| GO:1903409 | reactive oxygen species biosynthetic process | -23.66485189 | 21 |
| GO:1901654 | response to ketone | -23.63900455 | 24 |
| GO:0048608 | reproductive structure development | -23.31982964 | 31 |
| GO:0071222 | cellular response to lipopolysaccharide | -23.22250941 | 24 |
| GO:0061458 | reproductive system development | -23.20319483 | 31 |
| GO:0048871 | multicellular organismal homeostasis | -23.08104897 | 32 |
| GO:0090087 | regulation of peptide transport | -23.0629004 | 36 |
| GO:0018108 | peptidyl-tyrosine phosphorylation | -23.04872566 | 29 |
| GO:0045860 | positive regulation of protein kinase activity | -22.99250809 | 33 |
| GO:0018212 | peptidyl-tyrosine modification | -22.95077767 | 29 |
| GO:2001236 | regulation of extrinsic apoptotic signaling pathway | -22.89435065 | 22 |
| GO:0010817 | regulation of hormone levels | -22.76747429 | 33 |
| GO:0071219 | cellular response to molecule of bacterial origin | -22.53460282 | 24 |
| GO:0009410 | response to xenobiotic stimulus | -22.49045009 | 26 |
| GO:1904951 | positive regulation of establishment of protein localization | -22.16661022 | 29 |
| GO:0045765 | regulation of angiogenesis | -22.13637471 | 29 |
| GO:0010631 | epithelial cell migration | -22.12017533 | 28 |
| GO:1901342 | regulation of vasculature development | -22.08978617 | 30 |
| GO:0009314 | response to radiation | -22.03372244 | 30 |
| GO:0090132 | epithelium migration | -22.02344226 | 28 |
| GO:0090130 | tissue migration | -21.83256532 | 28 |
| GO:0007584 | response to nutrient | -21.30891595 | 23 |
| GO:0001936 | regulation of endothelial cell proliferation | -21.28172265 | 22 |
| GO:0032355 | response to estradiol | -21.22530371 | 20 |
| GO:0062012 | regulation of small molecule metabolic process | -21.14698323 | 30 |
| GO:0022407 | regulation of cell-cell adhesion | -21.07264195 | 29 |
| GO:0044706 | multi-multicellular organism process | -20.99317922 | 23 |
| GO:0048661 | positive regulation of smooth muscle cell proliferation | -20.84776789 | 18 |
| GO:0050730 | regulation of peptidyl-tyrosine phosphorylation | -20.76677918 | 24 |
| GO:0071214 | cellular response to abiotic stimulus | -20.76023112 | 26 |
| GO:0104004 | cellular response to environmental stimulus | -20.76023112 | 26 |
| GO:0051223 | regulation of protein transport | -20.55542309 | 33 |
| GO:0001935 | endothelial cell proliferation | -20.50425042 | 22 |
| GO:0051091 | positive regulation of DNA-binding transcription factor activity | -20.49558469 | 24 |
| GO:0003006 | developmental process involved in reproduction | -20.46494218 | 34 |
| GO:1901615 | organic hydroxy compound metabolic process | -20.43047702 | 31 |
| GO:0006935 | chemotaxis | -20.39173818 | 33 |
| GO:0042330 | taxis | -20.35116993 | 33 |
| GO:0050679 | positive regulation of epithelial cell proliferation | -20.32024768 | 22 |
| GO:0060326 | cell chemotaxis | -20.23787381 | 25 |
| GO:0043491 | protein kinase B signaling | -20.19501032 | 24 |
| GO:0051402 | neuron apoptotic process | -20.18787417 | 23 |
| GO:0030099 | myeloid cell differentiation | -20.14568219 | 28 |
| GO:0071496 | cellular response to external stimulus | -20.11930474 | 26 |
| GO:0001667 | ameboidal-type cell migration | -19.97958657 | 29 |
| GO:0071241 | cellular response to inorganic substance | -19.8766131 | 22 |
| GO:0031960 | response to corticosteroid | -19.87354734 | 20 |
| GO:0043542 | endothelial cell migration | -19.86809573 | 24 |
| GO:0071248 | cellular response to metal ion | -19.79828561 | 21 |
| GO:0007159 | leukocyte cell-cell adhesion | -19.78375064 | 26 |
| GO:0009411 | response to UV | -19.74035691 | 19 |
| GO:1903037 | regulation of leukocyte cell-cell adhesion | -19.67577026 | 25 |
| GO:1903034 | regulation of response to wounding | -19.60964454 | 21 |
| GO:0071375 | cellular response to peptide hormone stimulus | -19.57992353 | 25 |
| GO:0051047 | positive regulation of secretion | -19.48833942 | 26 |
| GO:0051384 | response to glucocorticoid | -19.38620573 | 19 |
| GO:0010632 | regulation of epithelial cell migration | -19.34768059 | 24 |
| GO:0048511 | rhythmic process | -19.21373089 | 24 |
| GO:0010634 | positive regulation of epithelial cell migration | -19.19953097 | 20 |
| GO:0042063 | gliogenesis | -19.18054662 | 24 |
| GO:0045766 | positive regulation of angiogenesis | -19.11195765 | 21 |
| GO:0071902 | positive regulation of protein serine/threonine kinase activity | -19.08446933 | 25 |
| GO:0090066 | regulation of anatomical structure size | -19.07866118 | 29 |
| GO:0009612 | response to mechanical stimulus | -19.06816277 | 21 |
| GO:0097756 | negative regulation of blood vessel diameter | -19.03709769 | 16 |
| GO:0051345 | positive regulation of hydrolase activity | -19.0221539 | 34 |
| GO:0002791 | regulation of peptide secretion | -18.99091793 | 27 |
| GO:0002573 | myeloid leukocyte differentiation | -18.98126369 | 21 |
| GO:0034762 | regulation of transmembrane transport | -18.91069514 | 30 |
| GO:0002685 | regulation of leukocyte migration | -18.89527029 | 21 |
| GO:0009416 | response to light stimulus | -18.75975079 | 24 |
| GO:0034612 | response to tumor necrosis factor | -18.75975079 | 24 |
| GO:0032868 | response to insulin | -18.69753527 | 23 |
| GO:0043281 | regulation of cysteine-type endopeptidase activity involved in apoptotic process | -18.68413356 | 21 |
| GO:0051222 | positive regulation of protein transport | -18.58505756 | 25 |
| GO:0097193 | intrinsic apoptotic signaling pathway | -18.52768439 | 23 |
| GO:0061041 | regulation of wound healing | -18.41289241 | 19 |
| GO:0043269 | regulation of ion transport | -18.38947088 | 32 |
| GO:0038034 | signal transduction in absence of ligand | -18.38035026 | 15 |
| GO:0097192 | extrinsic apoptotic signaling pathway in absence of ligand | -18.38035026 | 15 |
| GO:0009914 | hormone transport | -18.3575174 | 24 |
| GO:0045936 | negative regulation of phosphate metabolic process | -18.35678456 | 30 |
| GO:0050731 | positive regulation of peptidyl-tyrosine phosphorylation | -18.35163063 | 20 |
| GO:0010563 | negative regulation of phosphorus metabolic process | -18.33681144 | 30 |
| GO:0046649 | lymphocyte activation | -18.33029779 | 33 |
| GO:0002790 | peptide secretion | -18.31199633 | 29 |
| GO:0007565 | female pregnancy | -18.30708957 | 20 |
| GO:0007610 | behavior | -18.25729222 | 30 |
| GO:0050920 | regulation of chemotaxis | -18.23789555 | 21 |
| GO:0150076 | neuroinflammatory response | -18.19523537 | 15 |
| GO:1901216 | positive regulation of neuron death | -18.14758287 | 16 |
| GO:0052547 | regulation of peptidase activity | -18.12914861 | 27 |
| GO:1903532 | positive regulation of secretion by cell | -18.0891403 | 24 |
| GO:1904019 | epithelial cell apoptotic process | -18.08166554 | 17 |
| GO:0007596 | blood coagulation | -18.03056778 | 24 |
| GO:1904018 | positive regulation of vasculature development | -18.00437379 | 21 |
| GO:0007599 | hemostasis | -17.88577966 | 24 |
| GO:0050817 | coagulation | -17.85709926 | 24 |
| GO:0046883 | regulation of hormone secretion | -17.84525142 | 22 |
| GO:0042110 | T cell activation | -17.78167593 | 27 |
| GO:0046686 | response to cadmium ion | -17.77690759 | 14 |
| GO:0048638 | regulation of developmental growth | -17.77160275 | 24 |
| GO:2000116 | regulation of cysteine-type endopeptidase activity | -17.74012785 | 21 |
| GO:0014065 | phosphatidylinositol 3-kinase signaling | -17.5802033 | 18 |
| GO:0043523 | regulation of neuron apoptotic process | -17.54575054 | 20 |
| GO:0046879 | hormone secretion | -17.54225427 | 23 |
| GO:0050708 | regulation of protein secretion | -17.50561255 | 25 |
| GO:0042133 | neurotransmitter metabolic process | -17.4777816 | 18 |
| GO:0071276 | cellular response to cadmium ion | -17.46038736 | 12 |
| GO:0097237 | cellular response to toxic substance | -17.44829904 | 21 |
| GO:1902893 | regulation of pri-miRNA transcription by RNA polymerase II | -17.37302142 | 13 |
| GO:0042136 | neurotransmitter biosynthetic process | -17.36470164 | 16 |
| GO:0051098 | regulation of binding | -17.35593425 | 24 |
| GO:0051896 | regulation of protein kinase B signaling | -17.34151918 | 21 |
| GO:0010595 | positive regulation of endothelial cell migration | -17.29841283 | 17 |
| GO:0062013 | positive regulation of small molecule metabolic process | -17.27722844 | 18 |
| GO:0061614 | pri-miRNA transcription by RNA polymerase II | -17.2530458 | 13 |
| GO:0008202 | steroid metabolic process | -17.21777764 | 23 |
| GO:0018209 | peptidyl-serine modification | -17.1888749 | 23 |
| GO:0007259 | JAK-STAT cascade | -17.17902152 | 18 |
| GO:1902895 | positive regulation of pri-miRNA transcription by RNA polymerase II | -17.15026625 | 12 |
| GO:0007346 | regulation of mitotic cell cycle | -17.09578613 | 30 |
| GO:0045862 | positive regulation of proteolysis | -17.062956 | 24 |
| GO:0009306 | protein secretion | -17.05818564 | 27 |
| GO:0045785 | positive regulation of cell adhesion | -16.93763986 | 25 |
| GO:0010001 | glial cell differentiation | -16.92621895 | 20 |
| GO:0023061 | signal release | -16.9004017 | 26 |
| GO:0019216 | regulation of lipid metabolic process | -16.86608974 | 25 |
| GO:0006801 | superoxide metabolic process | -16.82025349 | 14 |
| GO:0043405 | regulation of MAP kinase activity | -16.79410639 | 23 |
| GO:1902532 | negative regulation of intracellular signal transduction | -16.78580778 | 28 |
| GO:0052548 | regulation of endopeptidase activity | -16.72465444 | 25 |
| GO:0097696 | STAT cascade | -16.70730193 | 18 |
| GO:0018105 | peptidyl-serine phosphorylation | -16.69983424 | 22 |
| GO:0050921 | positive regulation of chemotaxis | -16.69930493 | 17 |
| GO:1903035 | negative regulation of response to wounding | -16.66854784 | 15 |
| GO:0045787 | positive regulation of cell cycle | -16.62838644 | 24 |
| GO:0007507 | heart development | -16.61367316 | 28 |
| GO:1904645 | response to amyloid-beta | -16.5843129 | 13 |
| GO:0070661 | leukocyte proliferation | -16.58177283 | 22 |
| GO:0050865 | regulation of cell activation | -16.44432911 | 29 |
| GO:0045471 | response to ethanol | -16.429712 | 16 |
| GO:0006809 | nitric oxide biosynthetic process | -16.39551216 | 14 |
| GO:1903706 | regulation of hemopoiesis | -16.3819452 | 26 |
| GO:1901215 | negative regulation of neuron death | -16.34165693 | 19 |
| GO:0043536 | positive regulation of blood vessel endothelial cell migration | -16.31420428 | 14 |
| GO:0048771 | tissue remodeling | -16.26497341 | 18 |
| GO:0043062 | extracellular structure organization | -16.24631707 | 25 |
| GO:0061045 | negative regulation of wound healing | -16.23403225 | 14 |
| GO:0048589 | developmental growth | -16.14980157 | 29 |
| GO:0007623 | circadian rhythm | -16.11440396 | 19 |
| GO:0046209 | nitric oxide metabolic process | -16.00003177 | 14 |
| GO:0055080 | cation homeostasis | -15.95567247 | 30 |
| GO:0006066 | alcohol metabolic process | -15.95489181 | 23 |
| GO:0099536 | synaptic signaling | -15.92399591 | 30 |
| GO:0071356 | cellular response to tumor necrosis factor | -15.91959111 | 21 |
| GO:0051092 | positive regulation of NF-kappaB transcription factor activity | -15.9127626 | 17 |
| GO:1903829 | positive regulation of cellular protein localization | -15.90687308 | 22 |
| GO:0002526 | acute inflammatory response | -15.89397005 | 19 |
| GO:0048015 | phosphatidylinositol-mediated signaling | -15.84873163 | 18 |
| GO:0098771 | inorganic ion homeostasis | -15.79827455 | 30 |
| GO:1904705 | regulation of vascular smooth muscle cell proliferation | -15.77522374 | 14 |
| GO:1990874 | vascular smooth muscle cell proliferation | -15.77522374 | 14 |
| GO:2001057 | reactive nitrogen species metabolic process | -15.77522374 | 14 |
| GO:0090068 | positive regulation of cell cycle process | -15.77299251 | 21 |
| GO:0045786 | negative regulation of cell cycle | -15.74366392 | 28 |
| GO:0048017 | inositol lipid-mediated signaling | -15.68890222 | 18 |
| GO:0042326 | negative regulation of phosphorylation | -15.68729796 | 25 |
| GO:2001242 | regulation of intrinsic apoptotic signaling pathway | -15.63999732 | 17 |
| GO:0030595 | leukocyte chemotaxis | -15.57529064 | 19 |
| GO:0030198 | extracellular matrix organization | -15.51702549 | 23 |
| GO:1903039 | positive regulation of leukocyte cell-cell adhesion | -15.50632479 | 19 |
| GO:0046427 | positive regulation of JAK-STAT cascade | -15.48861795 | 14 |
| GO:1900407 | regulation of cellular response to oxidative stress | -15.41916315 | 14 |
| GO:0010594 | regulation of endothelial cell migration | -15.37032444 | 19 |
| GO:0046425 | regulation of JAK-STAT cascade | -15.36308477 | 16 |
| GO:0009896 | positive regulation of catabolic process | -15.31108141 | 24 |
| GO:0032787 | monocarboxylic acid metabolic process | -15.30325176 | 28 |
| GO:0001659 | temperature homeostasis | -15.29279856 | 17 |
| GO:0022409 | positive regulation of cell-cell adhesion | -15.29028413 | 20 |
| GO:0050804 | modulation of chemical synaptic transmission | -15.28966977 | 24 |
| GO:1904894 | positive regulation of STAT cascade | -15.28274633 | 14 |
| GO:0007268 | chemical synaptic transmission | -15.26851968 | 29 |
| GO:0098916 | anterograde trans-synaptic signaling | -15.26851968 | 29 |
| GO:0099177 | regulation of trans-synaptic signaling | -15.26831146 | 24 |
| GO:1903708 | positive regulation of hemopoiesis | -15.26732627 | 18 |
| GO:0042542 | response to hydrogen peroxide | -15.21743657 | 16 |
| GO:0001505 | regulation of neurotransmitter levels | -15.20885869 | 22 |
| GO:0099537 | trans-synaptic signaling | -15.14445755 | 29 |
| GO:0008610 | lipid biosynthetic process | -15.12906075 | 29 |
| GO:0051897 | positive regulation of protein kinase B signaling | -15.12575685 | 17 |
| GO:0031100 | animal organ regeneration | -15.12489862 | 13 |
| GO:0032943 | mononuclear cell proliferation | -15.05373845 | 20 |
| GO:0071466 | cellular response to xenobiotic stimulus | -15.00317625 | 17 |
| GO:0044057 | regulation of system process | -14.99614179 | 27 |
| GO:0070371 | ERK1 and ERK2 cascade | -14.94667882 | 21 |
| GO:1904892 | regulation of STAT cascade | -14.93581893 | 16 |
| GO:0031349 | positive regulation of defense response | -14.8147811 | 25 |
| GO:0061900 | glial cell activation | -14.79632215 | 12 |
| GO:0014066 | regulation of phosphatidylinositol 3-kinase signaling | -14.78749735 | 15 |
| GO:0045639 | positive regulation of myeloid cell differentiation | -14.76768096 | 14 |
| GO:1902882 | regulation of response to oxidative stress | -14.76768096 | 14 |
| GO:0031400 | negative regulation of protein modification process | -14.7449057 | 27 |
| GO:0001890 | placenta development | -14.71036758 | 16 |
| GO:0031098 | stress-activated protein kinase signaling cascade | -14.6293983 | 20 |
| GO:0050890 | cognition | -14.6293983 | 20 |
| GO:0031331 | positive regulation of cellular catabolic process | -14.61236998 | 22 |
| GO:0045637 | regulation of myeloid cell differentiation | -14.5737931 | 19 |
| GO:0002793 | positive regulation of peptide secretion | -14.52732778 | 18 |
| GO:0032869 | cellular response to insulin stimulus | -14.52732778 | 18 |
| GO:0048145 | regulation of fibroblast proliferation | -14.52183914 | 13 |
| GO:0042391 | regulation of membrane potential | -14.49259489 | 23 |
| GO:0055065 | metal ion homeostasis | -14.4843064 | 27 |
| GO:0043406 | positive regulation of MAP kinase activity | -14.48388069 | 19 |
| GO:0001933 | negative regulation of protein phosphorylation | -14.47167972 | 23 |
| GO:1903426 | regulation of reactive oxygen species biosynthetic process | -14.46806958 | 14 |
| GO:0048144 | fibroblast proliferation | -14.45105886 | 13 |
| GO:1903827 | regulation of cellular protein localization | -14.44521773 | 25 |
| GO:0051186 | cofactor metabolic process | -14.43953568 | 26 |
| GO:0030225 | macrophage differentiation | -14.4233315 | 11 |
| GO:0072330 | monocarboxylic acid biosynthetic process | -14.41648813 | 21 |
| GO:0060627 | regulation of vesicle-mediated transport | -14.40911366 | 25 |
| GO:0002263 | cell activation involved in immune response | -14.38887461 | 28 |
| GO:0030003 | cellular cation homeostasis | -14.37259764 | 27 |
| GO:0043535 | regulation of blood vessel endothelial cell migration | -14.36566007 | 16 |
| GO:0009266 | response to temperature stimulus | -14.32972011 | 18 |
| GO:0048729 | tissue morphogenesis | -14.30937621 | 27 |
| GO:0071453 | cellular response to oxygen levels | -14.29733271 | 18 |
| GO:0043112 | receptor metabolic process | -14.27557708 | 17 |
| GO:0051051 | negative regulation of transport | -14.26348242 | 24 |
| GO:0045931 | positive regulation of mitotic cell cycle | -14.24113227 | 16 |
| GO:0031099 | regeneration | -14.23942529 | 17 |
| GO:0019932 | second-messenger-mediated signaling | -14.22479118 | 23 |
| GO:0045055 | regulated exocytosis | -14.17777013 | 29 |
| GO:0006873 | cellular ion homeostasis | -14.16872294 | 27 |
| GO:1905952 | regulation of lipid localization | -14.15947178 | 16 |
| GO:0070663 | regulation of leukocyte proliferation | -14.13765901 | 18 |
| GO:0001938 | positive regulation of endothelial cell proliferation | -14.1283957 | 14 |
| GO:0008625 | extrinsic apoptotic signaling pathway via death domain receptors | -14.11071496 | 13 |
| GO:0061061 | muscle structure development | -14.0761577 | 27 |
| GO:0002687 | positive regulation of leukocyte migration | -14.05285488 | 15 |
| GO:0009895 | negative regulation of catabolic process | -14.02247247 | 20 |
| GO:0046651 | lymphocyte proliferation | -13.99475985 | 19 |
| GO:0033273 | response to vitamin | -13.98049786 | 13 |
| GO:0006091 | generation of precursor metabolites and energy | -13.94745241 | 24 |
| GO:0010212 | response to ionizing radiation | -13.91623876 | 15 |
| GO:0051341 | regulation of oxidoreductase activity | -13.91286353 | 14 |
| GO:0051403 | stress-activated MAPK cascade | -13.88441493 | 19 |
| GO:0032147 | activation of protein kinase activity | -13.87203783 | 20 |
| GO:0010638 | positive regulation of organelle organization | -13.8332286 | 26 |
| GO:0046394 | carboxylic acid biosynthetic process | -13.82929767 | 23 |
| GO:0016053 | organic acid biosynthetic process | -13.81002258 | 23 |
| GO:2001235 | positive regulation of apoptotic signaling pathway | -13.65330481 | 16 |
| GO:0051348 | negative regulation of transferase activity | -13.64227801 | 19 |
| GO:0030072 | peptide hormone secretion | -13.62120401 | 18 |
| GO:1901655 | cellular response to ketone | -13.60815069 | 13 |
| GO:2000045 | regulation of G1/S transition of mitotic cell cycle | -13.57897518 | 16 |
| GO:0002688 | regulation of leukocyte chemotaxis | -13.5546096 | 14 |
| GO:0002366 | leukocyte activation involved in immune response | -13.55446499 | 27 |
| GO:0036473 | cell death in response to oxidative stress | -13.54858437 | 13 |
| GO:1904035 | regulation of epithelial cell apoptotic process | -13.54858437 | 13 |
| GO:0060249 | anatomical structure homeostasis | -13.53925822 | 22 |
| GO:0008217 | regulation of blood pressure | -13.50552972 | 16 |
| GO:0002274 | myeloid leukocyte activation | -13.46130958 | 26 |
| GO:1902175 | regulation of oxidative stress-induced intrinsic apoptotic signaling pathway | -13.45910645 | 9 |
| GO:0097529 | myeloid leukocyte migration | -13.45892397 | 17 |
| GO:0010959 | regulation of metal ion transport | -13.43370723 | 21 |
| GO:0006367 | transcription initiation from RNA polymerase II promoter | -13.4329488 | 16 |
| GO:0008585 | female gonad development | -13.43144857 | 13 |
| GO:0002694 | regulation of leukocyte activation | -13.42330097 | 25 |
| GO:1903201 | regulation of oxidative stress-induced cell death | -13.4150984 | 12 |
| GO:0043534 | blood vessel endothelial cell migration | -13.39697659 | 16 |
| GO:0042310 | vasoconstriction | -13.34478869 | 12 |
| GO:0002009 | morphogenesis of an epithelium | -13.33247195 | 24 |
| GO:0050728 | negative regulation of inflammatory response | -13.18545529 | 16 |
| GO:0097755 | positive regulation of blood vessel diameter | -13.15850072 | 11 |
| GO:0046545 | development of primary female sexual characteristics | -13.14961599 | 13 |
| GO:0032800 | receptor biosynthetic process | -13.13378526 | 9 |
| GO:0008631 | intrinsic apoptotic signaling pathway in response to oxidative stress | -13.12648494 | 10 |
| GO:0051249 | regulation of lymphocyte activation | -13.11001048 | 23 |
| GO:0030193 | regulation of blood coagulation | -13.07326744 | 12 |
| GO:1900046 | regulation of hemostasis | -13.00768867 | 12 |
| GO:0071887 | leukocyte apoptotic process | -12.98755979 | 13 |
| GO:0051235 | maintenance of location | -12.96242573 | 19 |
| GO:0010876 | lipid localization | -12.93633275 | 21 |
| GO:0031668 | cellular response to extracellular stimulus | -12.92710255 | 18 |
| GO:0010952 | positive regulation of peptidase activity | -12.91432991 | 16 |
| GO:0035094 | response to nicotine | -12.90983486 | 10 |
| GO:0051100 | negative regulation of binding | -12.88660938 | 15 |
| GO:0001558 | regulation of cell growth | -12.87644028 | 21 |
| GO:0006875 | cellular metal ion homeostasis | -12.85578483 | 24 |
| GO:1902806 | regulation of cell cycle G1/S phase transition | -12.84838947 | 16 |
| GO:0007249 | I-kappaB kinase/NF-kappaB signaling | -12.82245119 | 18 |
| GO:0050714 | positive regulation of protein secretion | -12.81568516 | 16 |
| GO:0050806 | positive regulation of synaptic transmission | -12.81158209 | 15 |
| GO:0071456 | cellular response to hypoxia | -12.78315576 | 16 |
| GO:0050863 | regulation of T cell activation | -12.77315242 | 19 |
| GO:0042509 | regulation of tyrosine phosphorylation of STAT protein | -12.75388498 | 12 |
| GO:0032768 | regulation of monooxygenase activity | -12.75275294 | 11 |
| GO:2000351 | regulation of endothelial cell apoptotic process | -12.75275294 | 11 |
| GO:0030879 | mammary gland development | -12.72740697 | 14 |
| GO:0006352 | DNA-templated transcription, initiation | -12.70566764 | 17 |
| GO:0014068 | positive regulation of phosphatidylinositol 3-kinase signaling | -12.69245688 | 12 |
| GO:0050818 | regulation of coagulation | -12.69245688 | 12 |
| GO:0043200 | response to amino acid | -12.6778878 | 13 |
| GO:1902105 | regulation of leukocyte differentiation | -12.66850986 | 18 |
| GO:0090276 | regulation of peptide hormone secretion | -12.62307215 | 16 |
| GO:0043122 | regulation of I-kappaB kinase/NF-kappaB signaling | -12.62097771 | 17 |
| GO:0033135 | regulation of peptidyl-serine phosphorylation | -12.60086315 | 14 |
| GO:0007260 | tyrosine phosphorylation of STAT protein | -12.57189165 | 12 |
| GO:0002683 | negative regulation of immune system process | -12.56135002 | 22 |
| GO:0016049 | cell growth | -12.54372213 | 22 |
| GO:0045428 | regulation of nitric oxide biosynthetic process | -12.52576742 | 11 |
| GO:0036294 | cellular response to decreased oxygen levels | -12.46711035 | 16 |
| GO:0046660 | female sex differentiation | -12.43328824 | 13 |
| GO:0006631 | fatty acid metabolic process | -12.415898 | 20 |
| GO:0014074 | response to purine-containing compound | -12.39625439 | 14 |
| GO:0072577 | endothelial cell apoptotic process | -12.38057794 | 11 |
| GO:0032944 | regulation of mononuclear cell proliferation | -12.37543054 | 16 |
| GO:0030336 | negative regulation of cell migration | -12.36545913 | 19 |
| GO:0051052 | regulation of DNA metabolic process | -12.33979336 | 21 |
| GO:0045834 | positive regulation of lipid metabolic process | -12.27707568 | 14 |
| GO:0042531 | positive regulation of tyrosine phosphorylation of STAT protein | -12.23994579 | 11 |
| GO:0050999 | regulation of nitric-oxide synthase activity | -12.2300816 | 10 |
| GO:0070372 | regulation of ERK1 and ERK2 cascade | -12.17990928 | 18 |
| GO:0031348 | negative regulation of defense response | -12.1614144 | 17 |
| GO:0035265 | organ growth | -12.14363466 | 15 |
| GO:2001243 | negative regulation of intrinsic apoptotic signaling pathway | -12.11767755 | 12 |
| GO:2000134 | negative regulation of G1/S transition of mitotic cell cycle | -12.10928834 | 13 |
| GO:0040013 | negative regulation of locomotion | -12.09888698 | 20 |
| GO:0001894 | tissue homeostasis | -12.07954698 | 16 |
| GO:0001503 | ossification | -12.07954186 | 20 |
| GO:0042108 | positive regulation of cytokine biosynthetic process | -12.05740656 | 9 |
| GO:2000146 | negative regulation of cell motility | -12.0434266 | 19 |
| GO:1905475 | regulation of protein localization to membrane | -12.01240682 | 15 |
| GO:0042098 | T cell proliferation | -11.98006644 | 15 |
| GO:1902107 | positive regulation of leukocyte differentiation | -11.97148877 | 14 |
| GO:0007050 | cell cycle arrest | -11.96516734 | 16 |
| GO:0045444 | fat cell differentiation | -11.93691156 | 16 |
| GO:1901361 | organic cyclic compound catabolic process | -11.90013379 | 26 |
| GO:1902807 | negative regulation of cell cycle G1/S phase transition | -11.8470189 | 13 |
| GO:0045073 | regulation of chemokine biosynthetic process | -11.81594573 | 7 |
| GO:0043270 | positive regulation of ion transport | -11.78214812 | 17 |
| GO:0001655 | urogenital system development | -11.74735883 | 18 |
| GO:0010564 | regulation of cell cycle process | -11.72561752 | 26 |
| GO:0002763 | positive regulation of myeloid leukocyte differentiation | -11.72519169 | 10 |
| GO:0000082 | G1/S transition of mitotic cell cycle | -11.70899452 | 17 |
| GO:0042445 | hormone metabolic process | -11.68847107 | 16 |
| GO:0030100 | regulation of endocytosis | -11.68480084 | 17 |
| GO:0006469 | negative regulation of protein kinase activity | -11.66149765 | 16 |
| GO:0031663 | lipopolysaccharide-mediated signaling pathway | -11.64676945 | 10 |
| GO:0042593 | glucose homeostasis | -11.63464655 | 16 |
| GO:1902042 | negative regulation of extrinsic apoptotic signaling pathway via death domain receptors | -11.6166459 | 9 |
| GO:0033500 | carbohydrate homeostasis | -11.60791676 | 16 |
| GO:0071383 | cellular response to steroid hormone stimulus | -11.60570263 | 15 |
| GO:0034644 | cellular response to UV | -11.59646112 | 11 |
| GO:0030856 | regulation of epithelial cell differentiation | -11.57768789 | 14 |
| GO:0042033 | chemokine biosynthetic process | -11.56825897 | 7 |
| GO:0050755 | chemokine metabolic process | -11.56825897 | 7 |
| GO:0033138 | positive regulation of peptidyl-serine phosphorylation | -11.55601298 | 12 |
| GO:0006109 | regulation of carbohydrate metabolic process | -11.54564829 | 15 |
| GO:0034764 | positive regulation of transmembrane transport | -11.54564829 | 15 |
| GO:0048143 | astrocyte activation | -11.54275605 | 8 |
| GO:0048708 | astrocyte differentiation | -11.53695419 | 11 |
| GO:0045930 | negative regulation of mitotic cell cycle | -11.53083107 | 18 |
| GO:0070555 | response to interleukin-1 | -11.51585939 | 15 |
| GO:1902041 | regulation of extrinsic apoptotic signaling pathway via death domain receptors | -11.49429585 | 10 |
| GO:1901568 | fatty acid derivative metabolic process | -11.47511747 | 14 |
| GO:0030073 | insulin secretion | -11.45675038 | 15 |
| GO:0045926 | negative regulation of growth | -11.4500299 | 16 |
| GO:0010506 | regulation of autophagy | -11.38350102 | 18 |
| GO:0048872 | homeostasis of number of cells | -11.37264524 | 16 |
| GO:0051271 | negative regulation of cellular component movement | -11.34712244 | 19 |
| GO:0009615 | response to virus | -11.32138636 | 18 |
| GO:0007611 | learning or memory | -11.29626658 | 16 |
| GO:0072507 | divalent inorganic cation homeostasis | -11.29264737 | 21 |
| GO:0038127 | ERBB signaling pathway | -11.28307569 | 13 |
| GO:0050670 | regulation of lymphocyte proliferation | -11.28306646 | 15 |
| GO:0070374 | positive regulation of ERK1 and ERK2 cascade | -11.28306646 | 15 |
| GO:0006874 | cellular calcium ion homeostasis | -11.27777496 | 20 |
| GO:0060135 | maternal process involved in female pregnancy | -11.27575737 | 10 |
| GO:0032642 | regulation of chemokine production | -11.25086894 | 11 |
| GO:0022411 | cellular component disassembly | -11.2389886 | 22 |
| GO:0002696 | positive regulation of leukocyte activation | -11.23475708 | 19 |
| GO:0045429 | positive regulation of nitric oxide biosynthetic process | -11.22252609 | 9 |
| GO:0001959 | regulation of cytokine-mediated signaling pathway | -11.21085665 | 14 |
| GO:0044843 | cell cycle G1/S phase transition | -11.17526865 | 17 |
| GO:0008406 | gonad development | -11.17019937 | 15 |
| GO:1904407 | positive regulation of nitric oxide metabolic process | -11.13019479 | 9 |
| GO:0035994 | response to muscle stretch | -11.12831135 | 7 |
| GO:0030522 | intracellular receptor signaling pathway | -11.09733011 | 16 |
| GO:0033673 | negative regulation of kinase activity | -11.09733011 | 16 |
| GO:0097530 | granulocyte migration | -11.09602611 | 13 |
| GO:0002064 | epithelial cell development | -11.08702189 | 15 |
| GO:0061138 | morphogenesis of a branching epithelium | -11.08349291 | 14 |
| GO:0055074 | calcium ion homeostasis | -11.07446375 | 20 |
| GO:0042035 | regulation of cytokine biosynthetic process | -11.06835135 | 10 |
| GO:0006953 | acute-phase response | -11.0401053 | 9 |
| GO:0045137 | development of primary sexual characteristics | -11.00506861 | 15 |
| GO:0006690 | icosanoid metabolic process | -10.96790704 | 12 |
| GO:0021782 | glial cell development | -10.96790704 | 12 |
| GO:0050867 | positive regulation of cell activation | -10.96198034 | 19 |
| GO:0030810 | positive regulation of nucleotide biosynthetic process | -10.95215339 | 9 |
| GO:1900373 | positive regulation of purine nucleotide biosynthetic process | -10.95215339 | 9 |
| GO:1904646 | cellular response to amyloid-beta | -10.95215339 | 9 |
| GO:0002761 | regulation of myeloid leukocyte differentiation | -10.9255659 | 12 |
| GO:0051101 | regulation of DNA binding | -10.88360322 | 12 |
| GO:0006869 | lipid transport | -10.88358103 | 18 |
| GO:0034504 | protein localization to nucleus | -10.88135368 | 16 |
| GO:0032602 | chemokine production | -10.87921065 | 11 |
| GO:0009743 | response to carbohydrate | -10.8711115 | 15 |
| GO:0045861 | negative regulation of proteolysis | -10.84524863 | 18 |
| GO:0002690 | positive regulation of leukocyte chemotaxis | -10.82859686 | 11 |
| GO:0010660 | regulation of muscle cell apoptotic process | -10.82859686 | 11 |
| GO:0030168 | platelet activation | -10.81056128 | 13 |
| GO:0060759 | regulation of response to cytokine stimulus | -10.80766684 | 14 |
| GO:0001774 | microglial cell activation | -10.78228044 | 9 |
| GO:0002269 | leukocyte activation involved in inflammatory response | -10.78228044 | 9 |
| GO:1904707 | positive regulation of vascular smooth muscle cell proliferation | -10.78228044 | 9 |
| GO:0071621 | granulocyte chemotaxis | -10.75992304 | 12 |
| GO:0051767 | nitric-oxide synthase biosynthetic process | -10.74603303 | 7 |
| GO:0051769 | regulation of nitric-oxide synthase biosynthetic process | -10.74603303 | 7 |
| GO:0060965 | negative regulation of gene silencing by miRNA | -10.74603303 | 7 |
| GO:0042089 | cytokine biosynthetic process | -10.74462419 | 10 |
| GO:0032872 | regulation of stress-activated MAPK cascade | -10.71452918 | 15 |
| GO:0043467 | regulation of generation of precursor metabolites and energy | -10.70759014 | 13 |
| GO:0072503 | cellular divalent inorganic cation homeostasis | -10.68534818 | 20 |
| GO:0042107 | cytokine metabolic process | -10.68287519 | 10 |
| GO:0002673 | regulation of acute inflammatory response | -10.67373528 | 13 |
| GO:0001763 | morphogenesis of a branching structure | -10.66045361 | 14 |
| GO:0045907 | positive regulation of vasoconstriction | -10.64557628 | 8 |
| GO:0051054 | positive regulation of DNA metabolic process | -10.63787429 | 15 |
| GO:0070302 | regulation of stress-activated protein kinase signaling cascade | -10.63787429 | 15 |
| GO:0010657 | muscle cell apoptotic process | -10.63180815 | 11 |
| GO:0042116 | macrophage activation | -10.58396773 | 11 |
| GO:0043627 | response to estrogen | -10.56214207 | 10 |
| GO:0031334 | positive regulation of protein complex assembly | -10.56063656 | 16 |
| GO:0032409 | regulation of transporter activity | -10.56063656 | 16 |
| GO:1903524 | positive regulation of blood circulation | -10.50310563 | 10 |
| GO:0008630 | intrinsic apoptotic signaling pathway in response to DNA damage | -10.48983641 | 11 |
| GO:0032368 | regulation of lipid transport | -10.48346448 | 12 |
| GO:0030857 | negative regulation of epithelial cell differentiation | -10.46431825 | 9 |
| GO:0046683 | response to organophosphorus | -10.44527681 | 12 |
| GO:0005975 | carbohydrate metabolic process | -10.44383027 | 22 |
| GO:0043279 | response to alkaloid | -10.44352442 | 11 |
| GO:0016125 | sterol metabolic process | -10.44301733 | 13 |
| GO:0031669 | cellular response to nutrient levels | -10.43854322 | 15 |
| GO:0042176 | regulation of protein catabolic process | -10.43844023 | 18 |
| GO:0042554 | superoxide anion generation | -10.42814906 | 8 |
| GO:1901099 | negative regulation of signal transduction in absence of ligand | -10.42814906 | 8 |
| GO:2001240 | negative regulation of extrinsic apoptotic signaling pathway in absence of ligand | -10.42814906 | 8 |
| GO:0045080 | positive regulation of chemokine biosynthetic process | -10.41679365 | 6 |
| GO:0010888 | negative regulation of lipid storage | -10.4079906 | 7 |
| GO:0071229 | cellular response to acid chemical | -10.40547813 | 14 |
| GO:0043280 | positive regulation of cysteine-type endopeptidase activity involved in apoptotic process | -10.36983098 | 12 |
| GO:0006633 | fatty acid biosynthetic process | -10.34734556 | 13 |
| GO:0007162 | negative regulation of cell adhesion | -10.34204741 | 16 |
| GO:0010827 | regulation of glucose transmembrane transport | -10.33102552 | 10 |
| GO:0071260 | cellular response to mechanical stimulus | -10.33102552 | 10 |
| GO:0090322 | regulation of superoxide metabolic process | -10.32455598 | 8 |
| GO:0097242 | amyloid-beta clearance | -10.32455598 | 8 |
| GO:0010332 | response to gamma radiation | -10.31507194 | 9 |
| GO:0032091 | negative regulation of protein binding | -10.30748331 | 11 |
| GO:0003012 | muscle system process | -10.30219321 | 19 |
| GO:1903522 | regulation of blood circulation | -10.27807114 | 16 |
| GO:0007517 | muscle organ development | -10.26200587 | 18 |
| GO:0003158 | endothelium development | -10.25891621 | 12 |
| GO:0060149 | negative regulation of posttranscriptional gene silencing | -10.25257271 | 7 |
| GO:0060967 | negative regulation of gene silencing by RNA | -10.25257271 | 7 |
| GO:1903798 | regulation of production of miRNAs involved in gene silencing by miRNA | -10.25257271 | 7 |
| GO:1904996 | positive regulation of leukocyte adhesion to vascular endothelial cell | -10.25257271 | 7 |
| GO:0010742 | macrophage derived foam cell differentiation | -10.22410421 | 8 |
| GO:0090077 | foam cell differentiation | -10.22410421 | 8 |
| GO:0019915 | lipid storage | -10.22027309 | 10 |
| GO:0051251 | positive regulation of lymphocyte activation | -10.21197045 | 17 |
| GO:0051480 | regulation of cytosolic calcium ion concentration | -10.19307366 | 17 |
| GO:0010469 | regulation of signaling receptor activity | -10.1919166 | 13 |
| GO:0007566 | embryo implantation | -10.17165493 | 9 |
| GO:0045598 | regulation of fat cell differentiation | -10.15059997 | 12 |
| GO:0002674 | negative regulation of acute inflammatory response | -10.15010742 | 6 |
| GO:1904659 | glucose transmembrane transport | -10.13250373 | 11 |
| GO:0043525 | positive regulation of neuron apoptotic process | -10.10199711 | 9 |
| GO:0045981 | positive regulation of nucleotide metabolic process | -10.10199711 | 9 |
| GO:1900544 | positive regulation of purine nucleotide metabolic process | -10.10199711 | 9 |
| GO:0050796 | regulation of insulin secretion | -10.10097122 | 13 |
| GO:0051346 | negative regulation of hydrolase activity | -10.06551619 | 19 |
| GO:0070838 | divalent metal ion transport | -10.06551619 | 19 |
| GO:0045913 | positive regulation of carbohydrate metabolic process | -10.05968799 | 10 |
| GO:0032963 | collagen metabolic process | -10.04760539 | 11 |
| GO:0019229 | regulation of vasoconstriction | -10.03364018 | 9 |
| GO:1903428 | positive regulation of reactive oxygen species biosynthetic process | -10.03364018 | 9 |
| GO:0010950 | positive regulation of endopeptidase activity | -10.01168762 | 13 |
| GO:0043154 | negative regulation of cysteine-type endopeptidase activity involved in apoptotic process | -10.00756329 | 10 |
| GO:0030098 | lymphocyte differentiation | -10.00736862 | 17 |
| GO:0008645 | hexose transmembrane transport | -10.00577603 | 11 |
| GO:1990748 | cellular detoxification | -10.00577603 | 11 |
| GO:0071901 | negative regulation of protein serine/threonine kinase activity | -9.975545415 | 12 |
| GO:1900542 | regulation of purine nucleotide metabolic process | -9.975545415 | 12 |
| GO:0072511 | divalent inorganic cation transport | -9.973274601 | 19 |
| GO:0032722 | positive regulation of chemokine production | -9.966537783 | 9 |
| GO:0070920 | regulation of production of small RNA involved in gene silencing by RNA | -9.964522477 | 7 |
| GO:0043524 | negative regulation of neuron apoptotic process | -9.941320667 | 12 |
| GO:0071236 | cellular response to antibiotic | -9.941320667 | 12 |
| GO:0006816 | calcium ion transport | -9.940166325 | 18 |
| GO:0015749 | monosaccharide transmembrane transport | -9.923319338 | 11 |
| GO:1903799 | negative regulation of production of miRNAs involved in gene silencing by miRNA | -9.909228297 | 6 |
| GO:0007204 | positive regulation of cytosolic calcium ion concentration | -9.908686592 | 16 |
| GO:0035264 | multicellular organism growth | -9.907349445 | 12 |
| GO:0070665 | positive regulation of leukocyte proliferation | -9.907349445 | 12 |
| GO:0045927 | positive regulation of growth | -9.903043139 | 15 |
| GO:0071478 | cellular response to radiation | -9.866424619 | 13 |
| GO:0007569 | cell aging | -9.842417552 | 11 |
| GO:0034219 | carbohydrate transmembrane transport | -9.842417552 | 11 |
| GO:0006140 | regulation of nucleotide metabolic process | -9.840153641 | 12 |
| GO:0015980 | energy derivation by oxidation of organic compounds | -9.815103654 | 15 |
| GO:0016202 | regulation of striated muscle tissue development | -9.806922267 | 12 |
| GO:0042113 | B cell activation | -9.790757435 | 16 |
| GO:0008203 | cholesterol metabolic process | -9.773930833 | 12 |
| GO:0045807 | positive regulation of endocytosis | -9.773930833 | 12 |
| GO:2001056 | positive regulation of cysteine-type endopeptidase activity | -9.773930833 | 12 |
| GO:1900371 | regulation of purine nucleotide biosynthetic process | -9.763015981 | 11 |
| GO:0006919 | activation of cysteine-type endopeptidase activity involved in apoptotic process | -9.756710776 | 10 |
| GO:0070542 | response to fatty acid | -9.756710776 | 10 |
| GO:0007548 | sex differentiation | -9.750083736 | 15 |
| GO:0002444 | myeloid leukocyte mediated immunity | -9.747367442 | 20 |
| GO:0048754 | branching morphogenesis of an epithelial tube | -9.741176111 | 12 |
| GO:0060562 | epithelial tube morphogenesis | -9.732712853 | 16 |
| GO:0072521 | purine-containing compound metabolic process | -9.728618337 | 21 |
| GO:0030808 | regulation of nucleotide biosynthetic process | -9.723861465 | 11 |
| GO:1905953 | negative regulation of lipid localization | -9.70982844 | 9 |
| GO:1901861 | regulation of muscle tissue development | -9.708654942 | 12 |
| GO:0034349 | glial cell apoptotic process | -9.689538315 | 6 |
| GO:0032612 | interleukin-1 production | -9.685062769 | 11 |
| GO:0048634 | regulation of muscle organ development | -9.676364226 | 12 |
| GO:1902652 | secondary alcohol metabolic process | -9.676364226 | 12 |
| GO:0034103 | regulation of tissue remodeling | -9.66064597 | 10 |
| GO:0046824 | positive regulation of nucleocytoplasmic transport | -9.648383207 | 9 |
| GO:0050729 | positive regulation of inflammatory response | -9.644300926 | 12 |
| GO:0044089 | positive regulation of cellular component biogenesis | -9.639105687 | 20 |
| GO:0014706 | striated muscle tissue development | -9.618473565 | 17 |
| GO:0006694 | steroid biosynthetic process | -9.615440306 | 13 |
| GO:2000117 | negative regulation of cysteine-type endopeptidase activity | -9.61347384 | 10 |
| GO:0014002 | astrocyte development | -9.59536156 | 8 |
| GO:0010575 | positive regulation of vascular endothelial growth factor production | -9.579598788 | 7 |
| GO:0032770 | positive regulation of monooxygenase activity | -9.579598788 | 7 |
| GO:0008637 | apoptotic mitochondrial changes | -9.570741654 | 11 |
| GO:0060485 | mesenchyme development | -9.559647696 | 15 |
| GO:0040014 | regulation of multicellular organism growth | -9.528527043 | 9 |
| GO:0051129 | negative regulation of cellular component organization | -9.525258583 | 23 |
| GO:0010721 | negative regulation of cell development | -9.506408719 | 16 |
| GO:1905477 | positive regulation of protein localization to membrane | -9.496199395 | 11 |
| GO:0048662 | negative regulation of smooth muscle cell proliferation | -9.470052902 | 9 |
| GO:0120162 | positive regulation of cold-induced thermogenesis | -9.430225936 | 10 |
| GO:0071482 | cellular response to light stimulus | -9.386785346 | 11 |
| GO:0071674 | mononuclear cell migration | -9.385715108 | 10 |
| GO:0046890 | regulation of lipid biosynthetic process | -9.37688979 | 13 |
| GO:0042129 | regulation of T cell proliferation | -9.36549219 | 12 |
| GO:2001239 | regulation of extrinsic apoptotic signaling pathway in absence of ligand | -9.358802532 | 8 |
| GO:0051924 | regulation of calcium ion transport | -9.335923616 | 14 |
| GO:0034250 | positive regulation of cellular amide metabolic process | -9.335548409 | 12 |
| GO:0019318 | hexose metabolic process | -9.313542418 | 14 |
| GO:0031649 | heat generation | -9.300679153 | 6 |
| GO:0060537 | muscle tissue development | -9.286313215 | 17 |
| GO:0098754 | detoxification | -9.280128942 | 11 |
| GO:0090257 | regulation of muscle system process | -9.269077275 | 14 |
| GO:0032637 | interleukin-8 production | -9.255129993 | 10 |
| GO:0002697 | regulation of immune effector process | -9.254850989 | 18 |
| GO:0043393 | regulation of protein binding | -9.249326948 | 13 |
| GO:0055024 | regulation of cardiac muscle tissue development | -9.212550103 | 10 |
| GO:0046887 | positive regulation of hormone secretion | -9.210493105 | 11 |
| GO:1904036 | negative regulation of epithelial cell apoptotic process | -9.21008902 | 8 |
| GO:0006006 | glucose metabolic process | -9.199243354 | 13 |
| GO:0050870 | positive regulation of T cell activation | -9.199243354 | 13 |
| GO:0050671 | positive regulation of lymphocyte proliferation | -9.176102153 | 11 |
| GO:0098869 | cellular oxidant detoxification | -9.17042825 | 10 |
| GO:0051099 | positive regulation of binding | -9.15995295 | 12 |
| GO:0032946 | positive regulation of mononuclear cell proliferation | -9.141990059 | 11 |
| GO:0048146 | positive regulation of fibroblast proliferation | -9.138196898 | 8 |
| GO:0019439 | aromatic compound catabolic process | -9.138072093 | 22 |
| GO:0007187 | G protein-coupled receptor signaling pathway, coupled to cyclic nucleotide second messenger | -9.116488588 | 14 |
| GO:1901617 | organic hydroxy compound biosynthetic process | -9.116488588 | 14 |
| GO:0050680 | negative regulation of epithelial cell proliferation | -9.074666985 | 12 |
| GO:0010883 | regulation of lipid storage | -9.067855753 | 8 |
| GO:0046902 | regulation of mitochondrial membrane permeability | -9.0335147 | 9 |
| GO:1901987 | regulation of cell cycle phase transition | -9.024066552 | 18 |
| GO:0015850 | organic hydroxy compound transport | -9.010286827 | 14 |
| GO:0032611 | interleukin-1 beta production | -9.006339778 | 10 |
| GO:0032731 | positive regulation of interleukin-1 beta production | -8.999001843 | 8 |
| GO:0061756 | leukocyte adhesion to vascular endothelial cell | -8.999001843 | 8 |
| GO:0002275 | myeloid cell activation involved in immune response | -8.987770523 | 19 |
| GO:0043900 | regulation of multi-organism process | -8.98775474 | 17 |
| GO:0034765 | regulation of ion transmembrane transport | -8.981791171 | 18 |
| GO:0098542 | defense response to other organism | -8.9772304 | 20 |
| GO:0032930 | positive regulation of superoxide anion generation | -8.964094063 | 6 |
| GO:0060749 | mammary gland alveolus development | -8.964094063 | 6 |
| GO:0061377 | mammary gland lobule development | -8.964094063 | 6 |
| GO:1902176 | negative regulation of oxidative stress-induced intrinsic apoptotic signaling pathway | -8.964094063 | 6 |
| GO:0071346 | cellular response to interferon-gamma | -8.936045241 | 12 |
| GO:0042759 | long-chain fatty acid biosynthetic process | -8.935348031 | 7 |
| GO:0106106 | cold-induced thermogenesis | -8.910670822 | 11 |
| GO:0120161 | regulation of cold-induced thermogenesis | -8.910670822 | 11 |
| GO:0071347 | cellular response to interleukin-1 | -8.908831107 | 12 |
| GO:0032388 | positive regulation of intracellular transport | -8.88600761 | 13 |
| GO:1900182 | positive regulation of protein localization to nucleus | -8.882578087 | 9 |
| GO:0008286 | insulin receptor signaling pathway | -8.878645418 | 11 |
| GO:0043254 | regulation of protein complex assembly | -8.870538464 | 18 |
| GO:0051353 | positive regulation of oxidoreductase activity | -8.865519745 | 8 |
| GO:0090407 | organophosphate biosynthetic process | -8.851408663 | 21 |
| GO:0022898 | regulation of transmembrane transporter activity | -8.844957757 | 14 |
| GO:1901030 | positive regulation of mitochondrial outer membrane permeabilization involved in apoptotic signaling pathway | -8.840626653 | 7 |
| GO:0032652 | regulation of interleukin-1 production | -8.81049968 | 10 |
| GO:0007160 | cell-matrix adhesion | -8.793673731 | 13 |
| GO:0001701 | in utero embryonic development | -8.78064044 | 16 |
| GO:0043123 | positive regulation of I-kappaB kinase/NF-kappaB signaling | -8.775207835 | 12 |
| GO:0032386 | regulation of intracellular transport | -8.762761396 | 17 |
| GO:0001893 | maternal placenta development | -8.748910674 | 7 |
| GO:0034405 | response to fluid shear stress | -8.748910674 | 7 |
| GO:2000352 | negative regulation of endothelial cell apoptotic process | -8.748910674 | 7 |
| GO:0009165 | nucleotide biosynthetic process | -8.748393252 | 16 |
| GO:0007188 | adenylate cyclase-modulating G protein-coupled receptor signaling pathway | -8.748180567 | 13 |
| GO:0022617 | extracellular matrix disassembly | -8.737757329 | 9 |
| GO:0001541 | ovarian follicle development | -8.737312536 | 8 |
| GO:0042743 | hydrogen peroxide metabolic process | -8.737312536 | 8 |
| GO:0045840 | positive regulation of mitotic nuclear division | -8.737312536 | 8 |
| GO:0006606 | protein import into nucleus | -8.722111869 | 11 |
| GO:1901990 | regulation of mitotic cell cycle phase transition | -8.704266302 | 17 |
| GO:1901293 | nucleoside phosphate biosynthetic process | -8.700384055 | 16 |
| GO:0008643 | carbohydrate transport | -8.691500658 | 11 |
| GO:1900408 | negative regulation of cellular response to oxidative stress | -8.675063609 | 8 |
| GO:1903202 | negative regulation of oxidative stress-induced cell death | -8.675063609 | 8 |
| GO:0042692 | muscle cell differentiation | -8.668616045 | 16 |
| GO:0016999 | antibiotic metabolic process | -8.661113867 | 11 |
| GO:0046620 | regulation of organ growth | -8.660654536 | 10 |
| GO:1904994 | regulation of leukocyte adhesion to vascular endothelial cell | -8.660018617 | 7 |
| GO:2001171 | positive regulation of ATP biosynthetic process | -8.660018617 | 7 |
| GO:0045844 | positive regulation of striated muscle tissue development | -8.644379802 | 9 |
| GO:0048636 | positive regulation of muscle organ development | -8.644379802 | 9 |
| GO:0018107 | peptidyl-threonine phosphorylation | -8.624084436 | 10 |
| GO:0009117 | nucleotide metabolic process | -8.622945042 | 21 |
| GO:0006636 | unsaturated fatty acid biosynthetic process | -8.613990759 | 8 |
| GO:0071385 | cellular response to glucocorticoid stimulus | -8.613990759 | 8 |
| GO:1901863 | positive regulation of muscle tissue development | -8.598594914 | 9 |
| GO:0010821 | regulation of mitochondrion organization | -8.59466968 | 12 |
| GO:0033559 | unsaturated fatty acid metabolic process | -8.587858135 | 10 |
| GO:0032885 | regulation of polysaccharide biosynthetic process | -8.573785014 | 7 |
| GO:0002262 | myeloid cell homeostasis | -8.571269842 | 11 |
| GO:1990845 | adaptive thermogenesis | -8.571269842 | 11 |
| GO:0032732 | positive regulation of interleukin-1 production | -8.55405176 | 8 |
| GO:0050994 | regulation of lipid catabolic process | -8.55405176 | 8 |
| GO:1902883 | negative regulation of response to oxidative stress | -8.55405176 | 8 |
| GO:0006753 | nucleoside phosphate metabolic process | -8.548607503 | 21 |
| GO:0045088 | regulation of innate immune response | -8.546521957 | 17 |
| GO:0072657 | protein localization to membrane | -8.545866221 | 20 |
| GO:0032928 | regulation of superoxide anion generation | -8.531234793 | 6 |
| GO:0055093 | response to hyperoxia | -8.531234793 | 6 |
| GO:0006163 | purine nucleotide metabolic process | -8.52519666 | 19 |
| GO:0046700 | heterocycle catabolic process | -8.517018626 | 21 |
| GO:0010822 | positive regulation of mitochondrion organization | -8.516412916 | 10 |
| GO:0090559 | regulation of membrane permeability | -8.508760203 | 9 |
| GO:0034976 | response to endoplasmic reticulum stress | -8.492118803 | 14 |
| GO:0051193 | regulation of cofactor metabolic process | -8.481182379 | 10 |
| GO:0005996 | monosaccharide metabolic process | -8.45444417 | 14 |
| GO:0090316 | positive regulation of intracellular protein transport | -8.45444165 | 11 |
| GO:0042752 | regulation of circadian rhythm | -8.446272393 | 10 |
| GO:0045446 | endothelial cell differentiation | -8.446272393 | 10 |
| GO:0071384 | cellular response to corticosteroid stimulus | -8.437417448 | 8 |
| GO:2001244 | positive regulation of intrinsic apoptotic signaling pathway | -8.437417448 | 8 |
| GO:0051146 | striated muscle cell differentiation | -8.435716008 | 14 |
| GO:0034341 | response to interferon-gamma | -8.421254446 | 12 |
| GO:1990266 | neutrophil migration | -8.411677514 | 10 |
| GO:2000273 | positive regulation of signaling receptor activity | -8.408700597 | 7 |
| GO:0046849 | bone remodeling | -8.378143044 | 9 |
| GO:0006913 | nucleocytoplasmic transport | -8.374604635 | 15 |
| GO:0035821 | modification of morphology or physiology of other organism | -8.368947842 | 11 |
| GO:1901991 | negative regulation of mitotic cell cycle phase transition | -8.357605398 | 13 |
| GO:0022612 | gland morphogenesis | -8.343411988 | 10 |
| GO:1903578 | regulation of ATP metabolic process | -8.343411988 | 10 |
| GO:0042533 | tumor necrosis factor biosynthetic process | -8.32958362 | 7 |
| GO:0042534 | regulation of tumor necrosis factor biosynthetic process | -8.32958362 | 7 |
| GO:0051169 | nuclear transport | -8.325239304 | 15 |
| GO:0006914 | autophagy | -8.319409714 | 18 |
| GO:0043299 | leukocyte degranulation | -8.319409714 | 18 |
| GO:0061919 | process utilizing autophagic mechanism | -8.319409714 | 18 |
| GO:0046165 | alcohol biosynthetic process | -8.31292638 | 11 |
| GO:0018210 | peptidyl-threonine modification | -8.309731124 | 10 |
| GO:0014909 | smooth muscle cell migration | -8.293674647 | 9 |
| GO:0032677 | regulation of interleukin-8 production | -8.293674647 | 9 |
| GO:0006164 | purine nucleotide biosynthetic process | -8.288445517 | 14 |
| GO:0050995 | negative regulation of lipid catabolic process | -8.279331954 | 6 |
| GO:0034284 | response to monosaccharide | -8.277902212 | 12 |
| GO:1900180 | regulation of protein localization to nucleus | -8.276344922 | 10 |
| GO:0006940 | regulation of smooth muscle contraction | -8.270034932 | 8 |
| GO:0010573 | vascular endothelial growth factor production | -8.270034932 | 8 |
| GO:0060969 | negative regulation of gene silencing | -8.252590224 | 7 |
| GO:0014013 | regulation of gliogenesis | -8.243248585 | 10 |
| GO:0009408 | response to heat | -8.230307219 | 11 |
| GO:2000378 | negative regulation of reactive oxygen species metabolic process | -8.216127431 | 8 |
| GO:0010951 | negative regulation of endopeptidase activity | -8.214215917 | 13 |
| GO:0097194 | execution phase of apoptosis | -8.211185355 | 9 |
| GO:0050769 | positive regulation of neurogenesis | -8.190785046 | 17 |
| GO:0046688 | response to copper ion | -8.177612007 | 7 |
| GO:0150077 | regulation of neuroinflammatory response | -8.177612007 | 7 |
| GO:0042180 | cellular ketone metabolic process | -8.174059054 | 13 |
| GO:0002532 | production of molecular mediator involved in inflammatory response | -8.17065569 | 9 |
| GO:0050918 | positive chemotaxis | -8.163113003 | 8 |
| GO:0010869 | regulation of receptor biosynthetic process | -8.16230271 | 6 |
| GO:0051962 | positive regulation of nervous system development | -8.159155627 | 18 |
| GO:0002764 | immune response-regulating signaling pathway | -8.153769893 | 20 |
| GO:0007589 | body fluid secretion | -8.130588891 | 9 |
| GO:0042100 | B cell proliferation | -8.130588891 | 9 |
| GO:1901184 | regulation of ERBB signaling pathway | -8.130588891 | 9 |
| GO:0051170 | import into nucleus | -8.122689561 | 11 |
| GO:0045123 | cellular extravasation | -8.110963625 | 8 |
| GO:0032881 | regulation of polysaccharide metabolic process | -8.104548679 | 7 |
| GO:0051701 | interaction with host | -8.09384641 | 12 |
| GO:0072522 | purine-containing compound biosynthetic process | -8.09304058 | 14 |
| GO:0032651 | regulation of interleukin-1 beta production | -8.090975032 | 9 |
| GO:0048010 | vascular endothelial growth factor receptor signaling pathway | -8.090975032 | 9 |
| GO:2001169 | regulation of ATP biosynthetic process | -8.090975032 | 9 |
| GO:0033209 | tumor necrosis factor-mediated signaling pathway | -8.06993055 | 11 |
| GO:0001885 | endothelial cell development | -8.059652575 | 8 |
| GO:0002637 | regulation of immunoglobulin production | -8.059652575 | 8 |
| GO:0032370 | positive regulation of lipid transport | -8.059652575 | 8 |
| GO:0043255 | regulation of carbohydrate biosynthetic process | -8.051804505 | 9 |
| GO:0097327 | response to antineoplastic agent | -8.051804505 | 9 |
| GO:0019430 | removal of superoxide radicals | -8.05051636 | 6 |
| GO:0034754 | cellular hormone metabolic process | -8.050503114 | 10 |
| GO:0010623 | programmed cell death involved in cell development | -8.042025431 | 5 |
| GO:0010745 | negative regulation of macrophage derived foam cell differentiation | -8.042025431 | 5 |
| GO:1901028 | regulation of mitochondrial outer membrane permeabilization involved in apoptotic signaling pathway | -8.033307273 | 7 |
| GO:0072594 | establishment of protein localization to organelle | -8.027064059 | 18 |
| GO:0070301 | cellular response to hydrogen peroxide | -8.013068001 | 9 |
| GO:0051188 | cofactor biosynthetic process | -8.006794931 | 14 |
| GO:1901988 | negative regulation of cell cycle phase transition | -7.978421934 | 13 |
| GO:0051785 | positive regulation of nuclear division | -7.959444601 | 8 |
| GO:0010720 | positive regulation of cell development | -7.94461798 | 18 |
| GO:0010466 | negative regulation of peptidase activity | -7.940287481 | 13 |
| GO:0006909 | phagocytosis | -7.93174875 | 15 |
| GO:0030593 | neutrophil chemotaxis | -7.899373853 | 9 |
| GO:0042572 | retinol metabolic process | -7.895950863 | 7 |
| GO:0045776 | negative regulation of blood pressure | -7.895950863 | 7 |
| GO:0070227 | lymphocyte apoptotic process | -7.862298435 | 8 |
| GO:0044770 | cell cycle phase transition | -7.855906808 | 19 |
| GO:0030194 | positive regulation of blood coagulation | -7.840950059 | 6 |
| GO:0045932 | negative regulation of muscle contraction | -7.840950059 | 6 |
| GO:0071450 | cellular response to oxygen radical | -7.840950059 | 6 |
| GO:0071451 | cellular response to superoxide | -7.840950059 | 6 |
| GO:1900048 | positive regulation of hemostasis | -7.840950059 | 6 |
| GO:0001889 | liver development | -7.83738647 | 10 |
| GO:0009409 | response to cold | -7.829680652 | 7 |
| GO:1903580 | positive regulation of ATP metabolic process | -7.829680652 | 7 |
| GO:0051881 | regulation of mitochondrial membrane potential | -7.814818461 | 8 |
| GO:0031330 | negative regulation of cellular catabolic process | -7.790896496 | 13 |
| GO:0014812 | muscle cell migration | -7.789277432 | 9 |
| GO:0019233 | sensory perception of pain | -7.789277432 | 9 |
| GO:0048568 | embryonic organ development | -7.781822935 | 16 |
| GO:0016485 | protein processing | -7.773338213 | 14 |
| GO:0035924 | cellular response to vascular endothelial growth factor stimulus | -7.768039732 | 8 |
| GO:0050805 | negative regulation of synaptic transmission | -7.768039732 | 8 |
| GO:0044270 | cellular nitrogen compound catabolic process | -7.76631149 | 20 |
| GO:0006692 | prostanoid metabolic process | -7.764920919 | 7 |
| GO:0006693 | prostaglandin metabolic process | -7.764920919 | 7 |
| GO:0035196 | production of miRNAs involved in gene silencing by miRNA | -7.764920919 | 7 |
| GO:0060078 | regulation of postsynaptic membrane potential | -7.749623323 | 10 |
| GO:0061008 | hepaticobiliary system development | -7.749623323 | 10 |
| GO:0048738 | cardiac muscle tissue development | -7.747807471 | 12 |
| GO:0036296 | response to increased oxygen levels | -7.742436413 | 6 |
| GO:0050820 | positive regulation of coagulation | -7.742436413 | 6 |
| GO:0061419 | positive regulation of transcription from RNA polymerase II promoter in response to hypoxia | -7.730538347 | 4 |
| GO:0002700 | regulation of production of molecular mediator of immune response | -7.720820139 | 10 |
| GO:0007612 | learning | -7.720820139 | 10 |
| GO:0001822 | kidney development | -7.71802509 | 13 |
| GO:0030195 | negative regulation of blood coagulation | -7.701606312 | 7 |
| GO:0050873 | brown fat cell differentiation | -7.701606312 | 7 |
| GO:0006939 | smooth muscle contraction | -7.682569696 | 9 |
| GO:0090594 | inflammatory response to wounding | -7.678270318 | 5 |
| GO:2001028 | positive regulation of endothelial cell chemotaxis | -7.678270318 | 5 |
| GO:0055123 | digestive system development | -7.66387142 | 10 |
| GO:0002253 | activation of immune response | -7.649144125 | 20 |
| GO:1901989 | positive regulation of cell cycle phase transition | -7.647719951 | 9 |
| GO:0000303 | response to superoxide | -7.647682753 | 6 |
| GO:0001844 | protein insertion into mitochondrial membrane involved in apoptotic signaling pathway | -7.647682753 | 6 |
| GO:0060055 | angiogenesis involved in wound healing | -7.647682753 | 6 |
| GO:0030308 | negative regulation of cell growth | -7.64732663 | 11 |
| GO:0046777 | protein autophosphorylation | -7.645190849 | 12 |
| GO:0061028 | establishment of endothelial barrier | -7.63967564 | 7 |
| GO:0071675 | regulation of mononuclear cell migration | -7.63967564 | 7 |
| GO:1900047 | negative regulation of hemostasis | -7.63967564 | 7 |
| GO:0010675 | regulation of cellular carbohydrate metabolic process | -7.635719736 | 10 |
| GO:0022408 | negative regulation of cell-cell adhesion | -7.62380274 | 11 |
| GO:0044772 | mitotic cell cycle phase transition | -7.582778131 | 18 |
| GO:0051205 | protein insertion into membrane | -7.579071523 | 7 |
| GO:0071622 | regulation of granulocyte chemotaxis | -7.579071523 | 7 |
| GO:0046822 | regulation of nucleocytoplasmic transport | -7.579059316 | 9 |
| GO:0072001 | renal system development | -7.558290843 | 13 |
| GO:0000305 | response to oxygen radical | -7.556417258 | 6 |
| GO:1904062 | regulation of cation transmembrane transport | -7.550941909 | 14 |
| GO:0062014 | negative regulation of small molecule metabolic process | -7.545235615 | 9 |
| GO:0044262 | cellular carbohydrate metabolic process | -7.523561313 | 13 |
| GO:0050768 | negative regulation of neurogenesis | -7.523561313 | 13 |
| GO:0051770 | positive regulation of nitric-oxide synthase biosynthetic process | -7.517653507 | 5 |
| GO:0010743 | regulation of macrophage derived foam cell differentiation | -7.468396655 | 6 |
| GO:0097421 | liver regeneration | -7.468396655 | 6 |
| GO:0031050 | dsRNA processing | -7.461630665 | 7 |
| GO:0032757 | positive regulation of interleukin-8 production | -7.461630665 | 7 |
| GO:0070918 | production of small RNA involved in gene silencing by RNA | -7.461630665 | 7 |
| GO:0031623 | receptor internalization | -7.445717756 | 9 |
| GO:0001937 | negative regulation of endothelial cell proliferation | -7.416868476 | 8 |
| GO:0030811 | regulation of nucleotide catabolic process | -7.416868476 | 8 |
| GO:0050819 | negative regulation of coagulation | -7.404695581 | 7 |
| GO:0097345 | mitochondrial outer membrane permeabilization | -7.404695581 | 7 |
| GO:0010948 | negative regulation of cell cycle process | -7.398363633 | 14 |
| GO:0000187 | activation of MAPK activity | -7.391517513 | 10 |
| GO:0010039 | response to iron ion | -7.383402343 | 6 |
| GO:0045986 | negative regulation of smooth muscle contraction | -7.368496084 | 5 |
| GO:0043271 | negative regulation of ion transport | -7.365352733 | 10 |
| GO:0050777 | negative regulation of immune response | -7.339372433 | 10 |
| GO:0030217 | T cell differentiation | -7.332675218 | 12 |
| GO:0002221 | pattern recognition receptor signaling pathway | -7.308337339 | 11 |
| GO:0042311 | vasodilation | -7.301237158 | 6 |
| GO:0051204 | protein insertion into mitochondrial membrane | -7.301237158 | 6 |
| GO:0001776 | leukocyte homeostasis | -7.29469152 | 8 |
| GO:0006970 | response to osmotic stress | -7.29469152 | 8 |
| GO:2000106 | regulation of leukocyte apoptotic process | -7.29469152 | 8 |
| GO:0071398 | cellular response to fatty acid | -7.294171173 | 7 |
| GO:0042594 | response to starvation | -7.286741396 | 11 |
| GO:0010906 | regulation of glucose metabolic process | -7.286025612 | 9 |
| GO:0007005 | mitochondrion organization | -7.280297936 | 17 |
| GO:0017038 | protein import | -7.265263767 | 11 |
| GO:0050866 | negative regulation of cell activation | -7.265263767 | 11 |
| GO:0006754 | ATP biosynthetic process | -7.262514804 | 10 |
| GO:0046034 | ATP metabolic process | -7.255162331 | 13 |
| GO:0010507 | negative regulation of autophagy | -7.255006125 | 8 |
| GO:0060291 | long-term synaptic potentiation | -7.255006125 | 8 |
| GO:0051048 | negative regulation of secretion | -7.22265879 | 11 |
| GO:0051194 | positive regulation of cofactor metabolic process | -7.221722648 | 6 |
| GO:0060251 | regulation of glial cell proliferation | -7.221722648 | 6 |
| GO:0032755 | positive regulation of interleukin-6 production | -7.215820125 | 8 |
| GO:0002703 | regulation of leukocyte mediated immunity | -7.201529154 | 11 |
| GO:0007173 | epidermal growth factor receptor signaling pathway | -7.193682671 | 9 |
| GO:0002446 | neutrophil mediated immunity | -7.166410929 | 16 |
| GO:0001952 | regulation of cell-matrix adhesion | -7.163451385 | 9 |
| GO:0007093 | mitotic cell cycle checkpoint | -7.162481194 | 10 |
| GO:0045742 | positive regulation of epidermal growth factor receptor signaling pathway | -7.144696774 | 6 |
| GO:0051961 | negative regulation of nervous system development | -7.142727213 | 13 |
| GO:0001892 | embryonic placenta development | -7.138899607 | 8 |
| GO:0042058 | regulation of epidermal growth factor receptor signaling pathway | -7.138899607 | 8 |
| GO:0043470 | regulation of carbohydrate catabolic process | -7.138899607 | 8 |
| GO:0001836 | release of cytochrome c from mitochondria | -7.136147024 | 7 |
| GO:0043388 | positive regulation of DNA binding | -7.136147024 | 7 |
| GO:0006805 | xenobiotic metabolic process | -7.133487384 | 9 |
| GO:0007254 | JNK cascade | -7.118137036 | 11 |
| GO:0016051 | carbohydrate biosynthetic process | -7.118137036 | 11 |
| GO:0002819 | regulation of adaptive immune response | -7.113472673 | 10 |
| GO:0043009 | chordate embryonic development | -7.111977386 | 18 |
| GO:0015711 | organic anion transport | -7.107700274 | 16 |
| GO:0006959 | humoral immune response | -7.107634564 | 14 |
| GO:0051899 | membrane depolarization | -7.101142747 | 8 |
| GO:0010574 | regulation of vascular endothelial growth factor production | -7.085396623 | 7 |
| GO:1902110 | positive regulation of mitochondrial membrane permeability involved in apoptotic process | -7.085396623 | 7 |
| GO:0002246 | wound healing involved in inflammatory response | -7.065610848 | 4 |
| GO:0090400 | stress-induced premature senescence | -7.065610848 | 4 |
| GO:0032635 | interleukin-6 production | -7.0651145 | 10 |
| GO:0002576 | platelet degranulation | -7.045155906 | 9 |
| GO:0030888 | regulation of B cell proliferation | -7.03555318 | 7 |
| GO:0055025 | positive regulation of cardiac muscle tissue development | -7.03555318 | 7 |
| GO:1905954 | positive regulation of lipid localization | -7.026982818 | 8 |
| GO:0000075 | cell cycle checkpoint | -7.016351568 | 11 |
| GO:0010661 | positive regulation of muscle cell apoptotic process | -6.997533376 | 6 |
| GO:0030224 | monocyte differentiation | -6.997533376 | 6 |
| GO:0032094 | response to food | -6.997533376 | 6 |
| GO:1901186 | positive regulation of ERBB signaling pathway | -6.997533376 | 6 |
| GO:1903131 | mononuclear cell differentiation | -6.997533376 | 6 |
| GO:0006937 | regulation of muscle contraction | -6.993762575 | 10 |
| GO:0009206 | purine ribonucleoside triphosphate biosynthetic process | -6.993762575 | 10 |
| GO:1901992 | positive regulation of mitotic cell cycle phase transition | -6.99055961 | 8 |
| GO:0031571 | mitotic G1 DNA damage checkpoint | -6.986586098 | 7 |
| GO:1902686 | mitochondrial outer membrane permeabilization involved in programmed cell death | -6.986586098 | 7 |
| GO:0034248 | regulation of cellular amide metabolic process | -6.980970974 | 16 |
| GO:0002544 | chronic inflammatory response | -6.975931982 | 5 |
| GO:1902004 | positive regulation of amyloid-beta formation | -6.975931982 | 5 |
| GO:0009145 | purine nucleoside triphosphate biosynthetic process | -6.970287181 | 10 |
| GO:0003073 | regulation of systemic arterial blood pressure | -6.954561369 | 8 |
| GO:1901888 | regulation of cell junction assembly | -6.954561369 | 8 |
| GO:0044783 | G1 DNA damage checkpoint | -6.938466303 | 7 |
| GO:0044819 | mitotic G1/S transition checkpoint | -6.938466303 | 7 |
| GO:0045453 | bone resorption | -6.938466303 | 7 |
| GO:0002699 | positive regulation of immune effector process | -6.936808003 | 11 |
| GO:0009792 | embryo development ending in birth or egg hatching | -6.934877215 | 18 |
| GO:0042770 | signal transduction in response to DNA damage | -6.930869168 | 9 |
| GO:1904706 | negative regulation of vascular smooth muscle cell proliferation | -6.927137588 | 6 |
| GO:0016241 | regulation of macroautophagy | -6.923788395 | 10 |
| GO:0051196 | regulation of coenzyme metabolic process | -6.918978838 | 8 |
| GO:0035794 | positive regulation of mitochondrial membrane permeability | -6.891166147 | 7 |
| GO:0006898 | receptor-mediated endocytosis | -6.881267213 | 13 |
| GO:0045732 | positive regulation of protein catabolic process | -6.878209627 | 11 |
| GO:2001020 | regulation of response to DNA damage stimulus | -6.878209627 | 11 |
| GO:0002705 | positive regulation of leukocyte mediated immunity | -6.87515156 | 9 |
| GO:0061042 | vascular wound healing | -6.859941985 | 5 |
| GO:0007190 | activation of adenylate cyclase activity | -6.858711227 | 6 |
| GO:0009201 | ribonucleoside triphosphate biosynthetic process | -6.855144119 | 10 |
| GO:0045833 | negative regulation of lipid metabolic process | -6.849025355 | 8 |
| GO:0034655 | nucleobase-containing compound catabolic process | -6.848555743 | 18 |
| GO:1902108 | regulation of mitochondrial membrane permeability involved in apoptotic process | -6.844659312 | 7 |
| GO:0009152 | purine ribonucleotide biosynthetic process | -6.836748011 | 12 |
| GO:0048598 | embryonic morphogenesis | -6.832867073 | 17 |
| GO:0030183 | B cell differentiation | -6.820345946 | 9 |
| GO:1903038 | negative regulation of leukocyte cell-cell adhesion | -6.820345946 | 9 |
| GO:0050810 | regulation of steroid biosynthetic process | -6.814637333 | 8 |
| GO:0009205 | purine ribonucleoside triphosphate metabolic process | -6.807310084 | 13 |
| GO:0048762 | mesenchymal cell differentiation | -6.801444399 | 11 |
| GO:0046626 | regulation of insulin receptor signaling pathway | -6.798920732 | 7 |
| GO:1900015 | regulation of cytokine production involved in inflammatory response | -6.798920732 | 7 |
| GO:1905710 | positive regulation of membrane permeability | -6.798920732 | 7 |
| GO:0038083 | peptidyl-tyrosine autophosphorylation | -6.792149962 | 6 |
| GO:0071548 | response to dexamethasone | -6.792149962 | 6 |
| GO:0042359 | vitamin D metabolic process | -6.750077305 | 5 |
| GO:0051000 | positive regulation of nitric-oxide synthase activity | -6.750077305 | 5 |
| GO:0019217 | regulation of fatty acid metabolic process | -6.746998048 | 8 |
| GO:0042102 | positive regulation of T cell proliferation | -6.746998048 | 8 |
| GO:0051604 | protein maturation | -6.742677461 | 14 |
| GO:0010907 | positive regulation of glucose metabolic process | -6.727357555 | 6 |
| GO:0009168 | purine ribonucleoside monophosphate biosynthetic process | -6.721665994 | 10 |
| GO:0046328 | regulation of JNK cascade | -6.721665994 | 10 |
| GO:0009199 | ribonucleoside triphosphate metabolic process | -6.720229153 | 13 |
| GO:0000079 | regulation of cyclin-dependent protein serine/threonine kinase activity | -6.713731261 | 8 |
| GO:1903076 | regulation of protein localization to plasma membrane | -6.713731261 | 8 |
| GO:0009144 | purine nucleoside triphosphate metabolic process | -6.705888361 | 13 |
| GO:0009167 | purine ribonucleoside monophosphate metabolic process | -6.69159614 | 13 |
| GO:0007006 | mitochondrial membrane organization | -6.687145915 | 9 |
| GO:0060079 | excitatory postsynaptic potential | -6.680823089 | 8 |
| GO:1901570 | fatty acid derivative biosynthetic process | -6.680823089 | 8 |
| GO:0009127 | purine nucleoside monophosphate biosynthetic process | -6.678256439 | 10 |
| GO:0038061 | NIK/NF-kappaB signaling | -6.678256439 | 10 |
| GO:0048639 | positive regulation of developmental growth | -6.678256439 | 10 |
| GO:0033861 | negative regulation of NAD(P)H oxidase activity | -6.673014491 | 3 |
| GO:0060557 | positive regulation of vitamin D biosynthetic process | -6.673014491 | 3 |
| GO:0060559 | positive regulation of calcidiol 1-monooxygenase activity | -6.673014491 | 3 |
| GO:0002534 | cytokine production involved in inflammatory response | -6.666080972 | 7 |
| GO:0042698 | ovulation cycle | -6.666080972 | 7 |
| GO:0050715 | positive regulation of cytokine secretion | -6.666080972 | 7 |
| GO:0045124 | regulation of bone resorption | -6.664245039 | 6 |
| GO:0009150 | purine ribonucleotide metabolic process | -6.662001521 | 16 |
| GO:0002285 | lymphocyte activation involved in immune response | -6.656748064 | 10 |
| GO:0051147 | regulation of muscle cell differentiation | -6.656748064 | 10 |
| GO:0009126 | purine nucleoside monophosphate metabolic process | -6.649008069 | 13 |
| GO:0031281 | positive regulation of cyclase activity | -6.645730818 | 5 |
| GO:1901522 | positive regulation of transcription from RNA polymerase II promoter involved in cellular response to chemical stimulus | -6.645730818 | 5 |
| GO:0010565 | regulation of cellular ketone metabolic process | -6.635368725 | 10 |
| GO:0006720 | isoprenoid metabolic process | -6.635325313 | 9 |
| GO:0033157 | regulation of intracellular protein transport | -6.634166291 | 11 |
| GO:0009260 | ribonucleotide biosynthetic process | -6.625542124 | 12 |
| GO:0014823 | response to activity | -6.623187098 | 7 |
| GO:0033627 | cell adhesion mediated by integrin | -6.623187098 | 7 |
| GO:0050795 | regulation of behavior | -6.623187098 | 7 |
| GO:0061180 | mammary gland epithelium development | -6.623187098 | 7 |
| GO:0002367 | cytokine production involved in immune response | -6.616054062 | 8 |
| GO:0009142 | nucleoside triphosphate biosynthetic process | -6.614117024 | 10 |
| GO:0048167 | regulation of synaptic plasticity | -6.592991588 | 10 |
| GO:0001660 | fever generation | -6.592567507 | 4 |
| GO:0002676 | regulation of chronic inflammatory response | -6.592567507 | 4 |
| GO:0032070 | regulation of deoxyribonuclease activity | -6.592567507 | 4 |
| GO:0045348 | positive regulation of MHC class II biosynthetic process | -6.592567507 | 4 |
| GO:0046631 | alpha-beta T cell activation | -6.584303058 | 9 |
| GO:0071156 | regulation of cell cycle arrest | -6.584179468 | 8 |
| GO:1904029 | regulation of cyclin-dependent protein kinase activity | -6.584179468 | 8 |
| GO:0007189 | adenylate cyclase-activating G protein-coupled receptor signaling pathway | -6.559084285 | 9 |
| GO:0055017 | cardiac muscle tissue growth | -6.552635997 | 8 |
| GO:0044321 | response to leptin | -6.546381785 | 5 |
| GO:0045821 | positive regulation of glycolytic process | -6.546381785 | 5 |
| GO:1902993 | positive regulation of amyloid precursor protein catabolic process | -6.546381785 | 5 |
| GO:2000637 | positive regulation of gene silencing by miRNA | -6.546381785 | 5 |
| GO:0034105 | positive regulation of tissue remodeling | -6.542735987 | 6 |
| GO:0051966 | regulation of synaptic transmission, glutamatergic | -6.53935768 | 7 |
| GO:0006936 | muscle contraction | -6.537507894 | 13 |
| GO:0031644 | regulation of neurological system process | -6.534056787 | 9 |
| GO:0031589 | cell-substrate adhesion | -6.523777065 | 13 |
| GO:0007219 | Notch signaling pathway | -6.50972577 | 10 |
| GO:0009259 | ribonucleotide metabolic process | -6.505049205 | 16 |
| GO:0046390 | ribose phosphate biosynthetic process | -6.5008952 | 12 |
| GO:0009161 | ribonucleoside monophosphate metabolic process | -6.496450354 | 13 |
| GO:0010863 | positive regulation of phospholipase C activity | -6.484191864 | 6 |
| GO:0002821 | positive regulation of adaptive immune response | -6.459929433 | 8 |
| GO:0046323 | glucose import | -6.458016583 | 7 |
| GO:0042535 | positive regulation of tumor necrosis factor biosynthetic process | -6.451580143 | 5 |
| GO:0046697 | decidualization | -6.451580143 | 5 |
| GO:0050927 | positive regulation of positive chemotaxis | -6.451580143 | 5 |
| GO:0060148 | positive regulation of posttranscriptional gene silencing | -6.451580143 | 5 |
| GO:2001026 | regulation of endothelial cell chemotaxis | -6.451580143 | 5 |
| GO:0009141 | nucleoside triphosphate metabolic process | -6.44232968 | 13 |
| GO:0002758 | innate immune response-activating signal transduction | -6.44000798 | 12 |
| GO:0048259 | regulation of receptor-mediated endocytosis | -6.429648452 | 8 |
| GO:0009156 | ribonucleoside monophosphate biosynthetic process | -6.428375811 | 10 |
| GO:0030574 | collagen catabolic process | -6.427031413 | 6 |
| GO:1900076 | regulation of cellular response to insulin stimulus | -6.418235989 | 7 |
| GO:0099565 | chemical synaptic transmission, postsynaptic | -6.399668458 | 8 |
| GO:0046886 | positive regulation of hormone biosynthetic process | -6.398311305 | 4 |
| GO:1903800 | positive regulation of production of miRNAs involved in gene silencing by miRNA | -6.398311305 | 4 |
| GO:0042119 | neutrophil activation | -6.381099202 | 15 |
| GO:0014015 | positive regulation of gliogenesis | -6.379027091 | 7 |
| GO:1900271 | regulation of long-term synaptic potentiation | -6.371192849 | 6 |
| GO:0001676 | long-chain fatty acid metabolic process | -6.369983917 | 8 |
| GO:0005976 | polysaccharide metabolic process | -6.369983917 | 8 |
| GO:2000278 | regulation of DNA biosynthetic process | -6.369983917 | 8 |
| GO:0002224 | toll-like receptor signaling pathway | -6.364004017 | 9 |
| GO:0030813 | positive regulation of nucleotide catabolic process | -6.360934225 | 5 |
| GO:0035902 | response to immobilization stress | -6.360934225 | 5 |
| GO:0042730 | fibrinolysis | -6.360934225 | 5 |
| GO:0050926 | regulation of positive chemotaxis | -6.360934225 | 5 |
| GO:0051197 | positive regulation of coenzyme metabolic process | -6.360934225 | 5 |
| GO:1900739 | regulation of protein insertion into mitochondrial membrane involved in apoptotic signaling pathway | -6.360934225 | 5 |
| GO:1900740 | positive regulation of protein insertion into mitochondrial membrane involved in apoptotic signaling pathway | -6.360934225 | 5 |
| GO:0009746 | response to hexose | -6.348863619 | 10 |
| GO:0007423 | sensory organ development | -6.343239486 | 16 |
| GO:0016101 | diterpenoid metabolic process | -6.34058945 | 8 |
| GO:0072332 | intrinsic apoptotic signaling pathway by p53 class mediator | -6.340374505 | 7 |
| GO:2000027 | regulation of animal organ morphogenesis | -6.337079851 | 11 |
| GO:0002686 | negative regulation of leukocyte migration | -6.316618441 | 6 |
| GO:0007595 | lactation | -6.316618441 | 6 |
| GO:0048512 | circadian behavior | -6.316618441 | 6 |
| GO:1900274 | regulation of phospholipase C activity | -6.316618441 | 6 |
| GO:0036230 | granulocyte activation | -6.316220718 | 15 |
| GO:0048640 | negative regulation of developmental growth | -6.31147982 | 8 |
| GO:0060419 | heart growth | -6.31147982 | 8 |
| GO:0019693 | ribose phosphate metabolic process | -6.303654224 | 16 |
| GO:0034329 | cell junction assembly | -6.303630047 | 11 |
| GO:0000271 | polysaccharide biosynthetic process | -6.302263459 | 7 |
| GO:0002757 | immune response-activating signal transduction | -6.302217045 | 17 |
| GO:0060964 | regulation of gene silencing by miRNA | -6.282649934 | 8 |
| GO:0045672 | positive regulation of osteoclast differentiation | -6.274101039 | 5 |
| GO:0032675 | regulation of interleukin-6 production | -6.270657543 | 9 |
| GO:0051607 | defense response to virus | -6.270478827 | 11 |
| GO:0006110 | regulation of glycolytic process | -6.264679761 | 7 |
| GO:0055021 | regulation of cardiac muscle tissue growth | -6.264679761 | 7 |
| GO:1901224 | positive regulation of NIK/NF-kappaB signaling | -6.264679761 | 7 |
| GO:0007622 | rhythmic behavior | -6.263254161 | 6 |
| GO:0014009 | glial cell proliferation | -6.263254161 | 6 |
| GO:0046850 | regulation of bone remodeling | -6.263254161 | 6 |
| GO:0097300 | programmed necrotic cell death | -6.263254161 | 6 |
| GO:0034330 | cell junction organization | -6.262792737 | 12 |
| GO:0046434 | organophosphate catabolic process | -6.237621522 | 11 |
| GO:0009124 | nucleoside monophosphate biosynthetic process | -6.232881742 | 10 |
| GO:0009123 | nucleoside monophosphate metabolic process | -6.232669432 | 13 |
| GO:0034767 | positive regulation of ion transmembrane transport | -6.224969564 | 9 |
| GO:0042368 | vitamin D biosynthetic process | -6.224258145 | 4 |
| GO:0060736 | prostate gland growth | -6.224258145 | 4 |
| GO:0045912 | negative regulation of carbohydrate metabolic process | -6.211049379 | 6 |
| GO:0001654 | eye development | -6.207199345 | 13 |
| GO:1990776 | response to angiotensin | -6.190778505 | 5 |
| GO:0032412 | regulation of ion transmembrane transporter activity | -6.188876704 | 11 |
| GO:0009267 | cellular response to starvation | -6.179916199 | 9 |
| GO:0055088 | lipid homeostasis | -6.179916199 | 9 |
| GO:0060147 | regulation of posttranscriptional gene silencing | -6.170030574 | 8 |
| GO:0060966 | regulation of gene silencing by RNA | -6.170030574 | 8 |
| GO:0006733 | oxidoreduction coenzyme metabolic process | -6.157652128 | 10 |
| GO:0045089 | positive regulation of innate immune response | -6.156731776 | 13 |
| GO:0150063 | visual system development | -6.156731776 | 13 |
| GO:0014855 | striated muscle cell proliferation | -6.154958937 | 7 |
| GO:1902930 | regulation of alcohol biosynthetic process | -6.154958937 | 7 |
| GO:0000904 | cell morphogenesis involved in differentiation | -6.148362565 | 18 |
| GO:0002065 | columnar/cuboidal epithelial cell differentiation | -6.142527565 | 8 |
| GO:1901222 | regulation of NIK/NF-kappaB signaling | -6.115276417 | 8 |
| GO:0007263 | nitric oxide mediated signal transduction | -6.110699171 | 5 |
| GO:2000108 | positive regulation of leukocyte apoptotic process | -6.110699171 | 5 |
| GO:0048260 | positive regulation of receptor-mediated endocytosis | -6.109931112 | 6 |
| GO:0002218 | activation of innate immune response | -6.107116109 | 12 |
| GO:1904375 | regulation of protein localization to cell periphery | -6.088272891 | 8 |
| GO:0048880 | sensory system development | -6.082187413 | 13 |
| GO:0007598 | blood coagulation, extrinsic pathway | -6.0728788 | 3 |
| GO:0006721 | terpenoid metabolic process | -6.061512853 | 8 |
| GO:0006112 | energy reserve metabolic process | -6.049523172 | 7 |
| GO:0014910 | regulation of smooth muscle cell migration | -6.049523172 | 7 |
| GO:0042446 | hormone biosynthetic process | -6.049523172 | 7 |
| GO:0045445 | myoblast differentiation | -6.049523172 | 7 |
| GO:0035270 | endocrine system development | -6.034992273 | 8 |
| GO:0001516 | prostaglandin biosynthetic process | -6.03362511 | 5 |
| GO:0005979 | regulation of glycogen biosynthetic process | -6.03362511 | 5 |
| GO:0010962 | regulation of glucan biosynthetic process | -6.03362511 | 5 |
| GO:0046457 | prostanoid biosynthetic process | -6.03362511 | 5 |
| GO:0048147 | negative regulation of fibroblast proliferation | -6.03362511 | 5 |
| GO:0032874 | positive regulation of stress-activated MAPK cascade | -6.027002898 | 9 |
| GO:0051781 | positive regulation of cell division | -6.015276491 | 7 |
| GO:0060420 | regulation of heart growth | -6.015276491 | 7 |
| GO:0034605 | cellular response to heat | -6.008707219 | 8 |
| GO:0030307 | positive regulation of cell growth | -6.005739834 | 9 |
| GO:0051302 | regulation of cell division | -6.005739834 | 9 |
| GO:0019722 | calcium-mediated signaling | -5.994141087 | 10 |
| GO:0070304 | positive regulation of stress-activated protein kinase signaling cascade | -5.984616962 | 9 |
| GO:0046031 | ADP metabolic process | -5.982653856 | 8 |
| GO:0046889 | positive regulation of lipid biosynthetic process | -5.9814613 | 7 |
| GO:0002712 | regulation of B cell mediated immunity | -5.965850576 | 6 |
| GO:0002889 | regulation of immunoglobulin mediated immune response | -5.965850576 | 6 |
| GO:0006977 | DNA damage response, signal transduction by p53 class mediator resulting in cell cycle arrest | -5.965850576 | 6 |
| GO:0070228 | regulation of lymphocyte apoptotic process | -5.965850576 | 6 |
| GO:0032680 | regulation of tumor necrosis factor production | -5.963632615 | 9 |
| GO:0002675 | positive regulation of acute inflammatory response | -5.959343711 | 5 |
| GO:0010508 | positive regulation of autophagy | -5.956828442 | 8 |
| GO:0060359 | response to ammonium ion | -5.931227324 | 8 |
| GO:0090399 | replicative senescence | -5.922504434 | 4 |
| GO:0072431 | signal transduction involved in mitotic G1 DNA damage checkpoint | -5.919697764 | 6 |
| GO:1902400 | intracellular signal transduction involved in G1 DNA damage checkpoint | -5.919697764 | 6 |
| GO:0016042 | lipid catabolic process | -5.917650303 | 12 |
| GO:0034101 | erythrocyte homeostasis | -5.905846937 | 8 |
| GO:1903555 | regulation of tumor necrosis factor superfamily cytokine production | -5.901494506 | 9 |
| GO:0002250 | adaptive immune response | -5.899687371 | 17 |
| GO:0010165 | response to X-ray | -5.887664205 | 5 |
| GO:0035767 | endothelial cell chemotaxis | -5.887664205 | 5 |
| GO:0038128 | ERBB2 signaling pathway | -5.887664205 | 5 |
| GO:0051385 | response to mineralocorticoid | -5.887664205 | 5 |
| GO:0051968 | positive regulation of synaptic transmission, glutamatergic | -5.887664205 | 5 |
| GO:0060333 | interferon-gamma-mediated signaling pathway | -5.88250528 | 7 |
| GO:0030324 | lung development | -5.881048181 | 9 |
| GO:0019218 | regulation of steroid metabolic process | -5.880683799 | 8 |
| GO:1905114 | cell surface receptor signaling pathway involved in cell-cell signaling | -5.880512595 | 16 |
| GO:0060047 | heart contraction | -5.879496821 | 11 |
| GO:0007265 | Ras protein signal transduction | -5.865198032 | 12 |
| GO:0032640 | tumor necrosis factor production | -5.860732481 | 9 |
| GO:0021700 | developmental maturation | -5.849960107 | 11 |
| GO:0048469 | cell maturation | -5.840545906 | 9 |
| GO:0045454 | cell redox homeostasis | -5.830000199 | 6 |
| GO:0046456 | icosanoid biosynthetic process | -5.830000199 | 6 |
| GO:0072413 | signal transduction involved in mitotic cell cycle checkpoint | -5.830000199 | 6 |
| GO:1902402 | signal transduction involved in mitotic DNA damage checkpoint | -5.830000199 | 6 |
| GO:1902403 | signal transduction involved in mitotic DNA integrity checkpoint | -5.830000199 | 6 |
| GO:0033028 | myeloid cell apoptotic process | -5.818414765 | 5 |
| GO:1902003 | regulation of amyloid-beta formation | -5.818414765 | 5 |
| GO:0030323 | respiratory tube development | -5.800554265 | 9 |
| GO:1905330 | regulation of morphogenesis of an epithelium | -5.800554265 | 9 |
| GO:0043312 | neutrophil degranulation | -5.791408655 | 14 |
| GO:0042362 | fat-soluble vitamin biosynthetic process | -5.789842927 | 4 |
| GO:0045346 | regulation of MHC class II biosynthetic process | -5.789842927 | 4 |
| GO:0045651 | positive regulation of macrophage differentiation | -5.789842927 | 4 |
| GO:0046942 | carboxylic acid transport | -5.787855123 | 12 |
| GO:0034308 | primary alcohol metabolic process | -5.787088837 | 7 |
| GO:0048565 | digestive tract development | -5.78213693 | 8 |
| GO:0015849 | organic acid transport | -5.775117149 | 12 |
| GO:0043433 | negative regulation of DNA-binding transcription factor activity | -5.761061769 | 9 |
| GO:0071706 | tumor necrosis factor superfamily cytokine production | -5.761061769 | 9 |
| GO:0002283 | neutrophil activation involved in immune response | -5.760371273 | 14 |
| GO:0031341 | regulation of cell killing | -5.756029511 | 7 |
| GO:0051591 | response to cAMP | -5.756029511 | 7 |
| GO:0030431 | sleep | -5.751440075 | 5 |
| GO:0035633 | maintenance of permeability of blood-brain barrier | -5.751440075 | 5 |
| GO:0043276 | anoikis | -5.751440075 | 5 |
| GO:0033619 | membrane protein proteolysis | -5.743578109 | 6 |
| GO:0046324 | regulation of glucose import | -5.743578109 | 6 |
| GO:1903531 | negative regulation of secretion by cell | -5.741499214 | 9 |
| GO:0003015 | heart process | -5.734182623 | 11 |
| GO:0035249 | synaptic transmission, glutamatergic | -5.725329769 | 7 |
| GO:0009166 | nucleotide catabolic process | -5.702734726 | 9 |
| GO:0002260 | lymphocyte homeostasis | -5.701525463 | 6 |
| GO:0010518 | positive regulation of phospholipase activity | -5.701525463 | 6 |
| GO:0010676 | positive regulation of cellular carbohydrate metabolic process | -5.701525463 | 6 |
| GO:0010803 | regulation of tumor necrosis factor-mediated signaling pathway | -5.701525463 | 6 |
| GO:0009135 | purine nucleoside diphosphate metabolic process | -5.686807784 | 8 |
| GO:0009179 | purine ribonucleoside diphosphate metabolic process | -5.686807784 | 8 |
| GO:0034390 | smooth muscle cell apoptotic process | -5.686599276 | 5 |
| GO:0034391 | regulation of smooth muscle cell apoptotic process | -5.686599276 | 5 |
| GO:0071312 | cellular response to alkaloid | -5.686599276 | 5 |
| GO:0002677 | negative regulation of chronic inflammatory response | -5.676862576 | 3 |
| GO:0014805 | smooth muscle adaptation | -5.676862576 | 3 |
| GO:0038033 | positive regulation of endothelial cell chemotaxis by VEGF-activated vascular endothelial growth factor receptor signaling pathway | -5.676862576 | 3 |
| GO:0046136 | positive regulation of vitamin metabolic process | -5.676862576 | 3 |
| GO:0046666 | retinal cell programmed cell death | -5.676862576 | 3 |
| GO:0080184 | response to phenylpropanoid | -5.676862576 | 3 |
| GO:0032352 | positive regulation of hormone metabolic process | -5.666940912 | 4 |
| GO:0045342 | MHC class II biosynthetic process | -5.666940912 | 4 |
| GO:0045725 | positive regulation of glycogen biosynthetic process | -5.666940912 | 4 |
| GO:1901550 | regulation of endothelial cell development | -5.666940912 | 4 |
| GO:1903140 | regulation of establishment of endothelial barrier | -5.666940912 | 4 |
| GO:0030316 | osteoclast differentiation | -5.66497846 | 7 |
| GO:0050764 | regulation of phagocytosis | -5.66497846 | 7 |
| GO:0034113 | heterotypic cell-cell adhesion | -5.660210616 | 6 |
| GO:0046888 | negative regulation of hormone secretion | -5.660210616 | 6 |
| GO:0008584 | male gonad development | -5.640290617 | 8 |
| GO:0043401 | steroid hormone mediated signaling pathway | -5.640290617 | 8 |
| GO:0006820 | anion transport | -5.627646136 | 16 |
| GO:0070873 | regulation of glycogen metabolic process | -5.623764219 | 5 |
| GO:0009185 | ribonucleoside diphosphate metabolic process | -5.617309141 | 8 |
| GO:0046546 | development of primary male sexual characteristics | -5.617309141 | 8 |
| GO:1901292 | nucleoside phosphate catabolic process | -5.589233082 | 9 |
| GO:0070265 | necrotic cell death | -5.579698469 | 6 |
| GO:0015908 | fatty acid transport | -5.576963492 | 7 |
| GO:0044773 | mitotic DNA damage checkpoint | -5.576963492 | 7 |
| GO:0019362 | pyridine nucleotide metabolic process | -5.570710242 | 9 |
| GO:0046496 | nicotinamide nucleotide metabolic process | -5.570710242 | 9 |
| GO:0030212 | hyaluronan metabolic process | -5.562817982 | 5 |
| GO:0045730 | respiratory burst | -5.562817982 | 5 |
| GO:0090050 | positive regulation of cell migration involved in sprouting angiogenesis | -5.562817982 | 5 |
| GO:0002440 | production of molecular mediator of immune response | -5.553713505 | 11 |
| GO:0002460 | adaptive immune response based on somatic recombination of immune receptors built from immunoglobulin superfamily domains | -5.552940829 | 12 |
| GO:0030730 | sequestering of triglyceride | -5.552471719 | 4 |
| GO:0030949 | positive regulation of vascular endothelial growth factor receptor signaling pathway | -5.552471719 | 4 |
| GO:0050665 | hydrogen peroxide biosynthetic process | -5.552471719 | 4 |
| GO:0070875 | positive regulation of glycogen metabolic process | -5.552471719 | 4 |
| GO:0050864 | regulation of B cell activation | -5.533991672 | 9 |
| GO:0070498 | interleukin-1-mediated signaling pathway | -5.519882294 | 7 |
| GO:0090287 | regulation of cellular response to growth factor stimulus | -5.513343798 | 11 |
| GO:0019359 | nicotinamide nucleotide biosynthetic process | -5.505068554 | 8 |
| GO:0019363 | pyridine nucleotide biosynthetic process | -5.505068554 | 8 |
| GO:0032733 | positive regulation of interleukin-10 production | -5.503653594 | 5 |
| GO:0043029 | T cell homeostasis | -5.503653594 | 5 |
| GO:0051930 | regulation of sensory perception of pain | -5.503653594 | 5 |
| GO:0045670 | regulation of osteoclast differentiation | -5.501861308 | 6 |
| GO:1903672 | positive regulation of sprouting angiogenesis | -5.501861308 | 6 |
| GO:2000060 | positive regulation of ubiquitin-dependent protein catabolic process | -5.491801182 | 7 |
| GO:0030260 | entry into host cell | -5.483136405 | 8 |
| GO:0044409 | entry into host | -5.483136405 | 8 |
| GO:0051806 | entry into cell of other organism involved in symbiotic interaction | -5.483136405 | 8 |
| GO:0009749 | response to glucose | -5.479714606 | 9 |
| GO:0002824 | positive regulation of adaptive immune response based on somatic recombination of immune receptors built from immunoglobulin superfamily domains | -5.464018327 | 7 |
| GO:0044070 | regulation of anion transport | -5.464018327 | 7 |
| GO:0016239 | positive regulation of macroautophagy | -5.463894073 | 6 |
| GO:0050766 | positive regulation of phagocytosis | -5.463894073 | 6 |
| GO:0071479 | cellular response to ionizing radiation | -5.463894073 | 6 |
| GO:0019933 | cAMP-mediated signaling | -5.461831388 | 9 |
| GO:0051828 | entry into other organism involved in symbiotic interaction | -5.461370689 | 8 |
| GO:0034205 | amyloid-beta formation | -5.446172932 | 5 |
| GO:0046326 | positive regulation of glucose import | -5.446172932 | 5 |
| GO:0051931 | regulation of sensory perception | -5.446172932 | 5 |
| GO:0045187 | regulation of circadian sleep/wake cycle, sleep | -5.44536328 | 4 |
| GO:1900221 | regulation of amyloid-beta clearance | -5.44536328 | 4 |
| GO:0016052 | carbohydrate catabolic process | -5.444050952 | 9 |
| GO:0071897 | DNA biosynthetic process | -5.444050952 | 9 |
| GO:0072524 | pyridine-containing compound metabolic process | -5.444050952 | 9 |
| GO:0045580 | regulation of T cell differentiation | -5.439769123 | 8 |
| GO:0072525 | pyridine-containing compound biosynthetic process | -5.439769123 | 8 |
| GO:0071560 | cellular response to transforming growth factor beta stimulus | -5.438987879 | 10 |
| GO:0002708 | positive regulation of lymphocyte mediated immunity | -5.4365279 | 7 |
| GO:0030301 | cholesterol transport | -5.4365279 | 7 |
| GO:0046632 | alpha-beta T cell differentiation | -5.4365279 | 7 |
| GO:0019748 | secondary metabolic process | -5.426535347 | 6 |
| GO:0060038 | cardiac muscle cell proliferation | -5.426535347 | 6 |
| GO:0008016 | regulation of heart contraction | -5.423978269 | 10 |
| GO:0000077 | DNA damage checkpoint | -5.418329471 | 8 |
| GO:0030330 | DNA damage response, signal transduction by p53 class mediator | -5.409324241 | 7 |
| GO:0009755 | hormone-mediated signaling pathway | -5.408794251 | 9 |
| GO:0009108 | coenzyme biosynthetic process | -5.394162811 | 10 |
| GO:0002714 | positive regulation of B cell mediated immunity | -5.390285771 | 5 |
| GO:0002891 | positive regulation of immunoglobulin mediated immune response | -5.390285771 | 5 |
| GO:0042307 | positive regulation of protein import into nucleus | -5.390285771 | 5 |
| GO:1902991 | regulation of amyloid precursor protein catabolic process | -5.390285771 | 5 |
| GO:0072347 | response to anesthetic | -5.389766923 | 6 |
| GO:0090303 | positive regulation of wound healing | -5.389766923 | 6 |
| GO:0001523 | retinoid metabolic process | -5.382401852 | 7 |
| GO:0044774 | mitotic DNA integrity checkpoint | -5.382401852 | 7 |
| GO:0003057 | regulation of the force of heart contraction by chemical signal | -5.377755849 | 3 |
| GO:0070141 | response to UV-A | -5.377755849 | 3 |
| GO:0002822 | regulation of adaptive immune response based on somatic recombination of immune receptors built from immunoglobulin superfamily domains | -5.375927189 | 8 |
| GO:0060541 | respiratory system development | -5.373936313 | 9 |
| GO:0002286 | T cell activation involved in immune response | -5.355755393 | 7 |
| GO:0002706 | regulation of lymphocyte mediated immunity | -5.354960308 | 8 |
| GO:0032371 | regulation of sterol transport | -5.353571401 | 6 |
| GO:0032374 | regulation of cholesterol transport | -5.353571401 | 6 |
| GO:0042982 | amyloid precursor protein metabolic process | -5.353571401 | 6 |
| GO:0071559 | response to transforming growth factor beta | -5.349941066 | 10 |
| GO:0045780 | positive regulation of bone resorption | -5.344736621 | 4 |
| GO:0046852 | positive regulation of bone remodeling | -5.344736621 | 4 |
| GO:0051023 | regulation of immunoglobulin secretion | -5.344736621 | 4 |
| GO:0060252 | positive regulation of glial cell proliferation | -5.344736621 | 4 |
| GO:0030890 | positive regulation of B cell proliferation | -5.33590896 | 5 |
| GO:0033574 | response to testosterone | -5.33590896 | 5 |
| GO:0006090 | pyruvate metabolic process | -5.334146838 | 8 |
| GO:0045600 | positive regulation of fat cell differentiation | -5.317932137 | 6 |
| GO:0008277 | regulation of G protein-coupled receptor signaling pathway | -5.313484756 | 8 |
| GO:0051149 | positive regulation of muscle cell differentiation | -5.303269651 | 7 |
| GO:0042596 | fear response | -5.282965696 | 5 |
| GO:0048246 | macrophage chemotaxis | -5.282965696 | 5 |
| GO:1904591 | positive regulation of protein import | -5.282965696 | 5 |
| GO:0005977 | glycogen metabolic process | -5.282833202 | 6 |
| GO:0072401 | signal transduction involved in DNA integrity checkpoint | -5.282833202 | 6 |
| GO:0072422 | signal transduction involved in DNA damage checkpoint | -5.282833202 | 6 |
| GO:0009132 | nucleoside diphosphate metabolic process | -5.27260687 | 8 |
| GO:0055067 | monovalent inorganic cation homeostasis | -5.27260687 | 8 |
| GO:0060402 | calcium ion transport into cytosol | -5.27260687 | 8 |
| GO:0060968 | regulation of gene silencing | -5.27260687 | 8 |
| GO:0006839 | mitochondrial transport | -5.263252045 | 10 |
| GO:0072331 | signal transduction by p53 class mediator | -5.263252045 | 10 |
| GO:0002902 | regulation of B cell apoptotic process | -5.249861945 | 4 |
| GO:0007252 | I-kappaB phosphorylation | -5.249861945 | 4 |
| GO:0010875 | positive regulation of cholesterol efflux | -5.249861945 | 4 |
| GO:1900409 | positive regulation of cellular response to oxidative stress | -5.249861945 | 4 |
| GO:0006073 | cellular glucan metabolic process | -5.248259345 | 6 |
| GO:0010517 | regulation of phospholipase activity | -5.248259345 | 6 |
| GO:0044042 | glucan metabolic process | -5.248259345 | 6 |
| GO:0072395 | signal transduction involved in cell cycle checkpoint | -5.248259345 | 6 |
| GO:0030278 | regulation of ossification | -5.238336739 | 9 |
| GO:0030258 | lipid modification | -5.220758201 | 10 |
| GO:0031570 | DNA integrity checkpoint | -5.212377151 | 8 |
| GO:0006096 | glycolytic process | -5.20139039 | 7 |
| GO:0043620 | regulation of DNA-templated transcription in response to stress | -5.20139039 | 7 |
| GO:0008306 | associative learning | -5.180628996 | 6 |
| GO:0033077 | T cell differentiation in thymus | -5.180628996 | 6 |
| GO:0060193 | positive regulation of lipase activity | -5.180628996 | 6 |
| GO:0006757 | ATP generation from ADP | -5.176537882 | 7 |
| GO:0031214 | biomineral tissue development | -5.172927353 | 8 |
| GO:0009110 | vitamin biosynthetic process | -5.160126561 | 4 |
| GO:0017000 | antibiotic biosynthetic process | -5.160126561 | 4 |
| GO:0046827 | positive regulation of protein export from nucleus | -5.160126561 | 4 |
| GO:0010810 | regulation of cell-substrate adhesion | -5.15655234 | 9 |
| GO:0046661 | male sex differentiation | -5.153408165 | 8 |
| GO:0043500 | muscle adaptation | -5.151923561 | 7 |
| GO:0050707 | regulation of cytokine secretion | -5.151923561 | 7 |
| GO:0033555 | multicellular organismal response to stress | -5.147545046 | 6 |
| GO:0006732 | coenzyme metabolic process | -5.145186619 | 12 |
| GO:0002248 | connective tissue replacement involved in inflammatory response wound healing | -5.136640551 | 3 |
| GO:0008635 | activation of cysteine-type endopeptidase activity involved in apoptotic process by cytochrome c | -5.136640551 | 3 |
| GO:0009820 | alkaloid metabolic process | -5.136640551 | 3 |
| GO:0031622 | positive regulation of fever generation | -5.136640551 | 3 |
| GO:0035234 | ectopic germ cell programmed cell death | -5.136640551 | 3 |
| GO:0038089 | positive regulation of cell migration by vascular endothelial growth factor signaling pathway | -5.136640551 | 3 |
| GO:0060558 | regulation of calcidiol 1-monooxygenase activity | -5.136640551 | 3 |
| GO:0061044 | negative regulation of vascular wound healing | -5.136640551 | 3 |
| GO:0007088 | regulation of mitotic nuclear division | -5.134023842 | 8 |
| GO:0006775 | fat-soluble vitamin metabolic process | -5.132051696 | 5 |
| GO:0010828 | positive regulation of glucose transmembrane transport | -5.132051696 | 5 |
| GO:0070266 | necroptotic process | -5.132051696 | 5 |
| GO:0015918 | sterol transport | -5.127543261 | 7 |
| GO:0050868 | negative regulation of T cell activation | -5.127543261 | 7 |
| GO:0051817 | modification of morphology or physiology of other organism involved in symbiotic interaction | -5.127543261 | 7 |
| GO:0019058 | viral life cycle | -5.121545259 | 11 |
| GO:0042866 | pyruvate biosynthetic process | -5.103392924 | 7 |
| GO:0090150 | establishment of protein localization to membrane | -5.097741034 | 11 |
| GO:0002639 | positive regulation of immunoglobulin production | -5.084181052 | 5 |
| GO:0005978 | glycogen biosynthetic process | -5.084181052 | 5 |
| GO:0009250 | glucan biosynthetic process | -5.084181052 | 5 |
| GO:0010874 | regulation of cholesterol efflux | -5.084181052 | 5 |
| GO:0140014 | mitotic nuclear division | -5.083017542 | 10 |
| GO:0031016 | pancreas development | -5.082775037 | 6 |
| GO:0006925 | inflammatory cell apoptotic process | -5.075011026 | 4 |
| GO:0032069 | regulation of nuclease activity | -5.075011026 | 4 |
| GO:0042749 | regulation of circadian sleep/wake cycle | -5.075011026 | 4 |
| GO:0048305 | immunoglobulin secretion | -5.075011026 | 4 |
| GO:0050802 | circadian sleep/wake cycle, sleep | -5.075011026 | 4 |
| GO:1902884 | positive regulation of response to oxidative stress | -5.075011026 | 4 |
| GO:0051153 | regulation of striated muscle cell differentiation | -5.055766421 | 7 |
| GO:0002312 | B cell activation involved in immune response | -5.051064671 | 6 |
| GO:0048678 | response to axon injury | -5.051064671 | 6 |
| GO:0019935 | cyclic-nucleotide-mediated signaling | -5.045650208 | 9 |
| GO:0016579 | protein deubiquitination | -5.042821779 | 10 |
| GO:0070849 | response to epidermal growth factor | -5.037435571 | 5 |
| GO:0030218 | erythrocyte differentiation | -5.032282648 | 7 |
| GO:0016311 | dephosphorylation | -5.030330541 | 13 |
| GO:0071158 | positive regulation of cell cycle arrest | -5.019788642 | 6 |
| GO:0017157 | regulation of exocytosis | -5.014701709 | 9 |
| GO:0010288 | response to lead ion | -4.994071083 | 4 |
| GO:0045649 | regulation of macrophage differentiation | -4.994071083 | 4 |
| GO:0051043 | regulation of membrane protein ectodomain proteolysis | -4.994071083 | 4 |
| GO:1900273 | positive regulation of long-term synaptic potentiation | -4.994071083 | 4 |
| GO:0002698 | negative regulation of immune effector process | -4.985955751 | 7 |
| GO:0015718 | monocarboxylic acid transport | -4.983610572 | 8 |
| GO:0060401 | cytosolic calcium ion transport | -4.983610572 | 8 |
| GO:0034404 | nucleobase-containing small molecule biosynthetic process | -4.968871755 | 9 |
| GO:0002718 | regulation of cytokine production involved in immune response | -4.958495939 | 6 |
| GO:1903036 | positive regulation of response to wounding | -4.958495939 | 6 |
| GO:0045216 | cell-cell junction organization | -4.947245986 | 8 |
| GO:0030850 | prostate gland development | -4.947124827 | 5 |
| GO:0031279 | regulation of cyclase activity | -4.947124827 | 5 |
| GO:0045601 | regulation of endothelial cell differentiation | -4.947124827 | 5 |
| GO:0070231 | T cell apoptotic process | -4.947124827 | 5 |
| GO:0090199 | regulation of release of cytochrome c from mitochondria | -4.947124827 | 5 |
| GO:1900087 | positive regulation of G1/S transition of mitotic cell cycle | -4.947124827 | 5 |
| GO:1990090 | cellular response to nerve growth factor stimulus | -4.947124827 | 5 |
| GO:0010871 | negative regulation of receptor biosynthetic process | -4.934442801 | 3 |
| GO:0031620 | regulation of fever generation | -4.934442801 | 3 |
| GO:0032071 | regulation of endodeoxyribonuclease activity | -4.934442801 | 3 |
| GO:0033084 | regulation of immature T cell proliferation in thymus | -4.934442801 | 3 |
| GO:0036480 | neuron intrinsic apoptotic signaling pathway in response to oxidative stress | -4.934442801 | 3 |
| GO:1903376 | regulation of oxidative stress-induced neuron intrinsic apoptotic signaling pathway | -4.934442801 | 3 |
| GO:0001906 | cell killing | -4.929241534 | 8 |
| GO:0001649 | osteoblast differentiation | -4.923736388 | 9 |
| GO:0002719 | negative regulation of cytokine production involved in immune response | -4.916923781 | 4 |
| GO:0036003 | positive regulation of transcription from RNA polymerase II promoter in response to stress | -4.916923781 | 4 |
| GO:0051195 | negative regulation of cofactor metabolic process | -4.916923781 | 4 |
| GO:0090335 | regulation of brown fat cell differentiation | -4.916923781 | 4 |
| GO:1901889 | negative regulation of cell junction assembly | -4.916923781 | 4 |
| GO:0003044 | regulation of systemic arterial blood pressure mediated by a chemical signal | -4.90346969 | 5 |
| GO:0042987 | amyloid precursor protein catabolic process | -4.90346969 | 5 |
| GO:1903052 | positive regulation of proteolysis involved in cellular protein catabolic process | -4.895767639 | 7 |
| GO:0034763 | negative regulation of transmembrane transport | -4.873715074 | 7 |
| GO:0070664 | negative regulation of leukocyte proliferation | -4.86955202 | 6 |
| GO:0007264 | small GTPase mediated signal transduction | -4.86533717 | 13 |
| GO:0002204 | somatic recombination of immunoglobulin genes involved in immune response | -4.860759454 | 5 |
| GO:0002208 | somatic diversification of immunoglobulins involved in immune response | -4.860759454 | 5 |
| GO:0006984 | ER-nucleus signaling pathway | -4.860759454 | 5 |
| GO:0035272 | exocrine system development | -4.860759454 | 5 |
| GO:0038084 | vascular endothelial growth factor signaling pathway | -4.860759454 | 5 |
| GO:0045190 | isotype switching | -4.860759454 | 5 |
| GO:0050435 | amyloid-beta metabolic process | -4.860759454 | 5 |
| GO:0071354 | cellular response to interleukin-6 | -4.860759454 | 5 |
| GO:0022410 | circadian sleep/wake cycle process | -4.843236662 | 4 |
| GO:0071677 | positive regulation of mononuclear cell migration | -4.843236662 | 4 |
| GO:1904385 | cellular response to angiotensin | -4.843236662 | 4 |
| GO:2000679 | positive regulation of transcription regulatory region DNA binding | -4.843236662 | 4 |
| GO:0045619 | regulation of lymphocyte differentiation | -4.84094368 | 8 |
| GO:0034637 | cellular carbohydrate biosynthetic process | -4.840664454 | 6 |
| GO:0002449 | lymphocyte mediated immunity | -4.835467539 | 11 |
| GO:0070646 | protein modification by small protein removal | -4.824189723 | 10 |
| GO:0008361 | regulation of cell size | -4.823619901 | 8 |
| GO:0007420 | brain development | -4.820451842 | 16 |
| GO:0000186 | activation of MAPKK activity | -4.818955831 | 5 |
| GO:0101023 | vascular endothelial cell proliferation | -4.818955831 | 5 |
| GO:1905562 | regulation of vascular endothelial cell proliferation | -4.818955831 | 5 |
| GO:1990089 | response to nerve growth factor | -4.818955831 | 5 |
| GO:0050663 | cytokine secretion | -4.808694468 | 7 |
| GO:0002695 | negative regulation of leukocyte activation | -4.789298138 | 8 |
| GO:0006165 | nucleoside diphosphate phosphorylation | -4.787390166 | 7 |
| GO:0038066 | p38MAPK cascade | -4.778022815 | 5 |
| GO:0043124 | negative regulation of I-kappaB kinase/NF-kappaB signaling | -4.778022815 | 5 |
| GO:0045599 | negative regulation of fat cell differentiation | -4.778022815 | 5 |
| GO:0045661 | regulation of myoblast differentiation | -4.778022815 | 5 |
| GO:0097366 | response to bronchodilator | -4.778022815 | 5 |
| GO:0001783 | B cell apoptotic process | -4.772719239 | 4 |
| GO:0002092 | positive regulation of receptor internalization | -4.772719239 | 4 |
| GO:0006706 | steroid catabolic process | -4.772719239 | 4 |
| GO:0070633 | transepithelial transport | -4.772719239 | 4 |
| GO:0001993 | regulation of systemic arterial blood pressure by norepinephrine-epinephrine | -4.760273257 | 3 |
| GO:0033080 | immature T cell proliferation in thymus | -4.760273257 | 3 |
| GO:0035672 | oligopeptide transmembrane transport | -4.760273257 | 3 |
| GO:0042045 | epithelial fluid transport | -4.760273257 | 3 |
| GO:0051121 | hepoxilin metabolic process | -4.760273257 | 3 |
| GO:0051122 | hepoxilin biosynthetic process | -4.760273257 | 3 |
| GO:0060556 | regulation of vitamin D biosynthetic process | -4.760273257 | 3 |
| GO:0071550 | death-inducing signaling complex assembly | -4.760273257 | 3 |
| GO:0097011 | cellular response to granulocyte macrophage colony-stimulating factor stimulus | -4.760273257 | 3 |
| GO:0097012 | response to granulocyte macrophage colony-stimulating factor | -4.760273257 | 3 |
| GO:0051301 | cell division | -4.747129446 | 14 |
| GO:0046939 | nucleotide phosphorylation | -4.745318487 | 7 |
| GO:0043409 | negative regulation of MAPK cascade | -4.738612086 | 8 |
| GO:0010524 | positive regulation of calcium ion transport into cytosol | -4.737926506 | 5 |
| GO:0097306 | cellular response to alcohol | -4.728682922 | 6 |
| GO:2000177 | regulation of neural precursor cell proliferation | -4.728682922 | 6 |
| GO:0006766 | vitamin metabolic process | -4.72454569 | 7 |
| GO:0045727 | positive regulation of translation | -4.72454569 | 7 |
| GO:0001963 | synaptic transmission, dopaminergic | -4.705116205 | 4 |
| GO:0009651 | response to salt stress | -4.705116205 | 4 |
| GO:0033081 | regulation of T cell differentiation in thymus | -4.705116205 | 4 |
| GO:0046885 | regulation of hormone biosynthetic process | -4.705116205 | 4 |
| GO:0072539 | T-helper 17 cell differentiation | -4.705116205 | 4 |
| GO:1903203 | regulation of oxidative stress-induced neuron death | -4.705116205 | 4 |
| GO:1904469 | positive regulation of tumor necrosis factor secretion | -4.705116205 | 4 |
| GO:1901137 | carbohydrate derivative biosynthetic process | -4.703577191 | 16 |
| GO:0032760 | positive regulation of tumor necrosis factor production | -4.701539324 | 6 |
| GO:0106027 | neuron projection organization | -4.701539324 | 6 |
| GO:1905897 | regulation of response to endoplasmic reticulum stress | -4.701539324 | 6 |
| GO:0002931 | response to ischemia | -4.698634945 | 5 |
| GO:0070741 | response to interleukin-6 | -4.698634945 | 5 |
| GO:0051783 | regulation of nuclear division | -4.688854355 | 8 |
| GO:0032088 | negative regulation of NF-kappaB transcription factor activity | -4.674721227 | 6 |
| GO:0061097 | regulation of protein tyrosine kinase activity | -4.674721227 | 6 |
| GO:0016447 | somatic recombination of immunoglobulin gene segments | -4.660117972 | 5 |
| GO:0060688 | regulation of morphogenesis of a branching structure | -4.660117972 | 5 |
| GO:0016236 | macroautophagy | -4.654939565 | 10 |
| GO:1903557 | positive regulation of tumor necrosis factor superfamily cytokine production | -4.648221466 | 6 |
| GO:0072655 | establishment of protein localization to mitochondrion | -4.643147714 | 7 |
| GO:0002360 | T cell lineage commitment | -4.640201959 | 4 |
| GO:0033598 | mammary gland epithelial cell proliferation | -4.640201959 | 4 |
| GO:0042745 | circadian sleep/wake cycle | -4.640201959 | 4 |
| GO:0071280 | cellular response to copper ion | -4.640201959 | 4 |
| GO:0071880 | adenylate cyclase-activating adrenergic receptor signaling pathway | -4.640201959 | 4 |
| GO:0072378 | blood coagulation, fibrin clot formation | -4.640201959 | 4 |
| GO:1903579 | negative regulation of ATP metabolic process | -4.640201959 | 4 |
| GO:2000181 | negative regulation of blood vessel morphogenesis | -4.623903683 | 8 |
| GO:0007043 | cell-cell junction assembly | -4.623209144 | 7 |
| GO:0009308 | amine metabolic process | -4.623209144 | 7 |
| GO:1905517 | macrophage migration | -4.62234709 | 5 |
| GO:0032990 | cell part morphogenesis | -4.608646581 | 15 |
| GO:0010749 | regulation of nitric oxide mediated signal transduction | -4.607292492 | 3 |
| GO:0032025 | response to cobalt ion | -4.607292492 | 3 |
| GO:0033083 | regulation of immature T cell proliferation | -4.607292492 | 3 |
| GO:0051918 | negative regulation of fibrinolysis | -4.607292492 | 3 |
| GO:0071394 | cellular response to testosterone stimulus | -4.607292492 | 3 |
| GO:1900222 | negative regulation of amyloid-beta clearance | -4.607292492 | 3 |
| GO:1990535 | neuron projection maintenance | -4.607292492 | 3 |
| GO:0072073 | kidney epithelium development | -4.603430179 | 7 |
| GO:0002090 | regulation of receptor internalization | -4.585295348 | 5 |
| GO:0010656 | negative regulation of muscle cell apoptotic process | -4.585295348 | 5 |
| GO:0019369 | arachidonic acid metabolic process | -4.585295348 | 5 |
| GO:0031294 | lymphocyte costimulation | -4.585295348 | 5 |
| GO:1903749 | positive regulation of establishment of protein localization to mitochondrion | -4.585295348 | 5 |
| GO:0001782 | B cell homeostasis | -4.577776162 | 4 |
| GO:0008209 | androgen metabolic process | -4.577776162 | 4 |
| GO:0032373 | positive regulation of sterol transport | -4.577776162 | 4 |
| GO:0032376 | positive regulation of cholesterol transport | -4.577776162 | 4 |
| GO:0033032 | regulation of myeloid cell apoptotic process | -4.577776162 | 4 |
| GO:0071624 | positive regulation of granulocyte chemotaxis | -4.577776162 | 4 |
| GO:0030900 | forebrain development | -4.572212406 | 11 |
| GO:0042737 | drug catabolic process | -4.564341951 | 7 |
| GO:0070585 | protein localization to mitochondrion | -4.564341951 | 7 |
| GO:0042306 | regulation of protein import into nucleus | -4.548937231 | 5 |
| GO:0048008 | platelet-derived growth factor receptor signaling pathway | -4.548937231 | 5 |
| GO:0060043 | regulation of cardiac muscle cell proliferation | -4.548937231 | 5 |
| GO:0003007 | heart morphogenesis | -4.545990451 | 9 |
| GO:0002040 | sprouting angiogenesis | -4.544861251 | 8 |
| GO:0071322 | cellular response to carbohydrate stimulus | -4.525865274 | 7 |
| GO:0090277 | positive regulation of peptide hormone secretion | -4.520262715 | 6 |
| GO:0007176 | regulation of epidermal growth factor-activated receptor activity | -4.517660096 | 4 |
| GO:0007271 | synaptic transmission, cholinergic | -4.517660096 | 4 |
| GO:0010758 | regulation of macrophage chemotaxis | -4.517660096 | 4 |
| GO:0010800 | positive regulation of peptidyl-threonine phosphorylation | -4.517660096 | 4 |
| GO:0036475 | neuron death in response to oxidative stress | -4.517660096 | 4 |
| GO:0043457 | regulation of cellular respiration | -4.517660096 | 4 |
| GO:0071480 | cellular response to gamma radiation | -4.517660096 | 4 |
| GO:1902751 | positive regulation of cell cycle G2/M phase transition | -4.517660096 | 4 |
| GO:0043010 | camera-type eye development | -4.516925487 | 10 |
| GO:0032653 | regulation of interleukin-10 production | -4.513248556 | 5 |
| GO:1902808 | positive regulation of cell cycle G1/S phase transition | -4.513248556 | 5 |
| GO:1904064 | positive regulation of cation transmembrane transport | -4.506850922 | 7 |
| GO:0051592 | response to calcium ion | -4.487983134 | 7 |
| GO:0002381 | immunoglobulin production involved in immunoglobulin mediated immune response | -4.478206385 | 5 |
| GO:0033013 | tetrapyrrole metabolic process | -4.478206385 | 5 |
| GO:0002702 | positive regulation of production of molecular mediator of immune response | -4.471081302 | 6 |
| GO:0010522 | regulation of calcium ion transport into cytosol | -4.471081302 | 6 |
| GO:0006857 | oligopeptide transport | -4.47091047 | 3 |
| GO:0021534 | cell proliferation in hindbrain | -4.47091047 | 3 |
| GO:0031652 | positive regulation of heat generation | -4.47091047 | 3 |
| GO:0033079 | immature T cell proliferation | -4.47091047 | 3 |
| GO:0033197 | response to vitamin E | -4.47091047 | 3 |
| GO:0033483 | gas homeostasis | -4.47091047 | 3 |
| GO:0033860 | regulation of NAD(P)H oxidase activity | -4.47091047 | 3 |
| GO:0043619 | regulation of transcription from RNA polymerase II promoter in response to oxidative stress | -4.47091047 | 3 |
| GO:0045899 | positive regulation of RNA polymerase II transcriptional preinitiation complex assembly | -4.47091047 | 3 |
| GO:0051712 | positive regulation of killing of cells of other organism | -4.47091047 | 3 |
| GO:1903659 | regulation of complement-dependent cytotoxicity | -4.47091047 | 3 |
| GO:0048863 | stem cell differentiation | -4.468349559 | 9 |
| GO:0071549 | cellular response to dexamethasone stimulus | -4.459693654 | 4 |
| GO:0072538 | T-helper 17 type immune response | -4.459693654 | 4 |
| GO:1900543 | negative regulation of purine nucleotide metabolic process | -4.459693654 | 4 |
| GO:1900745 | positive regulation of p38MAPK cascade | -4.459693654 | 4 |
| GO:1902253 | regulation of intrinsic apoptotic signaling pathway by p53 class mediator | -4.459693654 | 4 |
| GO:1903364 | positive regulation of cellular protein catabolic process | -4.450679215 | 7 |
| GO:1903670 | regulation of sprouting angiogenesis | -4.450679215 | 7 |
| GO:0043502 | regulation of muscle adaptation | -4.446896342 | 6 |
| GO:0044264 | cellular polysaccharide metabolic process | -4.446896342 | 6 |
| GO:0060191 | regulation of lipase activity | -4.446896342 | 6 |
| GO:0002294 | CD4-positive, alpha-beta T cell differentiation involved in immune response | -4.44378894 | 5 |
| GO:1904589 | regulation of protein import | -4.44378894 | 5 |
| GO:1990778 | protein localization to cell periphery | -4.439221412 | 10 |
| GO:0007179 | transforming growth factor beta receptor signaling pathway | -4.437993076 | 8 |
| GO:0050871 | positive regulation of B cell activation | -4.432239172 | 7 |
| GO:1901343 | negative regulation of vasculature development | -4.423071718 | 8 |
| GO:0002042 | cell migration involved in sprouting angiogenesis | -4.42297465 | 6 |
| GO:0006885 | regulation of pH | -4.42297465 | 6 |
| GO:0090596 | sensory organ morphogenesis | -4.417741491 | 9 |
| GO:0061351 | neural precursor cell proliferation | -4.413937864 | 7 |
| GO:0002287 | alpha-beta T cell activation involved in immune response | -4.409975523 | 5 |
| GO:0002293 | alpha-beta T cell differentiation involved in immune response | -4.409975523 | 5 |
| GO:0032613 | interleukin-10 production | -4.409975523 | 5 |
| GO:0046622 | positive regulation of organ growth | -4.409975523 | 5 |
| GO:0070059 | intrinsic apoptotic signaling pathway in response to endoplasmic reticulum stress | -4.409975523 | 5 |
| GO:0008210 | estrogen metabolic process | -4.403732843 | 4 |
| GO:0034694 | response to prostaglandin | -4.403732843 | 4 |
| GO:0045980 | negative regulation of nucleotide metabolic process | -4.403732843 | 4 |
| GO:0071875 | adrenergic receptor signaling pathway | -4.403732843 | 4 |
| GO:0007200 | phospholipase C-activating G protein-coupled receptor signaling pathway | -4.399311002 | 6 |
| GO:0001678 | cellular glucose homeostasis | -4.377744055 | 7 |
| GO:0051250 | negative regulation of lymphocyte activation | -4.377744055 | 7 |
| GO:0002066 | columnar/cuboidal epithelial cell development | -4.376746451 | 5 |
| GO:0002437 | inflammatory response to antigenic stimulus | -4.376746451 | 5 |
| GO:0033344 | cholesterol efflux | -4.376746451 | 5 |
| GO:1900449 | regulation of glutamate receptor signaling pathway | -4.376746451 | 5 |
| GO:0002377 | immunoglobulin production | -4.364216449 | 8 |
| GO:0007631 | feeding behavior | -4.352737719 | 6 |
| GO:0001975 | response to amphetamine | -4.349647684 | 4 |
| GO:0042573 | retinoic acid metabolic process | -4.349647684 | 4 |
| GO:0045736 | negative regulation of cyclin-dependent protein serine/threonine kinase activity | -4.349647684 | 4 |
| GO:0090022 | regulation of neutrophil chemotaxis | -4.349647684 | 4 |
| GO:0031392 | regulation of prostaglandin biosynthetic process | -4.347891889 | 3 |
| GO:0032494 | response to peptidoglycan | -4.347891889 | 3 |
| GO:0097284 | hepatocyte apoptotic process | -4.347891889 | 3 |
| GO:0045165 | cell fate commitment | -4.331302357 | 9 |
| GO:0016445 | somatic diversification of immunoglobulins | -4.311967272 | 5 |
| GO:0008593 | regulation of Notch signaling pathway | -4.307137689 | 6 |
| GO:0007519 | skeletal muscle tissue development | -4.306941894 | 7 |
| GO:1903707 | negative regulation of hemopoiesis | -4.306941894 | 7 |
| GO:0007202 | activation of phospholipase C activity | -4.297320451 | 4 |
| GO:0032148 | activation of protein kinase B activity | -4.297320451 | 4 |
| GO:0033198 | response to ATP | -4.297320451 | 4 |
| GO:0046685 | response to arsenic-containing substance | -4.297320451 | 4 |
| GO:1904030 | negative regulation of cyclin-dependent protein kinase activity | -4.297320451 | 4 |
| GO:0035051 | cardiocyte differentiation | -4.28956169 | 7 |
| GO:0043618 | regulation of transcription from RNA polymerase II promoter in response to stress | -4.28469112 | 6 |
| GO:0032890 | regulation of organic acid transport | -4.280382297 | 5 |
| GO:0043550 | regulation of lipid kinase activity | -4.280382297 | 5 |
| GO:0072678 | T cell migration | -4.280382297 | 5 |
| GO:0015696 | ammonium transport | -4.262474296 | 6 |
| GO:0072009 | nephron epithelium development | -4.262474296 | 6 |
| GO:2000058 | regulation of ubiquitin-dependent protein catabolic process | -4.255174239 | 7 |
| GO:0002562 | somatic diversification of immune receptors via germline recombination within a single locus | -4.249311821 | 5 |
| GO:0016444 | somatic cell DNA recombination | -4.249311821 | 5 |
| GO:0042742 | defense response to bacterium | -4.248210425 | 10 |
| GO:0072659 | protein localization to plasma membrane | -4.247449808 | 9 |
| GO:0030947 | regulation of vascular endothelial growth factor receptor signaling pathway | -4.246644175 | 4 |
| GO:0032228 | regulation of synaptic transmission, GABAergic | -4.246644175 | 4 |
| GO:0045648 | positive regulation of erythrocyte differentiation | -4.246644175 | 4 |
| GO:0098801 | regulation of renal system process | -4.246644175 | 4 |
| GO:1904031 | positive regulation of cyclin-dependent protein kinase activity | -4.246644175 | 4 |
| GO:0002679 | respiratory burst involved in defense response | -4.235868171 | 3 |
| GO:0010870 | positive regulation of receptor biosynthetic process | -4.235868171 | 3 |
| GO:0016264 | gap junction assembly | -4.235868171 | 3 |
| GO:0030656 | regulation of vitamin metabolic process | -4.235868171 | 3 |
| GO:0031650 | regulation of heat generation | -4.235868171 | 3 |
| GO:0045741 | positive regulation of epidermal growth factor-activated receptor activity | -4.235868171 | 3 |
| GO:0061043 | regulation of vascular wound healing | -4.235868171 | 3 |
| GO:0097278 | complement-dependent cytotoxicity | -4.235868171 | 3 |
| GO:1902947 | regulation of tau-protein kinase activity | -4.235868171 | 3 |
| GO:0001569 | branching involved in blood vessel morphogenesis | -4.197521373 | 4 |
| GO:0002701 | negative regulation of production of molecular mediator of immune response | -4.197521373 | 4 |
| GO:0010543 | regulation of platelet activation | -4.197521373 | 4 |
| GO:0019048 | modulation by virus of host morphology or physiology | -4.197521373 | 4 |
| GO:0071634 | regulation of transforming growth factor beta production | -4.197521373 | 4 |
| GO:2000310 | regulation of NMDA receptor activity | -4.197521373 | 4 |
| GO:1903305 | regulation of regulated secretory pathway | -4.187847738 | 7 |
| GO:0050808 | synapse organization | -4.182573796 | 11 |
| GO:0032411 | positive regulation of transporter activity | -4.175820945 | 6 |
| GO:0033044 | regulation of chromosome organization | -4.167127634 | 10 |
| GO:0002768 | immune response-regulating cell surface receptor signaling pathway | -4.163939731 | 12 |
| GO:0002292 | T cell differentiation involved in immune response | -4.15903576 | 5 |
| GO:0071695 | anatomical structure maturation | -4.154884901 | 7 |
| GO:0043243 | positive regulation of protein complex disassembly | -4.149862954 | 4 |
| GO:0045191 | regulation of isotype switching | -4.149862954 | 4 |
| GO:0070423 | nucleotide-binding oligomerization domain containing signaling pathway | -4.149862954 | 4 |
| GO:0090218 | positive regulation of lipid kinase activity | -4.149862954 | 4 |
| GO:0016064 | immunoglobulin mediated immune response | -4.141226118 | 8 |
| GO:0001501 | skeletal system development | -4.13998911 | 12 |
| GO:0060538 | skeletal muscle organ development | -4.138573596 | 7 |
| GO:0014854 | response to inactivity | -4.133051933 | 3 |
| GO:0030213 | hyaluronan biosynthetic process | -4.133051933 | 3 |
| GO:0032310 | prostaglandin secretion | -4.133051933 | 3 |
| GO:0045898 | regulation of RNA polymerase II transcriptional preinitiation complex assembly | -4.133051933 | 3 |
| GO:0051709 | regulation of killing of cells of other organism | -4.133051933 | 3 |
| GO:0051917 | regulation of fibrinolysis | -4.133051933 | 3 |
| GO:2001279 | regulation of unsaturated fatty acid biosynthetic process | -4.133051933 | 3 |
| GO:0033692 | cellular polysaccharide biosynthetic process | -4.129874946 | 5 |
| GO:0007178 | transmembrane receptor protein serine/threonine kinase signaling pathway | -4.127416117 | 10 |
| GO:0038095 | Fc-epsilon receptor signaling pathway | -4.122373869 | 7 |
| GO:0072676 | lymphocyte migration | -4.113045665 | 6 |
| GO:0014912 | negative regulation of smooth muscle cell migration | -4.103587285 | 4 |
| GO:0035872 | nucleotide-binding domain, leucine rich repeat containing receptor signaling pathway | -4.103587285 | 4 |
| GO:0045923 | positive regulation of fatty acid metabolic process | -4.103587285 | 4 |
| GO:0048009 | insulin-like growth factor receptor signaling pathway | -4.103587285 | 4 |
| GO:0071604 | transforming growth factor beta production | -4.103587285 | 4 |
| GO:1905898 | positive regulation of response to endoplasmic reticulum stress | -4.103587285 | 4 |
| GO:2000144 | positive regulation of DNA-templated transcription, initiation | -4.103587285 | 4 |
| GO:0019724 | B cell mediated immunity | -4.10147689 | 8 |
| GO:0051155 | positive regulation of striated muscle cell differentiation | -4.101157625 | 5 |
| GO:1903747 | regulation of establishment of protein localization to mitochondrion | -4.101157625 | 5 |
| GO:0198738 | cell-cell signaling by wnt | -4.100468253 | 12 |
| GO:0030518 | intracellular steroid hormone receptor signaling pathway | -4.092522727 | 6 |
| GO:0051209 | release of sequestered calcium ion into cytosol | -4.092522727 | 6 |
| GO:0031647 | regulation of protein stability | -4.075890656 | 9 |
| GO:0031670 | cellular response to nutrient | -4.072871369 | 5 |
| GO:0051283 | negative regulation of sequestering of calcium ion | -4.072195069 | 6 |
| GO:0001818 | negative regulation of cytokine production | -4.068851781 | 10 |
| GO:0043903 | regulation of symbiosis, encompassing mutualism through parasitism | -4.062336475 | 8 |
| GO:0014037 | Schwann cell differentiation | -4.058619376 | 4 |
| GO:0032660 | regulation of interleukin-17 production | -4.058619376 | 4 |
| GO:0045685 | regulation of glial cell differentiation | -4.045004266 | 5 |
| GO:0072088 | nephron epithelium morphogenesis | -4.045004266 | 5 |
| GO:0003205 | cardiac chamber development | -4.043002736 | 7 |
| GO:0061564 | axon development | -4.038245516 | 12 |
| GO:0001821 | histamine secretion | -4.03806051 | 3 |
| GO:0035635 | entry of bacterium into host cell | -4.03806051 | 3 |
| GO:0051044 | positive regulation of membrane protein ectodomain proteolysis | -4.03806051 | 3 |
| GO:1900119 | positive regulation of execution phase of apoptosis | -4.03806051 | 3 |
| GO:0007613 | memory | -4.032112308 | 6 |
| GO:0051282 | regulation of sequestering of calcium ion | -4.032112308 | 6 |
| GO:0006417 | regulation of translation | -4.025889475 | 11 |
| GO:0006695 | cholesterol biosynthetic process | -4.017544888 | 5 |
| GO:1902653 | secondary alcohol biosynthetic process | -4.017544888 | 5 |
| GO:0001662 | behavioral fear response | -4.014890183 | 4 |
| GO:0032350 | regulation of hormone metabolic process | -4.014890183 | 4 |
| GO:0045740 | positive regulation of DNA replication | -4.014890183 | 4 |
| GO:0045746 | negative regulation of Notch signaling pathway | -4.014890183 | 4 |
| GO:0050691 | regulation of defense response to virus by host | -4.014890183 | 4 |
| GO:0051281 | positive regulation of release of sequestered calcium ion into cytosol | -4.014890183 | 4 |
| GO:1903427 | negative regulation of reactive oxygen species biosynthetic process | -4.014890183 | 4 |
| GO:2000279 | negative regulation of DNA biosynthetic process | -4.014890183 | 4 |
| GO:0051928 | positive regulation of calcium ion transport | -4.012350769 | 6 |
| GO:0007422 | peripheral nervous system development | -3.990482271 | 5 |
| GO:0072028 | nephron morphogenesis | -3.990482271 | 5 |
| GO:0010633 | negative regulation of epithelial cell migration | -3.973371885 | 6 |
| GO:0051208 | sequestering of calcium ion | -3.973371885 | 6 |
| GO:0002209 | behavioral defense response | -3.972335991 | 4 |
| GO:0007618 | mating | -3.972335991 | 4 |
| GO:0043267 | negative regulation of potassium ion transport | -3.972335991 | 4 |
| GO:0046825 | regulation of protein export from nucleus | -3.972335991 | 4 |
| GO:0051354 | negative regulation of oxidoreductase activity | -3.972335991 | 4 |
| GO:1900117 | regulation of execution phase of apoptosis | -3.972335991 | 4 |
| GO:0043367 | CD4-positive, alpha-beta T cell differentiation | -3.963805886 | 5 |
| GO:0003014 | renal system process | -3.954148575 | 6 |
| GO:0002070 | epithelial cell maturation | -3.949801947 | 3 |
| GO:0002523 | leukocyte migration involved in inflammatory response | -3.949801947 | 3 |
| GO:0019372 | lipoxygenase pathway | -3.949801947 | 3 |
| GO:0034116 | positive regulation of heterotypic cell-cell adhesion | -3.949801947 | 3 |
| GO:0042447 | hormone catabolic process | -3.949801947 | 3 |
| GO:0071380 | cellular response to prostaglandin E stimulus | -3.949801947 | 3 |
| GO:0071391 | cellular response to estrogen stimulus | -3.949801947 | 3 |
| GO:1900451 | positive regulation of glutamate receptor signaling pathway | -3.949801947 | 3 |
| GO:0002200 | somatic diversification of immune receptors | -3.937505616 | 5 |
| GO:0090398 | cellular senescence | -3.937505616 | 5 |
| GO:0099601 | regulation of neurotransmitter receptor activity | -3.937505616 | 5 |
| GO:0044003 | modification by symbiont of host morphology or physiology | -3.93089788 | 4 |
| GO:0046621 | negative regulation of organ growth | -3.93089788 | 4 |
| GO:1904037 | positive regulation of epithelial cell apoptotic process | -3.93089788 | 4 |
| GO:2001257 | regulation of cation channel activity | -3.921351257 | 7 |
| GO:0032945 | negative regulation of mononuclear cell proliferation | -3.91157174 | 5 |
| GO:0001953 | negative regulation of cell-matrix adhesion | -3.890521257 | 4 |
| GO:0071364 | cellular response to epidermal growth factor stimulus | -3.890521257 | 4 |
| GO:0071470 | cellular response to osmotic stress | -3.890521257 | 4 |
| GO:1902622 | regulation of neutrophil migration | -3.890521257 | 4 |
| GO:0001960 | negative regulation of cytokine-mediated signaling pathway | -3.885994904 | 5 |
| GO:0046717 | acid secretion | -3.878963276 | 6 |
| GO:0009299 | mRNA transcription | -3.867398605 | 3 |
| GO:0010224 | response to UV-B | -3.867398605 | 3 |
| GO:0010715 | regulation of extracellular matrix disassembly | -3.867398605 | 3 |
| GO:0015732 | prostaglandin transport | -3.867398605 | 3 |
| GO:0030540 | female genitalia development | -3.867398605 | 3 |
| GO:0030812 | negative regulation of nucleotide catabolic process | -3.867398605 | 3 |
| GO:0035729 | cellular response to hepatocyte growth factor stimulus | -3.867398605 | 3 |
| GO:0051198 | negative regulation of coenzyme metabolic process | -3.867398605 | 3 |
| GO:0055089 | fatty acid homeostasis | -3.867398605 | 3 |
| GO:0070242 | thymocyte apoptotic process | -3.867398605 | 3 |
| GO:0090336 | positive regulation of brown fat cell differentiation | -3.867398605 | 3 |
| GO:1903209 | positive regulation of oxidative stress-induced cell death | -3.867398605 | 3 |
| GO:1905331 | negative regulation of morphogenesis of an epithelium | -3.867398605 | 3 |
| GO:2000811 | negative regulation of anoikis | -3.867398605 | 3 |
| GO:2001267 | regulation of cysteine-type endopeptidase activity involved in apoptotic signaling pathway | -3.867398605 | 3 |
| GO:0048285 | organelle fission | -3.860888098 | 11 |
| GO:0016126 | sterol biosynthetic process | -3.860766112 | 5 |
| GO:0032620 | interleukin-17 production | -3.851155439 | 4 |
| GO:0032965 | regulation of collagen biosynthetic process | -3.851155439 | 4 |
| GO:1901031 | regulation of response to reactive oxygen species | -3.851155439 | 4 |
| GO:1903053 | regulation of extracellular matrix organization | -3.851155439 | 4 |
| GO:1905521 | regulation of macrophage migration | -3.851155439 | 4 |
| GO:0045333 | cellular respiration | -3.848436181 | 7 |
| GO:0003206 | cardiac chamber morphogenesis | -3.84235774 | 6 |
| GO:0003231 | cardiac ventricle development | -3.84235774 | 6 |
| GO:0001570 | vasculogenesis | -3.835876701 | 5 |
| GO:0042476 | odontogenesis | -3.824292777 | 6 |
| GO:0007369 | gastrulation | -3.819904955 | 7 |
| GO:0001974 | blood vessel remodeling | -3.812753291 | 4 |
| GO:0006509 | membrane protein ectodomain proteolysis | -3.812753291 | 4 |
| GO:0010677 | negative regulation of cellular carbohydrate metabolic process | -3.812753291 | 4 |
| GO:0042771 | intrinsic apoptotic signaling pathway in response to DNA damage by p53 class mediator | -3.812753291 | 4 |
| GO:0045911 | positive regulation of DNA recombination | -3.812753291 | 4 |
| GO:0060443 | mammary gland morphogenesis | -3.812753291 | 4 |
| GO:0090311 | regulation of protein deacetylation | -3.812753291 | 4 |
| GO:1904467 | regulation of tumor necrosis factor secretion | -3.812753291 | 4 |
| GO:0006470 | protein dephosphorylation | -3.812081056 | 9 |
| GO:0050886 | endocrine process | -3.811318328 | 5 |
| GO:0042177 | negative regulation of protein catabolic process | -3.806383091 | 6 |
| GO:0032740 | positive regulation of interleukin-17 production | -3.790134379 | 3 |
| GO:0043217 | myelin maintenance | -3.790134379 | 3 |
| GO:0051608 | histamine transport | -3.790134379 | 3 |
| GO:0070230 | positive regulation of lymphocyte apoptotic process | -3.790134379 | 3 |
| GO:0150078 | positive regulation of neuroinflammatory response | -3.790134379 | 3 |
| GO:2001170 | negative regulation of ATP biosynthetic process | -3.790134379 | 3 |
| GO:0014031 | mesenchymal cell development | -3.787082955 | 5 |
| GO:0110110 | positive regulation of animal organ morphogenesis | -3.787082955 | 5 |
| GO:0016525 | negative regulation of angiogenesis | -3.77776403 | 7 |
| GO:0032570 | response to progesterone | -3.775270898 | 4 |
| GO:0045581 | negative regulation of T cell differentiation | -3.775270898 | 4 |
| GO:0048538 | thymus development | -3.775270898 | 4 |
| GO:2000142 | regulation of DNA-templated transcription, initiation | -3.775270898 | 4 |
| GO:0046718 | viral entry into host cell | -3.771020102 | 6 |
| GO:0071333 | cellular response to glucose stimulus | -3.771020102 | 6 |
| GO:0007193 | adenylate cyclase-inhibiting G protein-coupled receptor signaling pathway | -3.76316283 | 5 |
| GO:0009791 | post-embryonic development | -3.76316283 | 5 |
| GO:0055076 | transition metal ion homeostasis | -3.75356221 | 6 |
| GO:0060761 | negative regulation of response to cytokine stimulus | -3.739550479 | 5 |
| GO:0090049 | regulation of cell migration involved in sprouting angiogenesis | -3.739550479 | 5 |
| GO:0003197 | endocardial cushion development | -3.738667285 | 4 |
| GO:0008542 | visual learning | -3.738667285 | 4 |
| GO:0014075 | response to amine | -3.738667285 | 4 |
| GO:0022602 | ovulation cycle process | -3.738667285 | 4 |
| GO:0035722 | interleukin-12-mediated signaling pathway | -3.738667285 | 4 |
| GO:0048806 | genitalia development | -3.738667285 | 4 |
| GO:0071331 | cellular response to hexose stimulus | -3.736250412 | 6 |
| GO:0046330 | positive regulation of JNK cascade | -3.71908254 | 6 |
| GO:0071326 | cellular response to monosaccharide stimulus | -3.71908254 | 6 |
| GO:0002922 | positive regulation of humoral immune response | -3.717417255 | 3 |
| GO:0010544 | negative regulation of platelet activation | -3.717417255 | 3 |
| GO:0010663 | positive regulation of striated muscle cell apoptotic process | -3.717417255 | 3 |
| GO:0010666 | positive regulation of cardiac muscle cell apoptotic process | -3.717417255 | 3 |
| GO:0010759 | positive regulation of macrophage chemotaxis | -3.717417255 | 3 |
| GO:0033189 | response to vitamin A | -3.717417255 | 3 |
| GO:0034393 | positive regulation of smooth muscle cell apoptotic process | -3.717417255 | 3 |
| GO:0035728 | response to hepatocyte growth factor | -3.717417255 | 3 |
| GO:0044320 | cellular response to leptin stimulus | -3.717417255 | 3 |
| GO:0032436 | positive regulation of proteasomal ubiquitin-dependent protein catabolic process | -3.716238691 | 5 |
| GO:0070098 | chemokine-mediated signaling pathway | -3.716238691 | 5 |
| GO:0010799 | regulation of peptidyl-threonine phosphorylation | -3.702904153 | 4 |
| GO:0031952 | regulation of protein autophosphorylation | -3.702904153 | 4 |
| GO:0033628 | regulation of cell adhesion mediated by integrin | -3.702904153 | 4 |
| GO:0042220 | response to cocaine | -3.702904153 | 4 |
| GO:0051932 | synaptic transmission, GABAergic | -3.702904153 | 4 |
| GO:0055023 | positive regulation of cardiac muscle tissue growth | -3.702904153 | 4 |
| GO:1900744 | regulation of p38MAPK cascade | -3.702904153 | 4 |
| GO:1990774 | tumor necrosis factor secretion | -3.702904153 | 4 |
| GO:0035304 | regulation of protein dephosphorylation | -3.702056472 | 6 |
| GO:0061448 | connective tissue development | -3.690035546 | 8 |
| GO:0048812 | neuron projection morphogenesis | -3.685937522 | 13 |
| GO:0097553 | calcium ion transmembrane import into cytosol | -3.685170136 | 6 |
| GO:0002067 | glandular epithelial cell differentiation | -3.667945656 | 4 |
| GO:0003254 | regulation of membrane depolarization | -3.667945656 | 4 |
| GO:0010712 | regulation of collagen metabolic process | -3.667945656 | 4 |
| GO:0060711 | labyrinthine layer development | -3.667945656 | 4 |
| GO:0071349 | cellular response to interleukin-12 | -3.667945656 | 4 |
| GO:0002689 | negative regulation of leukocyte chemotaxis | -3.64875215 | 3 |
| GO:0003085 | negative regulation of systemic arterial blood pressure | -3.64875215 | 3 |
| GO:0032495 | response to muramyl dipeptide | -3.64875215 | 3 |
| GO:0042036 | negative regulation of cytokine biosynthetic process | -3.64875215 | 3 |
| GO:0051412 | response to corticosterone | -3.64875215 | 3 |
| GO:0060353 | regulation of cell adhesion molecule production | -3.64875215 | 3 |
| GO:0097062 | dendritic spine maintenance | -3.64875215 | 3 |
| GO:0042475 | odontogenesis of dentin-containing tooth | -3.648038268 | 5 |
| GO:0045921 | positive regulation of exocytosis | -3.648038268 | 5 |
| GO:0045664 | regulation of neuron differentiation | -3.635922619 | 13 |
| GO:0001961 | positive regulation of cytokine-mediated signaling pathway | -3.63375819 | 4 |
| GO:0043300 | regulation of leukocyte degranulation | -3.63375819 | 4 |
| GO:0043303 | mast cell degranulation | -3.63375819 | 4 |
| GO:0070671 | response to interleukin-12 | -3.63375819 | 4 |
| GO:0071715 | icosanoid transport | -3.63375819 | 4 |
| GO:1901571 | fatty acid derivative transport | -3.63375819 | 4 |
| GO:0072376 | protein activation cascade | -3.629615066 | 7 |
| GO:0045582 | positive regulation of T cell differentiation | -3.625861448 | 5 |
| GO:0072006 | nephron development | -3.618982163 | 6 |
| GO:0045185 | maintenance of protein location | -3.603952667 | 5 |
| GO:0072080 | nephron tubule development | -3.603952667 | 5 |
| GO:0002279 | mast cell activation involved in immune response | -3.600310218 | 4 |
| GO:0045540 | regulation of cholesterol biosynthetic process | -3.600310218 | 4 |
| GO:0045646 | regulation of erythrocyte differentiation | -3.600310218 | 4 |
| GO:0051972 | regulation of telomerase activity | -3.600310218 | 4 |
| GO:0106118 | regulation of sterol biosynthetic process | -3.600310218 | 4 |
| GO:0120039 | plasma membrane bounded cell projection morphogenesis | -3.598941411 | 13 |
| GO:0000280 | nuclear division | -3.598349523 | 10 |
| GO:0002363 | alpha-beta T cell lineage commitment | -3.583720803 | 3 |
| GO:0010829 | negative regulation of glucose transmembrane transport | -3.583720803 | 3 |
| GO:0010893 | positive regulation of steroid biosynthetic process | -3.583720803 | 3 |
| GO:0030728 | ovulation | -3.583720803 | 3 |
| GO:0030809 | negative regulation of nucleotide biosynthetic process | -3.583720803 | 3 |
| GO:0035357 | peroxisome proliferator activated receptor signaling pathway | -3.583720803 | 3 |
| GO:0035813 | regulation of renal sodium excretion | -3.583720803 | 3 |
| GO:0036499 | PERK-mediated unfolded protein response | -3.583720803 | 3 |
| GO:0071498 | cellular response to fluid shear stress | -3.583720803 | 3 |
| GO:0090312 | positive regulation of protein deacetylation | -3.583720803 | 3 |
| GO:0097709 | connective tissue replacement | -3.583720803 | 3 |
| GO:1900372 | negative regulation of purine nucleotide biosynthetic process | -3.583720803 | 3 |
| GO:1903055 | positive regulation of extracellular matrix organization | -3.583720803 | 3 |
| GO:0019319 | hexose biosynthetic process | -3.582306056 | 5 |
| GO:0030641 | regulation of cellular pH | -3.582306056 | 5 |
| GO:0048858 | cell projection morphogenesis | -3.574535142 | 13 |
| GO:0002448 | mast cell mediated immunity | -3.567572095 | 4 |
| GO:0003179 | heart valve morphogenesis | -3.567572095 | 4 |
| GO:0031103 | axon regeneration | -3.567572095 | 4 |
| GO:0060421 | positive regulation of heart growth | -3.567572095 | 4 |
| GO:2000677 | regulation of transcription regulatory region DNA binding | -3.567572095 | 4 |
| GO:0060993 | kidney morphogenesis | -3.560915934 | 5 |
| GO:0061326 | renal tubule development | -3.560915934 | 5 |
| GO:0070167 | regulation of biomineral tissue development | -3.560915934 | 5 |
| GO:0035303 | regulation of dephosphorylation | -3.552745601 | 7 |
| GO:0007632 | visual behavior | -3.535515926 | 4 |
| GO:0032964 | collagen biosynthetic process | -3.535515926 | 4 |
| GO:0043331 | response to dsRNA | -3.535515926 | 4 |
| GO:0043392 | negative regulation of DNA binding | -3.535515926 | 4 |
| GO:0035809 | regulation of urine volume | -3.52196664 | 3 |
| GO:0043369 | CD4-positive or CD8-positive, alpha-beta T cell lineage commitment | -3.52196664 | 3 |
| GO:0051900 | regulation of mitochondrial depolarization | -3.52196664 | 3 |
| GO:0060352 | cell adhesion molecule production | -3.52196664 | 3 |
| GO:0071157 | negative regulation of cell cycle arrest | -3.52196664 | 3 |
| GO:0071379 | cellular response to prostaglandin stimulus | -3.52196664 | 3 |
| GO:1901739 | regulation of myoblast fusion | -3.52196664 | 3 |
| GO:2000479 | regulation of cAMP-dependent protein kinase activity | -3.52196664 | 3 |
| GO:0035710 | CD4-positive, alpha-beta T cell activation | -3.518883352 | 5 |
| GO:0046634 | regulation of alpha-beta T cell activation | -3.518883352 | 5 |
| GO:1990868 | response to chemokine | -3.518883352 | 5 |
| GO:1990869 | cellular response to chemokine | -3.518883352 | 5 |
| GO:0048667 | cell morphogenesis involved in neuron differentiation | -3.515715519 | 12 |
| GO:0046486 | glycerolipid metabolic process | -3.510054792 | 10 |
| GO:0006749 | glutathione metabolic process | -3.504115428 | 4 |
| GO:0006968 | cellular defense response | -3.504115428 | 4 |
| GO:0010043 | response to zinc ion | -3.504115428 | 4 |
| GO:0001657 | ureteric bud development | -3.498230415 | 5 |
| GO:0006476 | protein deacetylation | -3.498230415 | 5 |
| GO:0006836 | neurotransmitter transport | -3.497278769 | 8 |
| GO:0016055 | Wnt signaling pathway | -3.492353564 | 11 |
| GO:0072163 | mesonephric epithelium development | -3.477813002 | 5 |
| GO:0072164 | mesonephric tubule development | -3.477813002 | 5 |
| GO:0001837 | epithelial to mesenchymal transition | -3.477496264 | 6 |
| GO:0031056 | regulation of histone modification | -3.477496264 | 6 |
| GO:0032655 | regulation of interleukin-12 production | -3.473345809 | 4 |
| GO:0045620 | negative regulation of lymphocyte differentiation | -3.473345809 | 4 |
| GO:0045744 | negative regulation of G protein-coupled receptor signaling pathway | -3.473345809 | 4 |
| GO:0072132 | mesenchyme morphogenesis | -3.473345809 | 4 |
| GO:0031639 | plasminogen activation | -3.463183188 | 3 |
| GO:0032682 | negative regulation of chemokine production | -3.463183188 | 3 |
| GO:0035162 | embryonic hemopoiesis | -3.463183188 | 3 |
| GO:0035743 | CD4-positive, alpha-beta T cell cytokine production | -3.463183188 | 3 |
| GO:0035812 | renal sodium excretion | -3.463183188 | 3 |
| GO:0090343 | positive regulation of cell aging | -3.463183188 | 3 |
| GO:1900017 | positive regulation of cytokine production involved in inflammatory response | -3.463183188 | 3 |
| GO:1901032 | negative regulation of response to reactive oxygen species | -3.463183188 | 3 |
| GO:1902254 | negative regulation of intrinsic apoptotic signaling pathway by p53 class mediator | -3.463183188 | 3 |
| GO:1903206 | negative regulation of hydrogen peroxide-induced cell death | -3.463183188 | 3 |
| GO:2000810 | regulation of bicellular tight junction assembly | -3.463183188 | 3 |
| GO:2001039 | negative regulation of cellular response to drug | -3.463183188 | 3 |
| GO:0046364 | monosaccharide biosynthetic process | -3.457626273 | 5 |
| GO:0050792 | regulation of viral process | -3.454234107 | 7 |
| GO:0031295 | T cell costimulation | -3.443183655 | 4 |
| GO:0090183 | regulation of kidney development | -3.443183655 | 4 |
| GO:0097164 | ammonium ion metabolic process | -3.442225014 | 7 |
| GO:0007215 | glutamate receptor signaling pathway | -3.437665536 | 5 |
| GO:0042632 | cholesterol homeostasis | -3.437665536 | 5 |
| GO:0008360 | regulation of cell shape | -3.432460482 | 6 |
| GO:0030902 | hindbrain development | -3.432460482 | 6 |
| GO:0048592 | eye morphogenesis | -3.432460482 | 6 |
| GO:0048525 | negative regulation of viral process | -3.417926238 | 5 |
| GO:0055092 | sterol homeostasis | -3.417926238 | 5 |
| GO:0032615 | interleukin-12 production | -3.413606835 | 4 |
| GO:0048016 | inositol phosphate-mediated signaling | -3.413606835 | 4 |
| GO:0060760 | positive regulation of response to cytokine stimulus | -3.413606835 | 4 |
| GO:0002438 | acute inflammatory response to antigenic stimulus | -3.407105102 | 3 |
| GO:0007617 | mating behavior | -3.407105102 | 3 |
| GO:0032469 | endoplasmic reticulum calcium ion homeostasis | -3.407105102 | 3 |
| GO:0034695 | response to prostaglandin E | -3.407105102 | 3 |
| GO:0042738 | exogenous drug catabolic process | -3.407105102 | 3 |
| GO:0044062 | regulation of excretion | -3.407105102 | 3 |
| GO:0044346 | fibroblast apoptotic process | -3.407105102 | 3 |
| GO:0046628 | positive regulation of insulin receptor signaling pathway | -3.407105102 | 3 |
| GO:0051349 | positive regulation of lyase activity | -3.407105102 | 3 |
| GO:0051882 | mitochondrial depolarization | -3.407105102 | 3 |
| GO:2000209 | regulation of anoikis | -3.407105102 | 3 |
| GO:2000241 | regulation of reproductive process | -3.402991294 | 6 |
| GO:0001823 | mesonephros development | -3.398403965 | 5 |
| GO:0010665 | regulation of cardiac muscle cell apoptotic process | -3.384594402 | 4 |
| GO:1903793 | positive regulation of anion transport | -3.384594402 | 4 |
| GO:0002027 | regulation of heart rate | -3.379094433 | 5 |
| GO:0045621 | positive regulation of lymphocyte differentiation | -3.359993482 | 5 |
| GO:1902106 | negative regulation of leukocyte differentiation | -3.359993482 | 5 |
| GO:2001022 | positive regulation of response to DNA damage stimulus | -3.359993482 | 5 |
| GO:0001658 | branching involved in ureteric bud morphogenesis | -3.356126515 | 4 |
| GO:0002886 | regulation of myeloid leukocyte mediated immunity | -3.356126515 | 4 |
| GO:0031102 | neuron projection regeneration | -3.356126515 | 4 |
| GO:0031638 | zymogen activation | -3.356126515 | 4 |
| GO:0043030 | regulation of macrophage activation | -3.356126515 | 4 |
| GO:0002053 | positive regulation of mesenchymal cell proliferation | -3.353501106 | 3 |
| GO:0009404 | toxin metabolic process | -3.353501106 | 3 |
| GO:0014829 | vascular smooth muscle contraction | -3.353501106 | 3 |
| GO:0032647 | regulation of interferon-alpha production | -3.353501106 | 3 |
| GO:0043302 | positive regulation of leukocyte degranulation | -3.353501106 | 3 |
| GO:0045662 | negative regulation of myoblast differentiation | -3.353501106 | 3 |
| GO:0060330 | regulation of response to interferon-gamma | -3.353501106 | 3 |
| GO:0060334 | regulation of interferon-gamma-mediated signaling pathway | -3.353501106 | 3 |
| GO:0060571 | morphogenesis of an epithelial fold | -3.353501106 | 3 |
| GO:1905564 | positive regulation of vascular endothelial cell proliferation | -3.353501106 | 3 |
| GO:0044282 | small molecule catabolic process | -3.349265263 | 10 |
| GO:1903050 | regulation of proteolysis involved in cellular protein catabolic process | -3.348465115 | 7 |
| GO:0051656 | establishment of organelle localization | -3.348143105 | 11 |
| GO:0032414 | positive regulation of ion transmembrane transporter activity | -3.341097073 | 5 |
| GO:0010662 | regulation of striated muscle cell apoptotic process | -3.32818436 | 4 |
| GO:0042093 | T-helper cell differentiation | -3.32818436 | 4 |
| GO:0061098 | positive regulation of protein tyrosine kinase activity | -3.32818436 | 4 |
| GO:0030111 | regulation of Wnt signaling pathway | -3.328135487 | 9 |
| GO:0000018 | regulation of DNA recombination | -3.322401286 | 5 |
| GO:0043266 | regulation of potassium ion transport | -3.322401286 | 5 |
| GO:0032526 | response to retinoic acid | -3.303902308 | 5 |
| GO:0035601 | protein deacylation | -3.303902308 | 5 |
| GO:0006309 | apoptotic DNA fragmentation | -3.302168396 | 3 |
| GO:0051123 | RNA polymerase II preinitiation complex assembly | -3.302168396 | 3 |
| GO:0060740 | prostate gland epithelium morphogenesis | -3.302168396 | 3 |
| GO:0090023 | positive regulation of neutrophil chemotaxis | -3.302168396 | 3 |
| GO:1905523 | positive regulation of macrophage migration | -3.302168396 | 3 |
| GO:0003170 | heart valve development | -3.300750084 | 4 |
| GO:0010659 | cardiac muscle cell apoptotic process | -3.300750084 | 4 |
| GO:0045843 | negative regulation of striated muscle tissue development | -3.300750084 | 4 |
| GO:0090342 | regulation of cell aging | -3.300750084 | 4 |
| GO:1903078 | positive regulation of protein localization to plasma membrane | -3.300750084 | 4 |
| GO:0018958 | phenol-containing compound metabolic process | -3.285596437 | 5 |
| GO:0043954 | cellular component maintenance | -3.273806723 | 4 |
| GO:0048635 | negative regulation of muscle organ development | -3.273806723 | 4 |
| GO:0055081 | anion homeostasis | -3.273806723 | 4 |
| GO:0032535 | regulation of cellular component size | -3.270681384 | 9 |
| GO:0010256 | endomembrane system organization | -3.26885575 | 10 |
| GO:0051640 | organelle localization | -3.268771707 | 13 |
| GO:0006641 | triglyceride metabolic process | -3.267480072 | 5 |
| GO:1901800 | positive regulation of proteasomal protein catabolic process | -3.267480072 | 5 |
| GO:0002026 | regulation of the force of heart contraction | -3.252928146 | 3 |
| GO:0010971 | positive regulation of G2/M transition of mitotic cell cycle | -3.252928146 | 3 |
| GO:0032607 | interferon-alpha production | -3.252928146 | 3 |
| GO:0034114 | regulation of heterotypic cell-cell adhesion | -3.252928146 | 3 |
| GO:0050996 | positive regulation of lipid catabolic process | -3.252928146 | 3 |
| GO:1900078 | positive regulation of cellular response to insulin stimulus | -3.252928146 | 3 |
| GO:2000353 | positive regulation of endothelial cell apoptotic process | -3.252928146 | 3 |
| GO:0034440 | lipid oxidation | -3.249549713 | 5 |
| GO:0042303 | molting cycle | -3.249549713 | 5 |
| GO:0042633 | hair cycle | -3.249549713 | 5 |
| GO:0010658 | striated muscle cell apoptotic process | -3.247338154 | 4 |
| GO:0070527 | platelet aggregation | -3.247338154 | 4 |
| GO:0070588 | calcium ion transmembrane transport | -3.244008618 | 8 |
| GO:0006275 | regulation of DNA replication | -3.231801956 | 5 |
| GO:0098732 | macromolecule deacylation | -3.231801956 | 5 |
| GO:0051898 | negative regulation of protein kinase B signaling | -3.22132903 | 4 |
| GO:1901862 | negative regulation of muscle tissue development | -3.22132903 | 4 |
| GO:2000401 | regulation of lymphocyte migration | -3.22132903 | 4 |
| GO:0030004 | cellular monovalent inorganic cation homeostasis | -3.214233489 | 5 |
| GO:1903510 | mucopolysaccharide metabolic process | -3.214233489 | 5 |
| GO:0002244 | hematopoietic progenitor cell differentiation | -3.208177509 | 6 |
| GO:0003180 | aortic valve morphogenesis | -3.205621865 | 3 |
| GO:0007205 | protein kinase C-activating G protein-coupled receptor signaling pathway | -3.205621865 | 3 |
| GO:0045830 | positive regulation of isotype switching | -3.205621865 | 3 |
| GO:0060512 | prostate gland morphogenesis | -3.205621865 | 3 |
| GO:0090200 | positive regulation of release of cytochrome c from mitochondria | -3.205621865 | 3 |
| GO:0097066 | response to thyroid hormone | -3.205621865 | 3 |
| GO:1902932 | positive regulation of alcohol biosynthetic process | -3.205621865 | 3 |
| GO:2000191 | regulation of fatty acid transport | -3.205621865 | 3 |
| GO:0046916 | cellular transition metal ion homeostasis | -3.196841089 | 5 |
| GO:0098693 | regulation of synaptic vesicle cycle | -3.196841089 | 5 |
| GO:0007588 | excretion | -3.195764741 | 4 |
| GO:0010830 | regulation of myotube differentiation | -3.195764741 | 4 |
| GO:0031640 | killing of cells of other organism | -3.195764741 | 4 |
| GO:0035773 | insulin secretion involved in cellular response to glucose stimulus | -3.195764741 | 4 |
| GO:0044364 | disruption of cells of other organism | -3.195764741 | 4 |
| GO:0045576 | mast cell activation | -3.195764741 | 4 |
| GO:0051926 | negative regulation of calcium ion transport | -3.195764741 | 4 |
| GO:0060675 | ureteric bud morphogenesis | -3.195764741 | 4 |
| GO:0038093 | Fc receptor signaling pathway | -3.182983595 | 7 |
| GO:0007009 | plasma membrane organization | -3.179621619 | 5 |
| GO:0050922 | negative regulation of chemotaxis | -3.170631358 | 4 |
| GO:0072171 | mesonephric tubule morphogenesis | -3.170631358 | 4 |
| GO:0090181 | regulation of cholesterol metabolic process | -3.170631358 | 4 |
| GO:0098586 | cellular response to virus | -3.170631358 | 4 |
| GO:0006941 | striated muscle contraction | -3.168847257 | 6 |
| GO:0001773 | myeloid dendritic cell activation | -3.160108426 | 3 |
| GO:0031063 | regulation of histone deacetylation | -3.160108426 | 3 |
| GO:0042044 | fluid transport | -3.160108426 | 3 |
| GO:0045822 | negative regulation of heart contraction | -3.160108426 | 3 |
| GO:0048873 | homeostasis of number of cells within a tissue | -3.160108426 | 3 |
| GO:0050901 | leukocyte tethering or rolling | -3.160108426 | 3 |
| GO:0007409 | axonogenesis | -3.156256459 | 10 |
| GO:0021543 | pallium development | -3.155913338 | 6 |
| GO:0006879 | cellular iron ion homeostasis | -3.145915598 | 4 |
| GO:1905207 | regulation of cardiocyte differentiation | -3.145915598 | 4 |
| GO:0030282 | bone mineralization | -3.145689335 | 5 |
| GO:0043901 | negative regulation of multi-organism process | -3.143065809 | 6 |
| GO:0043043 | peptide biosynthetic process | -3.129856489 | 13 |
| GO:0002548 | monocyte chemotaxis | -3.121604784 | 4 |
| GO:0010812 | negative regulation of cell-substrate adhesion | -3.121604784 | 4 |
| GO:1904377 | positive regulation of protein localization to cell periphery | -3.121604784 | 4 |
| GO:0033137 | negative regulation of peptidyl-serine phosphorylation | -3.116261614 | 3 |
| GO:0035666 | TRIF-dependent toll-like receptor signaling pathway | -3.116261614 | 3 |
| GO:0060142 | regulation of syncytium formation by plasma membrane fusion | -3.116261614 | 3 |
| GO:0070168 | negative regulation of biomineral tissue development | -3.116261614 | 3 |
| GO:1903205 | regulation of hydrogen peroxide-induced cell death | -3.116261614 | 3 |
| GO:0098662 | inorganic cation transmembrane transport | -3.114262851 | 13 |
| GO:1901796 | regulation of signal transduction by p53 class mediator | -3.105031734 | 6 |
| GO:0002753 | cytoplasmic pattern recognition receptor signaling pathway | -3.097686803 | 4 |
| GO:0071230 | cellular response to amino acid stimulus | -3.097686803 | 4 |
| GO:0086003 | cardiac muscle cell contraction | -3.097686803 | 4 |
| GO:2000573 | positive regulation of DNA biosynthetic process | -3.097686803 | 4 |
| GO:0070613 | regulation of protein processing | -3.080089918 | 6 |
| GO:0090288 | negative regulation of cellular response to growth factor stimulus | -3.080089918 | 6 |
| GO:2001252 | positive regulation of chromosome organization | -3.080089918 | 6 |
| GO:0071242 | cellular response to ammonium ion | -3.074150082 | 4 |
| GO:0014072 | response to isoquinoline alkaloid | -3.073968097 | 3 |
| GO:0019098 | reproductive behavior | -3.073968097 | 3 |
| GO:0043278 | response to morphine | -3.073968097 | 3 |
| GO:0045117 | azole transport | -3.073968097 | 3 |
| GO:0045737 | positive regulation of cyclin-dependent protein serine/threonine kinase activity | -3.073968097 | 3 |
| GO:0045940 | positive regulation of steroid metabolic process | -3.073968097 | 3 |
| GO:0045987 | positive regulation of smooth muscle contraction | -3.073968097 | 3 |
| GO:0060261 | positive regulation of transcription initiation from RNA polymerase II promoter | -3.073968097 | 3 |
| GO:0060292 | long-term synaptic depression | -3.073968097 | 3 |
| GO:0061082 | myeloid leukocyte cytokine production | -3.073968097 | 3 |
| GO:1902230 | negative regulation of intrinsic apoptotic signaling pathway in response to DNA damage | -3.073968097 | 3 |
| GO:1902624 | positive regulation of neutrophil migration | -3.073968097 | 3 |
| GO:0006986 | response to unfolded protein | -3.067740505 | 6 |
| GO:0006611 | protein export from nucleus | -3.05547089 | 6 |
| GO:0022900 | electron transport chain | -3.05547089 | 6 |
| GO:1903317 | regulation of protein maturation | -3.05547089 | 6 |
| GO:0008088 | axo-dendritic transport | -3.050983547 | 4 |
| GO:0045739 | positive regulation of DNA repair | -3.050983547 | 4 |
| GO:0002888 | positive regulation of myeloid leukocyte mediated immunity | -3.03312573 | 3 |
| GO:0003176 | aortic valve development | -3.03312573 | 3 |
| GO:0045589 | regulation of regulatory T cell differentiation | -3.03312573 | 3 |
| GO:0046320 | regulation of fatty acid oxidation | -3.03312573 | 3 |
| GO:0043488 | regulation of mRNA stability | -3.031167555 | 6 |
| GO:1904427 | positive regulation of calcium ion transmembrane transport | -3.028176603 | 4 |
| GO:0030968 | endoplasmic reticulum unfolded protein response | -3.016303783 | 5 |
| GO:0032479 | regulation of type I interferon production | -3.016303783 | 5 |
| GO:0010611 | regulation of cardiac muscle hypertrophy | -3.005719101 | 4 |
| GO:0072078 | nephron tubule morphogenesis | -3.005719101 | 4 |
| GO:0055007 | cardiac muscle cell differentiation | -3.000801322 | 5 |
| GO:0006338 | chromatin remodeling | -2.995289513 | 6 |
| GO:0002861 | regulation of inflammatory response to antigenic stimulus | -2.993642123 | 3 |
| GO:0010464 | regulation of mesenchymal cell proliferation | -2.993642123 | 3 |
| GO:0040018 | positive regulation of multicellular organism growth | -2.993642123 | 3 |
| GO:0042755 | eating behavior | -2.993642123 | 3 |
| GO:0043552 | positive regulation of phosphatidylinositol 3-kinase activity | -2.993642123 | 3 |
| GO:1901797 | negative regulation of signal transduction by p53 class mediator | -2.993642123 | 3 |
| GO:2001038 | regulation of cellular response to drug | -2.993642123 | 3 |
| GO:1903362 | regulation of cellular protein catabolic process | -2.990513567 | 7 |
| GO:0001704 | formation of primary germ layer | -2.985439873 | 5 |
| GO:0019079 | viral genome replication | -2.985439873 | 5 |
| GO:0032606 | type I interferon production | -2.985439873 | 5 |
| GO:0032481 | positive regulation of type I interferon production | -2.983601317 | 4 |
| GO:0060070 | canonical Wnt signaling pathway | -2.974822794 | 8 |
| GO:0072089 | stem cell proliferation | -2.970217184 | 5 |
| GO:0035019 | somatic stem cell population maintenance | -2.961813924 | 4 |
| GO:0021537 | telencephalon development | -2.961704453 | 7 |
| GO:0043902 | positive regulation of multi-organism process | -2.96008413 | 6 |
| GO:0000737 | DNA catabolic process, endonucleolytic | -2.955433445 | 3 |
| GO:0002756 | MyD88-independent toll-like receptor signaling pathway | -2.955433445 | 3 |
| GO:0010762 | regulation of fibroblast migration | -2.955433445 | 3 |
| GO:0016242 | negative regulation of macroautophagy | -2.955433445 | 3 |
| GO:0036474 | cell death in response to hydrogen peroxide | -2.955433445 | 3 |
| GO:0043516 | regulation of DNA damage response, signal transduction by p53 class mediator | -2.955433445 | 3 |
| GO:0046627 | negative regulation of insulin receptor signaling pathway | -2.955433445 | 3 |
| GO:0055022 | negative regulation of cardiac muscle tissue growth | -2.955433445 | 3 |
| GO:0055094 | response to lipoprotein particle | -2.955433445 | 3 |
| GO:0061117 | negative regulation of heart growth | -2.955433445 | 3 |
| GO:0070102 | interleukin-6-mediated signaling pathway | -2.955433445 | 3 |
| GO:1902692 | regulation of neuroblast proliferation | -2.955433445 | 3 |
| GO:2001024 | negative regulation of response to drug | -2.955433445 | 3 |
| GO:0014743 | regulation of muscle hypertrophy | -2.940347976 | 4 |
| GO:0072091 | regulation of stem cell proliferation | -2.940347976 | 4 |
| GO:0043487 | regulation of RNA stability | -2.925530201 | 6 |
| GO:0030500 | regulation of bone mineralization | -2.919194884 | 4 |
| GO:0061333 | renal tubule morphogenesis | -2.919194884 | 4 |
| GO:0061418 | regulation of transcription from RNA polymerase II promoter in response to hypoxia | -2.919194884 | 4 |
| GO:0070830 | bicellular tight junction assembly | -2.919194884 | 4 |
| GO:0000083 | regulation of transcription involved in G1/S transition of mitotic cell cycle | -2.918423388 | 3 |
| GO:0007435 | salivary gland morphogenesis | -2.918423388 | 3 |
| GO:0030262 | apoptotic nuclear changes | -2.918423388 | 3 |
| GO:0032735 | positive regulation of interleukin-12 production | -2.918423388 | 3 |
| GO:0043304 | regulation of mast cell degranulation | -2.918423388 | 3 |
| GO:0045066 | regulatory T cell differentiation | -2.918423388 | 3 |
| GO:0045922 | negative regulation of fatty acid metabolic process | -2.918423388 | 3 |
| GO:0070884 | regulation of calcineurin-NFAT signaling cascade | -2.918423388 | 3 |
| GO:0071868 | cellular response to monoamine stimulus | -2.918423388 | 3 |
| GO:0071870 | cellular response to catecholamine stimulus | -2.918423388 | 3 |
| GO:0110111 | negative regulation of animal organ morphogenesis | -2.918423388 | 3 |
| GO:0019751 | polyol metabolic process | -2.910670801 | 5 |
| GO:0032434 | regulation of proteasomal ubiquitin-dependent protein catabolic process | -2.910670801 | 5 |
| GO:0044106 | cellular amine metabolic process | -2.910670801 | 5 |
| GO:0051216 | cartilage development | -2.902846144 | 6 |
| GO:0032418 | lysosome localization | -2.8983464 | 4 |
| GO:0033143 | regulation of intracellular steroid hormone receptor signaling pathway | -2.8983464 | 4 |
| GO:0050688 | regulation of defense response to virus | -2.8983464 | 4 |
| GO:0003382 | epithelial cell morphogenesis | -2.8825423 | 3 |
| GO:0033006 | regulation of mast cell activation involved in immune response | -2.8825423 | 3 |
| GO:0033280 | response to vitamin D | -2.8825423 | 3 |
| GO:0043368 | positive T cell selection | -2.8825423 | 3 |
| GO:0071402 | cellular response to lipoprotein particle stimulus | -2.8825423 | 3 |
| GO:0106056 | regulation of calcineurin-mediated signaling | -2.8825423 | 3 |
| GO:1900077 | negative regulation of cellular response to insulin stimulus | -2.8825423 | 3 |
| GO:1901890 | positive regulation of cell junction assembly | -2.8825423 | 3 |
| GO:1905332 | positive regulation of morphogenesis of an epithelium | -2.8825423 | 3 |
| GO:0045995 | regulation of embryonic development | -2.881675367 | 5 |
| GO:0007626 | locomotory behavior | -2.880436856 | 6 |
| GO:1902275 | regulation of chromatin organization | -2.880436856 | 6 |
| GO:0006446 | regulation of translational initiation | -2.877794596 | 4 |
| GO:0043407 | negative regulation of MAP kinase activity | -2.877794596 | 4 |
| GO:0050672 | negative regulation of lymphocyte proliferation | -2.877794596 | 4 |
| GO:0060395 | SMAD protein signal transduction | -2.877794596 | 4 |
| GO:0002920 | regulation of humoral immune response | -2.867365201 | 5 |
| GO:0007276 | gamete generation | -2.866413276 | 12 |
| GO:0016575 | histone deacetylation | -2.85753185 | 4 |
| GO:0120192 | tight junction assembly | -2.85753185 | 4 |
| GO:0006471 | protein ADP-ribosylation | -2.84772643 | 3 |
| GO:0006921 | cellular component disassembly involved in execution phase of apoptosis | -2.84772643 | 3 |
| GO:0060260 | regulation of transcription initiation from RNA polymerase II promoter | -2.84772643 | 3 |
| GO:0070897 | transcription preinitiation complex assembly | -2.84772643 | 3 |
| GO:0071542 | dopaminergic neuron differentiation | -2.84772643 | 3 |
| GO:1905314 | semi-lunar valve development | -2.84772643 | 3 |
| GO:0120035 | regulation of plasma membrane bounded cell projection organization | -2.841056802 | 12 |
| GO:0060048 | cardiac muscle contraction | -2.839110625 | 5 |
| GO:0014032 | neural crest cell development | -2.837550828 | 4 |
| GO:0033238 | regulation of cellular amine metabolic process | -2.837550828 | 4 |
| GO:0051279 | regulation of release of sequestered calcium ion into cytosol | -2.837550828 | 4 |
| GO:0051168 | nuclear export | -2.82557951 | 6 |
| GO:0006638 | neutral lipid metabolic process | -2.825162602 | 5 |
| GO:0006639 | acylglycerol metabolic process | -2.825162602 | 5 |
| GO:0035966 | response to topologically incorrect protein | -2.814802588 | 6 |
| GO:0098742 | cell-cell adhesion via plasma-membrane adhesion molecules | -2.814650923 | 7 |
| GO:0003203 | endocardial cushion morphogenesis | -2.813917277 | 3 |
| GO:0045777 | positive regulation of blood pressure | -2.813917277 | 3 |
| GO:0060045 | positive regulation of cardiac muscle cell proliferation | -2.813917277 | 3 |
| GO:0071392 | cellular response to estradiol stimulus | -2.813917277 | 3 |
| GO:1903523 | negative regulation of blood circulation | -2.813917277 | 3 |
| GO:0007045 | cell-substrate adherens junction assembly | -2.798405991 | 4 |
| GO:0048041 | focal adhesion assembly | -2.798405991 | 4 |
| GO:0055117 | regulation of cardiac muscle contraction | -2.798405991 | 4 |
| GO:0071277 | cellular response to calcium ion | -2.798405991 | 4 |
| GO:0120193 | tight junction organization | -2.798405991 | 4 |
| GO:2001021 | negative regulation of response to DNA damage stimulus | -2.798405991 | 4 |
| GO:0031344 | regulation of cell projection organization | -2.791098188 | 12 |
| GO:0071824 | protein-DNA complex subunit organization | -2.788248774 | 7 |
| GO:0061013 | regulation of mRNA catabolic process | -2.782849436 | 6 |
| GO:0006778 | porphyrin-containing compound metabolic process | -2.781061033 | 3 |
| GO:0007431 | salivary gland development | -2.781061033 | 3 |
| GO:0010831 | positive regulation of myotube differentiation | -2.781061033 | 3 |
| GO:0033146 | regulation of intracellular estrogen receptor signaling pathway | -2.781061033 | 3 |
| GO:0042092 | type 2 immune response | -2.781061033 | 3 |
| GO:0043114 | regulation of vascular permeability | -2.781061033 | 3 |
| GO:0060416 | response to growth hormone | -2.781061033 | 3 |
| GO:0071867 | response to monoamine | -2.781061033 | 3 |
| GO:0071869 | response to catecholamine | -2.781061033 | 3 |
| GO:1902229 | regulation of intrinsic apoptotic signaling pathway in response to DNA damage | -2.781061033 | 3 |
| GO:0016054 | organic acid catabolic process | -2.77952623 | 7 |
| GO:0046395 | carboxylic acid catabolic process | -2.77952623 | 7 |
| GO:0015844 | monoamine transport | -2.779228833 | 4 |
| GO:0050829 | defense response to Gram-negative bacterium | -2.779228833 | 4 |
| GO:0055072 | iron ion homeostasis | -2.779228833 | 4 |
| GO:0097061 | dendritic spine organization | -2.779228833 | 4 |
| GO:0032092 | positive regulation of protein binding | -2.760306689 | 4 |
| GO:0043297 | apical junction assembly | -2.760306689 | 4 |
| GO:0048864 | stem cell development | -2.760306689 | 4 |
| GO:0006412 | translation | -2.756713462 | 12 |
| GO:0001990 | regulation of systemic arterial blood pressure by hormone | -2.749108089 | 3 |
| GO:0010613 | positive regulation of cardiac muscle hypertrophy | -2.749108089 | 3 |
| GO:0016572 | histone phosphorylation | -2.749108089 | 3 |
| GO:0030501 | positive regulation of bone mineralization | -2.749108089 | 3 |
| GO:0032892 | positive regulation of organic acid transport | -2.749108089 | 3 |
| GO:0046636 | negative regulation of alpha-beta T cell activation | -2.749108089 | 3 |
| GO:1902803 | regulation of synaptic vesicle transport | -2.741633474 | 4 |
| GO:0001942 | hair follicle development | -2.723203319 | 4 |
| GO:0030512 | negative regulation of transforming growth factor beta receptor signaling pathway | -2.723203319 | 4 |
| GO:0034109 | homotypic cell-cell adhesion | -2.723203319 | 4 |
| GO:0051453 | regulation of intracellular pH | -2.723203319 | 4 |
| GO:0015672 | monovalent inorganic cation transport | -2.722612431 | 10 |
| GO:0002251 | organ or tissue specific immune response | -2.718012614 | 3 |
| GO:0007212 | dopamine receptor signaling pathway | -2.718012614 | 3 |
| GO:0009112 | nucleobase metabolic process | -2.718012614 | 3 |
| GO:0014742 | positive regulation of muscle hypertrophy | -2.718012614 | 3 |
| GO:2000008 | regulation of protein localization to cell surface | -2.718012614 | 3 |
| GO:0034620 | cellular response to unfolded protein | -2.717657378 | 5 |
| GO:0002028 | regulation of sodium ion transport | -2.705010558 | 4 |
| GO:0002831 | regulation of response to biotic stimulus | -2.704705306 | 5 |
| GO:0051588 | regulation of neurotransmitter transport | -2.704705306 | 5 |
| GO:1902749 | regulation of cell cycle G2/M phase transition | -2.690248776 | 6 |
| GO:0010955 | negative regulation of protein processing | -2.687732175 | 3 |
| GO:1903318 | negative regulation of protein maturation | -2.687732175 | 3 |
| GO:0022404 | molting cycle process | -2.687049723 | 4 |
| GO:0022405 | hair cycle process | -2.687049723 | 4 |
| GO:0043506 | regulation of JUN kinase activity | -2.687049723 | 4 |
| GO:0043537 | negative regulation of blood vessel endothelial cell migration | -2.687049723 | 4 |
| GO:1903845 | negative regulation of cellular response to transforming growth factor beta stimulus | -2.687049723 | 4 |
| GO:0060828 | regulation of canonical Wnt signaling pathway | -2.677791127 | 7 |
| GO:0014033 | neural crest cell differentiation | -2.66931553 | 4 |
| GO:0098773 | skin epidermis development | -2.66931553 | 4 |
| GO:0090263 | positive regulation of canonical Wnt signaling pathway | -2.666461665 | 5 |
| GO:0002369 | T cell cytokine production | -2.658227415 | 3 |
| GO:0006308 | DNA catabolic process | -2.658227415 | 3 |
| GO:0015701 | bicarbonate transport | -2.658227415 | 3 |
| GO:0046006 | regulation of activated T cell proliferation | -2.658227415 | 3 |
| GO:0086004 | regulation of cardiac muscle cell contraction | -2.658227415 | 3 |
| GO:2000648 | positive regulation of stem cell proliferation | -2.658227415 | 3 |
| GO:1903169 | regulation of calcium ion transmembrane transport | -2.653913368 | 5 |
| GO:0001656 | metanephros development | -2.651802874 | 4 |
| GO:0006094 | gluconeogenesis | -2.651802874 | 4 |
| GO:0031058 | positive regulation of histone modification | -2.634506821 | 4 |
| GO:0010463 | mesenchymal cell proliferation | -2.629461759 | 3 |
| GO:0033173 | calcineurin-NFAT signaling cascade | -2.629461759 | 3 |
| GO:0045687 | positive regulation of glial cell differentiation | -2.629461759 | 3 |
| GO:0045665 | negative regulation of neuron differentiation | -2.611789495 | 6 |
| GO:0051053 | negative regulation of DNA metabolic process | -2.604682866 | 5 |
| GO:0007520 | myoblast fusion | -2.601401158 | 3 |
| GO:0014047 | glutamate secretion | -2.601401158 | 3 |
| GO:0031018 | endocrine pancreas development | -2.601401158 | 3 |
| GO:0034198 | cellular response to amino acid starvation | -2.601401158 | 3 |
| GO:0055026 | negative regulation of cardiac muscle tissue development | -2.601401158 | 3 |
| GO:0097178 | ruffle assembly | -2.601401158 | 3 |
| GO:0098815 | modulation of excitatory postsynaptic potential | -2.601401158 | 3 |
| GO:0034333 | adherens junction assembly | -2.600545593 | 4 |
| GO:0045778 | positive regulation of ossification | -2.600545593 | 4 |
| GO:0002429 | immune response-activating cell surface receptor signaling pathway | -2.587064027 | 9 |
| GO:0010761 | fibroblast migration | -2.574013861 | 3 |
| GO:0032309 | icosanoid secretion | -2.574013861 | 3 |
| GO:0035987 | endodermal cell differentiation | -2.574013861 | 3 |
| GO:0043330 | response to exogenous dsRNA | -2.574013861 | 3 |
| GO:0044275 | cellular carbohydrate catabolic process | -2.574013861 | 3 |
| GO:0097028 | dendritic cell differentiation | -2.574013861 | 3 |
| GO:0050807 | regulation of synapse organization | -2.573833921 | 6 |
| GO:0030203 | glycosaminoglycan metabolic process | -2.568735792 | 5 |
| GO:0015893 | drug transport | -2.555161469 | 6 |
| GO:2001023 | regulation of response to drug | -2.551113902 | 4 |
| GO:0007157 | heterophilic cell-cell adhesion via plasma membrane cell adhesion molecules | -2.547270216 | 3 |
| GO:0032369 | negative regulation of lipid transport | -2.547270216 | 3 |
| GO:0033003 | regulation of mast cell activation | -2.547270216 | 3 |
| GO:0042551 | neuron maturation | -2.547270216 | 3 |
| GO:0045933 | positive regulation of muscle contraction | -2.547270216 | 3 |
| GO:1903115 | regulation of actin filament-based movement | -2.547270216 | 3 |
| GO:0006942 | regulation of striated muscle contraction | -2.535022483 | 4 |
| GO:0043370 | regulation of CD4-positive, alpha-beta T cell differentiation | -2.521142486 | 3 |
| GO:0050798 | activated T cell proliferation | -2.521142486 | 3 |
| GO:0060324 | face development | -2.521142486 | 3 |
| GO:0070169 | positive regulation of biomineral tissue development | -2.521142486 | 3 |
| GO:1990928 | response to amino acid starvation | -2.521142486 | 3 |
| GO:0007044 | cell-substrate junction assembly | -2.519117312 | 4 |
| GO:0010717 | regulation of epithelial to mesenchymal transition | -2.519117312 | 4 |
| GO:0045069 | regulation of viral genome replication | -2.503394577 | 4 |
| GO:0048588 | developmental cell growth | -2.500323183 | 6 |
| GO:0006111 | regulation of gluconeogenesis | -2.495604686 | 3 |
| GO:0042149 | cellular response to glucose starvation | -2.495604686 | 3 |
| GO:0045058 | T cell selection | -2.495604686 | 3 |
| GO:0048641 | regulation of skeletal muscle tissue development | -2.495604686 | 3 |
| GO:0097720 | calcineurin-mediated signaling | -2.495604686 | 3 |
| GO:0022604 | regulation of cell morphogenesis | -2.481721932 | 9 |
| GO:0035967 | cellular response to topologically incorrect protein | -2.476711305 | 5 |
| GO:0050803 | regulation of synapse structure or activity | -2.47354651 | 6 |
| GO:0050830 | defense response to Gram-positive bacterium | -2.472481749 | 4 |
| GO:0010718 | positive regulation of epithelial to mesenchymal transition | -2.470632444 | 3 |
| GO:0048546 | digestive tract morphogenesis | -2.470632444 | 3 |
| GO:1903727 | positive regulation of phospholipid metabolic process | -2.470632444 | 3 |
| GO:2000725 | regulation of cardiac muscle cell differentiation | -2.470632444 | 3 |
| GO:0048709 | oligodendrocyte differentiation | -2.457284592 | 4 |
| GO:1903320 | regulation of protein modification by small protein conjugation or removal | -2.455925974 | 6 |
| GO:0097479 | synaptic vesicle localization | -2.454530369 | 5 |
| GO:0065004 | protein-DNA complex assembly | -2.447183719 | 6 |
| GO:0032873 | negative regulation of stress-activated MAPK cascade | -2.446202864 | 3 |
| GO:0070303 | negative regulation of stress-activated protein kinase signaling cascade | -2.446202864 | 3 |
| GO:1903587 | regulation of blood vessel endothelial cell proliferation involved in sprouting angiogenesis | -2.446202864 | 3 |
| GO:0050851 | antigen receptor-mediated signaling pathway | -2.444973745 | 7 |
| GO:0003300 | cardiac muscle hypertrophy | -2.442255738 | 4 |
| GO:0032649 | regulation of interferon-gamma production | -2.442255738 | 4 |
| GO:0034766 | negative regulation of ion transmembrane transport | -2.442255738 | 4 |
| GO:0051339 | regulation of lyase activity | -2.422294413 | 3 |
| GO:0055078 | sodium ion homeostasis | -2.422294413 | 3 |
| GO:2001238 | positive regulation of extrinsic apoptotic signaling pathway | -2.422294413 | 3 |
| GO:0002456 | T cell mediated immunity | -2.412689927 | 4 |
| GO:0019395 | fatty acid oxidation | -2.412689927 | 4 |
| GO:1905269 | positive regulation of chromatin organization | -2.412689927 | 4 |
| GO:0006022 | aminoglycan metabolic process | -2.411103473 | 5 |
| GO:0043949 | regulation of cAMP-mediated signaling | -2.398886813 | 3 |
| GO:0099172 | presynapse organization | -2.398886813 | 3 |
| GO:2000772 | regulation of cellular senescence | -2.398886813 | 3 |
| GO:0010596 | negative regulation of endothelial cell migration | -2.398146694 | 4 |
| GO:0014897 | striated muscle hypertrophy | -2.398146694 | 4 |
| GO:0010975 | regulation of neuron projection development | -2.381516592 | 9 |
| GO:0010921 | regulation of phosphatase activity | -2.379322643 | 5 |
| GO:0006584 | catecholamine metabolic process | -2.375960947 | 3 |
| GO:0009712 | catechol-containing compound metabolic process | -2.375960947 | 3 |
| GO:0071320 | cellular response to cAMP | -2.375960947 | 3 |
| GO:1901185 | negative regulation of ERBB signaling pathway | -2.375960947 | 3 |
| GO:2000179 | positive regulation of neural precursor cell proliferation | -2.375960947 | 3 |
| GO:0014896 | muscle hypertrophy | -2.369524552 | 4 |
| GO:0021761 | limbic system development | -2.369524552 | 4 |
| GO:0001706 | endoderm formation | -2.353498771 | 3 |
| GO:0001954 | positive regulation of cell-matrix adhesion | -2.353498771 | 3 |
| GO:0042304 | regulation of fatty acid biosynthetic process | -2.353498771 | 3 |
| GO:0051452 | intracellular pH reduction | -2.353498771 | 3 |
| GO:0090092 | regulation of transmembrane receptor protein serine/threonine kinase signaling pathway | -2.337483337 | 6 |
| GO:0086002 | cardiac muscle cell action potential involved in contraction | -2.331483235 | 3 |
| GO:1903307 | positive regulation of regulated secretory pathway | -2.331483235 | 3 |
| GO:0006289 | nucleotide-excision repair | -2.327709543 | 4 |
| GO:0032984 | protein-containing complex disassembly | -2.325045884 | 7 |
| GO:0002223 | stimulatory C-type lectin receptor signaling pathway | -2.314058574 | 4 |
| GO:0030520 | intracellular estrogen receptor signaling pathway | -2.309898211 | 3 |
| GO:0031529 | ruffle organization | -2.309898211 | 3 |
| GO:0061178 | regulation of insulin secretion involved in cellular response to glucose stimulus | -2.309898211 | 3 |
| GO:0086065 | cell communication involved in cardiac conduction | -2.309898211 | 3 |
| GO:0030177 | positive regulation of Wnt signaling pathway | -2.297694956 | 5 |
| GO:0002043 | blood vessel endothelial cell proliferation involved in sprouting angiogenesis | -2.288728429 | 3 |
| GO:0035306 | positive regulation of dephosphorylation | -2.288728429 | 3 |
| GO:0043551 | regulation of phosphatidylinositol 3-kinase activity | -2.288728429 | 3 |
| GO:0045851 | pH reduction | -2.288728429 | 3 |
| GO:0032609 | interferon-gamma production | -2.287172322 | 4 |
| GO:0050709 | negative regulation of protein secretion | -2.287172322 | 4 |
| GO:0002220 | innate immune response activating cell surface receptor signaling pathway | -2.273932153 | 4 |
| GO:0043666 | regulation of phosphoprotein phosphatase activity | -2.260824139 | 4 |
| GO:0006661 | phosphatidylinositol biosynthetic process | -2.247845993 | 4 |
| GO:0070252 | actin-mediated cell contraction | -2.247845993 | 4 |
| GO:0032515 | negative regulation of phosphoprotein phosphatase activity | -2.247577441 | 3 |
| GO:0098930 | axonal transport | -2.247577441 | 3 |
| GO:0014902 | myotube differentiation | -2.234995486 | 4 |
| GO:0031343 | positive regulation of cell killing | -2.227569467 | 3 |
| GO:0044272 | sulfur compound biosynthetic process | -2.210899765 | 5 |
| GO:0002792 | negative regulation of peptide secretion | -2.209668764 | 4 |
| GO:0021987 | cerebral cortex development | -2.209668764 | 4 |
| GO:0031398 | positive regulation of protein ubiquitination | -2.209668764 | 4 |
| GO:0007405 | neuroblast proliferation | -2.207923105 | 3 |
| GO:0010823 | negative regulation of mitochondrion organization | -2.207923105 | 3 |
| GO:0045824 | negative regulation of innate immune response | -2.207923105 | 3 |
| GO:0044839 | cell cycle G2/M phase transition | -2.204222675 | 6 |
| GO:0061136 | regulation of proteasomal protein catabolic process | -2.201563943 | 5 |
| GO:0043244 | regulation of protein complex disassembly | -2.197188368 | 4 |
| GO:0006644 | phospholipid metabolic process | -2.189670676 | 8 |
| GO:0000768 | syncytium formation by plasma membrane fusion | -2.188626568 | 3 |
| GO:0006521 | regulation of cellular amino acid metabolic process | -2.188626568 | 3 |
| GO:0032507 | maintenance of protein location in cell | -2.188626568 | 3 |
| GO:0051893 | regulation of focal adhesion assembly | -2.188626568 | 3 |
| GO:0090109 | regulation of cell-substrate junction assembly | -2.188626568 | 3 |
| GO:0140253 | cell-cell fusion | -2.188626568 | 3 |
| GO:2000514 | regulation of CD4-positive, alpha-beta T cell activation | -2.188626568 | 3 |
| GO:0042130 | negative regulation of T cell proliferation | -2.169668637 | 3 |
| GO:0046173 | polyol biosynthetic process | -2.169668637 | 3 |
| GO:0046637 | regulation of alpha-beta T cell differentiation | -2.169668637 | 3 |
| GO:0006949 | syncytium formation | -2.151038623 | 3 |
| GO:0032729 | positive regulation of interferon-gamma production | -2.151038623 | 3 |
| GO:0035308 | negative regulation of protein dephosphorylation | -2.151038623 | 3 |
| GO:0048247 | lymphocyte chemotaxis | -2.151038623 | 3 |
| GO:0007585 | respiratory gaseous exchange | -2.132726335 | 3 |
| GO:0034394 | protein localization to cell surface | -2.132726335 | 3 |
| GO:0051705 | multi-organism behavior | -2.132726335 | 3 |
| GO:0099504 | synaptic vesicle cycle | -2.128959055 | 5 |
| GO:0017015 | regulation of transforming growth factor beta receptor signaling pathway | -2.124743739 | 4 |
| GO:0071300 | cellular response to retinoic acid | -2.114722049 | 3 |
| GO:0016570 | histone modification | -2.108826468 | 8 |
| GO:1903844 | regulation of cellular response to transforming growth factor beta stimulus | -2.101480111 | 4 |
| GO:0045666 | positive regulation of neuron differentiation | -2.083410331 | 7 |
| GO:0006282 | regulation of DNA repair | -2.078635848 | 4 |
| GO:0008544 | epidermis development | -2.077494179 | 8 |
| GO:0035637 | multicellular organismal signaling | -2.076813103 | 5 |
| GO:0006575 | cellular modified amino acid metabolic process | -2.068304937 | 5 |
| GO:1902905 | positive regulation of supramolecular fiber organization | -2.068304937 | 5 |
| GO:0090101 | negative regulation of transmembrane receptor protein serine/threonine kinase signaling pathway | -2.067366952 | 4 |
| GO:0051187 | cofactor catabolic process | -2.062466386 | 3 |
| GO:1903391 | regulation of adherens junction organization | -2.062466386 | 3 |
| GO:0043087 | regulation of GTPase activity | -2.056913799 | 8 |
| GO:0015800 | acidic amino acid transport | -2.045605263 | 3 |
| GO:0050891 | multicellular organismal water homeostasis | -2.045605263 | 3 |
| GO:2001259 | positive regulation of cation channel activity | -2.045605263 | 3 |
| GO:0050853 | B cell receptor signaling pathway | -2.045127893 | 4 |
| GO:0031396 | regulation of protein ubiquitination | -2.043083717 | 5 |
| GO:0016569 | covalent chromatin modification | -2.04163715 | 8 |
| GO:0019226 | transmission of nerve impulse | -2.02900961 | 3 |
| GO:0043647 | inositol phosphate metabolic process | -2.02900961 | 3 |
| GO:0045806 | negative regulation of endocytosis | -2.02900961 | 3 |
| GO:0070988 | demethylation | -2.02900961 | 3 |
| GO:0030534 | adult behavior | -2.023277236 | 4 |
| GO:0042552 | myelination | -2.023277236 | 4 |
| GO:0006310 | DNA recombination | -2.01314753 | 6 |
| GO:0019730 | antimicrobial humoral response | -2.012493965 | 4 |
| GO:0099003 | vesicle-mediated transport in synapse | -2.002030495 | 5 |
| GO:0007272 | ensheathment of neurons | -2.001803546 | 4 |
| GO:0008366 | axon ensheathment | -2.001803546 | 4 |
