# Supplementary material for: Network Pharmacology Approach to Explore the Potential Mechanisms of Jieduan-Niwan Formula Treating Acute-on-Chronic Liver Failure
Source: Evid Based Complement Alternat Med. 2020 Dec 30;2020:1041307. doi: 10.1155/2020/1041307 (PMC7787753; doi:10.1155/2020/1041307)
Supplement: Supplementary Materials — Supplementary Material 1: Table S1: information of potentially bioactive compounds of Jieduan-Niwan Formula. Supplementary Material 2: Table S2: the details of targets from compounds in JDNW Formula. Supplementary Material 3: Table S3: 1471 known ACLF-related targets. Supplementary Material 4: Table S4: 168 potential targets shared in JDNW Formula and ACLF. Supplementary Material 5: Table S5: information of potentially bioactive compounds of 168 common targets. Supplementary Material 6: Table S6: GO cellular component enrichment analysis of key targets of JDNW Formula in the treatment of ACLF. Supplementary Material 7: Table S7: KEGG pathway enrichment analysis of key targets of JDNW Formula in the treatment of ACLF. [file 1041307.f1.zip › 1041307.f1/Table S7.1041307.v2.docx]

| **KEGG pathway enrichment analysis of key targets of JDNW Formula in the treatment of ACLF** | | | | | |
| --- | --- | --- | --- | --- | --- |
| **Term** | **Description** | **LogP** | **P-Value** | **Gene Number** | **RichFactor** |
| hsa05200 | Pathways in cancer | -75.5983369 | 2.5E-76 | 72 | 0.1252 |
| hsa05161 | Hepatitis B | -49.5943153 | 2.5E-50 | 39 | 0.2203 |
| ko04933 | AGE-RAGE signaling pathway in diabetic complications | -46.6091172 | 2.5E-47 | 32 | 0.3232 |
| ko05418 | Fluid shear stress and atherosclerosis | -46.296042 | 5.1E-47 | 35 | 0.2465 |
| hsa05418 | Fluid shear stress and atherosclerosis | -45.5910266 | 2.6E-46 | 35 | 0.2365 |
| hsa04933 | AGE-RAGE signaling pathway in diabetic complications | -45.3395692 | 4.6E-46 | 32 | 0.2991 |
| hsa05167 | kaposi sarcoma-associated herpesvirus infection | -44.4535932 | 3.5E-45 | 37 | 0.1907 |
| hsa05160 | Hepatitis C | -43.3617072 | 4.3E-44 | 35 | 0.2071 |
| hsa05163 | human cytomegalovirus infection | -42.4000646 | 4E-43 | 38 | 0.1583 |
| hsa05215 | Prostate cancer | -42.2202981 | 6E-43 | 30 | 0.2941 |
| hsa04151 | PI3K-Akt signaling pathway | -41.6499627 | 2.2E-42 | 43 | 0.1132 |
| ko04151 | PI3K-Akt signaling pathway | -37.7755975 | 1.7E-38 | 39 | 0.114 |
| hsa05205 | Proteoglycans in cancer | -37.6048633 | 2.5E-38 | 34 | 0.156 |
| hsa04668 | TNF signaling pathway | -36.7637331 | 1.7E-37 | 28 | 0.2435 |
| ko04657 | IL-17 signaling pathway | -35.8938803 | 1.3E-36 | 26 | 0.2796 |
| hsa05162 | Measles | -35.8512188 | 1.4E-36 | 30 | 0.1899 |
| hsa04657 | IL-17 signaling pathway | -35.6186519 | 2.4E-36 | 26 | 0.2737 |
| ko05205 | Proteoglycans in cancer | -35.5123141 | 3.1E-36 | 32 | 0.1576 |
| ko05215 | Prostate cancer | -34.853007 | 1.4E-35 | 25 | 0.2874 |
| hsa04010 | MAPK signaling pathway | -34.5747418 | 2.7E-35 | 36 | 0.1125 |
| ko04668 | TNF signaling pathway | -33.9821013 | 1E-34 | 26 | 0.2407 |
| hsa05212 | Pancreatic cancer | -33.6788944 | 2.1E-34 | 24 | 0.2927 |
| ko05219 | Bladder cancer | -33.5596404 | 2.8E-34 | 20 | 0.4878 |
| hsa05169 | Epstein-Barr virus infection | -32.7722631 | 1.7E-33 | 34 | 0.1137 |
| hsa05219 | Bladder cancer | -32.7504164 | 1.8E-33 | 20 | 0.4545 |
| ko05212 | Pancreatic cancer | -32.6858036 | 2.1E-33 | 22 | 0.3438 |
| ko05145 | Toxoplasmosis | -31.6544583 | 2.2E-32 | 25 | 0.2212 |
| hsa05166 | Human T-cell leukemia virus 1 infection | -31.5510479 | 2.8E-32 | 33 | 0.1119 |
| hsa05145 | Toxoplasmosis | -31.341508 | 4.6E-32 | 25 | 0.2155 |
| hsa05225 | hepatocellular carcinoma | -30.7167281 | 1.9E-31 | 28 | 0.1538 |
| hsa05164 | Influenza A | -30.7167281 | 1.9E-31 | 28 | 0.1538 |
| hsa05142 | Chagas disease | -30.5460737 | 2.8E-31 | 24 | 0.2243 |
| hsa01522 | Endocrine resistance | -30.0062875 | 9.9E-31 | 23 | 0.2396 |
| ko01522 | Endocrine resistance | -30.0062875 | 9.9E-31 | 23 | 0.2396 |
| hsa04210 | Apoptosis | -29.8578679 | 1.4E-30 | 26 | 0.1722 |
| ko05164 | Influenza A | -29.7905017 | 1.6E-30 | 27 | 0.1561 |
| hsa05222 | Small cell lung cancer | -29.6642622 | 2.2E-30 | 23 | 0.2323 |
| hsa04068 | foxo signaling pathway | -29.381569 | 4.2E-30 | 25 | 0.1825 |
| ko05142 | Chagas disease (American trypanosomiasis) | -29.3340546 | 4.6E-30 | 23 | 0.2255 |
| hsa04066 | HIF-1 signaling pathway | -29.3324124 | 4.7E-30 | 24 | 0.2017 |
| ko04210 | Apoptosis | -29.2969151 | 5E-30 | 25 | 0.1812 |
| ko05166 | HTLV-I infection | -29.2586755 | 5.5E-30 | 30 | 0.1172 |
| hsa01521 | EGFR tyrosine kinase inhibitor resistance | -28.4746481 | 3.4E-29 | 21 | 0.2658 |
| ko01521 | EGFR tyrosine kinase inhibitor resistance | -28.4746481 | 3.4E-29 | 21 | 0.2658 |
| hsa05223 | Non-small cell lung cancer | -27.8440659 | 1.4E-28 | 20 | 0.2857 |
| ko05206 | MicroRNAs in cancer | -27.2114329 | 6.1E-28 | 30 | 0.1003 |
| hsa05206 | MicroRNAs in cancer | -26.866566 | 1.4E-27 | 30 | 0.0977 |
| hsa05165 | human papillomavirus infection | -26.3791121 | 4.2E-27 | 31 | 0.0881 |
| ko05222 | Small cell lung cancer | -26.0531814 | 8.8E-27 | 20 | 0.2381 |
| ko05223 | Non-small cell lung cancer | -25.8345563 | 1.5E-26 | 18 | 0.3103 |
| ko05140 | Leishmaniasis | -25.5870623 | 2.6E-26 | 19 | 0.2603 |
| hsa01524 | Platinum drug resistance | -25.5870623 | 2.6E-26 | 19 | 0.2603 |
| ko01524 | Platinum drug resistance | -25.5870623 | 2.6E-26 | 19 | 0.2603 |
| hsa04932 | Non-alcoholic fatty liver disease | -25.4478051 | 3.6E-26 | 24 | 0.142 |
| hsa04625 | c-type lectin receptor signaling pathway | -25.4119961 | 3.9E-26 | 21 | 0.1963 |
| hsa05210 | Colorectal cancer | -24.975295 | 1.1E-25 | 20 | 0.2128 |
| hsa05220 | Chronic myeloid leukemia | -24.5141252 | 3.1E-25 | 19 | 0.2317 |
| hsa05218 | Melanoma | -24.4034331 | 3.9E-25 | 19 | 0.2289 |
| hsa05140 | Leishmaniasis | -24.1866043 | 6.5E-25 | 19 | 0.2235 |
| ko05220 | Chronic myeloid leukemia | -24.024121 | 9.5E-25 | 18 | 0.2535 |
| ko05210 | Colorectal cancer | -23.6389307 | 2.3E-24 | 17 | 0.2833 |
| ko05160 | Hepatitis C | -23.4418591 | 3.6E-24 | 21 | 0.1603 |
| ko05152 | Tuberculosis | -23.3580685 | 4.4E-24 | 23 | 0.1285 |
| hsa05214 | Glioma | -23.2045228 | 6.2E-24 | 18 | 0.2308 |
| ko05214 | Glioma | -23.0960944 | 8E-24 | 17 | 0.2656 |
| hsa05152 | Tuberculosis | -22.6407278 | 2.3E-23 | 23 | 0.1198 |
| ko05218 | Melanoma | -22.4712921 | 3.4E-23 | 17 | 0.2464 |
| ko04660 | T cell receptor signaling pathway | -22.4648719 | 3.4E-23 | 19 | 0.1845 |
| hsa04218 | cellular senescence | -22.2475511 | 5.7E-23 | 22 | 0.1272 |
| ko04932 | Non-alcoholic fatty liver disease (NAFLD) | -22.2136855 | 6.1E-23 | 21 | 0.1409 |
| ko04659 | Th17 cell differentiation | -22.1287071 | 7.4E-23 | 19 | 0.1776 |
| ko04380 | Osteoclast differentiation | -21.9614841 | 1.1E-22 | 20 | 0.1538 |
| hsa04659 | Th17 cell differentiation | -21.727904 | 1.9E-22 | 19 | 0.1696 |
| ko05133 | Pertussis | -21.6801003 | 2.1E-22 | 17 | 0.2237 |
| hsa04660 | T cell receptor signaling pathway | -21.5731869 | 2.7E-22 | 19 | 0.1667 |
| hsa04926 | relaxin signaling pathway | -21.5516859 | 2.8E-22 | 20 | 0.1471 |
| hsa04510 | Focal adhesion | -21.494386 | 3.2E-22 | 23 | 0.107 |
| ko05144 | Malaria | -21.4827745 | 3.3E-22 | 15 | 0.3061 |
| hsa05224 | Breast cancer | -21.425341 | 3.8E-22 | 21 | 0.1296 |
| hsa04115 | p53 signaling pathway | -21.3664262 | 4.3E-22 | 17 | 0.2152 |
| hsa04380 | Osteoclast differentiation | -21.3542849 | 4.4E-22 | 20 | 0.1439 |
| hsa05133 | Pertussis | -21.1648155 | 6.8E-22 | 17 | 0.2099 |
| ko04010 | MAPK signaling pathway | -21.0866596 | 8.2E-22 | 24 | 0.0941 |
| ko05224 | Breast cancer | -21.0356028 | 9.2E-22 | 20 | 0.1389 |
| hsa04014 | Ras signaling pathway | -20.9253543 | 1.2E-21 | 24 | 0.0927 |
| ko04510 | Focal adhesion | -20.884343 | 1.3E-21 | 22 | 0.1106 |
| hsa05170 | human immunodeficiency virus 1 infection | -20.8611567 | 1.4E-21 | 23 | 0.1004 |
| ko04620 | Toll-like receptor signaling pathway | -20.7674483 | 1.7E-21 | 18 | 0.1731 |
| hsa05144 | Malaria | -20.6180787 | 2.4E-21 | 15 | 0.2727 |
| hsa04620 | Toll-like receptor signaling pathway | -20.2284584 | 5.9E-21 | 18 | 0.1622 |
| hsa04931 | insulin resistance | -20.0814447 | 8.3E-21 | 18 | 0.1593 |
| hsa05226 | gastric cancer | -19.9824787 | 1E-20 | 20 | 0.1235 |
| ko04064 | NF-kappa B signaling pathway | -19.8956338 | 1.3E-20 | 17 | 0.1789 |
| ko04621 | NOD-like receptor signaling pathway | -19.5553772 | 2.8E-20 | 20 | 0.1176 |
| ko05169 | Epstein-Barr virus infection | -19.4251913 | 3.8E-20 | 21 | 0.1045 |
| hsa05202 | Transcriptional misregulation in cancer | -19.2894946 | 5.1E-20 | 21 | 0.1029 |
| ko05202 | Transcriptional misregulation in cancer | -19.0517642 | 8.9E-20 | 20 | 0.1111 |
| hsa04064 | NF-kappa B signaling pathway | -18.9656564 | 1.1E-19 | 17 | 0.1589 |
| hsa04621 | NOD-like receptor signaling pathway | -18.7642355 | 1.7E-19 | 20 | 0.1075 |
| ko05323 | Rheumatoid arthritis | -18.699524 | 2E-19 | 16 | 0.1778 |
| ko05162 | Measles | -18.6935246 | 2E-19 | 18 | 0.1343 |
| hsa04630 | JAK-STAT signaling pathway | -18.1143633 | 7.7E-19 | 19 | 0.1111 |
| hsa05323 | Rheumatoid arthritis | -17.9980719 | 1E-18 | 16 | 0.1616 |
| ko04060 | Cytokine-cytokine receptor interaction | -17.9827416 | 1E-18 | 22 | 0.0815 |
| ko05321 | Inflammatory bowel disease (IBD) | -17.6738023 | 2.1E-18 | 14 | 0.2154 |
| hsa05213 | Endometrial cancer | -17.4729385 | 3.4E-18 | 14 | 0.209 |
| hsa05321 | Inflammatory bowel disease | -17.4729385 | 3.4E-18 | 14 | 0.209 |
| hsa04060 | Cytokine-cytokine receptor interaction | -17.3343659 | 4.6E-18 | 23 | 0.0701 |
| ko04115 | p53 signaling pathway | -17.2788128 | 5.3E-18 | 14 | 0.2029 |
| ko04917 | Prolactin signaling pathway | -17.1841407 | 6.5E-18 | 14 | 0.2 |
| hsa04024 | cAMP signaling pathway | -17.0765282 | 8.4E-18 | 20 | 0.0885 |
| hsa04917 | Prolactin signaling pathway | -16.7327601 | 1.9E-17 | 14 | 0.1867 |
| ko04071 | Sphingolipid signaling pathway | -16.7265914 | 1.9E-17 | 16 | 0.1356 |
| ko05146 | Amoebiasis | -16.6685478 | 2.1E-17 | 15 | 0.1563 |
| hsa05143 | African trypanosomiasis | -16.5823063 | 2.6E-17 | 12 | 0.2727 |
| hsa04919 | thyroid hormone signaling pathway | -16.429712 | 3.7E-17 | 16 | 0.1301 |
| hsa04071 | Sphingolipid signaling pathway | -16.1459304 | 7.1E-17 | 16 | 0.125 |
| ko05168 | Herpes simplex infection | -16.1374972 | 7.3E-17 | 18 | 0.0973 |
| ko04630 | Jak-STAT signaling pathway | -16.1009654 | 7.9E-17 | 17 | 0.109 |
| ko05143 | African trypanosomiasis | -16.0170677 | 9.6E-17 | 11 | 0.3143 |
| hsa05146 | Amoebiasis | -15.9323774 | 1.2E-16 | 15 | 0.1402 |
| ko05132 | Salmonella infection | -15.8491818 | 1.4E-16 | 14 | 0.1628 |
| ko04012 | ErbB signaling pathway | -15.8491818 | 1.4E-16 | 14 | 0.1628 |
| ko05213 | Endometrial cancer | -15.8356248 | 1.5E-16 | 12 | 0.24 |
| hsa04015 | Rap1 signaling pathway | -15.7506155 | 1.8E-16 | 19 | 0.0833 |
| ko05014 | Amyotrophic lateral sclerosis (ALS) | -15.7213601 | 1.9E-16 | 12 | 0.2353 |
| ko04658 | Th1 and Th2 cell differentiation | -15.4191631 | 3.8E-16 | 14 | 0.1522 |
| ko05134 | Legionellosis | -15.2888496 | 5.1E-16 | 12 | 0.2182 |
| hsa04012 | ErbB signaling pathway | -15.2827463 | 5.2E-16 | 14 | 0.1489 |
| hsa05132 | Salmonella infection | -15.2157462 | 6.1E-16 | 14 | 0.1474 |
| ko04015 | Rap1 signaling pathway | -15.1566446 | 7E-16 | 18 | 0.0857 |
| hsa05014 | Amyotrophic lateral sclerosis | -15.0859337 | 8.2E-16 | 12 | 0.2105 |
| hsa04658 | Th1 and Th2 cell differentiation | -15.019364 | 9.6E-16 | 14 | 0.1429 |
| ko04370 | VEGF signaling pathway | -14.8909912 | 1.3E-15 | 12 | 0.2034 |
| hsa05134 | Legionellosis | -14.8909912 | 1.3E-15 | 12 | 0.2034 |
| hsa04215 | Apoptosis - multiple species | -14.4204124 | 3.8E-15 | 10 | 0.303 |
| ko04215 | Apoptosis - multiple species | -14.4204124 | 3.8E-15 | 10 | 0.303 |
| ko04024 | cAMP signaling pathway | -14.3484849 | 4.5E-15 | 17 | 0.0859 |
| hsa04370 | VEGF signaling pathway | -14.3483868 | 4.5E-15 | 12 | 0.1846 |
| hsa05168 | Herpes simplex virus 1 infection | -14.2308265 | 5.9E-15 | 25 | 0.0446 |
| hsa04062 | Chemokine signaling pathway | -14.2034721 | 6.3E-15 | 17 | 0.0842 |
| ko04920 | Adipocytokine signaling pathway | -14.0171932 | 9.6E-15 | 12 | 0.1739 |
| hsa05221 | Acute myeloid leukemia | -13.9377641 | 1.2E-14 | 12 | 0.1714 |
| hsa05203 | Viral carcinogenesis | -13.8885045 | 1.3E-14 | 17 | 0.0806 |
| ko04662 | B cell receptor signaling pathway | -13.8595987 | 1.4E-14 | 12 | 0.169 |
| ko04722 | Neurotrophin signaling pathway | -13.8081351 | 1.6E-14 | 14 | 0.1176 |
| hsa04920 | Adipocytokine signaling pathway | -13.7826584 | 1.6E-14 | 12 | 0.1667 |
| ko04152 | AMPK signaling pathway | -13.7564987 | 1.8E-14 | 14 | 0.1167 |
| hsa04722 | Neurotrophin signaling pathway | -13.5546096 | 2.8E-14 | 14 | 0.1129 |
| hsa04152 | AMPK signaling pathway | -13.3121434 | 4.9E-14 | 14 | 0.1085 |
| hsa04662 | B cell receptor signaling pathway | -13.1397423 | 7.2E-14 | 12 | 0.1481 |
| ko05203 | Viral carcinogenesis | -13.0145975 | 9.7E-14 | 16 | 0.0796 |
| hsa04217 | necroptosis | -12.9626197 | 1.1E-13 | 15 | 0.0893 |
| hsa04211 | Longevity regulating pathway | -12.9346455 | 1.2E-13 | 13 | 0.1193 |
| ko05230 | Central carbon metabolism in cancer | -12.7527529 | 1.8E-13 | 11 | 0.1692 |
| hsa04915 | Estrogen signaling pathway | -12.5593237 | 2.8E-13 | 14 | 0.0959 |
| ko05120 | Epithelial cell signaling in Helicobacter pylori infection | -12.5257674 | 3E-13 | 11 | 0.1618 |
| ko04664 | Fc epsilon RI signaling pathway | -12.5257674 | 3E-13 | 11 | 0.1618 |
| ko04062 | Chemokine signaling pathway | -12.4503889 | 3.5E-13 | 15 | 0.0824 |
| hsa05120 | Epithelial cell signaling in Helicobacter pylori infection | -12.3097097 | 4.9E-13 | 11 | 0.1549 |
| hsa04910 | Insulin signaling pathway | -12.1990497 | 6.3E-13 | 14 | 0.0903 |
| ko04915 | Estrogen signaling pathway | -12.1176775 | 7.6E-13 | 12 | 0.1224 |
| ko05221 | Acute myeloid leukemia | -11.9698844 | 1.1E-12 | 10 | 0.1818 |
| hsa05230 | Central carbon metabolism in cancer | -11.8428079 | 1.4E-12 | 11 | 0.141 |
| hsa04664 | Fc epsilon RI signaling pathway | -11.7179262 | 1.9E-12 | 11 | 0.1375 |
| ko04910 | Insulin signaling pathway | -11.5976008 | 2.5E-12 | 13 | 0.0942 |
| hsa04610 | Complement and coagulation cascades | -11.1958121 | 6.4E-12 | 11 | 0.1236 |
| hsa04726 | Serotonergic synapse | -11.0972676 | 8E-12 | 12 | 0.1008 |
| ko04150 | mTOR signaling pathway | -11.0960261 | 8E-12 | 13 | 0.0861 |
| ko04110 | Cell cycle | -10.8836032 | 1.3E-11 | 12 | 0.0968 |
| ko04622 | RIG-I-like receptor signaling pathway | -10.8710268 | 1.3E-11 | 10 | 0.1429 |
| hsa04150 | mTOR signaling pathway | -10.8453641 | 1.4E-11 | 13 | 0.0823 |
| hsa04622 | RIG-I-like receptor signaling pathway | -10.7446242 | 1.8E-11 | 10 | 0.1389 |
| ko05231 | Choline metabolism in cancer | -10.6801797 | 2.1E-11 | 11 | 0.1111 |
| ko04022 | cGMP-PKG signaling pathway | -10.6737353 | 2.1E-11 | 13 | 0.0798 |
| hsa04110 | Cell cycle | -10.5607938 | 2.7E-11 | 12 | 0.0909 |
| hsa04140 | Autophagy - animal | -10.5607938 | 2.7E-11 | 12 | 0.0909 |
| hsa04022 | cGMP-PKG signaling pathway | -10.3473456 | 4.5E-11 | 13 | 0.0751 |
| ko04610 | Complement and coagulation cascades | -10.3310255 | 4.7E-11 | 10 | 0.1266 |
| hsa05231 | Choline metabolism in cancer | -10.3074833 | 4.9E-11 | 11 | 0.1028 |
| hsa04923 | Regulation of lipolysis in adipocytes | -10.1716549 | 6.7E-11 | 9 | 0.1552 |
| hsa05211 | Renal cell carcinoma | -10.1660248 | 6.8E-11 | 10 | 0.122 |
| hsa05216 | Thyroid cancer | -10.1266118 | 7.5E-11 | 8 | 0.2051 |
| ko04020 | Calcium signaling pathway | -10.0710282 | 8.5E-11 | 13 | 0.0714 |
| hsa04650 | Natural killer cell mediated cytotoxicity | -9.97554541 | 1.1E-10 | 12 | 0.0811 |
| ko04921 | Oxytocin signaling pathway | -9.84015364 | 1.4E-10 | 12 | 0.0789 |
| hsa05010 | Alzheimer disease | -9.67009865 | 2.1E-10 | 13 | 0.0663 |
| hsa04921 | Oxytocin signaling pathway | -9.54944604 | 2.8E-10 | 12 | 0.0745 |
| hsa05332 | Graft-versus-host disease | -9.5145973 | 3.1E-10 | 8 | 0.1739 |
| ko04140 | Autophagy - animal | -9.45941335 | 3.5E-10 | 11 | 0.0859 |
| hsa04940 | Type I diabetes mellitus | -9.43577395 | 3.7E-10 | 8 | 0.1702 |
| hsa05330 | Allograft rejection | -9.43577395 | 3.7E-10 | 8 | 0.1702 |
| hsa05416 | Viral myocarditis | -9.41250835 | 3.9E-10 | 9 | 0.1286 |
| hsa04020 | Calcium signaling pathway | -9.40281658 | 4E-10 | 13 | 0.0631 |
| ko04650 | Natural killer cell mediated cytotoxicity | -9.28012894 | 5.2E-10 | 11 | 0.0827 |
| hsa04924 | Renin secretion | -9.2451775 | 5.7E-10 | 9 | 0.1233 |
| hsa01523 | Antifolate resistance | -9.23964717 | 5.8E-10 | 7 | 0.2258 |
| ko01523 | Antifolate resistance | -9.23964717 | 5.8E-10 | 7 | 0.2258 |
| hsa04913 | Ovarian steroidogenesis | -8.93157528 | 1.2E-09 | 8 | 0.1481 |
| hsa04371 | Apelin signaling pathway | -8.84686411 | 1.4E-09 | 11 | 0.0753 |
| hsa04640 | Hematopoietic cell lineage | -8.77249057 | 1.7E-09 | 10 | 0.0885 |
| hsa04928 | parathyroid hormone synthesis, secretion and action | -8.77249057 | 1.7E-09 | 10 | 0.0885 |
| ko04670 | Leukocyte transendothelial migration | -8.73485114 | 1.8E-09 | 10 | 0.0877 |
| hsa04725 | Cholinergic synapse | -8.69757465 | 2E-09 | 10 | 0.087 |
| ko05330 | Allograft rejection | -8.57378501 | 2.7E-09 | 7 | 0.1842 |
| hsa04670 | Leukocyte transendothelial migration | -8.48118238 | 3.3E-09 | 10 | 0.0826 |
| ko04623 | Cytosolic DNA-sensing pathway | -8.38064826 | 4.2E-09 | 8 | 0.127 |
| hsa04072 | Phospholipase D signaling pathway | -8.34084132 | 4.6E-09 | 11 | 0.0675 |
| ko04912 | GnRH signaling pathway | -8.33565584 | 4.6E-09 | 9 | 0.0978 |
| ko05332 | Graft-versus-host disease | -8.32958362 | 4.7E-09 | 7 | 0.1707 |
| hsa04623 | Cytosolic DNA-sensing pathway | -8.32486491 | 4.7E-09 | 8 | 0.125 |
| ko05211 | Renal cell carcinoma | -8.27003493 | 5.4E-09 | 8 | 0.1231 |
| ko04728 | Dopaminergic synapse | -8.17790688 | 6.6E-09 | 10 | 0.0769 |
| ko04940 | Type I diabetes mellitus | -8.17761201 | 6.6E-09 | 7 | 0.1628 |
| ko04640 | Hematopoietic cell lineage | -8.13058889 | 7.4E-09 | 9 | 0.0928 |
| hsa04934 | cushing syndrome | -8.12268956 | 7.5E-09 | 11 | 0.0643 |
| hsa04080 | Neuroactive ligand-receptor interaction | -8.11675557 | 7.6E-09 | 15 | 0.0405 |
| hsa04728 | Dopaminergic synapse | -8.11366987 | 7.7E-09 | 10 | 0.0758 |
| hsa05131 | Shigellosis | -8.11096363 | 7.7E-09 | 8 | 0.1176 |
| hsa04912 | GnRH signaling pathway | -8.09097503 | 8.1E-09 | 9 | 0.0918 |
| ko04930 | Type II diabetes mellitus | -7.96380145 | 1.1E-08 | 7 | 0.1522 |
| hsa05016 | Huntington disease | -7.96078239 | 1.1E-08 | 12 | 0.0541 |
| ko04080 | Neuroactive ligand-receptor interaction | -7.84633828 | 1.4E-08 | 13 | 0.0469 |
| ko04261 | Adrenergic signaling in cardiomyocytes | -7.74962332 | 1.8E-08 | 10 | 0.0694 |
| ko05216 | Thyroid cancer | -7.74243641 | 1.8E-08 | 6 | 0.2069 |
| ko04913 | Ovarian steroidogenesis | -7.70160631 | 2E-08 | 7 | 0.14 |
| ko04072 | Phospholipase D signaling pathway | -7.69223721 | 2E-08 | 10 | 0.0685 |
| hsa04261 | Adrenergic signaling in cardiomyocytes | -7.66387142 | 2.2E-08 | 10 | 0.068 |
| ko05204 | Chemical carcinogenesis | -7.45867821 | 3.5E-08 | 8 | 0.0976 |
| hsanan01 | drug metabolism | -7.41317683 | 3.9E-08 | 9 | 0.0769 |
| hsa04930 | Type II diabetes mellitus | -7.24049938 | 5.7E-08 | 7 | 0.1207 |
| hsa05020 | Prion disease | -7.22172265 | 6E-08 | 6 | 0.1714 |
| ko05020 | Prion diseases | -7.22172265 | 6E-08 | 6 | 0.1714 |
| ko04540 | Gap junction | -7.21582013 | 6.1E-08 | 8 | 0.0909 |
| hsa05204 | Chemical carcinogenesis | -7.17712174 | 6.7E-08 | 8 | 0.0899 |
| hsa04540 | Gap junction | -7.13889961 | 7.3E-08 | 8 | 0.0889 |
| ko04960 | Aldosterone-regulated sodium reabsorption | -7.07001195 | 8.5E-08 | 6 | 0.1622 |
| hsa04611 | Platelet activation | -7.04515591 | 9E-08 | 9 | 0.0698 |
| hsa04213 | Longevity regulating pathway - multiple species | -7.03555318 | 9.2E-08 | 7 | 0.1129 |
| ko04213 | Longevity regulating pathway - multiple species | -7.03555318 | 9.2E-08 | 7 | 0.1129 |
| hsa04960 | Aldosterone-regulated sodium reabsorption | -6.92713759 | 1.2E-07 | 6 | 0.1538 |
| ko04924 | Renin secretion | -6.89116615 | 1.3E-07 | 7 | 0.1077 |
| ko04137 | Mitophagy - animal | -6.89116615 | 1.3E-07 | 7 | 0.1077 |
| hsa04137 | Mitophagy - animal | -6.79892073 | 1.6E-07 | 7 | 0.1045 |
| ko04550 | Signaling pathways regulating pluripotency of stem cells | -6.76642506 | 1.7E-07 | 9 | 0.0647 |
| ko05031 | Amphetamine addiction | -6.75392651 | 1.8E-07 | 7 | 0.1029 |
| hsa04914 | Progesterone-mediated oocyte maturation | -6.68082309 | 2.1E-07 | 8 | 0.0777 |
| hsa00982 | Drug metabolism - cytochrome P450 | -6.66608097 | 2.2E-07 | 7 | 0.1 |
| ko00982 | Drug metabolism - cytochrome P450 | -6.66608097 | 2.2E-07 | 7 | 0.1 |
| hsa05031 | Amphetamine addiction | -6.6231871 | 2.4E-07 | 7 | 0.0986 |
| ko00980 | Metabolism of xenobiotics by cytochrome P450 | -6.49838491 | 3.2E-07 | 7 | 0.0946 |
| hsa00980 | Metabolism of xenobiotics by cytochrome P450 | -6.41823599 | 3.8E-07 | 7 | 0.0921 |
| hsa04550 | Signaling pathways regulating pluripotency of stem cells | -6.41169751 | 3.9E-07 | 9 | 0.0588 |
| ko04672 | Intestinal immune network for IgA production | -6.31661844 | 4.8E-07 | 6 | 0.1224 |
| ko04144 | Endocytosis | -6.27047883 | 5.4E-07 | 11 | 0.0423 |
| ko04810 | Regulation of actin cytoskeleton | -6.17630722 | 6.7E-07 | 10 | 0.0472 |
| hsa04144 | Endocytosis | -6.09328097 | 8.1E-07 | 11 | 0.0406 |
| ko05010 | Alzheimer's disease | -6.00573983 | 9.9E-07 | 9 | 0.0526 |
| ko05310 | Asthma | -5.95934371 | 1.1E-06 | 5 | 0.1613 |
| ko04666 | Fc gamma R-mediated phagocytosis | -5.88250528 | 1.3E-06 | 7 | 0.0769 |
| hsa04672 | Intestinal immune network for IgA production | -5.8300002 | 1.5E-06 | 6 | 0.1017 |
| hsa04810 | Regulation of actin cytoskeleton | -5.77094428 | 1.7E-06 | 10 | 0.0426 |
| ko04914 | Progesterone-mediated oocyte maturation | -5.72532977 | 1.9E-06 | 7 | 0.0729 |
| hsa05320 | Autoimmune thyroid disease | -5.61960931 | 2.4E-06 | 6 | 0.0938 |
| ko04310 | Wnt signaling pathway | -5.59450885 | 2.5E-06 | 8 | 0.0559 |
| ko04916 | Melanogenesis | -5.57696349 | 2.6E-06 | 7 | 0.0693 |
| ko05016 | Huntington's disease | -5.57071024 | 2.7E-06 | 9 | 0.0466 |
| hsa04916 | Melanogenesis | -5.46401833 | 3.4E-06 | 7 | 0.0667 |
| hsa04666 | Fc gamma R-mediated phagocytosis | -5.4365279 | 3.7E-06 | 7 | 0.066 |
| hsa04922 | Glucagon signaling pathway | -5.40932424 | 3.9E-06 | 7 | 0.0654 |
| ko04390 | Hippo signaling pathway | -5.35496031 | 4.4E-06 | 8 | 0.0519 |
| hsa05310 | Asthma | -5.33590896 | 4.6E-06 | 5 | 0.122 |
| ko04520 | Adherens junction | -5.31793214 | 4.8E-06 | 6 | 0.0833 |
| hsa04976 | Bile secretion | -5.21419595 | 6.1E-06 | 6 | 0.08 |
| hsa04310 | Wnt signaling pathway | -5.05780231 | 8.8E-06 | 8 | 0.0473 |
| hsa04520 | Adherens junction | -5.01978864 | 9.6E-06 | 6 | 0.0741 |
| hsa04390 | Hippo signaling pathway | -4.96536854 | 1.1E-05 | 8 | 0.046 |
| hsa04530 | Tight junction | -4.8759232 | 1.3E-05 | 8 | 0.0447 |
| ko05320 | Autoimmune thyroid disease | -4.77802282 | 1.7E-05 | 5 | 0.0943 |
| hsa04270 | Vascular smooth muscle contraction | -4.70394475 | 2E-05 | 7 | 0.0511 |
| hsa04911 | Insulin secretion | -4.70153932 | 2E-05 | 6 | 0.0652 |
| hsa05030 | Cocaine addiction | -4.69863495 | 2E-05 | 5 | 0.0909 |
| ko04972 | Pancreatic secretion | -4.59614945 | 2.5E-05 | 6 | 0.0625 |
| ko04730 | Long-term depression | -4.51324856 | 3.1E-05 | 5 | 0.0833 |
| hsa04713 | circadian entrainment | -4.49553491 | 3.2E-05 | 6 | 0.06 |
| ko04723 | Retrograde endocannabinoid signaling | -4.4710813 | 3.4E-05 | 6 | 0.0594 |
| hsa04730 | Long-term depression | -4.44378894 | 3.6E-05 | 5 | 0.0806 |
| ko04922 | Glucagon signaling pathway | -4.42297465 | 3.8E-05 | 6 | 0.0583 |
| hsa04972 | Pancreatic secretion | -4.42297465 | 3.8E-05 | 6 | 0.0583 |
| ko00830 | Retinol metabolism | -4.34408298 | 4.5E-05 | 5 | 0.0769 |
| hsa00830 | Retinol metabolism | -4.2803823 | 5.2E-05 | 5 | 0.0746 |
| ko04976 | Bile secretion | -4.15903576 | 6.9E-05 | 5 | 0.0704 |
| ko04530 | Tight junction | -4.10628438 | 7.8E-05 | 7 | 0.0412 |
| ko04971 | Gastric acid secretion | -4.04500427 | 9E-05 | 5 | 0.0667 |
| ko04270 | Vascular smooth muscle contraction | -4.03211231 | 9.3E-05 | 6 | 0.0496 |
| ko00380 | Tryptophan metabolism | -4.01489018 | 9.7E-05 | 4 | 0.1 |
| hsa05012 | Parkinson disease | -3.99663664 | 0.0001 | 7 | 0.0395 |
| hsa04971 | Gastric acid secretion | -3.99048227 | 0.0001 | 5 | 0.0649 |
| hsa04114 | Oocyte meiosis | -3.86058041 | 0.00014 | 6 | 0.0462 |
| ko04350 | TGF-beta signaling pathway | -3.81131833 | 0.00015 | 5 | 0.0595 |
| hsa05410 | Hypertrophic cardiomyopathy | -3.73955048 | 0.00018 | 5 | 0.0575 |
| ko04970 | Salivary secretion | -3.67048919 | 0.00021 | 5 | 0.0556 |
| ko05030 | Cocaine addiction | -3.66794566 | 0.00021 | 4 | 0.0816 |
| hsa04340 | Hedgehog signaling pathway | -3.66794566 | 0.00021 | 4 | 0.0816 |
| hsa00380 | Tryptophan metabolism | -3.63375819 | 0.00023 | 4 | 0.08 |
| ko00330 | Arginine and proline metabolism | -3.63375819 | 0.00023 | 4 | 0.08 |
| ko04514 | Cell adhesion molecules (CAMs) | -3.61898216 | 0.00024 | 6 | 0.0417 |
| hsa04350 | TGF-beta signaling pathway | -3.60395267 | 0.00025 | 5 | 0.0538 |
| hsa04970 | Salivary secretion | -3.58230606 | 0.00026 | 5 | 0.0532 |
| hsa04514 | Cell adhesion molecules | -3.49273597 | 0.00032 | 6 | 0.0395 |
| hsa05414 | Dilated cardiomyopathy | -3.477813 | 0.00033 | 5 | 0.0505 |
| ko05150 | Staphylococcus aureus infection | -3.44318366 | 0.00036 | 4 | 0.0714 |
| ko00140 | Steroid hormone biosynthesis | -3.3845944 | 0.00041 | 4 | 0.069 |
| hsa04750 | inflammatory mediator regulation of trp channels | -3.37909443 | 0.00042 | 5 | 0.0481 |
| hsa00140 | Steroid hormone biosynthesis | -3.30075008 | 0.0005 | 4 | 0.0656 |
| hsa04723 | Retrograde endocannabinoid signaling | -3.27555332 | 0.00053 | 6 | 0.0359 |
| hsa00330 | Arginine and proline metabolism | -3.22132903 | 0.0006 | 4 | 0.0625 |
| ko04720 | Long-term potentiation | -3.1459156 | 0.00071 | 4 | 0.0597 |
| hsa04720 | Long-term potentiation | -3.0976868 | 0.0008 | 4 | 0.058 |
| hsa05217 | Basal cell carcinoma | -3.05098355 | 0.00089 | 4 | 0.0563 |
| hsa05034 | Alcoholism | -3.0432802 | 0.00091 | 6 | 0.0324 |
| ko03320 | PPAR signaling pathway | -3.0281766 | 0.00094 | 4 | 0.0556 |
| ko04918 | Thyroid hormone synthesis | -2.98360132 | 0.00104 | 4 | 0.0541 |
| ko04612 | Antigen processing and presentation | -2.91919488 | 0.0012 | 4 | 0.0519 |
| ko00350 | Tyrosine metabolism | -2.91842339 | 0.00121 | 3 | 0.0857 |
| hsa04918 | Thyroid hormone synthesis | -2.8777946 | 0.00132 | 4 | 0.0506 |
| hsa05150 | Staphylococcus aureus infection | -2.85753185 | 0.00139 | 4 | 0.05 |
| hsa04612 | Antigen processing and presentation | -2.81784447 | 0.00152 | 4 | 0.0488 |
| ko05410 | Hypertrophic cardiomyopathy (HCM) | -2.79840599 | 0.00159 | 4 | 0.0482 |
| ko04146 | Peroxisome | -2.79840599 | 0.00159 | 4 | 0.0482 |
| hsa03320 | PPAR signaling pathway | -2.74163347 | 0.00181 | 4 | 0.0465 |
| hsa05322 | Systemic lupus erythematosus | -2.71765738 | 0.00192 | 5 | 0.0342 |
| ko05414 | Dilated cardiomyopathy | -2.66931553 | 0.00214 | 4 | 0.0444 |
| ko05032 | Morphine addiction | -2.65180287 | 0.00223 | 4 | 0.044 |
| hsa05032 | Morphine addiction | -2.63450682 | 0.00232 | 4 | 0.0435 |
| hsa04146 | Peroxisome | -2.63450682 | 0.00232 | 4 | 0.0435 |
| ko04973 | Carbohydrate digestion and absorption | -2.62946176 | 0.00235 | 3 | 0.0682 |
| hsa00350 | Tyrosine metabolism | -2.60140116 | 0.0025 | 3 | 0.0667 |
| hsa04216 | Ferroptosis | -2.57401386 | 0.00267 | 3 | 0.0652 |
| ko04070 | Phosphatidylinositol signaling system | -2.5511139 | 0.00281 | 4 | 0.0412 |
| ko04340 | Hedgehog signaling pathway | -2.54727022 | 0.00284 | 3 | 0.0638 |
| ko04961 | Endocrine and other factor-regulated calcium reabsorption | -2.54727022 | 0.00284 | 3 | 0.0638 |
| hsa04961 | Endocrine and other factor-regulated calcium reabsorption | -2.47063244 | 0.00338 | 3 | 0.06 |
| hsa04070 | Phosphatidylinositol signaling system | -2.45728459 | 0.00349 | 4 | 0.0388 |
| hsa04973 | Carbohydrate digestion and absorption | -2.39888681 | 0.00399 | 3 | 0.0566 |
| ko04360 | Axon guidance | -2.37932264 | 0.00418 | 5 | 0.0286 |
| ko00480 | Glutathione metabolism | -2.37596095 | 0.00421 | 3 | 0.0556 |
| ko05034 | Alcoholism | -2.32778941 | 0.0047 | 5 | 0.0278 |
| hsa04714 | thermogenesis | -2.21176516 | 0.00614 | 6 | 0.0221 |
| ko00590 | Arachidonic acid metabolism | -2.2079231 | 0.0062 | 3 | 0.0484 |
| hsa00590 | Arachidonic acid metabolism | -2.18862657 | 0.00648 | 3 | 0.0476 |
| hsa00480 | Glutathione metabolism | -2.18862657 | 0.00648 | 3 | 0.0476 |
| hsa04360 | Axon guidance | -2.17390943 | 0.0067 | 5 | 0.0255 |
| ko04114 | Oocyte meiosis | -2.17258344 | 0.00672 | 4 | 0.0323 |
| ko00010 | Glycolysis / Gluconeogenesis | -2.11472205 | 0.00768 | 3 | 0.0448 |
| ko05322 | Systemic lupus erythematosus | -2.06736695 | 0.00856 | 4 | 0.0301 |
| ko00562 | Inositol phosphate metabolism | -2.04560526 | 0.009 | 3 | 0.0423 |
